# Spoilage Indicators, Bacterial Dynamics, and Metabolomic Profiles of Chilled Yak Meat Under Tray, Vacuum, and Modified-Atmosphere Packaging: A Comparative Study with Integrated Multi-Omics Focused on Tray Versus MAP Conditions

**DOI:** 10.3390/foods15162903

**Published:** 2026-08-19

**Authors:** Zhoulin Wu, Sha Zhu, Shuo Wen, Jiamin Zhang, Xinhui Wang, Yongjie Wang

**Affiliations:** 1 Meat Processing Key Laboratory of Sichuan Province, College of Food and Biological Engineering, Chengdu University, Chengdu 610106, China; wuzhoulin@cdu.edu.cn (Z.W.);; 2 Department of Animal Science, College of Agriculture and Environmental Science, North Carolina Agricultural and Technical State University, Greensboro, NC 27411, USA

**Keywords:** yak meat, modified-atmosphere packaging, spoilage, microbial community, metabolome profile

## Abstract

Yak meat faces limitations in the packaging methods available to extend its shelf life and maintain quality. This study compared the effects of tray packaging (YM), vacuum packaging (YZ) and 70% O_2_/30% CO_2_ modified-atmosphere packaging (YP) on the extension efficiency of yak *longissimus dorsi* muscles stored at 4 °C. Physicochemical parameters and total viable counts (TVCs) were measured for all three groups, while 16S rRNA sequencing and untargeted metabolomics were performed only on YM and YP samples to explore the mechanisms underlying quality differences. YP treatment significantly delayed the pH increase and color deterioration compared with YM and YZ. Furthermore, YP samples exhibited lower increases in TVB-N, TVC, and drip loss than YZ and YM samples during storage. The genera *Brochothrix*, *Macrococcus*, *Photobacterium*, *Delftia*, *Acinetobacter*, *Pseudomonas*, and *Lactococcus* were considered predominant spoilage organisms of yak meat. Notably, *Lactococcus* and *Pseudomonas* showed a significantly decreased proportional representation in YP samples over the storage period. Through untargeted metabolomics, a total of 818 metabolites were identified. Several pathways, including 2-oxocarboxylic acid metabolism, histidine metabolism, biosynthesis of amino acids, and D-amino acid metabolism, were identified as the main pathways influencing the metabolic differences between the YP and YM samples near spoiled stages. Pearson correlation analysis showed that bacterial genera such as *Macrococcus*, *Photobacterium*, *Pseudomonas*, *Lactococcus*, and *Enhydrobacter* were significantly correlated with key differential metabolites. Furthermore, 1-methylhistidine, 3-methylhistidine, alpha-ketoglutaric acid, PC 34:2, ergothioneine, anserine, LPC 16:0, and L-methionine sulfone were positively correlated with predominant spoilage microorganisms. These findings enhance our understanding of the spoilage characteristics of yak meat stored under the most prevalent commercial option (tray packaging) and the most promising modified-atmosphere packaging alternative (70% O_2_/30% CO_2_ MAP).

## 1. Introduction

Yak meat is a nutrient-rich specialty meat characterized by low fat (~1%), high protein (~21%), and abundant essential amino acids, polyunsaturated fatty acids, and minerals [1]. It provides a stable food source for people indigenous to the Qinghai–Tibetan Plateau. Furthermore, it is considered healthy and organic due to the semi-domesticated nature of yak grazing. Recently, owing to the increasing focus on nutritional properties and health-conscious choices, the yak meat industry has experienced significant commercial expansion in China and abroad [2]. However, yak production faces complex challenges due to a narrow profit margin in farming. This is largely because yak meat has effectively limited access to distant markets [1]. These hurdles include the remoteness of core production regions, such as the Qinghai–Tibetan Plateau, which often lack adequate transport networks. Additionally, fresh yak meat is susceptible to contamination and spoilage during chilling storage, and at present, effective preservation methods are lacking.

Commercially, tray packaging, vacuum packaging, and modified-atmosphere packaging (MAP), combined with low temperature, have been widely used for the presentation of primal cuts of meat [3]. Tray packaging is convenient and has a low cost, but is limited by its relatively short shelf life [4]. Meanwhile, vacuum packaging creates an anaerobic environment that can help to control the growth of spoilage microorganisms in fresh meat, but it can also result in increasing meat juice loss and in an undesirable purple color [5,6]. Relatively, MAP alters the gas mixture, which not only prolongs the shelf life but also helps preserve the desired meat color [7]. Of note, high-oxygen MAP, which typically consists of 70~80% oxygen and 20~30% carbon dioxide, is more effective at preserving the color compared to that of ordinary packaging [8,9]. Given that tray packaging is currently the most prevalent commercial option and MAP shows the greatest potential for optimizing yak meat quality, both systems merit further systematic study.

The deterioration and spoilage of fresh meat quality are complex microbial processes. With the extension of storage time, the microbial profile evolves toward lower richness and diversity due to the predominance of a few spoilage organism species [10]. The following genera are frequently found to contribute closely to meat spoilage: *Pseudomonas*, *Carnobacterium*, *Brochothrix*, and *Lactobacillus*. Generally, the packaging method leads to different types of microorganisms, with certain types usually predominant. For instance, some facultative anaerobes, such as *Brochothrix thermosphacta*, were found to be dominant in meat products [11,12]. Simultaneously, *Pseudomonas* dominated the bacterial communities stored under aerobic packaging, whereas *Enterobacteriaceae* and *Lactobacilli* were under vacuum packaging [11,12]. Under MAP presentation conditions, high oxygen content was found increase the abundance of *Pseudomonas* spp. and *Lactobacillus sakei* in beef [13]. However, the microbiota can vary significantly depending on gas composition, and different bacterial species can exhibit diverse metabolic activities [13,14]. Notably, the physicochemical quality, natural microbiome, and processing methods of meat vary across animal species [15], and the microbial profiles of yak and beef under MAP conditions are likely to differ accordingly.

Current studies mostly focus on the influences of microflora on the shelf-life of yak meat. Accordingly, high-throughput sequencing has been widely used to examine alterations in bacterial communities during the storage. To date, only limited studies in the literature have monitored the freshness of yak meat under tray packaging and MAP. Furthermore, relying solely on microbial analysis may not be sufficient to fully understand the microbiological processes. As a powerful complement to conventional microbial analysis, non-target metabolomic techniques allows for the holistic evaluation of metabolic process changes in meat during storage [16]. In this study, the effects of different packaging on quality indicators of yak meat samples were investigated. Subsequently, bacterial community dynamics in yak meat samples during chilling storage under tray and modified-atmosphere conditions were evaluated using high-throughput sequencing, and metabolomic profiling was performed using ultra-high-performance liquid chromatography–mass spectrometry (LC-MS/MS). Furthermore, the interactions among bacterial communities and their correlations with metabolites were explored. The results will help to understand the development of microbial and metabolic characteristics in yak meat under tray packaging and MAP, and provide valuable references for subsequent meat storage.

## 2. Materials and Methods

### 2.1. Ethical Statement

**Figure 1:**
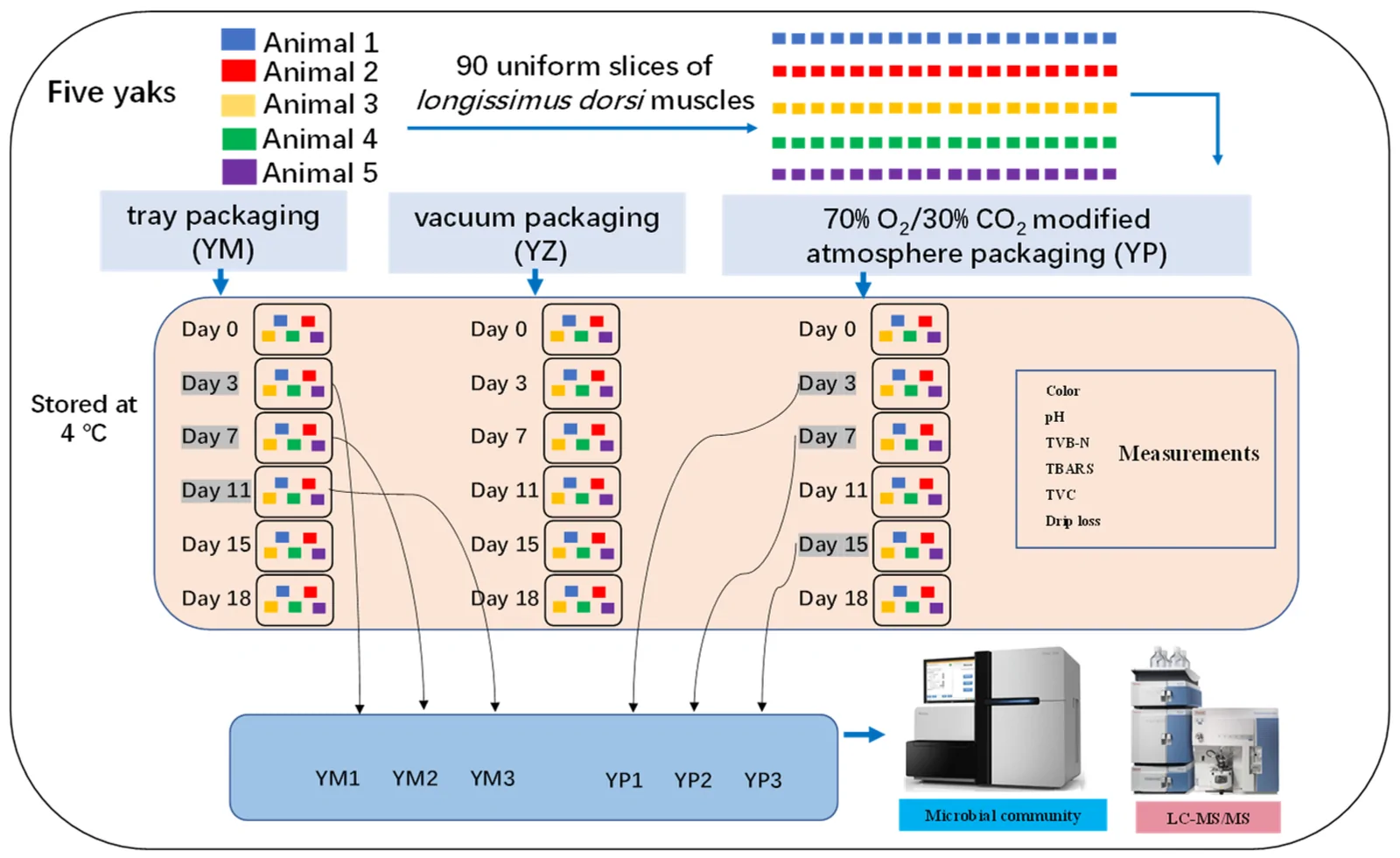
Scheme of the work procedure adopted.

Five yak steers (~24 months of age) grazing naturally in Hongyuan, Aba, Sichuan, China, were used in this study. The animals were transferred to a local abattoir and fasted for 24 h with free access to water before slaughter. After slaughter, the carcasses were chilled in air (~10 °C) within 2 h and then aged for 24 h at 4 °C. Subsequently, the *longissimus dorsi* muscles were excised, placed in sterile bags, transported to the laboratory in chilled containers with ice packs, and processed within 12 h for further analysis, as illustrated in Figure 1. Given that sampling was carried out as part of routine commercial slaughter, with no additional invasive steps, the study was exempt from formal ethical approval.

### 2.2. Meat Sample Preparation

After arrival, the samples were cleaned of visible fat and connective tissue. For each animal (*n* = 5), the loin was cut into 18 uniform slices (approximately 10 cm × 5 cm × 1.5 cm, weighing about 100 g), which were then randomly assigned to 18 treatment combinations (3 packaging treatments × 6 storage times) and packaged individually. These three packaging treatments were (1) vacuum packaging (YZ), (2) tray packaging (YM), and (3) modified-atmosphere packaging with 70% O_2_/30% CO_2_ (YP). This yielded five replicates per treatment combination and a total of 90 segments.

The YZ process was conducted with vacuum skin packaging at an absolute pressure of 100.0 ± 2.0 kPa, a sealing temperature of 140 °C, and a sealing time of 2.0 s. The packaging material was PA/PE, with an oxygen permeability rate of 2 cm^3^/(m^2^·24 h·0% RH) at 23 °C and a water vapor transmission rate of 4 g/(m^2^·24 h·90% RH) at 38 °C. For the YM treatment, samples were fully wrapped in polyvinyl chloride film and placed on polyethylene trays (22 cm × 13 cm × 4 cm). For the YP treatment, samples were placed on the same trays and sealed with polyethylene film (O_2_ permeability: 8.19 × 10^−4^ cm^3^/(m^2^·24 h·Pa); water vapor transmission rate: 6.43 g/(m^2^·24 h)) using a BVPJ-260 MAP machine (Jiaxing Aibo Industry, Zhejiang, China). The gas flushing procedure was performed as follows: the chamber was evacuated to a residual pressure of approximately 10 kPa, followed by flushing with the gas mixture (70% O_2_/30% CO_2_) for three cycles to ensure complete removal of residual air. The gas-to-product ratio was approximately 10.7 mL/g for the YP samples. The sealing conditions were set at a temperature of 140 °C, a pressure of 100.0 ± 2.0 kPa (absolute pressure), and a sealing time of 2.0 s.

### 2.3. Storage and Sampling

All packaged samples were stored at 4 °C for 0, 3, 7, 11, 15, and 18 days. In addition, the sampling times were adjusted according to the limit of 15 mg/100 g for fresh meat in the Chinese National Food Safety Standard (GB 2707–2016) [17] to account for expected differences in total volatile basic nitrogen (TVB-N) content. At each sampling time, meat was removed from the package for physicochemical analysis. Based on the widespread commercial application of tray packaging and the emerging potential of MAP for yak meat quality improvement, samples were collected at three time points for both packaging conditions (days 3, 7, and 11 for tray packaging; days 3, 7, and 15 for MAP) according to TVB-N results. The samples were designated as YM1, YM2, and YM3 (tray packaging) and YP1, YP2, and YP3 (MAP), corresponding to fresh, mid-fresh, and near-spoiled stages, respectively. Approximately 10 g of each sample was stored in a −80 °C freezer for subsequent 16S rRNA gene sequencing and metabolomics analyses.

### 2.4. pH, Color, and Drip Loss

The pH of meat samples was measured using a pH meter (Matthäus, Pöttmes, Germany) equipped with a penetrating electrode. The meter was calibrated using pH 4.0 and pH 7.0 standard buffer solutions prior to use. Triplicate measurements were taken by inserting the electrode approximately 2 cm into the meat sample, avoiding the fascia, and the mean values were calculated as the final pH parameters. The surface color of meat samples was determined using a colorimeter (Minolta CR-300) based on the CIE L*a*b* color system. Calibration was performed using white and black standard plates prior to measurements. For each sample, lightness (L*), redness (a*), and yellowness (b*) were randomly measured triplicates, and the mean values were calculated for each parameter. Drip loss of the samples was measured according to the guidelines provided by Choeun et al. [18] with slight modification. In brief, each sample (approximately 50 g) was accurately weighed, placed into a plastic bag, and suspended in a refrigerator at 4 °C for 24 h. After storage, the sample was blotted with filter paper to absorb water, and the final weight was recorded. Drip loss is expressed as a percentage of the initial sample weight.

### 2.5. Determination of TVB-N

TVB-N content was measured according to the method of previous work [19]. In brief, 20 g of minced meat was transferred to a dry, clean tapered flask, homogenized with 100 mL of distilled water for 30 min, and then passed through filter paper (Φ 15 cm). Next, 10 mL of the filtrate was mixed with 10 mL of MgO solution (10 g/L), and the mixture was subjected to distillation using a nitrogen analyzer (KDN-102C, Xianjian Instrument, Shanghai, China). The resulting distillate was trapped in a conical flask containing 20 mL of boric acid solution (20 g/L) with mixed indicator, followed by titration with HCl solution (0.01 mol/L). The TVB-N content was calculated and is expressed as mg per 100 g of meat.

### 2.6. Measurement of TBARS

The thiobarbituric acid reactive substance (TBARS) value was measured using a spectrophotometric method to assess lipid oxidation by quantifying the complex formed between thiobarbituric acid and malondialdehyde (MDA). Briefly, a minced meat sample weighing 5.0 g was transferred into a clean conical bottle, and then homogenized with 50.0 mL of trichloroacetic acid (TCA) solution on a constant-temperature oscillator at 50 °C for 30 min. Afterwards, the homogenate was cooled to room temperature and then centrifuged at 10,000× *g* for 15 min. The supernatant was filtered and a 5.0 mL aliquot of the filtrate was mixed with 5.0 mL of 0.02 M thiobarbituric acid (TBA) solution. The mixture was then heated in a boiling water bath at 90 °C for 40 min. After cooling to room temperature, the absorbance was measured at 532 nm using a spectrophotometer (Model 722, Shanghai Jinghua Technology Co., LTD., Shanghai, China). The result is expressed as mg malondialdehyde (MDA) per kilogram of meat.

### 2.7. Microbiological Analysis

The total viable count (TVC) was measured using the plate count method in accordance with the Chinese standard GB 4789.2-2022 [20]. Briefly, 25 g of meat sample was split into small pieces and homogenized with 225 mL of 0.89% sterile saline (1:9, m/v) in a side-filter bag for 2 min. Subsequently, a 10-fold dilution was prepared, and 100 μL of the appropriate concentration was inoculated onto the plate count agar plates (Difco, Detroit, MI, USA). At the same time, sterile saline was used as a control, and all of these plates were cultured at 37 °C for 48 h. Lastly, the colony counts were recorded and are expressed as colony-forming units (CFU/g).

### 2.8. Bacteriome Characterization in Meat Samples

To characterize the bacteriome present in yak meat samples and its fluctuations during 4 °C storage, 16S rRNA gene sequencing was conducted. Bacterial DNA was extracted from thawed meat samples, previously stored at −80 °C, using the TIANamp stool DNA isolation kit (Qiagen, Shanghai, China) following the manufacturer’s recommendations. The resulting DNA extracts were evaluated using a NanoDrop ND-1000 spectrophotometer (NanoDrop Technologies, Montchanin, DE, USA). PCR-amplification of the V3-V4 hypervariable regions of the 16S rRNA gene was performed with the following primers (forward: 5′-ACTCCTACGGGAGGCAGCA-3′, and reverse: 5′-GGACTACHVGGGTWTCTAAT-3′). Next, purified amplicons were pooled in equimolar amounts, and sequencing was performed on the Illumina HiSeq 2500 platform to generate 250 bp paired-end reads.

Sequencing data were analyzed using the Quantitative Insights into Microbial Ecology2 (QIIME2 v.2023.5) tool [21]. The obtained raw reads were preprocessed with Cutadapt software (1.9.1), removing primer sequences and discarding low-quality reads. Clean reads were then processed for demultiplexing, paired-end reads merging, and *de novo* operational taxonomic unit (OTU) picking. Next, the OTUs were aligned using the DEBLUR program [22], and taxonomic identity was assigned to each OTU by comparison against the Silva database (v.138) [23]. Based on the OTU information, alpha diversity was assessed using the Shannon and Chao1 indices. The Kruskal–Wallis test was applied to evaluate the statistical significance of differences in alpha diversity between YP and YM samples at different stages. Additionally, non-metric multidimensional scaling (NMDS) was employed to investigate the beta diversity of microbial communities based on Weighted UniFrac and Unweighted UniFrac matrices. Analysis of Similarities (ANOSIM) was applied to both distance matrices to assess the statistical significance of differences in beta diversity among groups, with *p* < 0.05 considered statistically significant. Both alpha and beta diversity visualizations were generated using the R package ggplot2 (v.3.5.0). Linear discriminant analysis effect size (LEfSe) was used to identify bacterial composition differences between the groups [24], using a logarithmic LDA score threshold of >2 to indicate significant discriminative features.

### 2.9. Non-Targeted Metabolomics Analysis

The metabolites in stored meat were detected using liquid chromatography–mass spectrometry (LC-MS)/MS technology according to the method of Wu et al. [25], with slight modifications. Approximately 50 mg of the meat samples was ground with liquid nitrogen, and the metabolites were extracted with 400 μL of 80% methanol–water solution containing 0.02 mg/mL of L-2-chlorophenylalanine as an internal standard. The homogenates were crushed for 30 min in an ice bath and then centrifuged at 13,000 rpm for 10 min at 4 °C. Subsequently, a supernatant aliquot was diluted in LC-MS-grade water to a final concentration of 53% methanol, then centrifuged at 15,000× *g* at 4 °C for 20 min. Finally, the supernatant was collected for LC-MS analysis. Quality control (QC) samples were prepared by pooling equal aliquots of each sample to monitor analytical deviations and system stability throughout the experiment. Blank sample was prepared by replacing the experimental samples with 53% methanol.

LC-MS/MS was conducted on a Vanquish UHPLC system (Thermo Fisher Scientific, Waltham, MA, USA) coupled with an Orbitrap Q Exactive HF-X mass spectrometer (Thermo Fisher Scientific, Waltham, MA, USA) system at Novogene Co., Ltd. (Beijing, China). A 5 μL sample was injected onto a Hypersil GOLD C18 column (100 × 2.1 mm, 1.9 μm) at 40 °C, with a flow rate of 0.2 mL/min. The mobile phases consisted of (A) 0.1% formic acid in water and (B) methanol. The LC gradient was programmed as follows: 0–1.5 min, 2% B; 1.5–3.0 min, 2–85% B; 3.0–10.0 min, 85–100% B; 10.0–10.1 min, 100–2% B; 10.1–12.0 min, 2% B. The eluents in the positive and negative polarity modes were 0.1% formic acid in water and methanol, respectively. The Q Exactive HF-X mass spectrometer was operated at a spray voltage of 3.5 kV in positive ion mode (ESI+) and −3.5 kV in negative ion mode (ESI−). Mass spectra were acquired over the m/z range of 100–1500; nitrogen was used as the sheath gas, with the flow rate set as 35 psi. The MS1 and MS2 resolutions were set to 60,000 FWHM and 15,000 FWHM, respectively. QC samples were injected at a frequency of one QC sample per 10 experimental samples. If the remaining number of samples in the final batch was fewer than 10, one QC sample was still added at the end of the run. Accordingly, a total of three QC samples were included in this study. Blank samples were injected consecutively for three runs. The first two injections were used to elute any residual compounds from the previous sample and to equilibrate the system, while the last injection was used for background subtraction in Compound Discoverer software (v.3.4) during library searching. Raw LC-MS/MS data were processed for peak alignment by Compound Discoverer 3.1 (CD3.1, Thermo Fisher) to perform peak alignment, peak picking, and quantitation for each metabolite. Missing values were imputed by default during the library search using the CD3.4 software, with the imputation rules detailed in the software manual. After data normalization, metabolic peaks with QC RSD >30% were filtered out using SIMCA-P (v14.1). Then, metabolite identification was performed using the mzVault, mzCloud, and Masslist databases. Matches confirmed by MS/MS spectral matching (mzVault and mzCloud) were assigned confidence level 2, whereas those identified solely by accurate mass matching (Masslist) were assigned level 3, according to the Metabolomics Standards Initiative (MSI) criteria. Differentially abundant metabolites (DAMs) were identified based on a Variable Importance in Projection (VIP) score > 1, fold-change > 1.2, and FDR-adjusted *p* < 0.05 (Benjamini–Hochberg correction). KEGG (https://www.genome.jp/kegg/pathway.html, accessed on 18 August 2026), HMDB (https://hmdb.ca/metabolites, accessed on 18 August 2026), and Metlin (https://metlin.scripps.edu, accessed on 18 August 2026) databases were used for metabolites annotation and pathway enrichment analysis.

### 2.10. Statistical Analysis

The results from each group are presented as the mean ± standard deviation (SD). For physicochemical parameters, linear mixed-effects regression models with packaging method, storage time, and their interaction as fixed effects, and carcass ID as a random intercept, were fitted using the “lme4” package in R (version 4.5.3). Denominator degrees of freedom and corresponding *p*-values were approximated using Satterthwaite’s method via the “lmerTest” package. To dissect the significant interaction, post hoc pairwise comparisons among packaging methods were performed at each storage time point using the “emmeans” package, with Tukey’s adjustment for multiple comparisons. Adjusted *p*-values < 0.05 were considered statistically significant. Correlations between selected bacterial genera and metabolites were calculated via Pearson’s correlation coefficient using R software (version 4.5.3), with significance set at |r| > 0.6 and FDR < 0.05, and visualized with the ggplot2 (version 3.5.0) package.

## 3. Results and Discussion

### 3.1. Changes in Meat Quality Attributes

**Figure 2:**
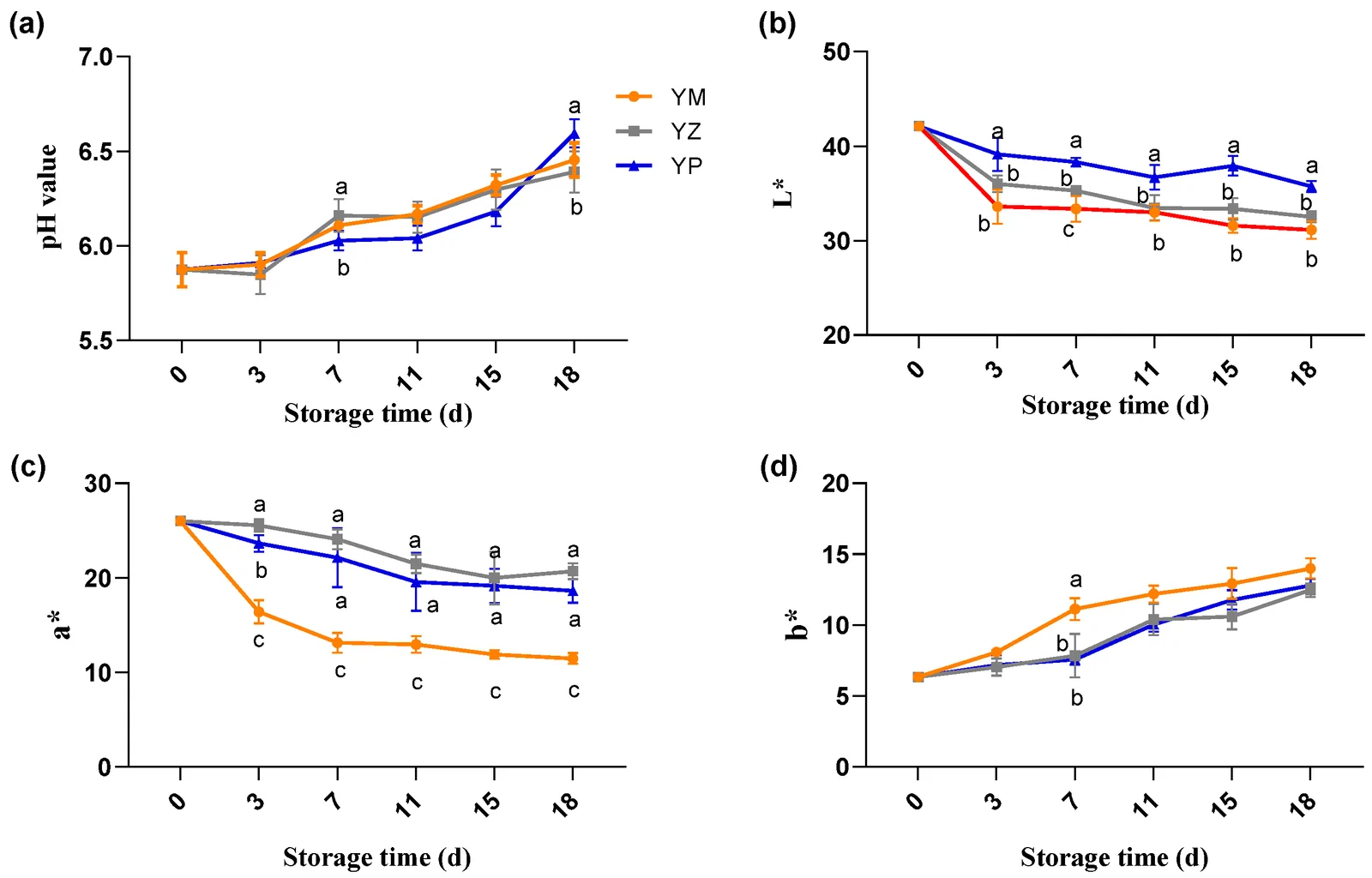
Changes in pH and color of yak meat samples over 18 days of chilled storage. Data are presented as mean ± SD (*n* = 5; error bars indicate SD). (**a**) pH, (**b**) L*, (**c**) a*, and (**d**) b* values. Statistical analysis was performed using linear mixed-effects regression models followed by Tukey’s adjustment for multiple comparisons. Different lowercase letters (a–c) above the bars at each storage time point indicate significant differences among packaging groups at *p* < 0.05.

In fresh meat and meat products, the pH value is always associated with postmortem metabolism, which exerts a significant impact on color, water retention, and shelf life [26]. A low-pH environment is associated with slower bacterial growth, but the accumulation of amines and ammonia during storage causes the pH of meat to rise [27]. The pH value of yak meat as affected by packaging methods and duration is presented in Figure 2a. The initial pH value of yak meat on day 0 was 5.87 ± 0.09, after which the pH value in all groups increased with the extension of storage time. Compared with the YM samples, the YP samples exhibited a relatively lower growth rate in pH value during the storage period. A lower pH can be attributed to limited proteolysis and reduced microbial growth, thereby resulting in fewer nitrogen-containing substances [28,29]. Bright-red color is a critical sensory attribute of meat freshness and quality. The L* value (Figure 2b) and a* value (Figure 2c) decreased with increasing storage time across all samples. It is worth noting that samples from the YP group had relatively higher values at each storage period, matching the experimental results of Muhlisin et al. [30], which showed higher L* values for chilled pork under a high-O_2_-MAP condition. The decline in lightness and redness may result from structural variations such as protein oxidation or cross-linking, which is most likely to occur under highly oxidized conditions [31]. Conversely, b* values across all samples exhibited a gradual increase over time (Figure 2d). Higher yellowness typically indicates that meat oxidation has occurred and that bacteria have proliferated to a greater extent in the meat [27]. The results suggest that modified-atmosphere packaging has inhibitory effects on spoilage microorganisms, slowing increases in pH and delaying meat color deterioration, which are better than those of tray or vacuum packaging.

**Figure 3:**
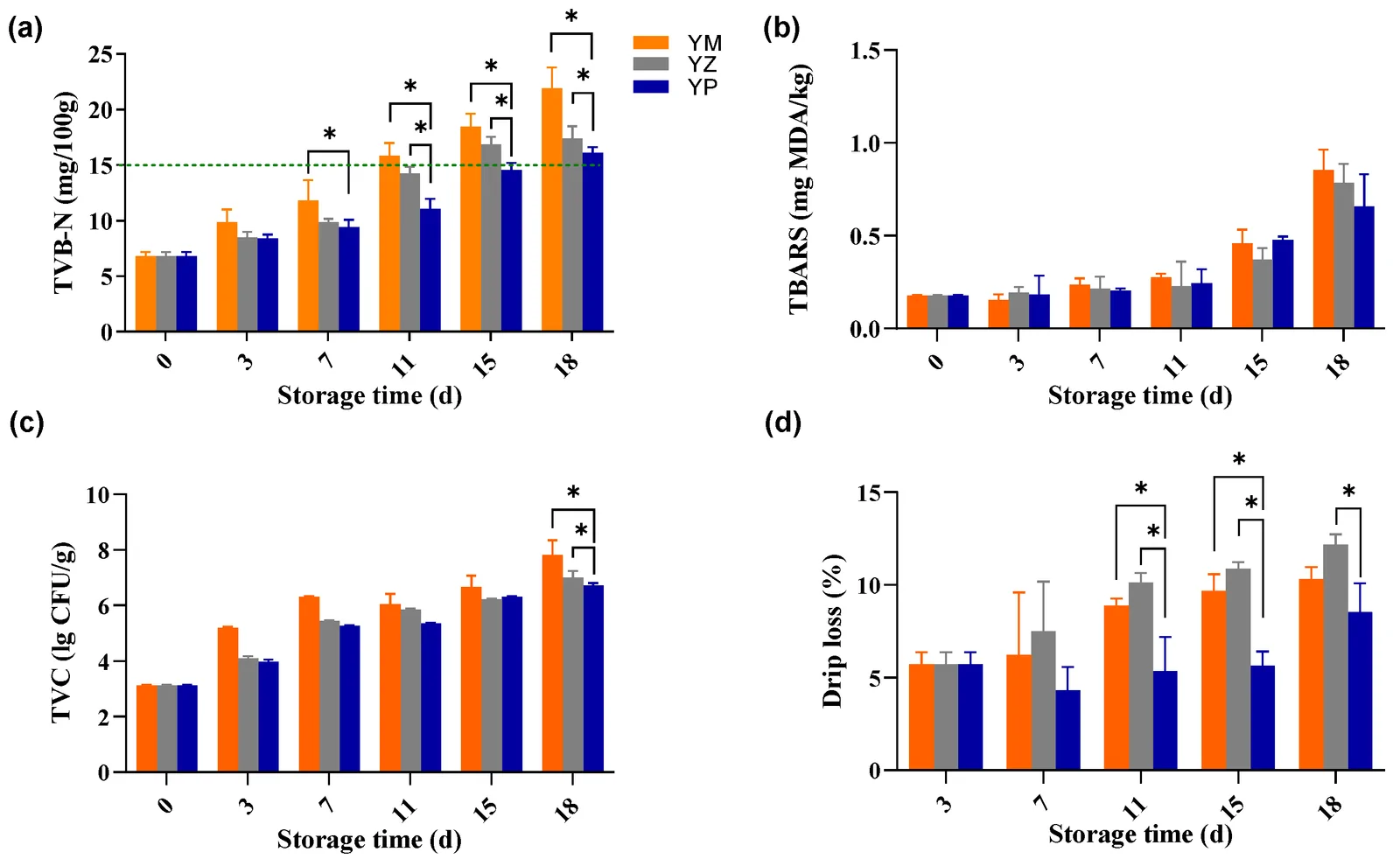
Changes in (**a**) TVB-N content, (**b**) TBARS value, (**c**) total viable counts (TVCs), and (**d**) drip loss of yak meat during 18 days of chilled storage. Data are presented as mean ± SD (*n* = 5; error bars indicate SD). Statistical analysis was performed using linear mixed-effects regression models followed by Tukey’s adjustment for multiple comparisons. Asterisks (*) above the bars at each storage time point indicate significant differences among packaging groups at *p* < 0.05.

When fresh meat decays during storage, the breakdown of proteins by microbial activity and proteolytic processes produces basic nitrogen-containing chemicals, such as amines and ammonia, collectively known as TVB-N [32]. The TVB-N content is often used as a crucial and sensitive measuring indicator of meat freshness. According to the Chinese National Food Safety Standard (GB 2707-2016) [17], the limit for fresh meat is 15 mg/100 g [33]. However, the acceptable limits reported in the literature range from 15 to 30 mg/100 g [34,35]. In this study, the TVB-N content steadily increased with storage time across all groups; however, the increase was substantially slower (*p* < 0.05) in the YP samples across all treatments, with the YM samples showing the fastest rate of increase. The concentration of TVB-N in the YM group rapidly increased to 15.85 ± 1.12 mg/100 g on day 11 of storage, whereas lower values of 9.88 ± 0.29 and 9.42 ± 0.65 mg/100 g were observed in the YZ and YP groups, respectively. The TVB-N concentration reached approximately 15 mg/100 g, the national standard limit, on day 11 in the YM group and on day 15 in the YP group (Figure 3a). Based on this finding, these time points (YM on day 11 and YP on day 15) were defined as near-spoiled stages and were used as exploratory comparison samples for subsequent multi-omics analysis. On day 18, the TVB-N concentration in all three groups exceeded the standard limit, indicating complete spoilage by the end of the study. In addition, TBARS content reflects the degree of lipid oxidation in meat, and excessive lipid oxidation is linked to off-flavor and off-odor. Generally, a TBARS concentration of 0.5 mg MDA/kg of TBARS is the threshold for detecting off-odor in raw meat [36]. Our results revealed an increasing trend in TBARS content over storage time across all samples, though at different rates. The TBARS values of the three groups did not exceed 0.5 mg MDA/kg, and no differences were observed among groups over time (Figure 3b). In addition to TVB-N, the microbial enumeration of TVC is another widely cited indicator of meat freshness. However, the acceptable limit for fresh meat varies across countries. In China, the former National Food Safety Standard (GB 16869-2005) [37] stipulated a limit of 6 log CFU/g; however, the new standard for fresh livestock (GB 2707-2016 [17]) no longer prescribes a TVC limit. In this study, the TVC value of all samples increased over time. Notably, YP samples kept a significantly lower (*p* < 0.05) TVC value on day 18 (Figure 3c). Drip loss is a commonly used indicator for assessing water-holding capacity. As shown in Figure 3d, the drip loss of samples from all groups increased with storage time. YP samples showed significantly lower (*p* < 0.05) values after 11 days of storage, indicating that yak meat stored under modified-atmosphere packaging had a better water-holding capacity. These findings revealed that modified-atmosphere packaging delayed quality deterioration.

### 3.2. Packaging Type Shift in Bacterial Community Structure

**Figure 4:**
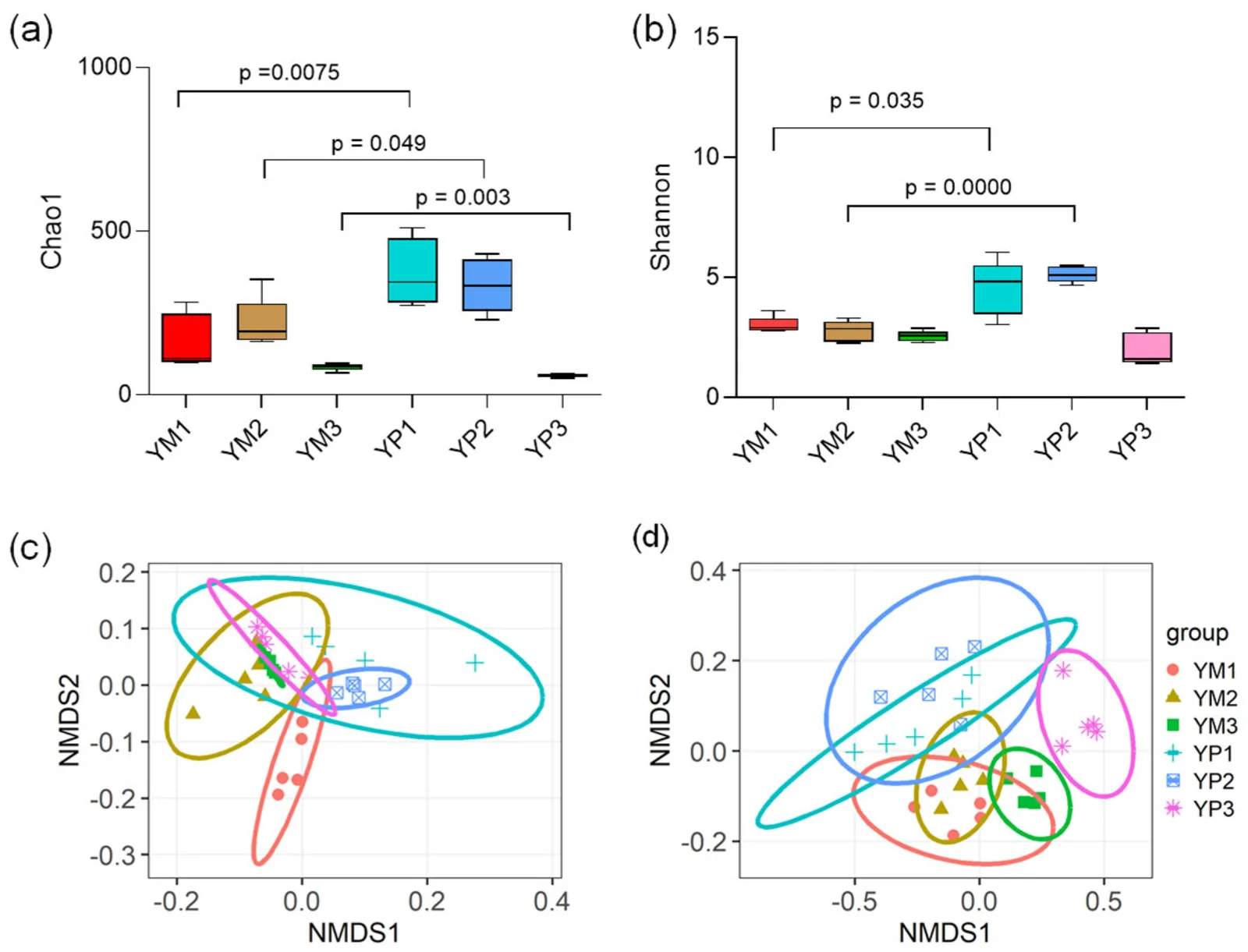
Differences in microbiota diversity between modified-atmosphere and tray-packaged yak meat samples during storage. Comparison of α-diversity in meat microbiota between YP and YM samples using (**a**) Shannon and (**b**) Chao1 indices. Non-metric multidimensional scaling (NMDS) plots of meat microbiota in YP and YM samples based on (**c**) Weighted UniFrac distances and (**d**) Unweighted UniFrac distances. α-diversity indices were evaluated using the Kruskal–Wallis test, and significant pairwise differences (*p* < 0.05) are indicated above the corresponding groups; ANOSIM based on Weighted UniFrac distance (R = 0.6179, *p* = 0.001) and Unweighted UniFrac distance (R = 0.7085, *p* = 0.001) confirmed significant separation among groups.

Packaging method and storage period were identified as significant factors influencing the shelf life of fresh meat, potentially leading to changes in bacterial structures. The Shannon and Chao1 of α-diversity indices of yak meat under different packaging methods are shown in Figure 4a,b. Both the Chao1 and Shannon values in the YP1, YP2, and YP3 groups were higher than those in the YM1, YM2, and YM3 groups. Chao1 is an indicator of species richness, and Shannon is a measure of how evenly abundant those species are in a sample. This result suggested that microbial growth and spoilage rates may vary with the gas mixture composition used in this study. Moreover, the values showed a marked decline in both groups throughout storage, which may be attributed to the decline in the initial microflora in the samples and to the fact that only a small number of specific spoilage organisms became dominant as storage time extended [38]. Bassey et al. [39] also revealed a decrease in the α-diversity indices of MAP-packaged pork under super-chilled storage, corroborating our result. Moreover, we assessed differences in bacterial community structure among groups using NMDS based on Weighted UniFrac (Figure 4c) and Unweighted UniFrac matrices (Figure 4d), which revealed markedly diverged separation between the YP and YM meat microbiota. ANOSIM confirmed significant separation among groups, with Weighted UniFrac distance (R = 0.6179, *p* = 0.001) and Unweighted UniFrac distance (R = 0.7085, *p* = 0.001) both indicating strong group discrimination.

**Figure 5:**
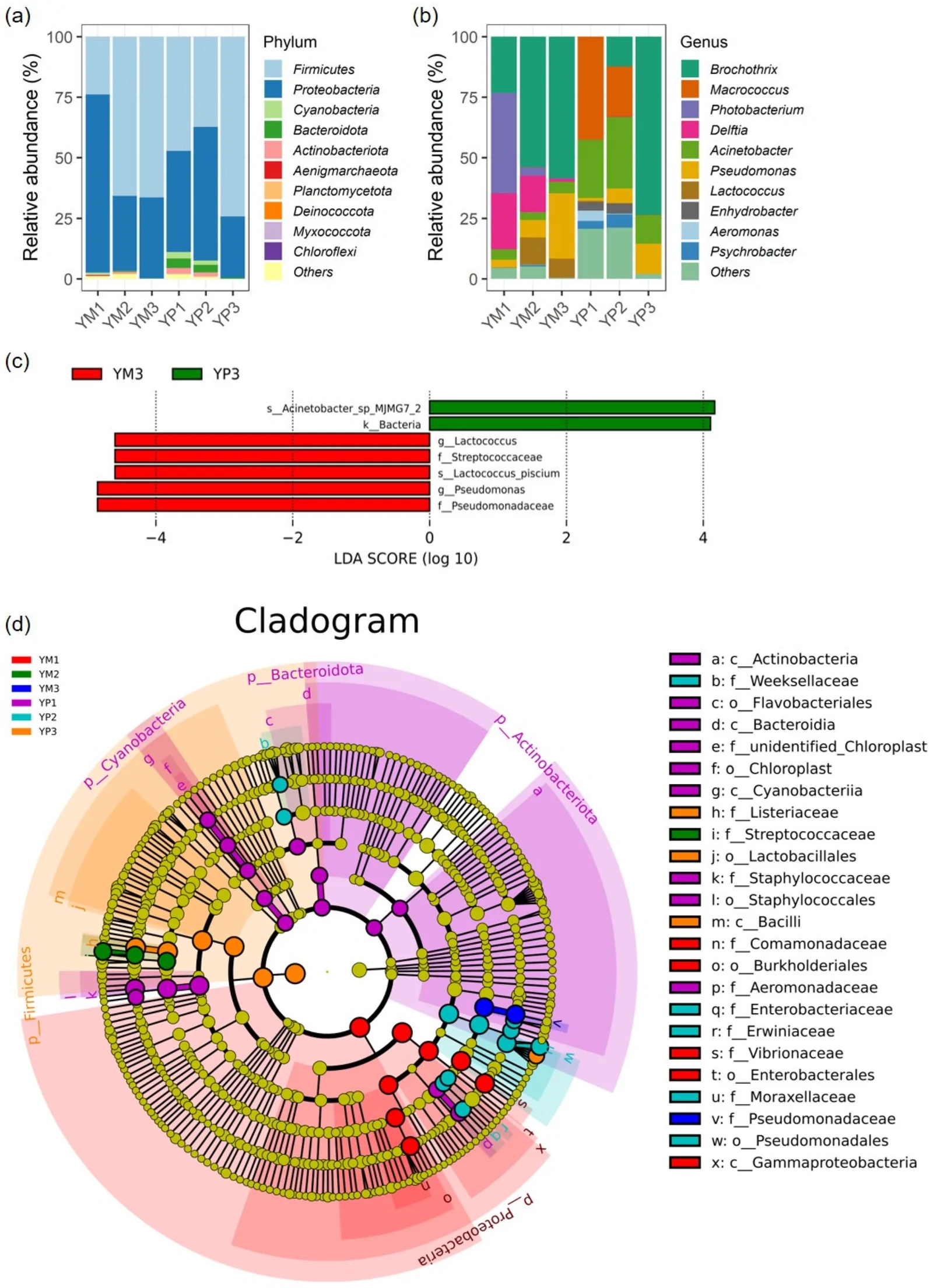
Differences in bacterial community composition and biomarkers between modified-atmosphere-packaged and tray-packaged yak meat samples during storage. Relative abundance of the top 10 bacterial (**a**) phylum and (**b**) genus levels. Differential OTUs identified by LEfSe between YP3 and YM3 groups (**c**). Cladogram illustrating statistically and biologically consistent taxonomic differences among groups (**d**). “Others” represents the remaining bacterial taxa not individually shown in the figure.

The relative abundance of bacterial communities in the groups was analyzed at the phylum and genus level. Firmicutes and Proteobacteria were the predominant phyla in all samples (Figure 5a). Similar phyla were also identified in fresh yak meat packed with oxygen barrier multilayer films [40] or stored under chilled or super-chilled conditions [41]. Furthermore, a comparison of bacterial composition at the genus level was made among samples during storage. As presented in Figure 5b, the main genera were *Brochothrix*, *Macrococcus*, *Photobacterium*, *Delftia*, *Acinetobacter*, *Pseudomonas*, and *Lactococcus*. Although *Enhydrobacter*, *Aeromonas*, and *Psychrobacter* were also detected, they had relatively low abundances. Under MAP conditions, the bacterial dominance was characterized by the succession from *Macrococcus* (42.47%) to *Brochothrix* (73.67%). *Pseudomonas* exhibited the same increasing trend in relative abundance over time in both YP and YM samples, and the relative abundance reached a high of 27.02% in the YM3 group. Notably, the relative abundance of *Lactococcus* was significantly decreased in YP samples compared to YM samples during storage.

Characteristics of the microbiota for the YP and YM samples were further explored at the OTU level using LEfSe analysis. At the near-spoiled stages, the YM meat was found to have significantly elevated biomarkers for the genera *Lactococcus* and *Pseudomonas* (Figure 5c), both of which are known major spoilage organisms in the microflora of fresh meat [42]. Species of the genus *Lactococcus* are commonly present in various environments, and the species *Lactococcus lactis* has been widely used for decades in the dairy industry. In addition, other species in the genus, such as psychrotrophic lactic acid bacteria, often dominated in chilled meat at the near-spoiled stages [43]. However, they have previously been described as playing a controversial role in meat spoilage. This mechanistic understanding of *Lactococcus’s* interaction with potent spoilers on yak meat under modified-atmosphere packaging conditions warrants further exploration. Furthermore, Figure 5d presents the cladogram, which illustrates the phylogenetic distribution of bacteria associated with yak meat under different packaging conditions and storage times. The significantly enriched biomarkers in YM1 were mainly enriched within the phylum Proteobacteria, while those in YP1 were mainly related to phyla Actinobacteriota and Bacteroidota. With the extension of storage time, the biomarkers shifted significantly: the YM2 group was characterized by *f_Streptococcaceae*, whereas the YP2 group was dominated by f_Enterobacteriaceae, f_Erwiniaceae, f_Moraxellaceae, and o_Pseudomonadales; f_Pseudomonadaceae characterized YM3, while those in YP3 mainly related to phylum Firmicutes.

### 3.3. Effects of Packaging on Yak Meat Metabolome Profile

**Figure 6:**
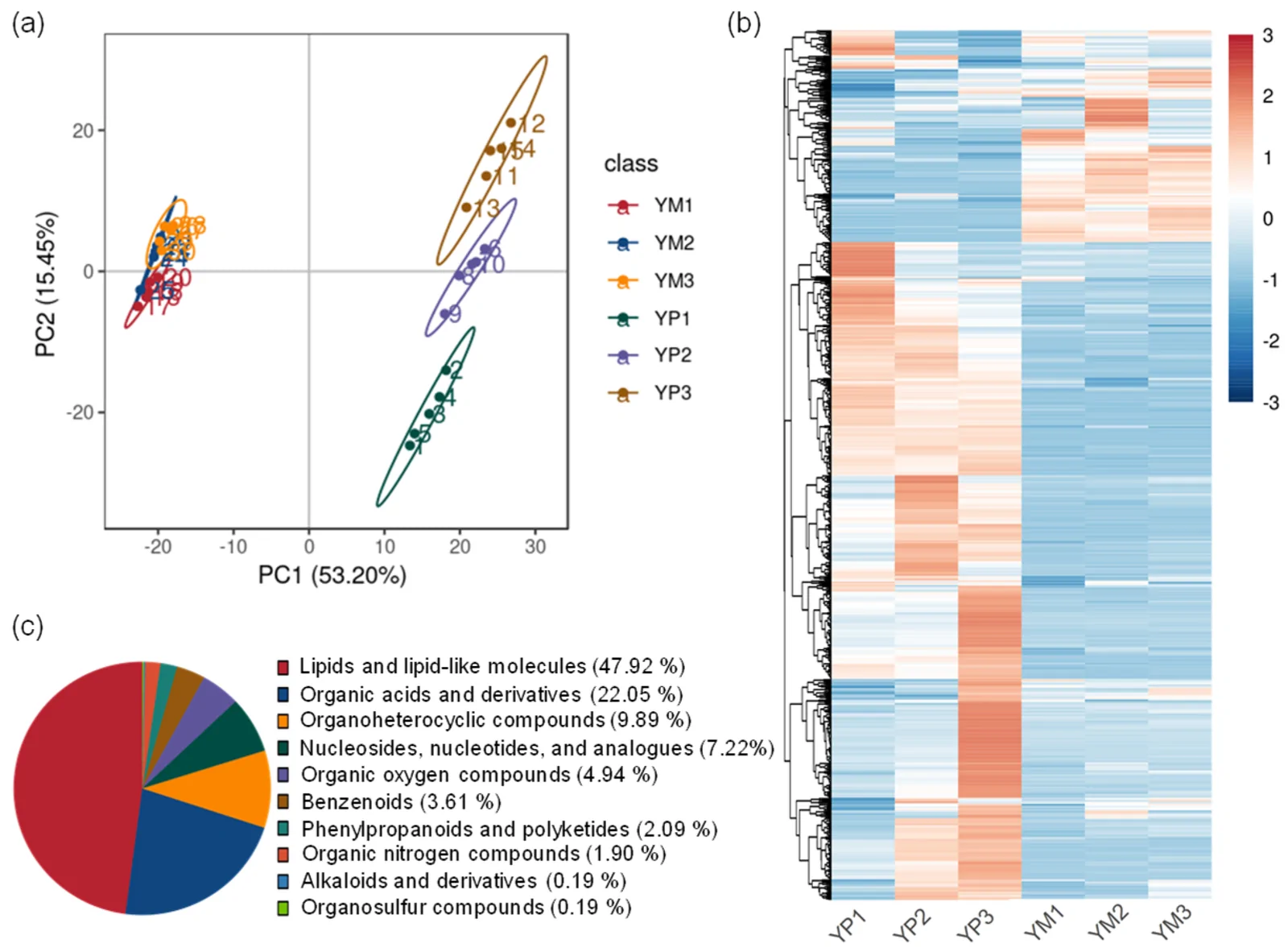
Overview of metabolites in modified-atmosphere-packaged and tray-packaged yak meat samples during storage based on LC-MS/MS analysis. PCA score plot displaying the separation of samples. (**a**) Heatmap visualization of the metabolites identified. (**b**) Classification and proportion of the metabolites identified (**c**).

Demonstrating metabolite changes in yak meat, a non-targeted metabolomics analysis was performed using a UHPLC system coupled to an Orbitrap Q Exactive HF-X mass spectrometer. A total of 818 metabolites were identified (Table S1), and these metabolites were analyzed using multivariate statistical analysis by principal component analysis (PCA). As shown in Figure 6a, the first principal component (PC1) and second principal component (PC2) were 53.20% and 15.45%, respectively. Meat samples with modified-atmosphere packaging treatments (YP1 to YP3) were clearly separated from those with tray packaging (YM1 to YM3), indicating substantial metabolic divergence between the packaging treatments. However, meat samples with tray packaging could not be clearly separated from one another, and they also clustered regularly with storage time. To better outline the dynamic changes in the metabolome across groups, the relative abundance of metabolites was normalized and clustered using heatmap analysis (Figure 6b). The results revealed two distinct clusters corresponding to the two packaging treatments, with the YP3 samples exhibiting the most pronounced increases in metabolite levels. These metabolites included 47.92% of lipids and lipid-like molecules; 22.05% organic acids and derivatives; 9.89% organoheterocyclic compounds; 7.22% nucleosides, nucleotides, and analogs; 4.94% organic oxygen compounds; 3.61% benzenoids; 2.09% phenylpropanoids and polyketides; 1.90% organic nitrogen compounds; 0.19% alkaloids and derivatives; and 0.19% organosulfur compounds (Figure 6c).

**Figure 7:**
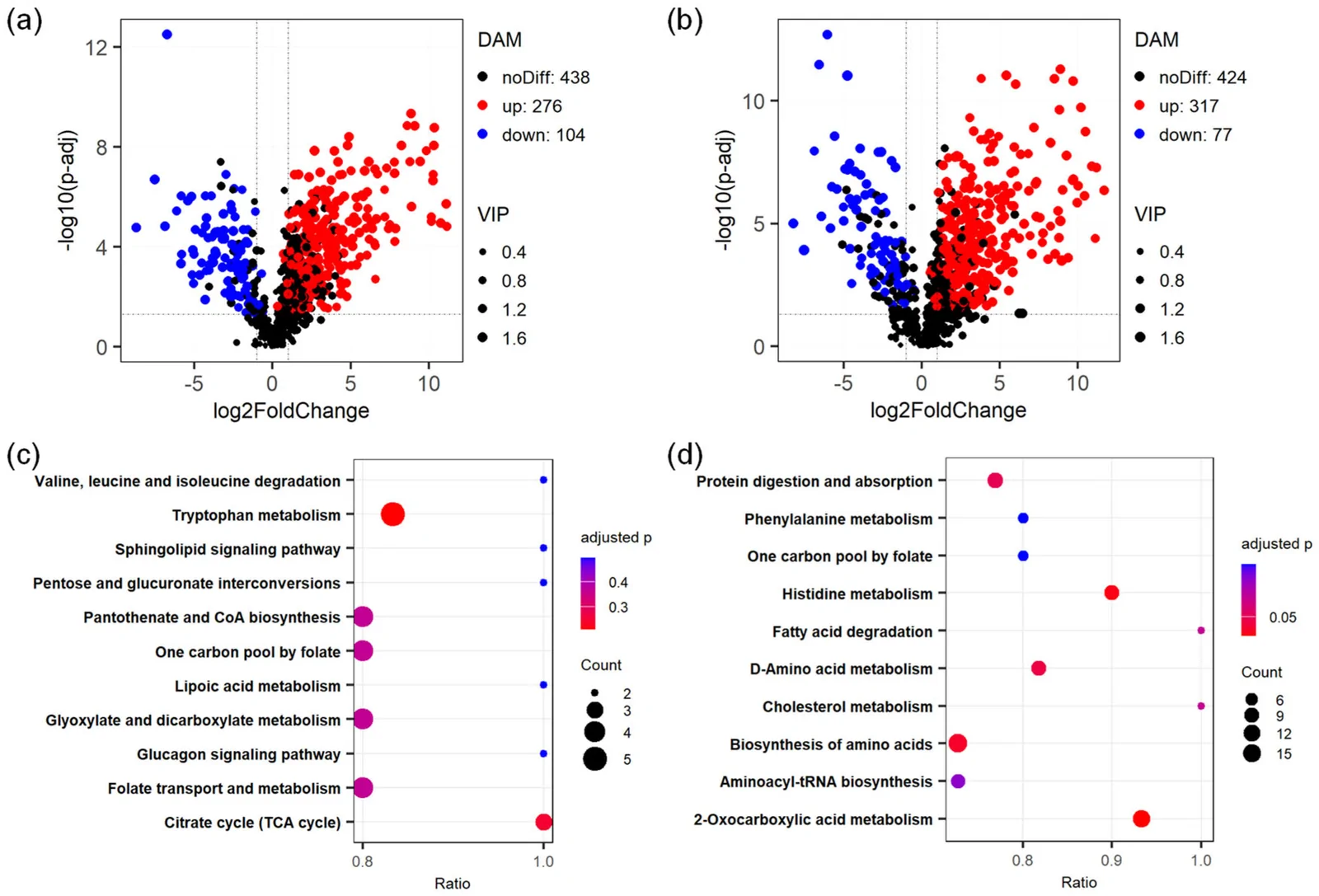
Differentially abundant metabolites (DAMs) and metabolic pathways across different storage periods. Volcano plots of the DAMs among the comparisons of YP1 vs. YM1 (**a**) and YP3 vs. YM3 (**b**); KEGG pathway analyses of the DAMs among the comparisons of YP1 vs. YM1 (**c**) and YP3 vs. YM3 (**d**).

Metabolites with VIP > 1, |fold change| ≥ 1.2, and adjusted *p* < 0.05 were defined as significantly differentially abundant metabolites (DAMs) for each comparison. As shown in Figure 7a, 380 DAMs were identified in the YP1 vs. YM1 comparison, including 276 that were upregulated and 104 that were downregulated. Upon extended storage, the total DAMs rose to 394 in the YP3 vs. YM3 comparison, with a marked increase in upregulated metabolites (317 up vs. 77 down), indicating a more pronounced metabolic response in near-spoiled stages (Figure 7b). To elucidate the potential metabolic pathways underlying MAP-induced spoilage of yak meat, KEGG enrichment analysis was conducted on these DAMs. Figure 7c presents the top enriched pathways in the YP1 vs. YM1 comparison, which were classified into three categories: core carbon metabolism (citrate cycle, glyoxylate and dicarboxylate metabolism, and pentose and glucuronate interconversions), amino acid and cofactor metabolism (tryptophan metabolism; one carbon pool by folate, pantothenate and CoA biosynthesis; and lipoic acid metabolism), and signal transduction (sphingolipid and glucagon signaling pathways). Nevertheless, all pathways exhibited adjusted *p* > 0.05, suggesting that although individual metabolic modules were affected, no single pathway was predominantly enriched under the current statistical criteria. In contrast, the DAMs in the comparison of YP3 vs. YM3 implicated more significant metabolic pathways, mainly associated with 2-oxocarboxylic acid metabolism, histidine metabolism, biosynthesis of amino acids, and D-amino acid metabolism (Figure 7d). Among these, 2-oxocarboxylic acid metabolism has been demonstrated to play important roles in suppressing bacterial proliferation under cold stress [44]. Histidine metabolism, biosynthesis of amino acids, and D-amino acid metabolism have been identified as a potential mechanism underlying spoilage in yak meat [15], refrigerated pork [45], and MAP pork [16]. These pathways are involved in the microbial degradation or utilization of proteins and lipids [45], suggesting that the two packaging methods exerted different biochemical effects on yak meat during storage and that these pathways are critical for maintaining meat stability. Moreover, the significant enrichment of these pathways at the later storage stage indicates that they play important roles in the packaging-related spoilage process.

**Figure 8:**
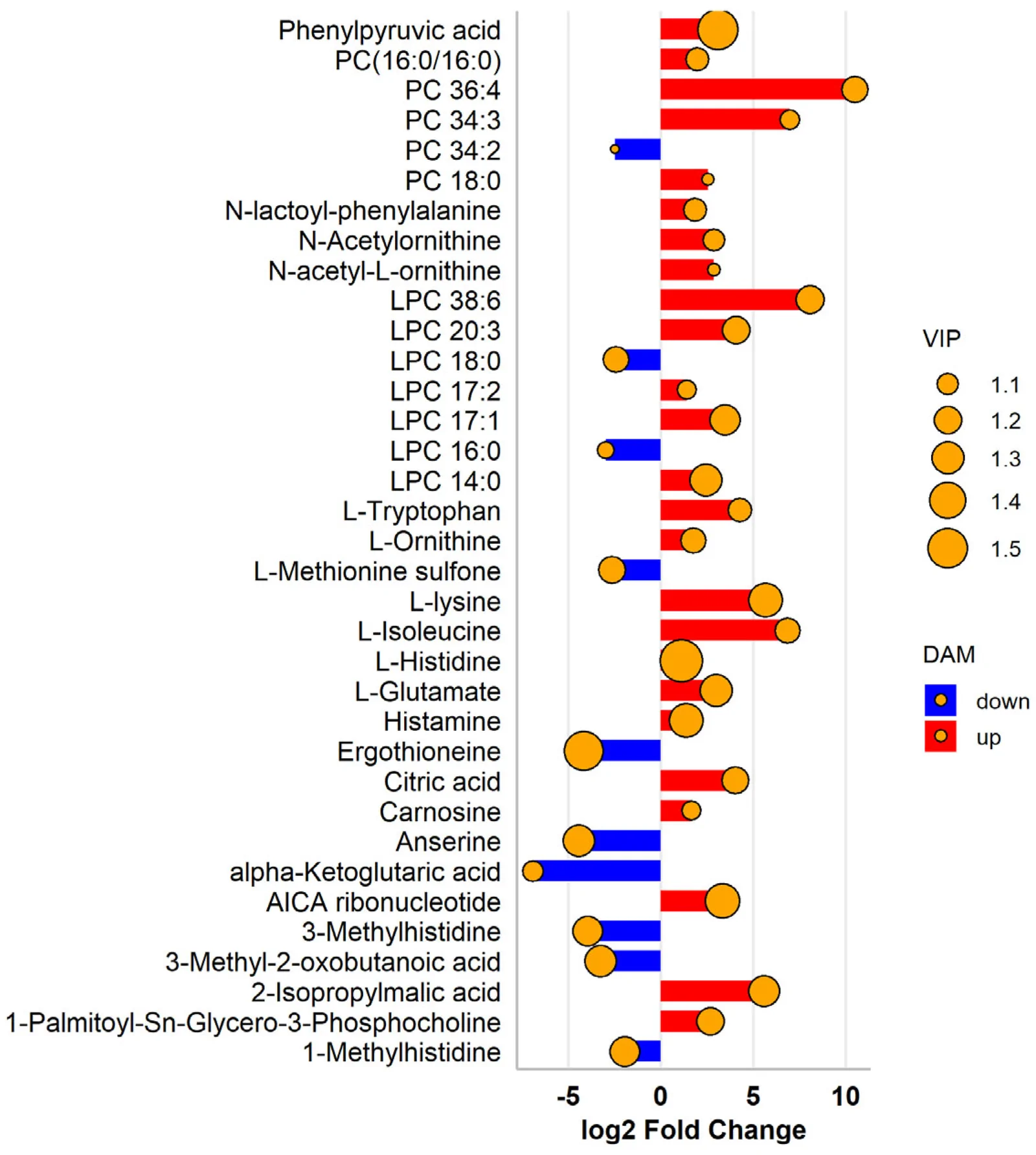
Stick plot analysis of the top 35 differentially abundant metabolites (DAMs) among the comparisons of YP3 vs. YM3. Dot size represents the Variable Importance in Projection (VIP) value, and upregulated and downregulated metabolites are in red and blue, respectively.

Figure 8 illustrates the top 35 most changed metabolites at the near-spoiled stages. The results demonstrated that metabolites related to lipid metabolic remodeling, namely PC (16:0/16:0), PC 36:4, PC 34:3, PC 18:0, LPC 38:6, LPC 20:3, LPC 17:2, LPC 17:1, LPC 14:0, and 1-palmitoyl-Sn-glycero-3-phosphocholine (lysoPC 16:0); those related to amino acid metabolic disorders, namely phenylpyruvic acid, N-lactoyl-phenylalanine, N-acetylornithine, N-acetyl-L-ornithine, L-tryptophan, L-ornithine, L-lysine, L-isoleucine, L-histidine, L-glutamate, histamine, and carnosine; and those related to energy metabolism, such as citric acid, AICA ribonucleotide (ZMP), and 2-isopropylmalic acid, were found to be significantly elevated in YP3. These metabolites are primarily produced through the degradation of proteins, lipids, and carbohydrates, and their accumulation results from complex biochemical reactions catalyzed by endogenous enzymes and microorganisms [3].

### 3.4. Correlation Analysis of Major Microorganisms and Metabolites

**Figure 9:**
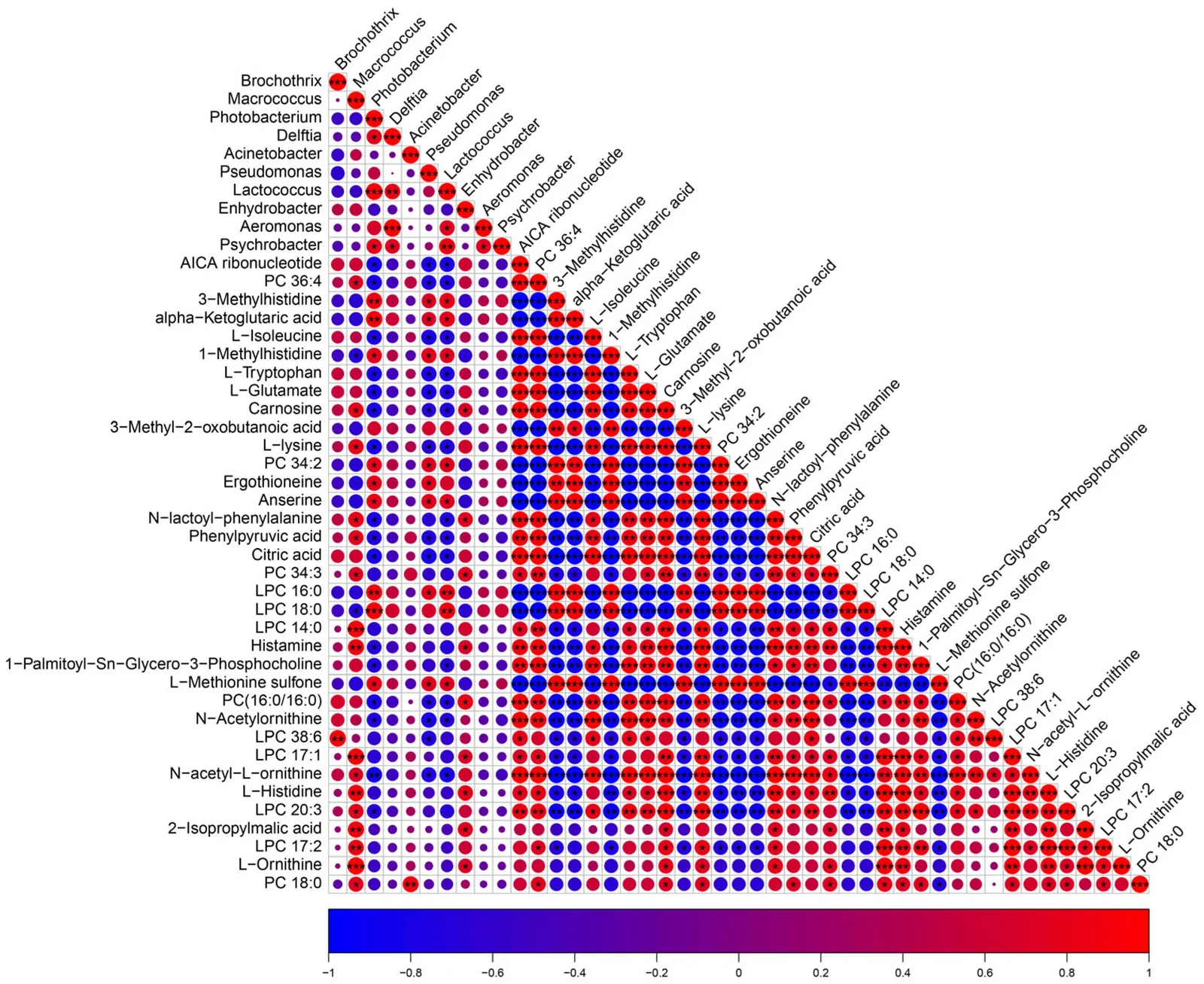
Correlation between major bacterial genera and metabolites in modified-atmosphere-packaged and tray-packaged yak meat samples at the near-spoiled stages. * Indicates significant difference (* adjusted *p* < 0.05; ** adjusted *p* < 0.01; *** adjusted *p* < 0.001).

The changes in bacterial genera significantly influence the accumulation and composition of metabolites in meat, thereby determining the deterioration of quality during storage [46]. Therefore, elucidating the correlation between microbial communities and metabolites under various packaging conditions during chilling storage is of paramount importance. As shown in Figure 9, Pearson correlation was used for statistical analysis of the relationship between the top 10 microbial genera and the top 35 DAMs in the comparisons of YP3 vs. YM3. Notably, multiple genera exhibited extensive correlations: *Macrococcus* was highly significantly correlated with 18 compounds, *Photobacterium* with 25 compounds, *Pseudomonas* with 21 compounds, *Lactococcus* with 22 compounds, and *Enhydrobacter* with 9 compounds. Of note, 1-methylhistidine, 3-methylhistidine, alpha-ketoglutaric acid, PC 34:2, ergothioneine, anserine, LPC 16:0, and L-methionine sulfone were positively correlated with *Pseudomonas*, and these metabolites were also positively correlated with several other genera, including *Photobacterium* and *Lactococcus*. Several previous works have demonstrated that *Pseudomonas*, *Photobacterium*, and *Lactococcus* were the key spoilage microorganisms detected in chilled meat under various packaging conditions [15,47]. These results suggested that the accumulated metabolites during storage may serve as metabolic signatures of spoilage in yak meat [45], particularly in meat samples stored under tray packaging, and were closely linked to the significantly enriched biomarkers of these genera in YM3 samples (Figure 5). Collectively, the relative abundance of bacterial genera *Pseudomonas*, *Photobacterium* and *Lactococcus* was strongly associated with specific metabolites, and their lower abundances in YP3 samples indicated that MAP inhibited the metabolic properties of microorganisms during storage, resulting in an increase in shelf life.

Several limitations of this study should be acknowledged. First, sensory evaluation was not performed, which limits our ability to directly correlate the observed physicochemical and microbial changes with the actual shelf-life of yak meat under tray packaging and 70% O_2_/30% CO_2_ modified-atmosphere packaging. Second, the initial and residual gas composition (O_2_ and CO_2_) in the packages should be monitored at each sampling time point during storage, and sufficient interpretation should be provided regarding how the specific gas composition regulates microbial succession and metabolic changes. Third, although tray packaging remains the most common commercial option and MAP shows the greatest promise for improving yak meat quality, the multi-omics profiles of vacuum-packaged samples were not fully characterized in this study. Fourth, the findings are based on a specific set of packaging conditions and storage temperatures, and may not be generalizable to other meat types or processing parameters. Future studies incorporating sensory analysis and broader experimental conditions are warranted to validate and extend our conclusions.

## 4. Conclusions

In this study, we characterized yak meat quality under three packaging conditions (YM, YZ, and YP) using physicochemical tests and total viable count (TVC) assays. The progression of spoilage indicators was slower in YP compared with YM and YZ. Integrative 16S rRNA sequencing and metabolomic analysis revealed distinct microbial and metabolic profiles between YP and YM, with *Lactococcus* and *Pseudomonas* dominating YM, and key differential pathways including 2-oxocarboxylic acid metabolism, histidine metabolism, biosynthesis of amino acids, and D-amino acid metabolism. Correlation analysis further linked specific genera (*Macrococcus*, *Photobacterium*, *Pseudomonas*, *Lactococcus*, and *Enhydrobacter*) to signature metabolites. These findings provide a scientific basis for MAP application in shelf-life extension of yak meat.

## Data Availability

The data presented in this study are available in the Sequence Read Archive (SRA) under accession number PRJNA1497126, and in the MetaboLights database under accession number MTBLS15217. These data were derived from the following resources available in the public domain: SRA (https://www.ncbi.nlm.nih.gov/sra, accessed on 18 July 2026) and MetaboLights (http://www.ebi.ac.uk/metabolights, accessed on 27 July 2026).

## Supplementary Materials

The following supporting information can be downloaded at: https://www.mdpi.com/article/10.3390/foods15162903/s1, Table S1. Summary of the identified metabolites across all samples.
