# Supplementary material for: Spoilage Indicators, Bacterial Dynamics, and Metabolomic Profiles of Chilled Yak Meat Under Tray, Vacuum, and Modified-Atmosphere Packaging: A Comparative Study with Integrated Multi-Omics Focused on Tray Versus MAP Conditions
Source: Foods. 2026 Aug 19;15(16):2903. doi: 10.3390/foods15162903 (PMC13511978; doi:10.3390/foods15162903)
Supplement: Supplementary file 1 [file foods-15-02903-s001.zip › foods-4435666-supplementary.pdf]

Table S1. Summary of the identified metabolites across all samples.							
**Compound_ID**	**Name**	**Formula**	**Molecular Weight**	**RT [min]**	**m/z**	**mzCloud_Results**	**mzVault_Results**
Com_1_all	DL-Carnitine	C7 H15 N O3	161.1052	1.372	162.11247	Full match	Full match
Com_2_all	Oleamide	C18 H35 N O	281.27172	9.956	282.27901	Full match	No results
Com_3_all	Hexadecanamide	C16 H33 N O	255.25614	9.735	256.26343	Full match	No results
Com_4_all	1-Stearoylglycerol	C21 H42 O4	358.30806	10.779	381.29725	Full match	Full match
Com_5_all	L-Phenylalanine	C9 H11 N O2	165.07909	4.956	166.08636	Full match	Full match
Com_6_all	Oleoyl ethylamide	C20 H39 N O	309.30302	10.795	332.29219	Full match	No results
Com_7_all	Tyramine	C8 H11 N O	137.08415	4.946	138.0915	No results	No results
Com_8_all	Stearamide	C18 H37 N O	283.28739	10.601	306.27654	Full match	No results
Com_9_all	Monoolein	C21 H40 O4	356.29265	10.16	379.28184	Full match	No results
Com_10_all	Hexanoylcarnitine	C13 H25 N O4	259.17851	5.523	260.18579	Full match	Full match
Com_11_all	LPC 18:2-SN1	C26 H50 N O7 P	519.33285	9.128	520.34022	No results	Full match
Com_12_all	CAR 18:1	C25 H48 N O4	425.3507	8.662	426.35796	No results	Invalid mass
Com_13_all	Palmitoylcarnitine	C23 H45 N O4	399.33498	8.471	400.34226	Full match	Not the top hit
Com_14_all	PC 17:1_17:1	C42 H80 N O8 P	757.56273	11.025	758.57004	No results	Full match
Com_15_all	PC O-18:1	C26 H52 N O7 P	521.34828	9.477	522.35552	No results	Full match
Com_16_all	5-OxoETE	C20 H30 O3	318.2172	7.994	319.22441	Invalid mass	No results
Com_17_all	tert-Butyl N-[1-(aminocarbonyl)-3-methylbutyl]carbamate	C11 H22 N2 O3	230.16333	5.311	231.1706	Full match	No results
Com_18_all	Propionylcarnitine	C10 H19 N O4	217.13159	3.349	218.13887	Full match	Full match
Com_19_all	Epigallocatechin	C15 H14 O7	306.07614	2.405	307.08342	No results	Invalid mass
Com_20_all	9-Oxo-10(E),12(E)-octadecadienoic acid	C18 H30 O3	294.21964	7.996	295.22649	Full match	No results
Com_21_all	2-Hydroxycinnamic acid	C9 H8 O3	164.04754	2.375	165.05481	Full match	Full match
Com_22_all	N-[2-chloro-6-(trifluoromethoxy)phenyl]-2,2-dimethylpropanamide	C12 H13 Cl F3 N O2	295.05884	1.3	296.06612	Full match	No results
Com_23_all	CAR 16:1	C23 H44 N O4	397.31941	7.939	398.32669	No results	Invalid mass
Com_24_all	PC 34:2	C42 H80 N O8 P	1559.08808	11.024	780.55131	No results	Invalid mass
Com_25_all	CAR 14:1	C21 H40 N O4	369.28805	7.29	370.29533	No results	Invalid mass
Com_26_all	2-Amino-1,3,4-octadecanetriol	C18 H39 N O3	317.29309	6.773	318.30037	Full match	No results
Com_27_all	PLK	C17 H32 N4 O4	356.24255	1.448	179.12859	Full match	No results
Com_28_all	N-Tetradecanamide	C14 H29 N O	227.22504	8.81	228.23233	No results	No results
Com_29_all	LPC 34:3	C42 H80 N O7 P	763.55014	10.478	764.55742	No results	Invalid mass
Com_30_all	Docosapentaenoic acid	C22 H34 O2	330.25594	10.237	331.26329	Full match	No results
Com_31_all	CAR 20:4	C27 H46 N O4	447.33513	8.155	448.3424	No results	Invalid mass
Com_32_all	1,4-dihydroxyheptadec-16-en-2-yl acetate	C19 H36 O4	328.26139	9.306	351.25063	Full match	No results
Com_33_all	L-5-Hydroxytryptophan	C11 H12 N2 O3	220.08508	4.997	221.09236	Full match	No results
Com_34_all	Ala-Ile	C9 H18 N2 O3	202.13209	5.162	203.13937	No results	Full match
Com_35_all	LSD-d3	C20 H22 [2]H3 N3 O	326.21986	10.685	327.22714	Full match	No results
Com_36_all	Prolylleucine	C11 H20 N2 O3	228.14762	3.153	229.1549	Full match	No results
Com_37_all	LPC 20:4-SN1	C28 H50 N O7 P	543.33306	9.06	566.32226	No results	Full match
Com_38_all	N6,N6,N6-Trimethyl-L-lysine	C9 H20 N2 O2	188.15272	1.25	189.15999	Full match	Full match
Com_39_all	PC 18:1_18:2	C44 H82 N O8 P	783.57848	11.695	784.58572	No results	Full match
Com_40_all	PC O-36:5	C44 H80 N O7 P	765.56651	10.786	766.5737	No results	Full match
Com_41_all	CAR 7:0	C14 H28 N O4	273.19417	5.678	274.20144	No results	Invalid mass
Com_42_all	LPC 34:2	C42 H82 N O7 P	765.56536	10.947	766.57264	No results	Invalid mass
Com_43_all	PC 38:4	C46 H84 N O8 P	831.57575	10.705	832.58365	No results	Invalid mass
Com_44_all	PC O-20:3	C28 H52 N O7 P	545.34875	9.249	546.35604	No results	Full match
Com_45_all	Decanoylcarnitine	C17 H33 N O4	315.24109	6.399	316.24837	Full match	Invalid mass
Com_46_all	CAR 20:5	C27 H44 N O4	445.31949	7.746	446.32676	No results	Invalid mass
Com_47_all	PC O-20:5	C28 H48 N O7 P	541.31753	8.383	542.3249	No results	Full match
Com_48_all	PC 36:5	C44 H78 N O8 P	779.5464	11.258	780.55366	No results	Full match
Com_49_all	(2E,4E)-N-(2-methylpropyl)dodeca-2,4-dienamide	C16 H29 N O	251.22507	8.629	252.23239	Full match	No results
Com_50_all	PC O-35:2	C43 H84 N O7 P	757.59928	10.718	758.60648	No results	Full match
Com_51_all	C-8 Ceramide-1-phosphate	C26 H52 N O6 P	505.35367	10.064	506.36099	Full match	Full match

Compound_ID	Name	Formula	Molecular Weight	RT [min]	m/z	mzCloud_Results	mzVault_Results
Com_52_all	Norbutorphanol	C16 H21 N O2	259.15355	5.151	260.16082	Invalid mass	No results
Com_53_all	CAR 22:0	C29 H58 N O4	483.42915	10.483	484.43643	No results	Invalid mass
Com_54_all	4,4'-dimethoxy[1,1'-biphenyl]-2-carbonitrile	C15 H13 N O2	239.09498	4.806	240.10225	Full match	No results
Com_55_all	N1-[4-hydroxy-6-(methoxymethyl)pyrimidin-2-yl]acetamide	C8 H11 N3 O3	197.08029	1.527	198.08757	Full match	Full match
Com_56_all	PC O-16:1	C24 H48 N O7 P	455.36146	8.593	456.36889	No results	Invalid mass
Com_57_all	Ecgonine	C9 H15 N O3	185.10542	1.816	186.1127	Full match	Full match
Com_58_all	2-[(3S)-1-Benzyl-3-pyrrolidinyl]-1,3-benzothiazole	C18 H18 N2 S	294.12171	4.991	295.12899	Invalid mass	No results
Com_59_all	CAR 17:0	C24 H48 N O4	413.35089	8.735	414.35816	No results	Invalid mass
Com_60_all	Oleoyl ethanolamide	C20 H39 N O2	325.29821	9.839	348.28743	Full match	No results
Com_61_all	Gly-Val	C7 H14 N2 O3	174.10064	2.227	175.10792	No results	Full match
Com_62_all	PC O-22:5	C30 H52 N O7 P	569.34853	9.101	570.35574	No results	Full match
Com_63_all	5-Hydroxyindole-3-acetic acid	C10 H9 N O3	191.05856	4.821	209.0924	Full match	Full match
Com_64_all	LPC 17:0-SN1	C25 H52 N O7 P	509.34855	9.912	510.35584	No results	Full match
Com_65_all	CAR 18:2	C25 H46 N O4	423.33513	8.438	424.34241	No results	Invalid mass
Com_66_all	Linoleoyl ethanolamide	C20 H37 N O2	323.2826	8.414	346.27182	Full match	No results
Com_67_all	TKK	C16 H33 N5 O5	375.25232	6.386	398.2416	Invalid mass	No results
Com_68_all	Oleic acid	C18 H34 O2	282.25596	10.718	283.26324	Full match	No results
Com_69_all	SM 8:1;2O/25:0	C38 H77 N2 O6 P	688.55275	10.698	689.55992	No results	Full match
Com_70_all	Bicyclo prostaglandin E2	C20 H30 O4	334.21298	6.772	335.2201	Full match	No results
Com_71_all	PC O-17:1	C25 H50 N O7 P	507.33306	9.038	508.34034	No results	Full match
Com_72_all	2-Linoleoyl glycerol	C21 H38 O4	336.26411	10.448	337.27139	Invalid mass	No results
Com_73_all	Levalbuterol	C13 H21 N O3	221.13939	5.871	222.14667	Invalid mass	No results
Com_74_all	2,4-dihydroxyheptadec-16-en-1-yl acetate	C19 H36 O4	350.24341	9.203	351.25069	Invalid mass	No results
Com_75_all	CAR 20:0	C27 H54 N O4	455.39773	10.164	494.3609	No results	Invalid mass
Com_76_all	LPC 36:4	C44 H82 N O7 P	789.56545	10.483	790.57273	No results	Invalid mass
Com_77_all	N-(9-oxodecyl)acetamide	C12 H23 N O2	235.15508	6.091	236.16236	Invalid mass	No results
Com_78_all	LPC 16:1-SN1	C24 H48 N O7 P	493.31745	9.221	516.3067	No results	Full match
Com_79_all	D-Sphingosine	C18 H37 N O2	299.2826	8.046	300.28989	Full match	Full match
Com_80_all	1-Methylhistidine	C7 H11 N3 O2	169.0853	1.245	170.09256	Full match	Full match
Com_81_all	methyl 3,4,5-trihydroxycyclohex-1-ene-1-carboxylate	C8 H12 O5	170.05568	2.764	171.06296	Invalid mass	No results
Com_82_all	CAR 15:1	C22 H42 N O4	383.30364	7.638	384.31091	No results	Invalid mass
Com_83_all	SM 8:1;2O/28:1	C41 H81 N2 O6 P	728.58314	9.949	729.5904	No results	Full match
Com_84_all	Cytidine	C9 H13 N3 O5	243.08573	1.748	244.09301	Full match	Full match
Com_85_all	PC O-33:2	C41 H80 N O7 P	729.56872	11.383	730.57549	No results	Full match
Com_86_all	5'-S-Methyl-5'-thioadenosine	C11 H15 N5 O3 S	297.08979	5.174	298.09706	Full match	Full match
Com_87_all	Guanine	C5 H5 N5 O	151.04955	3.559	152.05683	Full match	Full match
Com_88_all	Fenpropimorph	C20 H33 N O	303.25391	10.714	304.26118	Invalid mass	No results
Com_89_all	(11E,15Z)-9,10,13-trihydroxyoctadeca-11,15-dienoic acid	C18 H32 O5	328.22528	6.332	329.23219	Full match	No results
Com_90_all	LPC 17:1-SN1	C25 H50 N O7 P	507.33321	9.258	508.34052	No results	Full match
Com_91_all	2-Arachidonyl Glycerol ether	C23 H40 O3	364.29549	10.749	365.30276	Invalid mass	No results
Com_92_all	Styrene	C8 H8	104.06255	6.405	105.06982	No results	No results
Com_93_all	CAR 22:5	C29 H48 N O4	473.351	8.359	474.35827	No results	Invalid mass
Com_94_all	PC O-36:4	C44 H82 N O7 P	767.5833	11.384	768.59057	No results	Full match
Com_95_all	CAR 20:3	C27 H48 N O4	449.35089	8.476	450.35814	No results	Invalid mass
Com_96_all	L-Threonine	C4 H9 N O3	119.05836	1.567	120.06564	No results	No results
Com_97_all	4-(2,3-dihydro-1,4-benzodioxin-6-yl)butanoic acid	C12 H14 O4	244.0715	6.224	245.07878	Invalid mass	No results
Com_98_all	3-amino-2-phenyl-2H-pyrazolo[4,3-c]pyridine-4,6-diol	C12 H10 N4 O2	242.08065	5.823	243.08796	Full match	No results
Com_99_all	L-Isoleucine	C6 H13 N O2	131.09476	5.173	132.10204	Full match	No results
Com_100_all	L-Threonic acid-1,4-lactone	C4 H6 O4	118.02801	2.023	119.03529	Invalid mass	No results
Com_101_all	PC O-34:4	C42 H78 N O7 P	739.55248	10.128	740.55971	No results	Full match
Com_102_all	Nicotinamide adenine dinucleotide (NAD+)	C21 H27 N7 O14 P2	663.10949	1.854	332.56196	Full match	Full match
Com_103_all	3-(propan-2-yl)-octahydropyrrolo[1,2-a]pyrazine-1,4-dione	C10 H16 N2 O2	196.12145	5.245	197.12873	Full match	No results
Com_104_all	Palmitoleic Acid	C16 H30 O2	294.21981	7.645	277.21649	Invalid mass	No results

Compound_ID	Name	Formula	Molecular Weight	RT [min]	m/z	mzCloud_Results	mzVault_Results
Com_105_all	GPK	C13 H24 N4 O4	644.32888	4.893	323.17171	Invalid mass	No results
Com_106_all	25-hydroxycholecalciferol	C27 H44 O2	400.33431	11.216	401.3416	No results	Full match
Com_107_all	PC O-38:9	C46 H76 N O7 P	785.52283	10.754	786.5298	No results	Invalid mass
Com_108_all	PC O-22:4	C30 H54 N O7 P	571.36446	9.575	572.37174	No results	Full match
Com_109_all	PC 38:5	C46 H82 N O8 P	829.56002	11.366	830.5673	No results	Invalid mass
Com_110_all	CAR 15:0	C22 H44 N O4	385.31944	8.089	386.32672	No results	Invalid mass
Com_111_all	PC 18:2_19:2	C45 H82 N O8 P	795.57632	11.638	796.58419	No results	Full match
Com_112_all	Coumarin	C9 H6 O2	146.03697	2.578	147.04424	Full match	Full match
Com_113_all	1-morpholino-3-(4-nitrophenoxy)propan-2-ol	C13 H18 N2 O5	282.12187	4.763	283.12914	Full match	No results
Com_114_all	N~5~-(pyridin-2-ylmethyl)-1H-1,2,4-triazole-3,5-diamine	C8 H10 N6	190.09722	5.761	191.1045	Full match	No results
Com_115_all	Corey Lactone Diol	C8 H12 O4	172.07378	5.025	173.08106	No results	No results
Com_116_all	Guanosine monophosphate	C10 H14 N5 O8 P	363.05807	1.843	364.06534	No results	No results
Com_117_all	N~5~-(1,3,5-trimethyl-1H-pyrazol-4-yl)-1H-1,2,4-triazole-3,5-diamine	C8 H13 N7	207.12387	5.71	208.13111	Full match	No results
Com_118_all	N-Acetylmethionine	C7 H13 N O3 S	191.06192	5.284	214.0512	No results	Full match
Com_119_all	DL-Arginine	C6 H14 N4 O2	174.11188	1.522	175.11916	Full match	Full match
Com_120_all	Nicotinamide	C6 H6 N2 O	122.04809	1.752	123.05537	Full match	Full match
Com_121_all	CAR 10:1	C17 H32 N O4	313.22546	6.17	314.23274	No results	Invalid mass
Com_122_all	PE 18:1_18:2	C41 H76 N O8 P	741.53173	11.383	742.53907	No results	Full match
Com_123_all	PC O-38:7	C46 H80 N O7 P	789.55215	11.062	790.55943	No results	Invalid mass
Com_124_all	Eicosapentaenoic acid ethyl ester	C22 H34 O2	325.2982	9.021	326.30558	Invalid mass	No results
Com_125_all	CAR 18:3	C25 H44 N O4	421.31954	7.962	422.32683	No results	Invalid mass
Com_126_all	LPC 15:0-SN1	C23 H48 N O7 P	232.14547	9.032	482.3248	No results	Invalid mass
Com_127_all	Cholecalciferol	C27 H44 O	384.33934	11.091	367.33606	Full match	No results
Com_128_all	SM 36:1;2O	C41 H83 N2 O6 P	752.58295	10.369	753.59023	No results	Invalid mass
Com_129_all	13,14-Dihydro prostaglandin E1	C20 H36 O5	316.26163	7.511	317.26902	Invalid mass	No results
Com_130_all	2-[(7-methyl-2,3-dihydro-1H-inden-4-yl)oxy]pyridin-3-amine	C15 H16 N2 O	223.09592	5.182	241.12972	Invalid mass	Invalid mass
Com_131_all	Dl-3-Hydroxynorvaline	C5 H11 N O3	133.07396	5.17	116.07068	No results	No results
Com_132_all	16α-Hydroxydehydroepiandrosterone	C19 H28 O3	321.22823	6.97	322.2355	Invalid mass	No results
Com_133_all	PC O-36:3	C44 H84 N O7 P	769.59815	11.714	770.60514	No results	Full match
Com_134_all	α-Estradiol	C18 H24 O2	272.17297	9.593	273.18024	Invalid mass	No results
Com_135_all	CAR 18:0	C25 H50 N O4	427.36634	9.445	428.3739	No results	Invalid mass
Com_136_all	3-(5-phenyl-1,3-oxazol-2-yl)-4-(trifluoromethyl)pyridine	C15 H9 F3 N2 O	290.06279	3.288	291.07007	Invalid mass	No results
Com_137_all	C-6 NBD ceramide	C30 H49 N5 O6	597.34359	9.302	620.33286	Invalid mass	No results
Com_138_all	L-Anserine (beta-alanyl-N-methylhistidine) (nitrate salt)	C10 H16 N4 O3	240.12235	2.083	241.12961	No results	Full match
Com_139_all	Glu-Glu	C10 H16 N2 O7	276.09576	1.471	277.10306	No results	No results
Com_140_all	EPK	C16 H28 N4 O6	186.1006	1.479	187.10795	Invalid mass	No results
Com_141_all	PC 17:0_18:3	C43 H80 N O8 P	769.56189	10.118	770.5692	No results	Full match
Com_142_all	Methanandamide	C23 H39 N O2	383.28568	9.948	384.29296	Invalid mass	No results
Com_143_all	Thromboxane B1	C20 H36 O6	394.23361	6.693	395.24107	Invalid mass	No results
Com_144_all	CAR 19:0	C26 H52 N O4	441.38206	9.533	442.38934	No results	Invalid mass
Com_145_all	16(R)-HETE	C20 H32 O3	342.21708	8.782	343.22435	Invalid mass	No results
Com_146_all	VMK	C16 H32 N4 O4 S	358.20102	5.175	180.10783	Invalid mass	No results
Com_147_all	2-Benzyl-5-[(3S)-1-isopropyl-3-pyrrolidinyl]-1,3,4-oxadiazole	C16 H21 N3 O	271.16447	1.667	272.17175	Invalid mass	No results
Com_148_all	PC O-37:5	C45 H82 N O7 P	1559.16315	10.487	780.58885	No results	Invalid mass
Com_149_all	Pyroglutamic acid	C5 H7 N O3	129.04276	5.105	130.05004	No results	Full match
Com_150_all	3-[1-(dimethylamino)-1H-pyrrol-2-yl]-2-(2-furylcarbonyl)acrylonitrile	C14 H13 N3 O2	255.09855	1.279	256.10587	Invalid mass	No results
Com_151_all	10-Nitrolinoleate	C18 H31 N O4	347.20748	6.228	348.21475	Invalid mass	No results
Com_152_all	Glycylproline	C7 H12 N2 O3	172.08499	1.843	173.09227	Full match	No results
Com_153_all	4,7,8-trimethoxyfuro[2,3-b]quinoline	C14 H13 N O4	259.08235	5.689	260.08962	Invalid mass	No results
Com_154_all	PC 8:0_13:1	C29 H56 N O8 P	577.3747	9.765	578.3818	No results	Full match
Com_155_all	CAR 17:1	C24 H46 N O4	411.33517	8.295	412.34245	No results	Invalid mass
Com_156_all	2-(14,15-Epoxyeicosatrienoyl) glycerol	C23 H38 O5	376.26143	8.91	377.26874	Invalid mass	No results
Com_157_all	Pyridoxal 5'-Phosphate	C8 H10 N O6 P	247.02488	2.378	248.03216	No results	No results

Compound_ID	Name	Formula	Molecular Weight	RT [min]	m/z	mzCloud_Results	mzVault_Results
Com_158_all	Y-Glutamylcysteine	C8 H14 N2 O5 S	250.06257	1.635	251.06982	Full match	Full match
Com_159_all	N-Arachidonoyl-L-serine	C23 H37 N O4	391.27237	6.917	392.27964	Full match	Invalid mass
Com_160_all	SM 36:2;2O	C41 H81 N2 O6 P	750.56635	9.856	751.57362	No results	Invalid mass
Com_161_all	CAR 13:0	C20 H40 N O4	357.28801	7.338	358.29529	No results	Invalid mass
Com_162_all	Thymine	C5 H6 N2 O2	126.0431	4.857	127.05037	Full match	Full match
Com_163_all	Ergothioneine	C9 H15 N3 O2 S	229.08943	1.502	230.09671	No results	No results
Com_164_all	PC O-34:3	C42 H80 N O7 P	741.5684	8.626	742.57545	No results	Full match
Com_165_all	3-Methoxy prostaglandin F1α	C21 H38 O6	408.24904	7.6	409.2563	Invalid mass	No results
Com_166_all	L-Carnitine	C7 H15 N O3	161.10535	1.175	162.11263	No results	No results
Com_167_all	PC O-20:2	C28 H54 N O7 P	547.36443	9.738	548.37174	No results	Full match
Com_168_all	Palmitoyl sphingomyelin	C39 H79 N2 O6 P	702.56866	11.432	703.57582	Full match	Full match
Com_169_all	2-(2-hydroxy-3-methylbutanamido)-4-methylpentanoic acid	C11 H21 N O4	253.12959	5.525	254.13687	Invalid mass	No results
Com_170_all	PC O-38:8	C46 H78 N O7 P	787.53747	11.723	788.5448	No results	Invalid mass
Com_171_all	SM 8:1;2O/32:7	C45 H77 N2 O6 P	1545.10916	10.987	773.56186	No results	Invalid mass
Com_172_all	Spermine	C10 H26 N4	202.21605	3.165	203.22333	Full match	Full match
Com_173_all	Lysopc 18:2	C26 H50 N O7 P	519.33158	10.115	502.32725	No results	No results
Com_174_all	6-phenyl-1,2,3,4-tetrahydro-2,5-benzodiazocin-1-one	C16 H14 N2 O	232.09799	5.771	233.10526	Invalid mass	No results
Com_175_all	CAR 20:2	C27 H50 N O4	451.3667	9.145	452.37397	No results	Invalid mass
Com_176_all	LPC 36:5	C44 H80 N O7 P	787.53765	10.545	788.54547	No results	Invalid mass
Com_177_all	L-Methionine Methyl Ester	C6 H13 N O2 S	163.06687	4.074	164.07415	No results	No results
Com_178_all	PC 17:0_17:1	C42 H82 N O8 P	759.58039	8.572	760.58608	No results	Full match
Com_179_all	INK	C16 H31 N5 O5	188.11626	1.184	189.12354	Invalid mass	No match
Com_180_all	ERH	C17 H28 N8 O6	440.21786	7.575	441.22513	Invalid mass	No results
Com_181_all	L-arginine	C6 H14 N4 O2	174.11202	4.86	175.11921	No results	No results
Com_182_all	2-Arachidonoyl glycerol	C23 H38 O4	360.26637	8.848	361.27365	Invalid mass	No results
Com_183_all	SPB 16:1;2O	C16 H33 N O2	271.25131	7.309	272.25851	No results	Full match
Com_184_all	16,16-Dimethyl prostaglandin A2	C22 H34 O4	384.22787	7.72	385.23509	Invalid mass	No results
Com_185_all	PE O-16:1_20:4	C41 H74 N O7 P	741.53137	11.753	742.53832	No results	Invalid mass
Com_186_all	LPE 16:0	C21 H44 N O7 P	453.28591	9.245	454.29293	No results	Full match
Com_187_all	GLK	C14 H28 N4 O4	158.10569	4.727	317.21865	Invalid mass	No results
Com_188_all	cyclohexyl{4-[4-nitro-2-(1H-pyrrol-1-yl)phenyl]piperazino}methanone	C21 H26 N4 O3	382.19944	6.564	405.1888	Full match	No results
Com_189_all	PC 34:3	C42 H78 N O8 P	777.52653	9.839	778.53381	No results	Invalid mass
Com_190_all	2,3-dihydroxypropyl 12-methyltridecanoate	C17 H34 O4	324.22795	8.786	325.23518	Invalid mass	No results
Com_191_all	Linolelaidic Acid (C18:2N6T)	C18 H32 O2	280.24051	7.516	281.24792	No results	No results
Com_192_all	DLK	C16 H30 N4 O6	374.21697	4.725	188.11582	Full match	No results
Com_193_all	Oxymorphone	C17 H19 N O4	301.12754	2.203	302.13482	Invalid mass	No results
Com_194_all	CAR 19:1	C26 H50 N O4	439.36653	9.022	440.37383	No results	Invalid mass
Com_195_all	Lysopg 18:1	C24 H47 O9 P	510.29614	9.329	533.28536	No results	No results
Com_196_all	Cannabidivarin	C19 H26 O2	286.19709	7.366	287.2047	Invalid mass	No results
Com_197_all	S-Adenosylmethionine	C15 H22 N6 O5 S	199.06867	1.558	399.14456	Invalid mass	Invalid mass
Com_198_all	2-[(3S)-1-Benzyl-3-pyrrolidinyl]-1-methyl-1H-benzimidazole	C19 H21 N3	291.17372	8.384	292.18099	Full match	No results
Com_199_all	All trans-Retinal	C20 H28 O	284.21413	9.305	285.22143	No results	No results
Com_200_all	KKK	C18 H38 N6 O4	384.28512	9.184	385.2924	Invalid mass	No results
Com_201_all	PE O-16:1_18:2	C39 H74 N O7 P	699.52142	9.515	700.52759	No results	Full match
Com_202_all	(±)8-HEPE	C20 H30 O3	300.20913	7.49	301.2164	Invalid mass	No results
Com_203_all	N1-cyclooctyl-4-hydroxy-1-piperidinecarbothioamide	C14 H26 N2 O S	292.15351	5.853	293.16089	Invalid mass	No results
Com_204_all	PC O-38:5	C46 H84 N O7 P	793.59635	10.8	794.60362	No results	Full match
Com_205_all	N2,N2-Dimethylguanosine	C12 H17 N5 O5	311.12304	4.962	312.13027	No results	Full match
Com_206_all	Diphenylamine	C12 H11 N	169.08926	6.821	170.09654	Full match	No results
Com_207_all	Arachidonoyl amide	C20 H33 N O	303.25402	11.902	304.26129	Invalid mass	No results
Com_208_all	4-Hydroxy-3-methylbenzoic acid	C8 H8 O3	152.04737	1.545	153.05465	No results	No results
Com_209_all	(6E)-7-(2H-1,3-benzodioxol-5-yl)-1-(piperidin-1-yl)hept-6-en-1-one	C19 H25 N O3	315.17975	5.066	316.18702	Invalid mass	No results
Com_210_all	2-Aminobenzenesulfonic acid	C6 H7 N O3 S	173.01491	5.292	174.02225	No results	No results

Compound_ID	Name	Formula	Molecular Weight	RT [min]	m/z	mzCloud_Results	mzVault_Results
Com_211_all	9-Methyluric acid	C6 H6 N4 O3	182.04434	4.789	183.05162	Full match	No results
Com_212_all	PC 39:5	C47 H84 N O8 P	843.56393	10.674	844.57132	No results	Invalid mass
Com_213_all	1,7-bis(4-hydroxyphenyl)heptan-3-one	C19 H22 O3	336.1147	6.033	337.12197	Invalid mass	No results
Com_214_all	Y-L-Glutamyl-L-glutamic acid	C10 H16 N2 O7	276.09575	1.61	277.10302	Full match	No results
Com_215_all	PC O-17:0	C25 H52 N O7 P	509.34882	9.555	510.35615	No results	Full match
Com_216_all	Lysopc 14:0	C22 H46 N O7 P	467.30149	7.742	468.30877	No results	No results
Com_217_all	LPC 16:0-SN1	C24 H50 N O7 P	495.33275	9.801	496.34016	No results	Full match
Com_218_all	1-Methylguanine	C6 H7 N5 O	165.06533	4.769	166.0726	No results	No results
Com_219_all	MAG (18:3)	C21 H36 O4	352.26132	7.512	353.26847	No results	No results
Com_220_all	LPC 38:6	C46 H82 N O7 P	813.55115	10.424	814.55843	No results	Invalid mass
Com_221_all	PC 12:0_13:0	C33 H66 N O8 P	635.45331	10.389	636.46051	No results	Full match
Com_222_all	Coenzyme Q2	C19 H26 O4	318.18706	10.108	319.19434	Invalid mass	No results
Com_223_all	2-phenyl-2,4,6,7-tetrahydrothiino[4,3-c]pyrazol-3-ol	C12 H12 N2 O S	232.06885	5.826	233.0761	Invalid mass	No results
Com_224_all	PC 16:1_17:2	C41 H76 N O8 P	741.53204	10.712	742.53932	No results	Full match
Com_225_all	LPC 18:1-SN1	C26 H52 N O7 P	521.34876	10.088	522.35585	No results	Full match
Com_226_all	Sodium [dodecanoyl(methyl)amino]acetate	C15 H29 N O3	271.2149	7.75	272.22224	Full match	Full match
Com_227_all	Pipecolic acid	C6 H11 N O2	112.05264	5.173	113.0599	No results	Invalid mass
Com_228_all	LPC 38:7	C46 H80 N O7 P	811.53476	11.332	812.54204	No results	Invalid mass
Com_229_all	PC 40:7	C48 H82 N O8 P	853.55107	10.712	854.55835	No results	Invalid mass
Com_230_all	CAR 10:2	C17 H30 N O4	311.20981	5.953	312.21709	No results	Invalid mass
Com_231_all	Tetranor-12(S)-HETE	C16 H26 O3	248.17545	8.105	249.18273	Invalid mass	No results
Com_232_all	UR-144 N-Heptyl analog	C23 H33 N O	339.25263	5.442	362.24184	Invalid mass	No results
Com_233_all	5-Hydroxytryptophol	C10 H11 N O2	177.07907	5.211	160.07579	No results	No results
Com_234_all	6-(3-hydroxybutan-2-yl)-5-(hydroxymethyl)-4-methoxy-2H-pyran-2-one	C11 H16 O5	210.08715	5.692	211.09445	Invalid mass	No results
Com_235_all	4-(3,4-dihydro-2H-1,5-benzodioxepin-7-ylamino)-4-oxobutanoic acid	C13 H15 N O5	247.07992	5.696	248.0872	Invalid mass	No results
Com_236_all	PC O-32:3	C40 H76 N O7 P	713.53629	11.74	714.54364	No results	Full match
Com_237_all	CAR 20:1	C27 H52 N O4	453.38233	9.664	454.38957	No results	Invalid mass
Com_238_all	(2R)-2-[(2R,5S)-5-[(2S)-2-hydroxybutyl]oxolan-2-yl]propanoic acid	C11 H20 O4	216.13636	5.964	217.14369	Full match	No results
Com_239_all	16,16-Dimethyl prostaglandin A1	C22 H36 O4	386.24352	8.171	387.25076	Invalid mass	No results
Com_240_all	Enrofloxacin	C19 H22 F N3 O3	381.14747	6.361	382.15475	Invalid mass	No results
Com_241_all	HRH	C18 H28 N10 O4	448.2311	5.334	449.23837	Full match	No results
Com_242_all	1-Methyladenosine	C11 H15 N5 O4	281.11258	2.036	282.11986	No results	No results
Com_243_all	Etiocholanolone	C19 H30 O2	580.45562	10.448	598.48955	Invalid mass	No results
Com_244_all	6-Deoxy-D-glucose	C6 H12 O5	164.06873	5.637	203.03183	No results	No results
Com_245_all	Vitamin A	C20 H30 O	286.22961	10.041	287.23688	No results	No results
Com_246_all	1,2-dihydroxyheptadec-16-yn-4-yl acetate	C19 H34 O4	364.19986	8.078	365.20713	Invalid mass	No results
Com_247_all	13-OxoODE	C18 H30 O3	316.20405	6.797	317.21132	Invalid mass	No results
Com_248_all	Acetyl-L-carnitine	C9 H17 N O4	203.11601	1.062	204.12329	Full match	Full match
Com_249_all	3-Methyl-2-oxobutanoic acid	C5 H8 O3	116.04747	5.977	139.03668	No results	No results
Com_250_all	Lysopc 18:3	C26 H48 N O7 P	517.31531	10.114	518.32258	No results	No results
Com_251_all	Muscone	C16 H30 O	238.2297	9.791	239.23697	Full match	No results
Com_252_all	PC O-20:4	C28 H50 N O7 P	543.33301	8.005	544.34015	No results	Full match
Com_253_all	Limonin	C26 H30 O8	487.22851	4.786	488.23567	Invalid mass	No results
Com_254_all	2-hydroxy-6-[(8Z,11Z)-pentadeca-8,11,14-trien-1-yl]benzoic acid	C22 H30 O3	324.20687	8.47	325.21414	Invalid mass	No results
Com_255_all	PC O-35:3	C43 H82 N O7 P	755.58347	8.542	756.59075	No results	Full match
Com_256_all	SMK	C14 H28 N4 O5 S	728.35011	4.988	365.18233	Invalid mass	No results
Com_257_all	CAR 20:6	C27 H42 N O4	443.30383	7.629	444.3111	No results	Invalid mass
Com_258_all	LPC 17:2-SN2	C25 H48 N O7 P	1010.6351	8.499	506.32483	No results	Invalid mass
Com_259_all	2-{2-[(1-methyl-1H-pyrazol-5-yl)amino]-2-oxoethoxy}acetic acid	C8 H11 N3 O4	213.07689	5.735	214.08417	Invalid mass	No results
Com_260_all	4-morpholinobenzoic acid	C11 H13 N O3	229.07184	5.585	230.07912	Invalid mass	No results
Com_261_all	Cytidine 5'-diphosphocholine	C14 H26 N4 O11 P2	488.10802	1.58	489.1153	Full match	No results
Com_262_all	Lysopc 20:4	C28 H50 N O7 P	543.33245	8.978	544.33972	No results	No results
Com_263_all	PC 38:6	C46 H80 N O8 P	827.5299	10.242	828.53734	No results	Invalid mass

Compound_ID	Name	Formula	Molecular Weight	RT [min]	m/z	mzCloud_Results	mzVault_Results
Com_264_all	2-[2-(1-isobutylcyclohexyl)-1-methylethylidene]hydrazine-1-carboxamide	C14 H27 N3 O	291.16834	2.697	292.17561	Invalid mass	No results
Com_265_all	4-(anilinomethylidene)-3-methyl-4,5-dihydroisoxazol-5-one	C11 H10 N2 O2	202.07457	4.026	203.08185	Full match	No results
Com_266_all	3-hydroxy-2-octylpentanedioic acid	C13 H24 O5	242.15195	7.118	243.15938	Invalid mass	No results
Com_267_all	octadec-9-ynoic acid	C18 H32 O2	262.22987	10.114	263.23715	Invalid mass	No results
Com_268_all	1-(3,4-dimethoxyphenyl)ethan-1-one oxime	C10 H13 N O3	177.07965	1.826	178.08693	Invalid mass	No results
Com_269_all	Oxaceprol	C7 H11 N O4	173.06911	5.38	174.07638	No results	No results
Com_270_all	INH	C16 H26 N6 O5	382.19767	6.317	383.20494	Full match	No results
Com_271_all	Epitestosterone	C19 H28 O2	288.20908	6.736	289.21636	Full match	No results
Com_272_all	LPC 32:3	C40 H76 N O7 P	735.51853	11.791	736.52581	No results	Invalid mass
Com_273_all	VLH	C17 H29 N5 O4	367.22274	5.135	368.23001	Full match	No results
Com_274_all	PC O-14:0	C22 H46 N O7 P	429.34591	8.346	468.30914	No results	Invalid mass
Com_275_all	SM 34:1;2O	C39 H79 N2 O6 P	724.55535	11.709	725.56262	No results	Invalid mass
Com_276_all	4-oxododecanedioic acid	C12 H20 O5	261.15787	4.373	262.16515	Invalid mass	No results
Com_277_all	ELK	C17 H32 N4 O6	388.23206	5.097	389.23934	Full match	No results
Com_278_all	SPB 17:1;2O	C17 H35 N O2	285.26686	7.58	286.27415	No results	Full match
Com_279_all	LPC O-18:3	C26 H50 N O6 P	503.33762	9.561	504.34475	No results	Full match
Com_280_all	LNH	C16 H26 N6 O5	382.19842	5.842	383.20529	Invalid mass	No results
Com_281_all	N-Acetylsphingosine	C20 H39 N O3	363.27507	7.801	364.28234	Invalid mass	No results
Com_282_all	PC 40:5	C48 H86 N O8 P	857.57695	10.885	858.58423	No results	Invalid mass
Com_283_all	Ureidosuccinic acid	C5 H8 N2 O5	176.04288	5.656	177.05016	No results	No results
Com_284_all	CAR 19:2	C26 H48 N O4	437.35047	8.671	438.35775	No results	Invalid mass
Com_285_all	MMH	C16 H27 N5 O4 S2	400.13064	5.358	418.16454	Invalid mass	No results
Com_286_all	DPK	C15 H26 N4 O6	358.18509	2.166	359.19237	Full match	No results
Com_287_all	D-glutamine	C5 H10 N2 O3	146.06943	4.8	147.07671	No results	No results
Com_288_all	PC 26:3	C34 H62 N O8 P	643.42163	10.278	644.4292	No results	Full match
Com_289_all	Hydrocortisone acetate	C23 H32 O6	404.22079	9.319	427.21003	No results	No results
Com_290_all	PC 18:0	C26 H52 N O8 P	559.32753	7.861	560.33469	No results	Invalid mass
Com_291_all	PC O-18:2	C26 H50 N O7 P	519.33319	7.661	520.34036	No results	Full match
Com_292_all	Oleoyl-L-alpha-lysophosphatidic acid	C21 H40 Na O7 P	458.24123	9.493	459.2485	No results	No results
Com_293_all	Venlafaxine N-Oxide	C17 H27 N O3	293.19705	6.117	294.20433	Invalid mass	No results
Com_294_all	Methyl Palmitate	C17 H34 O2	292.23824	9.121	623.43902	Invalid mass	No results
Com_295_all	CAR 21:0	C28 H56 N O4	469.41365	10.079	470.42093	No results	Invalid mass
Com_296_all	1-adamantyl(piperidino)methanone	C16 H25 N O	225.20948	8.421	226.21674	Invalid mass	No results
Com_297_all	PC 12:0_13:1	C33 H64 N O8 P	633.43681	10.72	634.44411	No results	Full match
Com_298_all	S-Lactoyglutathione	C13 H21 N3 O8 S	379.10536	3.227	380.11264	No results	No results
Com_299_all	PC 17:1_17:2	C42 H78 N O8 P	755.54707	8.543	756.55338	No results	Full match
Com_300_all	CAR 18:4	C25 H42 N O4	419.30336	7.421	420.31063	No results	Invalid mass
Com_301_all	PC 17:0_18:2	C43 H82 N O8 P	771.57663	10.455	772.5839	No results	Full match
Com_302_all	PC 16:0_17:1	C41 H80 N O8 P	745.56244	9.161	728.55994	No results	Full match
Com_303_all	4-Methoxycinnamic Acid	C10 H10 O3	178.06323	1.878	179.07051	No results	No results
Com_304_all	Estrone	C18 H22 O2	287.18637	7.377	288.19364	Invalid mass	No results
Com_305_all	DL-Panthenol	C9 H19 N O4	205.13153	5.089	206.13918	No results	No results
Com_306_all	12(S)-HETE	C20 H32 O3	320.23511	7.381	303.23206	No results	No results
Com_307_all	PC O-18:3	C26 H48 N O7 P	517.31755	7.956	518.32477	No results	Full match
Com_308_all	Cys-Gly	C5 H10 N2 O3 S	178.04085	4.875	374.1139	No results	No results
Com_309_all	PC O-30:2	C38 H74 N O7 P	687.51991	10.739	688.52643	No results	Full match
Com_310_all	N1-[1-(2,4-dimethylphenyl)ethyl]-2,2-dimethylpropanamide	C15 H23 N O	216.14753	1.158	234.18139	Invalid mass	No results
Com_311_all	PC 10:0_13:1	C31 H60 N O8 P	605.40609	10.553	606.41335	No results	Full match
Com_312_all	Milbemycin A3 oxime	C31 H43 N O7	563.28652	5.843	564.29385	Invalid mass	No results
Com_313_all	Glycitin	C22 H22 O10	468.10391	6.046	469.11119	Invalid mass	No results
Com_314_all	PE O-16:1_22:5	C43 H76 N O7 P	749.53367	9.352	750.54094	No results	Full match
Com_315_all	PC 40:8	C48 H80 N O8 P	851.55332	11.211	852.5606	No results	Invalid mass
Com_316_all	CAR 24:0	C31 H62 N O4	511.46035	11.12	512.46763	No results	Invalid mass

Compound_ID	Name	Formula	Molecular Weight	RT [min]	m/z	mzCloud_Results	mzVault_Results
Com_317_all	Sphingosine (d18:1)	C18 H37 N O2	321.26451	8.025	322.27179	Invalid mass	No results
Com_318_all	PC O-37:7	C45 H78 N O7 P	775.53845	10.716	798.52631	No results	Invalid mass
Com_319_all	Choline Glycerophosphate	C8 H20 N O6 P	257.10231	2.202	258.10959	No results	No results
Com_320_all	PC 20:3_20:3	C48 H84 N O8 P	833.59274	8.564	834.60002	No results	Full match
Com_321_all	Monolaurin	C15 H30 O4	312.17278	7.591	335.16203	Invalid mass	No results
Com_322_all	Glycolithocholic acid	C26 H43 N O4	433.31713	8.386	434.32441	No results	No results
Com_323_all	PC O-40:7	C48 H84 N O7 P	817.59798	9.59	818.60526	No results	Full match
Com_324_all	WLH	C23 H30 N6 O4	454.23352	7.494	455.2408	Full match	No results
Com_325_all	CAR 19:3	C26 H46 N O4	435.33519	8.246	436.34247	No results	Invalid mass
Com_326_all	SM 8:1;2O/28:0	C41 H83 N2 O6 P	730.60027	8.575	731.60754	No results	Full match
Com_327_all	DGMG (18:2)	C33 H58 O14	678.38226	4.775	340.19841	No results	No results
Com_328_all	PC O-34:5	C42 H76 N O7 P	715.55223	10.859	716.55814	No results	Invalid mass
Com_329_all	PC O-32:0	C40 H82 N O7 P	719.58229	10.773	720.58957	No results	Full match
Com_330_all	PC O-36:9	C44 H72 N O7 P	757.50316	9.799	758.51043	No results	Full match
Com_331_all	Calcium D-Panthotenate	C18 H32 Ca N2 O10	476.16847	5.061	477.17575	No results	No results
Com_332_all	N-Methyldioctylamine	C17 H37 N	255.29272	8.355	256.3	Full match	No results
Com_333_all	N-Acetyl-DL-phenylalanine	C11 H13 N O3	207.08947	5.285	225.12316	No results	No results
Com_334_all	2-benzyl-6-hydroxy-2-azabicyclo[2.2.2]octan-3-one	C14 H17 N O2	231.12949	6.462	232.13676	Invalid mass	No results
Com_335_all	PC 36:4	C44 H80 N O8 P	803.53399	10.408	804.54175	No results	Invalid mass
Com_336_all	LysoPC 10:0	C18 H38 N O7 P	411.23884	7.947	412.24603	No results	No results
Com_337_all	PC 13:0_14:1	C35 H68 N O8 P	661.46997	11.017	662.47724	No results	Full match
Com_338_all	L-(-)-alpha-Amino-epsilon-Caprolactam	C6 H12 N2 O	128.09509	1.819	129.10236	No results	No results
Com_339_all	LPC 40:7	C48 H84 N O7 P	839.57076	11.377	840.57804	No results	Invalid mass
Com_340_all	PC O-33:3	C41 H78 N O7 P	727.55204	8.549	728.55932	No results	Full match
Com_341_all	PC 18:3_18:4	C44 H74 N O8 P	775.51262	10.253	776.51989	No results	Full match
Com_342_all	LPC 38:5	C46 H84 N O7 P	815.57671	11.665	816.58398	No results	Invalid mass
Com_343_all	1,3,5-Trimethylpyrazole	C6 H10 N2	110.08462	5.188	133.07388	No results	No results
Com_344_all	Octopamine	C8 H11 N O2	153.07921	4.996	307.16568	No results	No results
Com_345_all	Cystamine	C4 H12 N2 S2	152.04492	9.588	153.05222	No results	No results
Com_346_all	Dioctyldimethylammonium chloride	C18 H39 N	269.30838	8.284	270.31565	Full match	No results
Com_347_all	Biotin	C10 H16 N2 O3 S	244.08827	4.792	245.09554	No results	No results
Com_348_all	PPK	C16 H28 N4 O4	340.21145	4.76	341.21878	Full match	No results
Com_349_all	1-(2-furyl)pentane-1,4-dione	C9 H10 O3	188.04751	6.497	189.05479	Invalid mass	No results
Com_350_all	YPH	C20 H25 N5 O5	415.18625	4.815	208.6004	Full match	No results
Com_351_all	N-Acetyl-Asp-Glu	C11 H16 N2 O8	304.09012	9.598	153.05234	No results	No results
Com_352_all	Adenosine	C10 H13 N5 O4	267.09693	11.97	268.10421	Full match	Full match
Com_353_all	LPC 40:8	C48 H82 N O7 P	837.55864	9.825	838.56592	No results	Invalid mass
Com_354_all	acetoacetate	C4 H6 O3	102.03189	5.879	120.06573	No results	No results
Com_355_all	PC 16:0_16:1	C40 H78 N O8 P	731.54167	11.399	732.54895	No results	Invalid mass
Com_356_all	Asp-Phe	C13 H16 N2 O5	280.10634	5.492	281.11362	No results	No results
Com_357_all	Adrenosterone	C19 H24 O3	300.17294	6.656	323.16232	No results	No results
Com_358_all	PC 19:0_20:5	C47 H84 N O8 P	821.59221	10.746	822.59952	No results	Full match
Com_359_all	PC 17:0_17:0	C42 H84 N O8 P	761.58623	11.66	762.59351	No results	Invalid mass
Com_360_all	PC O-39:6	C47 H84 N O7 P	805.584	10.945	806.59062	No results	Invalid mass
Com_361_all	PC O-15:0	C23 H48 N O7 P	232.14539	8.112	482.32442	No results	Invalid mass
Com_362_all	Lysopa 16:0	C19 H39 O7 P	410.24304	9.285	393.23938	No results	No results
Com_363_all	PC O-34:2	C42 H82 N O7 P	743.5833	8.515	744.59058	No results	Full match
Com_364_all	Guggulsterone	C21 H28 O2	312.20877	9.835	335.19839	No results	No results
Com_365_all	PAF C-16	C26 H54 N O7 P	523.36425	10.861	524.37158	No results	No results
Com_366_all	Tetradecanedioic acid	C14 H26 O4	258.1834	8.189	259.19068	No results	No results
Com_367_all	PC O-32:2	C40 H78 N O7 P	715.55262	10.101	716.5589	No results	Full match
Com_368_all	Lysope 14:0	C19 H40 N O7 P	425.2546	8.527	426.26154	No results	No results
Com_369_all	Levodopa	C9 H11 N O4	197.06788	5.192	395.1442	No results	No results

Compound_ID	Name	Formula	Molecular Weight	RT [min]	m/z	mzCloud_Results	mzVault_Results
Com_370_all	PC 17:0_18:5	C43 H76 N O8 P	765.53227	8.433	766.53955	No results	Full match
Com_371_all	3-Methoxytyramine	C9 H13 N O2	167.09457	1.744	168.10185	No results	No results
Com_372_all	Kynurenic acid	C10 H7 N O3	189.04294	5.549	190.05016	No results	No results
Com_373_all	Heptadecanoic Acid	C17 H34 O2	292.2405	6.345	293.24777	Invalid mass	No results
Com_374_all	Glutathione	C10 H17 N3 O6 S	307.08421	9.61	308.09149	No results	No results
Com_375_all	PC 16:1_16:1	C40 H76 N O8 P	729.53347	11.124	730.54075	No results	Full match
Com_376_all	5'-Deoxy-5'-(Methylthio)Adenosine	C11 H15 N5 O3 S	297.08971	3.02	298.09698	No results	No results
Com_377_all	Methyl 3-indolyacetate	C11 H11 N O2	189.07927	4.887	190.08655	No results	No results
Com_378_all	PC 16:2_17:2	C41 H74 N O8 P	739.51476	11.805	740.52203	No results	Full match
Com_379_all	Isophorone	C9 H14 O	138.10453	6.024	139.1118	Full match	No results
Com_380_all	DL-Tryptophan	C11 H12 N2 O2	204.09005	5.209	188.07078	Full match	Full match
Com_381_all	Leucylproline	C11 H20 N2 O3	228.14756	5.199	229.15484	Full match	No results
Com_382_all	LPC O-16:1	C24 H50 N O6 P	479.33783	9.851	480.34515	No results	Full match
Com_383_all	DL-Norvaline	C5 H11 N O2	117.07905	1.372	118.08632	No results	No results
Com_384_all	L-Glutathione (reduced)	C10 H17 N3 O6 S	307.08388	1.984	308.09116	Full match	Full match
Com_385_all	Nicotinic acid	C6 H5 N O2	123.03219	1.938	124.03947	No results	Full match
Com_386_all	isoleucine	C6 H13 N O2	131.0947	3.065	132.10198	No results	No results
Com_387_all	L-Tyrosine	C9 H11 N O3	181.07413	2.346	182.08141	Full match	Full match
Com_388_all	Inosine	C10 H12 N4 O5	268.08091	3.542	269.08813	Full match	Full match
Com_389_all	Prostaglandin H1	C20 H34 O5	336.2278	8.339	337.23499	Invalid mass	No results
Com_390_all	LPC O-16:0	C24 H52 N O6 P	481.35342	10.01	482.3607	No results	Full match
Com_391_all	Creatinine	C4 H7 N3 O	113.05898	0.53	114.06626	Full match	Full match
Com_392_all	LPE 18:2	C23 H44 N O7 P	477.286	8.889	478.29325	No results	Full match
Com_393_all	L-Histidine	C6 H9 N3 O2	155.06965	1.352	156.07693	Full match	Full match
Com_394_all	Valylproline	C10 H18 N2 O3	214.13206	4.856	215.13933	Full match	No results
Com_395_all	Gly-Phe	C11 H14 N2 O3	222.10073	5.076	223.108	No results	No results
Com_396_all	LPE 18:1	C23 H46 N O7 P	479.30167	9.645	480.30896	No results	Full match
Com_397_all	α-Aspartylphenylalanine	C13 H16 N2 O5	280.10614	5.265	281.11342	Full match	Full match
Com_398_all	Gamma-Caprolactone	C6 H10 O2	114.06816	5.061	115.07542	No results	No results
Com_399_all	Methyltestosterone	C20 H30 O2	302.22472	9.895	303.23199	No results	No results
Com_400_all	D-Proline	C5 H9 N O2	115.06342	1.463	116.07069	No results	No results
Com_401_all	Chaetocin	C30 H28 N6 O6 S4	696.09493	1.788	697.1022	No results	No results
Com_402_all	L-Pyroglutamic acid	C5 H7 N O3	147.05331	2.277	148.06058	Invalid mass	Invalid mass
Com_403_all	L-Palmitoylcarnitine	C23 H45 N O4	399.33503	9.804	438.29819	No results	No results
Com_404_all	Glycyl-L-leucine	C8 H16 N2 O3	188.11632	5.174	189.1236	Full match	Full match
Com_405_all	Isoproterenol	C11 H17 N O3	211.12109	5.006	212.12805	No results	No results
Com_406_all	Sedoheptulose 1,7-bisphosphate	C7 H16 O13 P2	370.00597	1.311	371.01325	No results	No results
Com_407_all	NADH	C21 H29 N7 O14 P2	665.12508	1.853	333.56981	No results	No results
Com_408_all	p-Hydroxybenzaldehyde	C7 H6 O2	122.03698	2.352	123.04426	No results	No results
Com_409_all	L-Leucyl-L-Alanine	C9 H18 N2 O3	202.13191	1.612	185.12862	No results	No results
Com_410_all	LPC O-18:0	C26 H56 N O6 P	509.385	10.887	510.39229	No results	Full match
Com_411_all	Ala-Gln	C8 H15 N3 O4	217.1065	1.366	218.1138	No results	No results
Com_412_all	L-Kynurenine	C10 H12 N2 O3	208.08511	4.823	209.09239	Full match	Full match
Com_413_all	L-Alanyl-L-proline	C8 H14 N2 O3	186.10068	2.18	187.10795	Full match	No results
Com_414_all	N6-Succinyl Adenosine	C14 H17 N5 O8	383.10791	5.007	384.11519	No results	No results
Com_415_all	N-Acetyl-L-leucine	C8 H15 N O3	173.10536	1.477	174.11262	Full match	No results
Com_416_all	LPC O-18:1	C26 H54 N O6 P	507.36941	10.21	508.3767	No results	Full match
Com_417_all	LPC 16:1	C24 H48 N O7 P	515.29948	8.591	516.30676	No results	Invalid mass
Com_418_all	gamma-Glutamylleucine	C11 H20 N2 O5	260.13751	5.382	261.1448	No results	Full match
Com_419_all	N-Methylhydantoin	C4 H6 N2 O2	114.04315	5.206	132.07698	No results	No results
Com_420_all	LPC O-15:1	C23 H48 N O6 P	465.32241	9.297	466.3297	No results	Full match
Com_421_all	L-Alanyl-L-Lysine	C9 H19 N3 O3	217.14279	1.345	218.15008	No results	No results
Com_422_all	Uric acid	C5 H4 N4 O3	168.02848	1.808	169.03576	Full match	No results

Compound_ID	Name	Formula	Molecular Weight	RT [min]	m/z	mzCloud_Results	mzVault_Results
Com_423_all	N-Formylkynurenine	C11 H12 N2 O4	236.07997	4.901	237.08725	No results	No results
Com_424_all	Alanyltyrosine	C12 H16 N2 O4	252.11126	4.687	253.11857	Full match	No results
Com_425_all	3-Methylhistidine	C7 H11 N3 O2	169.08523	1.522	170.09253	Full match	Full match
Com_426_all	Lysope 16:0	C21 H44 N O7 P	453.28575	9.42	454.293	No results	No results
Com_427_all	Taurine	C2 H7 N O3 S	125.01479	1.337	126.022	Full match	No results
Com_428_all	L-Adrenaline	C9 H13 N O3	183.0899	5.189	201.12372	No results	No results
Com_429_all	S-Adenosylhomocysteine	C14 H20 N6 O5 S	384.1219	2.525	385.12908	Full match	Full match
Com_430_all	Ne-(1-Carboxymethyl)-L-lysine	C8 H16 N2 O4	204.11129	2.707	205.11857	No results	No results
Com_431_all	1,3-Dimethyluracil	C6 H8 N2 O2	140.05873	1.99	141.06606	No results	No results
Com_432_all	Thr-Leu	C10 H20 N2 O4	232.14257	2.886	233.14985	No results	No results
Com_433_all	LPC O-15:0	C23 H50 N O6 P	467.33793	9.561	468.34523	No results	Full match
Com_434_all	LPE 22:4	C27 H48 N O7 P	529.31698	9.507	530.32416	No results	Full match
Com_435_all	Thromboxane B2	C20 H34 O6	392.21779	6.317	393.22514	Invalid mass	No results
Com_436_all	LPC 18:2	C26 H50 N O7 P	541.31696	8.568	542.32397	No results	Invalid mass
Com_437_all	Porphobilinogen	C10 H14 N2 O4	226.09612	2.309	227.1034	No results	No results
Com_438_all	N-Acetylvaline	C7 H13 N O3	159.08979	1.353	160.09706	No results	No results
Com_439_all	LPE 20:3	C25 H46 N O7 P	481.3196	9.223	504.30887	No results	Invalid mass
Com_440_all	Cytidine 5'-Monophosphate-N-Acetylneuraminic Acid	C20 H31 N4 O16 P	614.14945	2.27	308.082	No results	No results
Com_441_all	cis-7-Hexadecenoic Acid	C16 H30 O2	254.22477	8.279	255.23204	No results	No results
Com_442_all	Prostaglandin A3	C20 H28 O4	332.19731	6.748	333.2043	Full match	No results
Com_443_all	Prostaglandin J2	C20 H30 O4	334.2141	6.758	335.221	Full match	No results
Com_444_all	Methyldopa	C10 H13 N O4	211.08472	5.191	212.092	No results	No results
Com_445_all	LPC 20:4	C28 H50 N O7 P	565.31487	9.053	566.32214	No results	Invalid mass
Com_446_all	LPC 20:3	C28 H52 N O7 P	567.33009	9.452	568.33736	No results	Invalid mass
Com_447_all	Methionine	C5 H11 N O2 S	132.02467	5.538	133.03189	Invalid mass	Invalid mass
Com_448_all	o-Cresol	C7 H8 O	108.05769	4.969	109.06496	No results	No results
Com_449_all	LPC 18:3	C26 H48 N O7 P	539.2994	8.62	540.30668	No results	Invalid mass
Com_450_all	23-Nordeoxycholic acid	C23 H38 O4	378.27716	8.852	361.27393	No results	No results
Com_451_all	N-Acetyl-L-carnosine	C11 H16 N4 O4	268.11736	1.828	269.12464	Full match	No results
Com_452_all	2,3-Dinor-TXB2	C18 H30 O6	342.20524	5.906	365.19452	No results	No results
Com_453_all	LPE 20:5	C25 H42 N O7 P	477.28839	8.377	500.27768	No results	Invalid mass
Com_454_all	8-Aminooctanoic acid	C8 H17 N O2	159.12615	4.604	160.13343	No results	No results
Com_455_all	Glu-Gln	C10 H17 N3 O6	275.11188	1.658	276.11916	No results	Full match
Com_456_all	Estriol	C18 H24 O3	288.17269	6.272	289.17995	Full match	No results
Com_457_all	Gly-Tyr	C11 H14 N2 O4	238.09556	4.688	239.10284	No results	No results
Com_458_all	LPC 18:0	C26 H54 N O7 P	545.34612	10.342	546.35339	No results	Invalid mass
Com_459_all	LPC O-14:0	C22 H48 N O6 P	453.3223	9.109	454.32968	No results	Full match
Com_460_all	SM 8:1;2O/26:1	C39 H77 N2 O6 P	700.55298	9.713	701.5602	No results	Full match
Com_461_all	Propionyl-L-carnitine	C10 H19 N O4	217.13161	1.768	218.13888	No results	No results
Com_462_all	L-Cysteine-glutathione gisulfide	C13 H22 N4 O8 S2	426.08791	1.475	427.09518	No results	No results
Com_463_all	Tetrahydrocorticosterone	C21 H34 O4	367.27245	7.157	368.27976	Invalid mass	Invalid mass
Com_464_all	3,8,9-trihydroxy-10-propyl-3,4,5,8,9,10-hexahydro-2H-oxecin-2-one	C12 H20 O5	266.11337	5.789	267.12064	Invalid mass	No results
Com_465_all	Melatonin	C13 H16 N2 O2	232.12149	5.363	233.12877	No results	No results
Com_466_all	Gly-Tyr-Ala	C14 H19 N3 O5	309.13297	4.736	310.14025	No results	No results
Com_467_all	Lactitol	C12 H24 O11	344.13336	2.299	345.14063	No results	No results
Com_468_all	Nonadecanoic acid	C19 H38 O2	298.28751	10.768	299.29478	No results	No results
Com_469_all	LPC O-17:2	C25 H50 N O6 P	491.33823	9.635	492.34551	No results	Full match
Com_470_all	4-Hydroxyretinoic Acid	C20 H28 O3	316.20387	7.754	339.19337	No results	No results
Com_471_all	Phenylpyruvic acid	C9 H8 O3	164.04758	4.687	182.08145	No results	No results
Com_472_all	Anserine	C10 H16 N4 O3	240.1225	2.297	121.06853	No results	No results
Com_473_all	LPE 18:3	C23 H42 N O7 P	493.28111	7.154	476.27771	No results	Invalid mass
Com_474_all	5-Methoxyindole-3-Carbaldehyde	C10 H9 N O2	175.0636	5.539	176.07087	No results	No results
Com_475_all	Taurocholic acid	C26 H45 N O7 S	515.2925	6.496	498.28909	Full match	No results

Compound_ID	Name	Formula	Molecular Weight	RT [min]	m/z	mzCloud_Results	mzVault_Results
Com_476_all	1-Palmitoyl-Sn-Glycero-3-Phosphocholine	C24 H50 N O7 P	495.33243	10.014	496.33971	No results	No results
Com_477_all	glutathione disulfide	C20 H32 N6 O12 S2	612.15252	4.644	307.08354	No results	No results
Com_478_all	5-Phenylvaleric Acid	C11 H14 O2	178.09884	7.354	357.20329	No results	No results
Com_479_all	Flavin adenine dinucleotide (FAD)	C27 H33 N9 O15 P2	785.15853	5.089	786.16582	Full match	Full match
Com_480_all	Acetylcarnitine	C9 H17 N O4	203.11603	8.801	204.12331	No results	No results
Com_481_all	5-Hydroxyindole	C8 H7 N O	133.05228	5.184	267.1113	No results	No results
Com_482_all	2-Methylbutyroylcarnitine	C12 H23 N O4	245.16295	6.129	246.17023	No results	No results
Com_483_all	Boc-beta-cyano-L-alanine	C9 H14 N2 O4	214.0955	5.379	215.10278	No results	No results
Com_484_all	Prostaglandin B2	C20 H30 O4	334.21437	5.916	335.22165	No results	No results
Com_485_all	11-Ketoetiocholanolone	C19 H28 O3	304.20381	7.178	305.21109	No results	No results
Com_486_all	Undecanedioic acid	C11 H20 O4	216.13528	4.87	471.233	No results	No results
Com_487_all	Lysopc 16:1	C24 H48 N O7 P	493.31724	9.711	494.32419	No results	No results
Com_488_all	Leu-Pro	C11 H20 N2 O3	228.14747	9.58	229.15469	No results	No results
Com_489_all	10-Undecenoic acid	C11 H20 O2	184.14647	5.704	185.15375	No results	No results
Com_490_all	Pantetheine	C11 H22 N2 O4 S	278.12927	6.173	296.16284	No results	No results
Com_491_all	Palmitic Acid	C16 H32 O2	256.24023	10.487	255.23296	Full match	No results
Com_492_all	alpha-Ketoglutaric acid	C5 H6 O5	146.02136	1.46	191.01957	No results	No results
Com_493_all	Tretinoin	C20 H28 O2	300.20883	9.142	299.20156	Full match	No results
Com_494_all	Adrenic acid	C22 H36 O2	332.27143	10.721	331.26416	Full match	Full match
Com_495_all	cis-Aconitic acid	C6 H6 O6	192.02686	2.142	191.01959	Invalid mass	Invalid mass
Com_496_all	L-Anserine	C10 H16 N4 O3	240.12211	1.363	239.11483	Full match	Full match
Com_497_all	Mestranol	C21 H26 O2	310.18154	8.077	309.17426	No results	Invalid mass
Com_498_all	(2R)-2,3-Dihydroxypropanoic acid	C3 H6 O4	106.02639	1.393	105.01911	Full match	Full match
Com_499_all	LPC 18:1	C26 H52 N O7 P	567.35373	9.684	566.34646	No results	Invalid mass
Com_500_all	LPE O-16:1	C21 H44 N O6 P	437.29063	9.793	436.28335	No results	Full match
Com_501_all	Pentadecanoic acid	C15 H30 O2	242.22456	9.931	241.21728	Full match	No results
Com_502_all	LPE O-18:2	C23 H46 N O6 P	463.30628	9.99	462.29901	No results	Full match
Com_503_all	16-Hydroxyhexadecanoic acid	C16 H32 O3	254.2245	9.922	253.21722	Invalid mass	No results
Com_504_all	2-Furoic acid	C5 H4 O3	112.01584	1.464	111.00856	No results	No results
Com_505_all	D-Mannose 6-phosphate	C6 H13 O9 P	260.02962	1.226	259.02234	Full match	Not the top hit
Com_506_all	3-[(methoxycarbonyl)amino]-2,2,3-trimethylbutanoic acid	C9 H17 N O4	203.11576	5.39	202.10849	Full match	No results
Com_507_all	acetyl phospahte	C2 H5 O5 P	139.98721	1.262	138.9798	No results	No results
Com_508_all	Prostaglandin F3α	C20 H32 O5	398.23372	8.366	397.22644	Invalid mass	No results
Com_509_all	D-Threose	C4 H8 O4	120.04203	1.356	101.02424	No results	No results
Com_510_all	Cyclohexaneacetic acid	C8 H14 O2	142.09921	5.802	187.09741	No results	No results
Com_511_all	20-Hydroxy-(5Z,8Z,11Z,14Z)-eicosatetraenoic acid	C20 H32 O3	320.23511	8.077	319.22783	Full match	Full match
Com_512_all	2-Methylbutyl beta-D-glucopyranoside	C11 H22 O6	296.14821	1.394	295.14093	Invalid mass	No results
Com_513_all	FAHFA 18:1/20:3	C38 H66 O4	586.49342	10.705	585.48615	No results	Full match
Com_514_all	LPE O-17:1	C22 H46 N O6 P	451.30619	10.092	450.29891	No results	Full match
Com_515_all	trans-10-Heptadecenoic Acid	C17 H32 O2	268.24021	10.27	267.23293	Full match	No results
Com_516_all	7-Methylxanthine	C6 H6 N4 O2	166.04746	1.494	165.04013	Invalid mass	No results
Com_517_all	FAHFA 18:2/20:4	C38 H62 O4	582.46218	10.119	581.4549	No results	Full match
Com_518_all	LPI 20:4	C29 H49 O12 P	620.29615	8.559	619.28887	No results	Full match
Com_519_all	FAHFA 18:0/20:2	C38 H70 O4	590.52493	11.377	589.51765	No results	Full match
Com_520_all	Gluconic acid	C6 H12 O7	196.05802	1.481	195.05074	Full match	Full match
Com_521_all	LPC 20:5	C28 H48 N O7 P	587.32234	8.362	586.31506	No results	Invalid mass
Com_522_all	8(S),15(S)-DiHETE	C20 H32 O4	336.23014	8.032	335.22291	Full match	No results
Com_523_all	N-Acetylaspartic acid	C6 H9 N O5	175.04776	1.468	174.04048	Full match	Full match
Com_524_all	FAHFA 16:0/18:2	C34 H62 O4	534.46216	10.488	533.45488	No results	Full match
Com_525_all	Uridine monophosphate (UMP)	C9 H13 N2 O9 P	324.03579	1.429	323.02851	Full match	Full match
Com_526_all	11(Z),14(Z)-Eicosadienoic Acid	C20 H36 O2	308.27162	10.976	307.26434	Full match	No results
Com_527_all	LPI 18:1	C27 H51 O12 P	598.31189	9.212	597.30461	No results	Full match
Com_528_all	11(E)-Eicosenoic Acid	C20 H38 O2	310.28734	11.61	309.28006	Full match	No results

Compound_ID	Name	Formula	Molecular Weight	RT [min]	m/z	mzCloud_Results	mzVault_Results
Com_529_all	2-Isopropylmalic acid	C7 H12 O5	130.06287	5.86	129.05559	Invalid mass	No results
Com_530_all	(±)9-HpODE	C18 H32 O4	312.2302	6.793	311.22293	Full match	No results
Com_531_all	FAHFA 18:1/3:0	C21 H38 O4	354.27692	10.788	353.26964	No results	Full match
Com_532_all	Suberic acid	C8 H14 O4	174.08898	5.619	173.08171	Full match	Full match
Com_533_all	D-(-)-Fructose	C6 H12 O6	180.0632	1.347	179.05593	Full match	Full match
Com_534_all	LPG 18:2	C24 H45 O9 P	508.27988	8.684	507.27261	No results	Full match
Com_535_all	8(S)-Hydroxy-(5Z,9E,11Z,14Z)-eicosatetraenoic acid	C20 H32 O3	320.23511	8.346	319.22784	Full match	Full match
Com_536_all	LPI 18:2	C27 H49 O12 P	596.29632	8.586	595.28904	No results	Full match
Com_537_all	Corchorifatty acid F	C18 H32 O5	328.2252	6.334	327.21793	Full match	No results
Com_538_all	(+/-)8(9)-DiHET	C20 H34 O4	338.24344	10.948	337.23616	Invalid mass	No results
Com_539_all	D-Sedoheptulose 7-phosphate	C7 H15 O10 P	290.04028	1.248	289.03303	Full match	Full match
Com_540_all	15-Deoxy-Δ12,14-prostaglandin D2	C20 H30 O4	316.20374	8.047	315.19646	Invalid mass	No results
Com_541_all	Corticosterone	C21 H30 O4	346.21192	9.6	345.20464	Invalid mass	No results
Com_542_all	LPG 18:1	C24 H47 O9 P	510.29556	9.309	509.28828	No results	Full match
Com_543_all	LPS 18:2	C24 H44 N O9 P	521.27517	8.943	520.2679	No results	Full match
Com_544_all	LPI 20:3	C29 H51 O12 P	622.31161	8.957	621.30434	No results	Full match
Com_545_all	Feruloyl Putrescine	C14 H20 N2 O3	264.14738	5.358	263.14011	No results	No results
Com_546_all	Maleic acid	C4 H4 O4	116.01075	2.006	115.00348	No results	Full match
Com_547_all	LPS 18:0	C24 H48 N O9 P	479.301	10.197	478.29372	No results	Invalid mass
Com_548_all	Tyrosylalanine	C12 H16 N2 O4	252.11102	5.199	251.10374	Full match	No results
Com_549_all	Lactobionic acid	C12 H22 O12	358.11244	2.904	357.10516	No results	No results
Com_550_all	LPG 16:0	C22 H45 O9 P	484.28009	9.285	483.27281	No results	Full match
Com_551_all	Resolvin E1	C20 H30 O5	350.20933	7.019	349.2021	Full match	No results
Com_552_all	FAHFA 16:1/18:3	C34 H58 O4	530.43078	9.829	529.42351	No results	Invalid mass
Com_553_all	D-Ribose-1-phosphate	C5 H11 O8 P	194.04225	1.468	193.03502	Invalid mass	Invalid mass
Com_554_all	12,13-EODE	C18 H32 O3	296.23508	8.623	295.22781	No results	No results
Com_555_all	N-Acetyl-1-aspartylglutamic acid	C11 H16 N2 O8	258.08519	1.544	303.0834	Invalid mass	Invalid mass
Com_556_all	19(R)-Hydroxy-prostaglandin E2	C20 H32 O6	368.21972	6.552	367.2125	Full match	Full match
Com_557_all	LPS 18:1	C24 H46 N O9 P	523.29086	9.545	522.28358	No results	Full match
Com_558_all	Δ17-6-keto prostaglandin F1α	C20 H32 O6	350.2094	6.558	349.20212	Invalid mass	No match
Com_559_all	(+/-)9,10-dihydroxy-12Z-octadecenoic acid	C18 H34 O4	332.25631	7.169	313.23847	Invalid mass	No results
Com_560_all	L-Tyrosinemethylester	C10 H13 N O3	195.08913	9.17	435.17645	No results	No results
Com_561_all	LPC O-18:2	C26 H52 N O6 P	551.35864	10.059	550.35137	No results	Invalid mass
Com_562_all	LPI 20:5	C29 H47 O12 P	618.28046	8.044	617.27318	No results	Full match
Com_563_all	LysoPE 18:0	C23 H48 N O7 P	481.31718	9.457	480.30991	No results	No results
Com_564_all	LPC 17:0	C25 H52 N O7 P	555.35346	9.891	554.34618	No results	Invalid mass
Com_565_all	Monobutyl phthalate	C12 H14 O4	222.08912	6.227	221.08185	Full match	No results
Com_566_all	N-Acetyl-D-alloisoleucine	C8 H15 N O3	173.10509	5.621	172.09781	Full match	Full match
Com_567_all	Guanosine monophosphate (GMP)	C10 H14 N5 O8 P	363.05781	1.447	362.05053	Full match	Full match
Com_568_all	(±)9(10)-DiHOME	C18 H34 O4	314.2458	7.155	313.23853	Full match	No results
Com_569_all	L-Methionine sulfone	C5 H11 N O4 S	181.04066	1.38	180.03337	No results	No results
Com_570_all	LPE O-18:0	C23 H50 N O6 P	467.33735	10.77	466.33007	No results	Full match
Com_571_all	12-epi Leukotriene B4	C20 H32 O4	318.21944	7.504	317.21216	Invalid mass	No results
Com_572_all	FAHFA 18:2/3:0	C21 H36 O4	352.2613	10.224	351.25402	No results	Full match
Com_573_all	8-iso-15-keto Prostaglandin F2α	C20 H32 O5	334.21448	7.242	333.2072	Invalid mass	No results
Com_574_all	FAHFA 18:1/19:2	C37 H66 O4	574.4935	10.791	573.48622	No results	Full match
Com_575_all	Arginine	C6 H14 N4 O2	174.11134	1.331	173.10406	No results	Full match
Com_576_all	12-Epileukotriene B4	C20 H32 O4	336.23	7.769	335.22279	Full match	Full match
Com_577_all	LPC O-14:1	C22 H46 N O6 P	497.31196	8.827	496.30472	No results	Invalid mass
Com_578_all	LPE O-15:1	C20 H42 N O6 P	423.27509	9.251	422.26781	No results	Full match
Com_579_all	LPS 20:3	C26 H46 N O9 P	547.29144	9.036	546.28497	No results	Full match
Com_580_all	PC(16:0/16:0)	C40 H80 N O8 P	733.56229	9.838	732.55501	No results	No results
Com_581_all	FAHFA 20:4/3:0	C23 H36 O4	376.26112	10.13	375.25385	No results	Full match

Compound_ID	Name	Formula	Molecular Weight	RT [min]	m/z	mzCloud_Results	mzVault_Results
Com_582_all	12-Hydroxydodecanoic acid	C12 H24 O3	216.17238	6.902	215.1651	Full match	No results
Com_583_all	Cholic acid	C24 H40 O5	408.28753	7.275	407.28024	Full match	Full match
Com_584_all	LPE O-16:2	C21 H42 N O6 P	435.27483	9.24	434.26755	No results	Full match
Com_585_all	Ethylmalonate	C5 H8 O4	132.04203	4.695	131.03475	No results	No results
Com_586_all	FAHFA 20:4/20:4	C40 H62 O4	606.46239	10.068	605.45511	No results	Full match
Com_587_all	8-iso Prostaglandin A2	C20 H30 O4	316.20375	7.369	315.19648	Invalid mass	No results
Com_588_all	LPI 22:5	C31 H51 O12 P	646.31178	8.815	645.3045	No results	Full match
Com_589_all	trans-Cinnamic acid	C9 H8 O2	148.05225	4.945	147.04497	Full match	No results
Com_590_all	(±)18-HEPE	C20 H30 O3	318.21937	7.685	317.21209	Full match	No results
Com_591_all	L-Ornithine	C5 H12 N2 O2	132.08962	1.335	131.08234	No results	No results
Com_592_all	Ethyl chrysanthemumate	C12 H20 O2	196.14617	6.636	195.13889	No results	No results
Com_593_all	LPE O-18:1	C23 H48 N O6 P	465.32187	10.138	464.31459	No results	Full match
Com_594_all	Docosatrienoic acid	C22 H38 O2	334.28728	11.306	333.28	Full match	No results
Com_595_all	4-[2-(2-oxo-1-imidazolidinyl)ethyl]-1lambda~6~,4-thiazinane-1,1-dione	C9 H17 N3 O3 S	248.10072	1.508	247.09344	Invalid mass	No match
Com_596_all	Dimetghyl 4-Hydroxyisophthalate	C10 H10 O5	210.05272	5.532	209.04543	No results	No results
Com_597_all	LPG 18:0	C24 H49 O9 P	512.31118	10.363	511.30391	No results	Full match
Com_598_all	Taurochenodeoxycholic acid	C26 H45 N O6 S	499.29679	7.062	498.28952	Full match	No results
Com_599_all	Prostaglandin K2	C20 H30 O5	332.19893	7.221	331.19167	Invalid mass	No results
Com_600_all	Ethyl myristate	C16 H32 O2	256.24006	10.75	255.23278	Full match	No results
Com_601_all	1-Caffeoylquinic Acid	C16 H18 O9	354.09388	1.331	353.0866	No results	No results
Com_602_all	15(S)-HpEPE	C20 H30 O4	334.21443	7.769	333.20716	Full match	No results
Com_603_all	gamma-Glutamyltyrosine	C14 H18 N2 O6	310.11675	4.797	309.10947	No results	Full match
Com_604_all	3-Hydroxydecanoic acid	C10 H20 O3	188.14111	6.903	187.13383	Full match	Full match
Com_605_all	N-Acetyl-L-histidine	C8 H11 N3 O3	197.07976	1.408	196.07249	No results	No results
Com_606_all	LPC O-16:2	C24 H48 N O6 P	523.3269	9.279	522.31962	No results	Invalid mass
Com_607_all	2-Hydroxyestradiol	C18 H24 O3	288.16855	5.551	287.16128	Invalid mass	No results
Com_608_all	20-Carboxy-Leukotriene B4	C20 H30 O6	366.20416	5.966	365.19689	No results	No results
Com_609_all	LNAPE 18:0/N-16:0	C39 H78 N O8 P	719.54642	10.477	718.53905	No results	Full match
Com_610_all	Pyridoxal-5'-phosphate	C8 H10 N O6 P	247.02456	2.382	246.01729	No results	Full match
Com_611_all	3'-Dephosphocoenzyme A	C21 H35 N7 O13 P2 S	687.14898	4.899	686.1417	Full match	No results
Com_612_all	LPI 20:2	C29 H53 O12 P	624.32707	9.529	623.31979	No results	Full match
Com_613_all	FAHFA 17:0/19:2	C36 H66 O4	562.49352	10.808	561.48624	No results	Full match
Com_614_all	LPS 20:4	C26 H44 N O9 P	545.27536	8.684	544.26808	No results	Full match
Com_615_all	Neopterin	C9 H11 N5 O4	253.08067	6.067	252.0734	Full match	No results
Com_616_all	Lauric Acid	C12 H24 O2	200.17748	6.97	199.1702	No results	No results
Com_617_all	3-Phosphoglyceric acid	C3 H7 O7 P	185.99268	1.422	230.99086	Full match	No results
Com_618_all	Royal jelly acid	C10 H18 O3	186.12537	7.147	167.10751	No results	No results
Com_619_all	2-Methylpentanedioic acid	C6 H10 O4	146.05776	1.077	145.05048	No results	No results
Com_620_all	FAHFA 20:4/17:2	C37 H60 O4	568.44663	10.029	567.43936	No results	Full match
Com_621_all	4-amino-2-(4-chlorophenyl)-6-(methylthio)pyrimidine-5-carbonitrile	C12 H9 Cl N4 S	276.02481	1.244	275.01757	Full match	No match
Com_622_all	LPG 16:1	C22 H43 O9 P	482.26441	8.559	481.25713	No results	Full match
Com_623_all	LPI 22:4	C31 H53 O12 P	648.32766	9.357	647.32038	No results	Full match
Com_624_all	Lauric acid ethyl ester	C14 H28 O2	228.2089	9.47	227.20163	Full match	No results
Com_625_all	α-D-Glucose-1,6-bisphosphate	C6 H14 O12 P2	339.9962	1.181	338.98888	Full match	No results
Com_626_all	8-iso-15-keto Prostaglandin E2	C20 H30 O5	332.19883	8.031	331.19155	Invalid mass	No results
Com_627_all	N-Acetyl-Dl-glutamic acid	C7 H11 N O5	189.06357	1.479	188.05615	No results	No results
Com_628_all	19(R)-hydroxy Prostaglandin A2	C20 H30 O5	332.19895	6.702	331.19162	Invalid mass	No results
Com_629_all	LPC 20:2	C28 H54 N O7 P	593.36928	9.732	592.36201	No results	Invalid mass
Com_630_all	Elaidic acid	C18 H34 O2	282.2557	9.944	281.24842	Full match	No results
Com_631_all	LPE O-18:3	C23 H44 N O6 P	461.29088	9.489	460.28353	No results	Full match
Com_632_all	Adenosine diphosphate ribose	C15 H23 N5 O14 P2	559.07159	1.434	558.06431	Full match	No results
Com_633_all	D-2-Aminoadipic acid	C6 H11 N O4	161.06853	1.988	160.06126	No results	No results
Com_634_all	PC 8:0_16:0	C32 H64 N O8 P	667.4427	11.627	666.43542	No results	Invalid mass

Compound_ID	Name	Formula	Molecular Weight	RT [min]	m/z	mzCloud_Results	mzVault_Results
Com_635_all	MGDG O-24:4_16:0	C49 H88 O9	880.67152	10.489	879.66425	No results	Invalid mass
Com_636_all	10-Hydroxydecanoic acid	C10 H20 O3	188.14108	6.281	187.13381	Full match	Full match
Com_637_all	N-Acetylornithine	C7 H14 N2 O3	174.10014	1.486	173.09283	Full match	No results
Com_638_all	LPS 22:5	C28 H46 N O9 P	571.29034	8.928	570.28307	No results	Full match
Com_639_all	6-Ketoprostaglandin F1α	C20 H34 O6	352.22493	7.26	351.21765	Invalid mass	No results
Com_640_all	17-AAG	C31 H43 N3 O8	567.28108	5.906	566.27374	Invalid mass	No results
Com_641_all	Prostaglandin A1 ethyl ester	C22 H36 O4	346.25085	8.934	345.24358	Invalid mass	No results
Com_642_all	Dodecanedioic acid	C12 H22 O4	460.30349	6.452	229.14447	Invalid mass	No results
Com_643_all	LPE 20:1	C25 H50 N O7 P	507.3322	10.469	506.32492	No results	Full match
Com_644_all	Prostaglandin H2	C20 H32 O5	334.21448	6.378	333.20723	Invalid mass	No results
Com_645_all	Biopterin	C9 H11 N5 O3	237.08579	5.893	236.07851	No results	No results
Com_646_all	Glycerol-3-phosphate	C3 H9 O6 P	172.01355	1.258	171.00624	No results	No results
Com_647_all	Indole-3-acetic acid	C10 H9 N O2	175.0631	4.903	174.05582	Full match	No results
Com_648_all	4-Butylresorcinol	C10 H14 O2	166.09942	6.363	331.19163	No results	No results
Com_649_all	LPG 15:0	C21 H43 O9 P	470.26438	8.398	469.25711	No results	Full match
Com_650_all	LPG 13:0	C19 H39 O9 P	442.23298	7.646	441.22571	No results	Full match
Com_651_all	LPI 16:1	C25 H47 O12 P	570.28028	8.253	569.27301	No results	Full match
Com_652_all	LPC 17:2	C25 H48 N O7 P	551.32238	8.476	550.3151	No results	Invalid mass
Com_653_all	Delta-Tridecalactone	C13 H24 O2	212.17749	6.895	211.17018	No results	No results
Com_654_all	LNAPE 18:2/N-24:7	C47 H76 N O8 P	813.51572	11.09	812.50844	No results	Invalid mass
Com_655_all	1-[(1R,2S,3R,5R)-5-Cyclohexyl-2,3-dihydroxycyclopentyl]-3-ethylurea	C14 H26 N2 O3	316.19953	5.839	315.19225	Invalid mass	No results
Com_656_all	11-keto Testosterone (CRM)	C19 H26 O3	348.19366	6.53	347.18628	Invalid mass	No results
Com_657_all	LPI 18:3	C27 H47 O12 P	594.28056	8.273	593.27328	No results	Full match
Com_658_all	4-Acetamidobutyric Acid	C6 H11 N O3	145.07343	1.431	144.06615	No results	No results
Com_659_all	LNAPE 18:1/N-3:0	C26 H50 N O8 P	535.32753	9.635	534.32026	No results	Full match
Com_660_all	L-Arabinitol	C5 H12 O5	152.06816	1.598	151.06088	No results	No results
Com_661_all	D-Xylonic Acid	C5 H10 O6	166.04807	5.887	211.04632	No results	No results
Com_662_all	N-Acetyl-α-D-glucosamine 1-phosphate	C8 H16 N O9 P	265.07963	1.501	300.04886	Invalid mass	Invalid mass
Com_663_all	D-Erythrose 4-phosphate	C4 H9 O7 P	200.00843	1.215	199.00113	No results	No results
Com_664_all	2-Hydroxy-2-methylbutanedioic acid	C5 H8 O5	148.03697	1.264	184.98552	No results	No results
Com_665_all	LPE O-19:1	C24 H50 N O6 P	479.33733	11.05	478.33006	No results	Full match
Com_666_all	Methyl EudesMate	C11 H14 O5	226.08418	5.725	207.06631	No results	No results
Com_667_all	LNAPE 18:2/N-3:0	C26 H48 N O8 P	533.31142	9.127	532.30415	No results	Full match
Com_668_all	Thymopentin	C30 H49 N9 O9	679.3674	9.688	678.36021	No results	No results
Com_669_all	17(S)-HpDHA	C22 H32 O4	360.23011	7.188	359.22283	Full match	No results
Com_670_all	gamma-Glutamylmethionine	C10 H18 N2 O5 S	278.09383	4.882	277.08655	No results	Full match
Com_671_all	DL-4-Hydroxyphenyllactic acid	C9 H10 O4	182.05528	5.439	181.048	Invalid mass	No results
Com_672_all	(±)10(11)-EpDPA	C22 H32 O3	344.23496	9.054	343.22785	Full match	No results
Com_673_all	Lithocholic Acid	C24 H40 O3	422.30093	11.602	421.29365	Invalid mass	No results
Com_674_all	D-Ribulose 1,5-bisphosphate	C5 H12 O11 P2	309.98551	1.185	308.97823	No results	No results
Com_675_all	lipoamide	C8 H15 N O S2	205.0594	1.492	455.11855	No results	No results
Com_676_all	LPG 18:3	C24 H43 O9 P	506.2644	8.151	505.25713	No results	Full match
Com_677_all	N2-Methylguanosine	C11 H15 N5 O5	297.1074	4.847	296.10013	Full match	Full match
Com_678_all	Tridecylic acid	C13 H26 O2	214.19323	7.186	213.18596	Full match	No results
Com_679_all	Chlorogenic Acid Methyl Ester	C17 H20 O9	368.10951	1.295	367.10223	No results	No results
Com_680_all	Arachidic acid	C20 H40 O2	312.30314	11.642	311.29586	No results	No results
Com_681_all	LPS 22:6	C28 H44 N O9 P	569.27535	8.661	568.26807	No results	Full match
Com_682_all	LPS 20:5	C26 H42 N O9 P	543.25957	8.214	542.2523	No results	Full match
Com_683_all	LPS 18:3	C24 H42 N O9 P	519.25953	8.233	518.25225	No results	Full match
Com_684_all	Thymidine 5'-monophosphate	C10 H15 N2 O8 P	322.05646	2.325	321.04918	Full match	Full match
Com_685_all	Desthiobiotin	C10 H18 N2 O3	214.13167	3.931	213.1244	Full match	No results
Com_686_all	4-(octyloxy)benzoic acid	C15 H22 O3	250.15689	6.639	249.14962	Full match	No results
Com_687_all	FAHFA 18:0/3:0	C21 H40 O4	356.29259	11.436	355.28532	No results	Full match

Compound_ID	Name	Formula	Molecular Weight	RT [min]	m/z	mzCloud_Results	mzVault_Results
Com_688_all	LPE 20:0	C25 H52 N O7 P	509.34773	11.1	508.34046	No results	Full match
Com_689_all	LPG 20:3	C26 H47 O9 P	534.29574	9.292	533.28846	No results	Full match
Com_690_all	L-Ascorbate	C6 H8 O6	176.03198	1.451	211.00119	No results	No results
Com_691_all	alpha-Benzylsuccinic acid	C11 H12 O4	208.07321	5.821	207.06589	No results	No results
Com_692_all	Taurolithocholic acid	C26 H45 N O5 S	483.30166	7.583	482.29438	No results	No results
Com_693_all	(2S)-4-Oxo-2-phenyl-3,4-dihydro-2H-chromen-7-yl beta-D-glucopyranoside	C21 H22 O8	448.14152	6.684	447.13424	Invalid mass	No results
Com_694_all	LPE 15:0	C20 H42 N O7 P	439.26939	8.625	438.26212	No results	Full match
Com_695_all	5-Methyluridine	C10 H14 N2 O6	258.08529	4.778	257.07804	No results	No results
Com_696_all	3-Indoleacrylic acid	C11 H9 N O2	187.06329	4.813	186.05602	No results	No results
Com_697_all	2-Deoxyuridine	C9 H12 N2 O5	228.07469	4.77	227.06742	No results	No results
Com_698_all	UDP-galactose	C15 H24 N2 O17 P2	566.05444	1.253	565.04716	No results	No results
Com_699_all	Dl-Glyceraldehyde3-phosphate	C3 H7 O6 P	169.99886	5.391	168.99158	No results	No results
Com_700_all	2'-Deoxyinosine	C10 H12 N4 O4	252.08576	4.554	251.07848	Full match	Full match
Com_701_all	Adenylosuccinic acid	C14 H18 N5 O11 P	463.07395	4.624	462.06668	No results	Full match
Com_702_all	13Z,16Z-Docosadienoic Acid	C22 H40 O2	336.30318	9.562	335.2959	Full match	No results
Com_703_all	7-Hydroxy-3,4-dihydrocarbostyril	C9 H9 N O2	163.0631	5.963	162.0558	No results	No results
Com_704_all	Ribulose-5-phosphate	C5 H11 O8 P	230.01957	5.284	229.0123	No results	No results
Com_705_all	AICA ribonucleotide	C9 H15 N4 O8 P	338.06232	1.592	337.05554	Full match	No results
Com_706_all	LPS 22:4	C28 H48 N O9 P	573.30684	9.417	572.29956	No results	Full match
Com_707_all	N'1-[1-(2-hydroxyphenyl)ethylidene]-3-methoxybenzene-1-carbohydrazide	C16 H16 N2 O3	568.23556	9.715	283.1105	Invalid mass	No results
Com_708_all	23-Norcholic acid	C23 H38 O5	394.27214	7.483	393.26486	No results	No results
Com_709_all	NSI-189	C22 H30 N4 O	366.24342	9.28	365.23615	No results	No results
Com_710_all	L-Glutathione oxidized	C20 H32 N6 O12 S2	612.15206	2.397	611.14478	Full match	Full match
Com_711_all	Carnosine	C9 H14 N4 O3	226.10656	1.284	225.09928	Full match	Full match
Com_712_all	Inosine-5'-monophosphate (IMP)	C10 H13 N4 O8 P	348.04702	1.799	347.03975	Full match	Full match
Com_713_all	Pantothenic acid	C9 H17 N O5	219.11061	5.034	218.10333	Full match	Full match
Com_714_all	(+/-)12(13)-DiHOME	C18 H34 O4	314.24568	9.165	295.22782	Full match	No results
Com_715_all	Xanthine	C5 H4 N4 O2	152.03319	2.235	151.02591	Full match	No results
Com_716_all	Xanthosine	C10 H12 N4 O6	284.07585	5.231	283.06857	Full match	Full match
Com_717_all	Azelaic acid	C9 H16 O4	188.10475	5.793	187.09747	No results	No results
Com_718_all	all-cis-4,7,10,13,16-Docosapentaenoic acid	C22 H34 O2	330.25595	10.241	329.24867	Full match	No results
Com_719_all	8Z,11Z,14Z-Eicosatrienoic acid	C20 H34 O2	306.25586	10.449	305.24859	Full match	No results
Com_720_all	Eicosapentaenoic acid	C20 H30 O2	302.22456	9.527	301.21729	Full match	No results
Com_721_all	Hypoxanthine	C5 H4 N4 O	136.0383	2.053	135.03102	Full match	Full match
Com_722_all	Mevalonic acid	C6 H12 O4	148.07334	3.214	147.06606	Full match	No results
Com_723_all	Guanosine	C10 H13 N5 O5	283.09161	3.641	282.08434	Full match	Full match
Com_724_all	13(S)-HOTrE	C18 H30 O3	294.21966	7.909	293.21239	Full match	No results
Com_725_all	Lysops 22:5	C28 H46 N O9 P	571.28902	9.206	570.28175	No results	No results
Com_726_all	L-Glutamate	C5 H9 N O4	147.05292	1.478	146.04561	No results	No results
Com_727_all	Phenylacetylglycine	C10 H11 N O3	193.07378	5.471	192.06651	Full match	Full match
Com_728_all	Dibutyl sebacate	C18 H34 O4	314.24586	7.056	313.23859	No results	No results
Com_729_all	N-lactoyl-phenylalanine	C12 H15 N O4	237.10015	5.657	236.09287	No results	No results
Com_730_all	Citric acid	C6 H8 O7	192.02677	1.871	191.01949	No results	No results
Com_731_all	Glycocholic acid	C26 H43 N O6	465.30884	6.756	464.30156	Full match	Full match
Com_732_all	LPE 22:5	C27 H46 N O7 P	527.301	9.26	526.29373	No results	Full match
Com_733_all	Creatine	C4 H9 N3 O2	131.06919	1.391	130.06191	Full match	Full match
Com_734_all	Arachidonic acid	C20 H32 O2	304.24023	10.01	303.23295	Full match	No results
Com_735_all	H-Gly-Pro-OH	C7 H12 N2 O3	172.08454	1.429	217.08276	No results	No results
Com_736_all	(5-L-Glutamyl)-L-Amino Acid	C8 H14 N2 O5	218.09003	1.46	217.08275	No results	No results
Com_737_all	L-Argininosuccinate	C10 H18 N4 O6	290.12246	1.386	289.11526	No results	No results
Com_738_all	Uridine	C9 H12 N2 O6	244.06951	2.323	243.06223	Full match	Full match
Com_739_all	Reduced nicotinamide adenine dinucleotide	C21 H29 N7 O14 P2	665.12524	2.68	664.11797	No results	Full match
Com_740_all	LPC 22:5	C30 H52 N O7 P	615.35361	9.085	614.34633	No results	Invalid mass

Compound_ID	Name	Formula	Molecular Weight	RT [min]	m/z	mzCloud_Results	mzVault_Results
Com_741_all	8,15-Dihete	C20 H32 O4	336.22995	8.421	317.21213	No results	No results
Com_742_all	(R)-3-Hydroxy myristic acid	C14 H28 O3	244.20377	7.719	243.1965	Full match	No results
Com_743_all	LPC O-17:0	C25 H54 N O6 P	541.37437	10.314	540.3671	No results	Invalid mass
Com_744_all	DL-Citrulline	C6 H13 N3 O3	175.09538	1.556	220.09359	No results	No results
Com_745_all	Docosahexaenoic acid	C22 H32 O2	328.24022	9.939	327.23294	Full match	Full match
Com_746_all	N-acetyl-L-ornithine	C7 H14 N2 O3	174.10019	1.398	173.09285	No results	No results
Com_747_all	LPC 16:0	C24 H50 N O7 P	541.33816	9.455	540.33088	No results	Invalid mass
Com_748_all	Homo-Gamma-Linolenic Acid (C20:3)	C20 H34 O2	306.25572	10.824	305.24845	No results	No results
Com_749_all	Lipoic acid	C8 H14 O2 S2	206.04255	2.447	205.03526	Full match	No results
Com_750_all	Asp-Phe methyl ester	C14 H18 N2 O5	294.12169	4.976	293.11442	No results	No results
Com_751_all	N-Acetylneuraminic acid	C11 H19 N O9	309.10595	1.507	308.09867	No results	No results
Com_752_all	Lysopc 16:2 (2N Isomer)	C24 H46 N O7 P	491.30092	8.974	490.29365	No results	No results
Com_753_all	Adenosine 5'-monophosphate	C10 H14 N5 O7 P	347.06285	1.713	346.05558	Full match	Full match
Com_754_all	Histamine	C5 H9 N3	111.07937	1.366	110.0721	No results	Full match
Com_755_all	Stearic acid	C18 H36 O2	284.27152	11.379	283.26425	Full match	No results
Com_756_all	Dibenzoyl Thiamine	C26 H26 N4 O4 S	490.16877	1.342	489.16149	No results	No results
Com_757_all	LPC 22:4	C30 H54 N O7 P	617.36919	9.567	616.36191	No results	Invalid mass
Com_758_all	Prostaglandin E2	C20 H32 O5	352.22496	6.266	351.21771	No results	No results
Com_759_all	LPE 17:0	C22 H46 N O7 P	467.30088	9.843	466.29361	No results	Full match
Com_760_all	Purine	C5 H4 N4	120.04198	1.478	119.0347	Invalid mass	No results
Com_761_all	Hippuric acid	C9 H9 N O3	179.0581	5.36	178.05082	Full match	Full match
Com_762_all	LPE 18:0	C23 H48 N O7 P	481.31679	9.999	480.30933	No results	Full match
Com_763_all	Glycerophospho-N-palmitoyl ethanolamine	C21 H44 N O7 P	453.28553	9.408	452.27826	Full match	Full match
Com_764_all	L-lysine	C6 H14 N2 O2	146.10536	1.251	145.09808	No results	No results
Com_765_all	LPC 17:1	C25 H50 N O7 P	553.33806	9.248	552.33078	No results	Invalid mass
Com_766_all	L-Hydroxyproline	C5 H9 N O3	131.05803	2.481	130.05075	No results	No results
Com_767_all	Glycoursodeoxycholic acid	C26 H43 N O5	449.31411	7.445	448.30683	Full match	Full match
Com_768_all	Ala-trp	C14 H17 N3 O3	275.12715	5.286	274.11987	No results	No results
Com_769_all	Lysops 22:6	C28 H44 N O9 P	569.27292	8.822	568.26564	No results	No results
Com_770_all	LPC O-17:1	C25 H52 N O6 P	539.35865	10.288	538.35138	No results	Invalid mass
Com_771_all	LPE 22:6	C27 H44 N O7 P	525.28522	8.933	524.27808	No results	Full match
Com_772_all	Thymidine	C10 H14 N2 O5	242.09038	4.848	241.08311	Full match	No results
Com_773_all	Adenosine diphosphate (ADP)	C10 H15 N5 O10 P2	427.02936	1.47	426.02208	Full match	No results
Com_774_all	Indole-3-lactic acid	C11 H11 N O3	205.07374	5.592	204.06646	Full match	Full match
Com_775_all	Lysopc 18:1	C26 H52 N O7 P	521.34824	10.157	566.34637	No results	No results
Com_776_all	LPE 17:1	C22 H44 N O7 P	465.28539	9.211	464.27812	No results	Full match
Com_777_all	N-Acetyl-L-methionine	C7 H13 N O3 S	191.06154	5.268	190.05427	Full match	Full match
Com_778_all	5-Hydroxytryptophan	C11 H12 N2 O3	220.08472	4.741	219.07744	Full match	Full match
Com_779_all	15(S)-HpETE	C20 H32 O4	336.23012	7.507	335.22289	Full match	Full match
Com_780_all	1-Methyluric acid	C6 H6 N4 O3	182.04397	4.775	181.03669	No results	No results
Com_781_all	Isobutyryl carnitine	C11 H21 N O4	231.14695	5.008	276.14518	No results	No results
Com_782_all	L-Asparagine	C4 H8 N2 O3	132.05321	1.287	131.04592	No results	No results
Com_783_all	LysoPE 18:2	C23 H44 N O7 P	477.28564	8.587	476.27837	No results	No results
Com_784_all	D-Phenylalanine	C9 H11 N O2	165.07873	4.942	164.07146	No results	No results
Com_785_all	PC 9:0_9:0	C26 H52 N O8 P	583.34848	7.64	582.34139	No results	Invalid mass
Com_786_all	Prostaglandin E1	C20 H34 O5	354.24068	7.446	353.2334	No results	No results
Com_787_all	13-Hpotre(R)	C18 H30 O4	310.21457	7.158	309.20729	No results	No results
Com_788_all	N-Acetyl-L-tyrosine	C11 H13 N O4	223.0845	5.645	222.07722	Full match	Full match
Com_789_all	N4-Acetylcytidine	C11 H15 N3 O6	285.09615	4.878	284.08887	Full match	Full match
Com_790_all	Sebacic acid	C10 H18 O4	202.12044	5.999	201.11316	No results	No results
Com_791_all	12-oxo Phytodienoic Acid	C18 H28 O3	292.20387	7.369	291.19659	Full match	No results
Com_792_all	LPE 20:4	C25 H44 N O7 P	501.28572	9.013	500.27844	No results	Full match
Com_793_all	5-(tert-butyl)-2-methyl-N-(5-methyl-3-isoxazolyl)-3-furamide	C14 H18 N2 O3	262.13173	5.283	261.12446	Full match	No results

Compound_ID	Name	Formula	Molecular Weight	RT [min]	m/z	mzCloud_Results	mzVault_Results
Com_794_all	Punicic Acid	C18 H30 O2	278.22386	10.47	277.21695	No results	No results
Com_795_all	LPC 22:6	C30 H50 N O7 P	613.3379	8.801	612.33063	No results	Invalid mass
Com_796_all	LPC 14:0	C22 H46 N O7 P	513.30644	8.321	512.29917	No results	Invalid mass
Com_797_all	Maltotriose	C18 H32 O16	504.17117	9.149	503.16389	No results	No results
Com_798_all	LPC 15:0	C23 H48 N O7 P	527.32237	8.836	526.3151	No results	Invalid mass
Com_799_all	3-Oxo-7alpha,12alpha-hydroxy-5beta-cholanoic acid	C24 H38 O5	406.27195	6.527	405.26469	No results	No results
Com_800_all	Lysopc 17:0	C25 H52 N O7 P	509.34783	10.325	508.34055	No results	No results
Com_801_all	Lysope 18:1	C23 H46 N O7 P	479.30116	8.576	478.29391	No results	No results
Com_802_all	Quinoline-4-carboxylic acid	C10 H7 N O2	173.04747	5.638	172.0402	No results	No results
Com_803_all	Gamma-Glu-Leu	C11 H20 N2 O5	260.13718	5.5	259.12991	No results	No results
Com_804_all	dUMP	C9 H13 N2 O8 P	308.04081	2.777	307.03364	No results	No results
Com_805_all	Cyclic ADP-ribose	C15 H21 N5 O13 P2	541.06057	1.838	540.05329	Full match	No results
Com_806_all	2-deoxyglucose-6-phosphate	C6 H13 O8 P	244.03481	1.44	289.03302	No results	No results
Com_807_all	LPE 16:1	C21 H42 N O7 P	451.26986	9.155	450.26258	No results	Full match
Com_808_all	Tetranor-12R-HETE	C16 H26 O3	266.18823	7.371	265.18095	Full match	No results
Com_809_all	D-Glucosamine 6-phosphate	C6 H14 N O8 P	259.04591	1.251	258.03864	No results	No results
Com_810_all	mesaconic acid	C5 H6 O4	130.02639	1.466	129.01912	No results	No results
Com_811_all	4-Methyl-2-Oxopentanoic Acid	C6 H10 O3	130.06283	5.476	129.05555	No results	No results
Com_812_all	Pyridoxine	C8 H11 N O3	169.07373	5.002	168.06645	No results	No results
Com_813_all	3-methyl-5-oxo-5-(4-toluidino)pentanoic acid	C13 H17 N O3	235.12091	5.685	234.11363	Full match	No results
Com_814_all	D-Gluconic acid	C6 H12 O7	196.05852	1.888	195.05096	No results	No results
Com_815_all	Spiculisporic Acid	C17 H28 O6	328.18837	6.144	327.1811	No results	No results
Com_816_all	L-Tryptophan	C11 H12 N2 O2	204.08956	5.448	249.08751	No results	No results
Com_817_all	Asp-glu	C9 H14 N2 O7	262.0799	1.422	261.07261	No results	No results
Com_818_all	13,14-dihydro-15-keto-tetranor Prostaglandin E2	C16 H26 O5	280.17085	8.201	279.16357	Invalid mass	No results

MassList_Results	Class_I	Class_II	Class_III	Class_IV	all_YM1_1	all_YM1_2	all_YM1_3	all_YM1_4
Full match	Organic nitrogen compounds	Organonitrogen compounds	Quaternary ammonium salts	Carnitines	26288014488	27582192272	28277634076	27301948131
No results	Lipids and lipid-like molecules	Fatty Acyls	Fatty amides	Fatty amides	21929338227	29838280033	23780949031	26506758099
No results	Lipids and lipid-like molecules	Fatty Acyls	Fatty amides	Fatty amides	6591598311	6967269307	5548432392	6291254575
No results	Lipids and lipid-like molecules	Glycerolipids	Monoradylglycerols	1-monoacylglycerols	7482942759	5336646327	5952706593	6208002123
Full match	Organic acids and derivatives	Carboxylic acids and derivatives	Amino acids, peptides, and analogues	Phenylalanine and derivatives	5611922658	5166098037	6615915105	5514416425
No results	Lipids and lipid-like molecules	Fatty Acyls	Fatty amides	N-acyl amines	3247206414	4810649424	3100119875	4281978767
Full match	Benzenoids	Benzene and substituted derivatives	Phenethylamines	Phenethylamines	2603502.91	2928056.389	2331336.369	2218105.029
No results	Organic acids and derivatives	Carboximidic acids and derivatives	Carboximidic acids	Carboximidic acids	2770383709	3664328357	2587494763	3239913568
Full match	Lipids and lipid-like molecules	Glycerolipids	Monoradylglycerols	1-monoacylglycerols	72724686.6	65945381.7	142005710.1	110764411.2
No results	Lipids and lipid-like molecules	Fatty Acyls	Fatty acid esters	Acyl carnitines	1708231204	1797882913	1791878551	1341907563
Full match					71238367.95	67170190.07	80308411.13	80671076.64
No results					1310625142	898134057	1089185269	1199981168
Full match	Lipids and lipid-like molecules	Fatty Acyls	Fatty acid esters	Acyl carnitines	1474873993	980300487.9	1110365744	1116119734
No results					3038149051	2951947496	2975268914	1222758978
Full match					373805498.8	427309504.7	370979365.7	549942020.4
No results	Lipids and lipid-like molecules	Fatty Acyls	Fatty acids and conjugates	Long-chain fatty acids	55750944.47	74955368.46	73362473.27	86566618.28
No results	Organic acids and derivatives	Carboxylic acids and derivatives	Amino acids, peptides, and analogues	Leucine and derivatives	458812795.6	525463322.2	589212175.1	465032878.9
Full match	Lipids and lipid-like molecules	Fatty Acyls	Fatty acid esters	Acyl carnitines	2467891918	2175757982	2158698871	2438037104
No results	Phenylpropanoids and polyketides	Flavonoids	Flavans	Epigallocatechins	3873966549	3023710570	2507876187	2185201725
Full match	Lipids and lipid-like molecules	Fatty Acyls	Lineolic acids and derivatives	Lineolic acids and derivatives	157435.1865	162598.4532	166050.9435	159929.7217
Full match	Phenylpropanoids and polyketides	Cinnamic acids and derivatives	Hydroxycinnamic acids and derivatives	Hydroxycinnamic acids	615805629.2	560973826.3	631939047.2	590551863.6
No results					611728127.2	630924827.5	538846893.8	460149835
No results					342551070	341600628.9	381378730.5	408954761
No results	Lipids and lipid-like molecules	Glycerophospholipids	Glycerophosphocholines	Phosphocholines	1467829495	1329742611	1449176010	652231278.3
No results					126601790.2	197967003.1	208893999	209126736.2
No results	Organic nitrogen compounds	Organonitrogen compounds	Amines	1,3-aminoalcohols	409793173.4	354548638.3	470576241.3	323705671.1
No results					1421373725	1304221796	1372808099	841213682.6
Full match	Lipids and lipid-like molecules	Fatty Acyls	Fatty amides	Fatty amides	233241697.8	346578001.4	265490595.4	261134875.4
No results					236966247.9	204580395.7	209926069.6	216723301.5
Full match	Lipids and lipid-like molecules	Fatty Acyls	Fatty acids and conjugates	Very long-chain fatty acids	36613154.56	31104076.98	32384165.06	38057674.91
No results					290667146.2	272576738.4	293116433.3	222227803.1
No results					17733299.63	14654342.27	32808189.09	23003101.2
Full match	Organoheterocyclic compounds	Indoles and derivatives	Tryptamines and derivatives	Serotonins	75442685.47	72101328.37	71986391.37	78257735
Full match	Organic acids and derivatives	Carboxylic acids and derivatives	Amino acids, peptides, and analogues	Dipeptides	77653482.21	74614161.28	90089151.88	66005873.43
No results					265480927.8	194869754.8	202141227.5	228376035
Full match	Organic acids and derivatives	Carboxylic acids and derivatives	Amino acids, peptides, and analogues	Dipeptides	1141380661	2037800453	1323417662	1232302587
Full match					19626365.48	18461640.58	22557710.32	23732632
No results	Organic acids and derivatives	Carboxylic acids and derivatives	Amino acids, peptides, and analogues	L-alpha-amino acids	201853674.7	197694487.6	199424954.8	179307976.4
No results					352070761.4	353931560.7	333111104.3	113849059
No results					806185537.8	708436887	737536851.4	993783608.3
No results					224356144.2	242007144.3	221159957.6	166045002
No results					806185537.8	525628724.3	555653900.7	734960328.7
No results	Lipids and lipid-like molecules	Glycerophospholipids	Glycerophosphocholines	Phosphocholines	13738184.24	12746579.88	12235223.37	11694523.38
No results					13388503	11157412.84	14287356.3	18570360.23
No results	Lipids and lipid-like molecules	Fatty Acyls	Fatty acid esters	Acyl carnitines	139228261.5	131604019.6	134641532.7	86748649.11
No results					130572217.4	116374085.1	148053517.3	110691455
No results					56214666.45	50207228.34	61411604.77	66764197.41
No results	Lipids and lipid-like molecules	Glycerophospholipids	Glycerophosphocholines	Phosphocholines	151875096.9	127006798.8	147879013.3	83123656.99
No results					103707616.2	126867590.2	104481331.6	128919964.7
No results					100635123.5	86998231.66	81589242.12	90908964.87
No results	Lipids and lipid-like molecules	Sphingolipids	Phosphosphingolipids	Phosphosphingolipids	53062889.74	51284152.99	52245353.63	52439907.9

MassList_Results	Class_I	Class_II	Class_III	Class_IV	all_YM1_1	all_YM1_2	all_YM1_3	all_YM1_4
No results	Benzenoids	Phenanthrenes and derivatives	Not available	Phenanthrenes and derivatives	45801339.94	41562871.37	61821110.53	40001451.54
No results					33584861.11	21305653.93	20802726.74	32001570.02
No results					50829439.48	47247286.73	40759748.72	38442434.76
Full match					279196937.4	239604769.3	338525871.7	209539066.9
No results					2529792.742	1531357.397	1724612.483	2218248.748
No results	Alkaloids and derivatives	Tropane alkaloids	Not available	Tropane alkaloids	357693092.3	294601103.5	232168157.1	246540180.5
No match	Organoheterocyclic compounds	Benzothiazoles	Not available	Benzothiazoles	48117034.64	40458044.83	54756749.92	47380693.24
No results					61074200.89	44895942	45648051.87	55116597.9
No results	Organic nitrogen compounds	Organonitrogen compounds	Amines	N-acylethanolamines	53656048.58	49107397.42	55573039.42	47053245.03
Full match	Organic acids and derivatives	Carboxylic acids and derivatives	Amino acids, peptides, and analogues	Dipeptides	53767069.48	35166791.59	50258913.13	48900135.6
No results					32254315.05	27517585.01	37458759.52	42816702.34
Full match	Organoheterocyclic compounds	Indoles and derivatives	Indolyl carboxylic acids and derivatives	Indole-3-acetic acid derivatives	75851667.36	73494702.2	70838600.67	66323365.08
Full match					2647658.386	2200725.229	3891700.769	2835056.305
No results					92566899.96	68670032.01	69704007.53	98435909.86
No results	Organic nitrogen compounds	Organonitrogen compounds	Amines	N-acylethanolamines	7993701.317	9551490.947	13129936.72	13605492.74
No results					385830.4906	373089.3474	356444.4546	332195.7734
No results	Lipids and lipid-like molecules	Fatty Acyls	Fatty acids and conjugates	Long-chain fatty acids	10443424.67	5886261.455	6226166.677	4677738.268
No results					34087847.33	28802387.54	39434657.98	31418035.52
Full match	Lipids and lipid-like molecules	Fatty Acyls	Eicosanoids	Prostaglandins and related compounds	12653906.77	12047671.73	18532204.47	13893210
No results					18960965.53	18941188.19	23248067.04	22390044.91
No results	Lipids and lipid-like molecules	Fatty Acyls	Lineolic acids and derivatives	Lineolic acids and derivatives	60399292.14	61068820.37	48967096.84	4961848.42
No results					86759431.07	60241117.89	151537880.2	32614939.17
No results					22301614.98	17628533.25	41214073.94	23003101.2
No results					59406059.82	52472705.92	56994851.43	59238150.59
No results					238581786.5	191115778.2	265785051	428093520.4
No results					64893645.7	72685268.39	140749352	39484010.22
Full match					10560923.88	8345327.147	10057715.75	3671580.017
Full match	Organic nitrogen compounds	Organonitrogen compounds	Amines	1,2-aminoalcohols	32671766.21	27274587.88	32215040.39	32429017.12
Full match	Organic acids and derivatives	Carboxylic acids and derivatives	Amino acids, peptides, and analogues	Histidine and derivatives	75521221.02	59612782.62	68014790.3	63072470.97
No results					43636640.26	46979170.31	38165219.98	36284971.55
No results					58191627.61	56826323.7	84422588.59	55586462.63
No results					91159112.79	89113769.24	88198951.5	58452815.38
Full match	Nucleosides, nucleotides, and analogues	Pyrimidine nucleosides	Not available	Pyrimidine nucleosides	14281261.8	14584903.54	14048669.34	13806327.57
No results					47201476.54	43910989.9	45284429.29	36986259.11
Full match	Nucleosides, nucleotides, and analogues	5'-deoxyribonucleosides	5'-deoxy-5'-thionucleosides	5'-deoxy-5'-thionucleosides	28181992.92	31252450.71	26240469.54	21894478.57
Full match	Organoheterocyclic compounds	Imidazopyrimidines	Purines and purine derivatives	Purines and purine derivatives	36647373.65	38963171.5	44873860	34033273.32
No results	Benzenoids	Benzene and substituted derivatives	Phenylpropanes	Phenylpropanes	45885264.01	60721419.75	45166857.16	52109384.69
No results					378309.4367	443732.2029	657016.8875	388829.3092
No results					4893592.319	4300163.255	5133361.134	5116040.726
No results	Lipids and lipid-like molecules	Endocannabinoids	Not available	Endocannabinoids	107405187.7	98454205.53	74483351.98	77940437.1
Full match	Benzenoids	Benzene and substituted derivatives	Styrenes	Styrenes	55352705.35	51877885.67	49328718.97	51188338.46
No results					29683085.73	28265466.81	37349909.87	26232773.11
No results					258751867.9	240002419.1	238463762.1	232503693
No results					26671216.1	25096715.45	28456144.94	18628641.59
Full match	Organic acids and derivatives	Carboxylic acids and derivatives	Amino acids, peptides, and analogues	L-alpha-amino acids	3344946.466	162559402.7	157989628.2	154287411.8
No results					13750615.69	11963056.09	18595413.26	19115025.24
No results					15988327.68	14710561.92	15599936.74	15668373.97
Full match	Organic acids and derivatives	Carboxylic acids and derivatives	Amino acids, peptides, and analogues	Isoleucine and derivatives	105789378.9	149852019.7	50294998.56	121105836.8
No results	Organoheterocyclic compounds	Lactones	Gamma butyrolactones	Gamma butyrolactones	64242424.3	63085792.19	64522872.99	61508393.8
No results					52835679.05	52697387.36	52129913.47	49454578.32
Full match	Nucleosides, nucleotides, and analogues	(5'->5')-dinucleotides	Not available	(5'->5')-dinucleotides	21259333.86	125259036.8	33198902.62	76078938.14
No results					13153207.99	15651494.32	18108991.2	13895737.93
No match	Lipids and lipid-like molecules	Fatty Acyls	Fatty acids and conjugates	Long-chain fatty acids	12857619.43	15206300.86	15149576.84	14016442.08

MassList_Results	Class_I	Class_II	Class_III	Class_IV	all_YM1_1	all_YM1_2	all_YM1_3	all_YM1_4
No results					6056740.12	2040998.568	3162579.909	4734578.389
Full match					958266.7363	969601.017	1004261.738	1948422.091
No results					1599312.697	1901296.311	1517125.777	1655231.453
No results					2564956.576	2397303.466	3128387.841	2914747.245
No results	Lipids and lipid-like molecules	Glycerophospholipids	Glycerophosphocholines	Phosphocholines	30965853.02	27913877.92	27727780.22	15044865.14
No results					32724805.52	23989838.72	25438129.53	30663160.24
No results					5987936.009	5234700.128	6067589.94	2208278.802
Full match	Phenylpropanoids and polyketides	Coumarins and derivatives	Not available	Coumarins and derivatives	176784606.4	158012483.4	127915314.4	94035573.72
No results					15265820.69	7521824.284	9987985.55	10507759.86
No results					49630214.24	48573117.63	46384111.85	43610248.5
Full match	Organoheterocyclic compounds	Lactones	Gamma butyrolactones	Gamma butyrolactones	37759156.53	35613665.1	34454170.11	39849876.91
Full match	Nucleosides, nucleotides, and analogues	Purine nucleotides	Purine ribonucleotides	Purine ribonucleoside monophosphates	132303519	139739935.7	120210071.5	117646736.8
No results					32797850.7	22860369.45	33143334.96	12491022.04
Full match	Organic acids and derivatives	Carboxylic acids and derivatives	Amino acids, peptides, and analogues	Methionine and derivatives	15838063.45	18090164.24	22072321.25	17839450.76
Full match	Organic acids and derivatives	Carboxylic acids and derivatives	Amino acids, peptides, and analogues	Arginine and derivatives	51821206.2	43648724.62	54271139.02	51550985.66
Full match	Organoheterocyclic compounds	Pyridines and derivatives	Pyridinecarboxylic acids and derivatives	Nicotinamides	86458732.61	116941758.4	127180239.6	119933176
No results					39494954.12	44043814.24	41812970.24	39601658.97
No results					19950517.3	19653574.6	18845434.76	9999700.093
No results					524507.1121	508810.3231	508976.1308	385736.64
No results	Lipids and lipid-like molecules	Fatty Acyls	Fatty acid esters	Fatty acid esters	2025504.247	3174334.679	2609690.722	1774881.244
No results					24133222.15	22644941.31	26350494.8	30628921.22
No results					3198184.902	3057559.565	3503058.431	3353404.09
No results	Lipids and lipid-like molecules	Steroids and steroid derivatives	Vitamin D and derivatives	Vitamin D and derivatives	934878.3907	931648.8956	1025456.181	2059889.42
No results					167312614.7	146855891	151276330.9	147373897.8
No results	Lipids and lipid-like molecules	Fatty Acyls	Eicosanoids	Prostaglandins and related compounds	126630.1376	120296.9143	213378.1483	116378.2684
No results					59887110.85	23694033.07	61837193.7	55075267.87
Full match	Organic acids and derivatives	Carboxylic acids and derivatives	Amino acids, peptides, and analogues	Alpha amino acids	70526874.24	78187509.41	63272654.88	72881448.11
No results	Lipids and lipid-like molecules	Steroids and steroid derivatives	Androstane steroids	Androgens and derivatives	25898485.37	15260732.41	31563417.16	11230414.9
No results					85636107.2	88187913.88	95201022.4	76030434.78
No results	Lipids and lipid-like molecules	Steroids and steroid derivatives	Estrane steroids	Estrogens and derivatives	2745213.056	2341176.897	2491798.603	2204924.371
No results					22955127.5	18025780.31	19565029.31	29175581.58
No results					132403131.3	134079447.3	146371193.9	144764886.9
No results	Lipids and lipid-like molecules	Sphingolipids	Ceramides	Ceramides	13288804.24	7324867.98	11816757.33	11481165.1
Full match					154255247.1	153889296.2	153021423.7	151083038.3
Full match	Organic acids and derivatives	Carboxylic acids and derivatives	Amino acids, peptides, and analogues	Dipeptides	1832881.99	1655267.846	1930756.584	1687123.489
No results					2966563.545	1463914.488	1774687.16	1528441.984
No results					20300082.68	22289978.35	21954233.72	11536858.29
No results	Lipids and lipid-like molecules	Fatty Acyls	Fatty amides	N-acyl amines	1878696.203	2315059.515	1914870.423	1994279.921
No results					1325929.772	1221389.049	1364927.736	1100401.677
No results					14431226.54	9914905.771	10390553.66	13850133.58
No results	Lipids and lipid-like molecules	Fatty Acyls	Eicosanoids	Hydroxyeicosatetraenoic acids	5126797.401	1708966.315	2656137.313	2042736.739
No results					345660.4855	357689.4051	397544.5674	823630.1471
No results	Organic nitrogen compounds	Organonitrogen compounds	Amines	Aralkylamines	27770552.68	24196683.23	28864592.52	27607883.94
No results					24477459.38	19051878.73	21305634.28	24269289.72
Full match	Organic acids and derivatives	Carboxylic acids and derivatives	Amino acids, peptides, and analogues	Alpha amino acids and derivatives	151683701.3	274148653.4	273444508.9	270875026.4
No results					12778569.91	14237215	14500517.15	13045485.95
No results	Lipids and lipid-like molecules	Fatty Acyls	Lineolic acids and derivatives	Lineolic acids and derivatives	9456412.741	6581748.568	5297752.953	15365537.1
Full match	Organic acids and derivatives	Carboxylic acids and derivatives	Amino acids, peptides, and analogues	Dipeptides	15762009.47	13206960.01	13987965.38	14251407.72
No results					18804602.76	19680552.67	17651635.37	14871717.98
No results					3032234.596	2911196.365	3464779.327	7698687.263
No results					31250778.21	26776491.67	29739270.2	24970831.8
No results	Lipids and lipid-like molecules	Glycerolipids	Monoradylglycerols	2-monoacylglycerols	379813.9619	236556.2303	934413.7593	652184.3645
Full match	Organoheterocyclic compounds	Pyridines and derivatives	Pyridine carboxaldehydes	Pyridoxals and derivatives	35264396.59	38123367.56	37282404.62	30649315.04

MassList_Results	Class_I	Class_II	Class_III	Class_IV	all_YM1_1	all_YM1_2	all_YM1_3	all_YM1_4
Full match	Organic acids and derivatives	Carboxylic acids and derivatives	Amino acids, peptides, and analogues	N-acyl-alpha amino acids	7850559.052	9508658.78	9661898.231	8788220.749
No results	Organic acids and derivatives	Carboxylic acids and derivatives	Amino acids, peptides, and analogues	N-acyl-alpha amino acids	3419399.534	6059635.719	5204852.056	6976019.492
No results					38953736.77	24311632.21	17307056.51	12197359.44
No results					18432944.1	10202309.52	12367338.74	12136558.57
Full match	Organoheterocyclic compounds	Diazines	Pyrimidines and pyrimidine derivatives	Hydroxypyrimidines	18019253.06	17720232.86	15965463.06	16414915.24
Full match	Organic acids and derivatives	Carboxylic acids and derivatives	Amino acids, peptides, and analogues	Histidine and derivatives	12434157.62	12585322.17	13055314.25	14665241.97
No results					124469623.5	38760529.44	42108179.61	39533832.9
No results	Lipids and lipid-like molecules	Fatty Acyls	Eicosanoids	Prostaglandins and related compounds	272068.4012	261844.83	273942.9035	265416.8372
Full match	Organic nitrogen compounds	Organonitrogen compounds	Quaternary ammonium salts	Carnitines	3280308.045	3643385.393	3501959.744	3788489.951
Full match					1340107.661	239528.5778	1345596.63	1395082.337
No results	Lipids and lipid-like molecules	Sphingolipids	Phosphosphingolipids	Phosphosphingolipids	19128416.48	17734678.4	23738673.47	18839480.09
No results					2776895.833	3295210.357	2374841.08	2733916.673
No results					333989.1629	490326.9829	355830.3049	307431.5829
No results					28666273.76	26210269.27	26484498.19	23009333.4
Full match	Organic nitrogen compounds	Organonitrogen compounds	Amines	Dialkylamines	88798702.27	106858570.7	132922088.5	84556567.32
Full match	Lipids and lipid-like molecules	Glycerophospholipids	Glycerophosphocholines	Phosphocholines	6595925.418	6622563.314	17210656.53	6877683.646
No results					6186005.975	6655961.874	9530665.781	6717295.247
No results					15066576.62	13105895.58	10234044.94	17419395.21
No results					887457.0818	1016785.059	834745.6839	970408.0746
Full match					71392458.9	71833840.03	50372843.14	59445300.07
No results					16428795.2	16579488.43	14102374.7	12860105.58
No match					5217687.265	5618086.306	5783793.247	5904127.926
No results					1174152.98	723145.7058	5587560.805	6930555.083
Full match	Organic acids and derivatives	Carboxylic acids and derivatives	Amino acids, peptides, and analogues	L-alpha-amino acids	14108855.8	10677290.6	12387165.86	14092818.16
No results	Lipids and lipid-like molecules	Endocannabinoids	Not available	Endocannabinoids	12307735.68	7400172.721	10421660.15	8536480.84
No results					8218461.724	4921132.644	8828275.526	7359461.226
No results	Lipids and lipid-like molecules	Fatty Acyls	Eicosanoids	Prostaglandins and related compounds	3230786.86	3865449.222	5591251.314	4932956.69
No results					3650751.252	4558649.689	2071944.439	2263247.071
Full match	Lipids and lipid-like molecules	Glycerophospholipids	Glycerophosphoethanolamines	1-acyl-sn-glycero-3-phosphoethanolamines	794575.0103	559595.1055	511992.4613	680277.4468
No results					445079.8685	157000.6866	1716734.976	372658.6274
No results					623857.5554	171201.0658	242943.2009	172322.2373
No results	Lipids and lipid-like molecules	Glycerophospholipids	Glycerophosphocholines	Phosphocholines	3050089.535	2632927.912	2249536.742	2029332.249
No results					1901333.838	1777773.095	2252399.529	2082600.636
Full match					1803824.147	2638946.165	1859153.273	1841819.405
No results					10577171.01	9947552.85	8067296.415	9717500.446
No results	Benzenoids	Phenanthrenes and derivatives	Not available	Phenanthrenes and derivatives	13827168	7811182.993	7710227.22	9124915.139
No results					10009142.45	5824356.382	6242491.523	8586753.897
Full match	Lipids and lipid-like molecules	Glycerophospholipids	Glycerophosphocholines	1-acyl-sn-glycero-3-phosphocholines	11946742.9	11077589.35	14145058.52	11366129.31
No results	Lipids and lipid-like molecules	Prenol lipids	Monoterpenoids	Aromatic monoterpenoids	1546926.057	1313431.766	1287725.78	1289268.423
No results	Nucleosides, nucleotides, and analogues	5'-deoxyribonucleosides	5'-deoxy-5'-thionucleosides	5'-deoxy-5'-thionucleosides	17732511.2	21043039	24013899.87	14917741.94
No results	Organoheterocyclic compounds	Benzimidazoles	Not available	Benzimidazoles	1866563.284	1253399.811	8730256.115	2056712.762
Full match	Lipids and lipid-like molecules	Prenol lipids	Retinoids	Retinoids	866812.1357	620362.5787	559013.4238	645171.1089
No results					13488439.78	23995482.78	25375259.05	15041964.04
No results					44021942.71	44441218.07	42675557.79	43135141.57
No match					10897229.32	3991910.531	6496826.459	4687346.091
No results					1108213.322	1086915.677	1039947.675	1079787.003
No results					26390070.76	20376772.18	22352549.84	103192602.7
Full match	Nucleosides, nucleotides, and analogues	Purine nucleosides	Not available	Purine nucleosides	2236105.468	2837328.193	2982528.958	2165330.955
Full match	Benzenoids	Benzene and substituted derivatives	Aniline and substituted anilines	Aniline and substituted anilines	2040960.114	2386421.974	2886400.736	1949354.838
No results	Lipids and lipid-like molecules	Fatty Acyls	Fatty amides	Fatty amides	1973488.706	1306764.91	2005078.688	2274393.639
Full match	Benzenoids	Benzene and substituted derivatives	Benzoic acids and derivatives	Hydroxybenzoic acid derivatives	6148017.937	1141226.005	7992250.02	7496910.537
No results					3910215.91	2616482.694	3561632.538	2957555.368
Full match	Benzenoids	Benzene and substituted derivatives	Benzenesulfonic acids and derivatives	Benzenesulfonic acids and derivatives	4255810.785	5436744.06	3673685.92	2888225.05

MassList_Results	Class_I	Class_II	Class_III	Class_IV	all_YM1_1	all_YM1_2	all_YM1_3	all_YM1_4
Full match	Organoheterocyclic compounds	Imidazopyrimidines	Purines and purine derivatives	Xanthines	884755.0085	785056.3434	703100.9027	783395.4387
No results	Lipids and lipid-like molecules	Glycerophospholipids	Glycerophosphocholines	Phosphocholines	443402.5349	582777.3494	394142.684	501090.877
No results					177073.7057	481970.664	700830.2135	702049.245
Full match	Organic acids and derivatives	Carboxylic acids and derivatives	Amino acids, peptides, and analogues	Glutamic acid and derivatives	4311401.705	3466007.959	4198523.44	4422795.628
Full match					481090.0902	771316.3176	685122.468	718872.3239
Full match	Lipids and lipid-like molecules	Glycerophospholipids	Glycerophosphocholines	1-acyl-sn-glycero-3-phosphocholines	6554451.362	5659741.677	7511926.056	5046084.209
Full match					567972.8303	380138.7832	311295.3164	422561.325
Full match	Organoheterocyclic compounds	Imidazopyrimidines	Purines and purine derivatives	6-oxopurines	1613032.687	1273547.304	1200836.623	1243787.526
Full match	Lipids and lipid-like molecules	Glycerolipids	Monoradylglycerols	Monoacylglycerols	1003974.663	789376.4278	1330985.467	706435.6043
No results					93886.28388	100437.2293	102094.6111	90027.16253
No results					1432715.943	1102130.59	1416404.553	2194706.251
No results	Lipids and lipid-like molecules	Prenol lipids	Quinone and hydroquinone lipids	Ubiquinones	6122942.023	6191645.672	5938765.055	6057588.914
No results					698514.5993	1124733.609	2340003.834	1565159.78
No results					147187.4405	163292.7434	138850.3082	320138.6204
Full match					849191.4386	912617.8434	917935.4812	789197.6132
No results					1022114.161	1774117.467	1987749.734	512445.5605
No results	Organic acids and derivatives	Carboxylic acids and derivatives	Amino acids, peptides, and analogues	Alpha amino acids	8995580.69	9492907.171	8966488.218	60391165.28
No results					1301689.656	1298702.157	1446294.946	1855247.181
No results	Lipids and lipid-like molecules	Glycerophospholipids	Glycerophosphocholines	Phosphocholines	216140.8494	239791.0592	203897.992	231511.5601
No results					1891288.317	2012647.563	2074883.19	2555118.037
No results	Lipids and lipid-like molecules	Fatty Acyls	Fatty acids and conjugates	Long-chain fatty acids	9129731.583	6642596.642	9991487.602	7905197.353
No results	Organoheterocyclic compounds	Indoles and derivatives	N-alkylindoles	N-alkylindoles	1822322.911	2968698.256	1796159.528	2587847.291
Full match	Organoheterocyclic compounds	Indoles and derivatives	Hydroxyindoles	Hydroxyindoles	7967274.111	9340120.501	8555876.301	9787997.957
No results					2677947.408	2616036.242	2710344.817	2634460.144
No results					2988156.183	3202056.219	2732835.294	2358274.542
No results					14325888.03	16046220.86	13636382.91	9680131.578
No results					11088617.91	9840508.73	10050513.93	12655831.77
Full match					1520545.136	1086950.558	1468585.93	1222971.735
No results	Lipids and lipid-like molecules	Fatty Acyls	Eicosanoids	Prostaglandins and related compounds	4445439.972	3844074.821	4350953.046	3501079.393
No results	Organoheterocyclic compounds	Quinolines and derivatives	Quinoline carboxylic acids	Quinoline carboxylic acids	904141.0384	683313.911	612282.4675	640083.0191
No results					1408572.897	1916216.794	3124295.216	1527084.605
Full match	Nucleosides, nucleotides, and analogues	Purine nucleosides	Not available	Purine nucleosides	4885282.071	2379461.422	4295329.257	4397455.109
No results	Lipids and lipid-like molecules	Steroids and steroid derivatives	Androstane steroids	Androgens and derivatives	145361.2097	149727.0338	144111.1585	129940.0912
Full match	Organic oxygen compounds	Organooxygen compounds	Carbohydrates and carbohydrate conjugates	Hexoses	6499516.146	6681945.93	8006273.947	5984579.096
Full match	Lipids and lipid-like molecules	Prenol lipids	Retinoids	Retinoids	2486321.063	1299220.973	2543553.242	2412515.571
No results					786601.8806	1350756.449	1245445.469	1341765.983
No match	Lipids and lipid-like molecules	Fatty Acyls	Lineolic acids and derivatives	Lineolic acids and derivatives	1905843.173	1091969.243	1046969.218	1429374.252
Full match	Lipids and lipid-like molecules	Fatty Acyls	Fatty acid esters	Acyl carnitines	27795739.25	46494946.48	49054978.95	41095954.82
Full match	Organic acids and derivatives	Keto acids and derivatives	Short-chain keto acids and derivatives	Short-chain keto acids and derivatives	5239019.512	3864799.059	3980984.6	5252774.228
Full match					973938.4963	1021869.592	811830.3487	773488.5764
No results	Organic oxygen compounds	Organooxygen compounds	Carbonyl compounds	Cyclic ketones	3372223.487	2133767.112	2452324.228	3164573.033
Full match					5477304.27	4719403.501	5869726.586	4734764.036
No results	Lipids and lipid-like molecules	Prenol lipids	Triterpenoids	Limonoids	4060568.632	3140439.345	3047017.082	2612133.746
No results					1315114.215	660902.7724	799762.6654	1149502.997
No results					32678697.36	32235712.08	28501146.76	26881399.27
No results					1388181.259	1416920.361	1396731.066	1305623.791
No results					4789541.365	4502417.647	6908788.899	5060806.422
No results					1041122.039	967279.6706	1212189.599	1046074.453
No results					3403872.196	3055600.127	3315221.569	4239696.486
No results					1823552.683	1715942.621	1624091.359	2695450.298
Full match	Nucleosides, nucleotides, and analogues	Pyrimidine nucleotides	Pyrimidine ribonucleotides	Pyrimidine ribonucleoside diphosphates	8979574.662	9005977.322	8951742.771	9090953.2
Full match	Lipids and lipid-like molecules	Glycerophospholipids	Glycerophosphocholines	Phosphocholines	2589209.431	1652455.809	3374923.029	6963155.442
No results	Lipids and lipid-like molecules	Glycerophospholipids	Glycerophosphocholines	Phosphocholines	301833.8159	310285.6815	296609.7343	323723.0147

MassList_Results	Class_I	Class_II	Class_III	Class_IV	all_YM1_1	all_YM1_2	all_YM1_3	all_YM1_4
No results					26303302.55	27851133.42	18810665.35	19294605.2
No results					10715373.71	9724957.495	9976176.932	10980458.75
No results					142415.0539	185382.488	145112.4341	142383.8027
No results					6884311.594	7513231.747	7419440.918	4825600.528
No match					19347330.33	19727129.83	17620753.66	16976596.55
Full match	Organic acids and derivatives	Carboxylic acids and derivatives	Amino acids, peptides, and analogues	Proline and derivatives	4106181.31	1266417.362	3420302.006	3136253.419
No results					777536.2564	1161567.375	1337867.317	1191043.761
Full match	Lipids and lipid-like molecules	Steroids and steroid derivatives	Androstane steroids	Androgens and derivatives	2803177.582	1028972.96	1724209.675	644162.4193
No results					9605790.101	4716262.741	8680230.207	6947800.375
No results					1185053.164	1282904.565	1629005.889	1142310.062
No results					1205917.53	1046147.158	1230011.736	1316683.272
No results					856661.0927	4649675.466	1348936.152	1551127.807
No results					24518493.43	22048061.77	23797359.04	22435633.76
No results					3751020.857	2387753.581	4477080.567	3816258.947
No results					2526129.134	2297824.969	2640367.395	2240543.306
No results					5294433.881	4504910.085	5585324.543	3937813.643
No results					1608697.742	1577781.786	1509602.387	1676146.997
No results	Lipids and lipid-like molecules	Sphingolipids	Ceramides	Ceramides	4833756.84	4808996.377	5381313.783	4554996.344
No results	Lipids and lipid-like molecules	Glycerophospholipids	Glycerophosphocholines	Phosphocholines	1844743.816	1885538.966	1622802.277	1626662.868
Full match	Organic acids and derivatives	Carboxylic acids and derivatives	Amino acids, peptides, and analogues	Aspartic acid and derivatives	927184.1167	1212631.974	1283706.975	803461.4909
No results					4694366.909	2856040.107	3407511.125	2814489.07
No results					3833230.346	3417026.58	3895111.481	3402274.291
No results					14845126.15	13588620.06	11717175.28	10436655.95
Full match	Organic acids and derivatives	Carboxylic acids and derivatives	Amino acids, peptides, and analogues	D-alpha-amino acids	20700883.03	22541546.16	20333177.83	20136461.73
No results	Lipids and lipid-like molecules	Glycerophospholipids	Glycerophosphocholines	Phosphocholines	166558.9127	176541.4844	163074.7722	168432.8838
Full match	Lipids and lipid-like molecules	Steroids and steroid derivatives	Pregnane steroids	Gluco/mineralocorticoids, progestogins and derivatives	739753.1749	8060994.006	5260635.206	881490.3643
No results	Lipids and lipid-like molecules	Glycerophospholipids	Glycerophosphocholines	Phosphocholines	637673.7203	958523.1987	1016593.992	1800411.761
Full match					5486521.901	5006769.623	4581427.132	4167012.095
Full match	Lipids and lipid-like molecules	Glycerophospholipids	Glycerophosphates	1-acylglycerol-3-phosphates	896638.4927	934067.6694	810866.2275	855660.2083
No results	Benzenoids	Phenol ethers	Anisoles	Anisoles	4789597.025	2662720.446	8337202.034	1838643.369
No results	Lipids and lipid-like molecules	Fatty Acyls	Fatty acid esters	Fatty acid methyl esters	126330.5378	120604.4869	120846.1271	121367.0833
No results					1800081.33	1466707.921	1644306.104	2700268.669
No results					4043200.662	3623613.5	3831090.141	3902251.915
No results					313154.1395	451069.3025	262886.755	513505.2121
Full match	Organic acids and derivatives	Carboxylic acids and derivatives	Amino acids, peptides, and analogues	Oligopeptides	610296.3665	405489.9966	504983.294	445133.9712
No results					8933574.412	8364925.017	7439359.222	5112471.04
No results					1878244.908	3344872.236	3023482.502	2919873.341
No results					45012375	44911337.69	42112294.06	42455286.62
No results					40575214.22	40579956.05	18774619.08	19234723.91
Full match	Phenylpropanoids and polyketides	Cinnamic acids and derivatives	Cinnamic acids	Cinnamic acids	2344312.702	1702623.307	1867385.575	1743204.917
No results	Lipids and lipid-like molecules	Steroids and steroid derivatives	Estrane steroids	Estrogens and derivatives	188535.4205	177780.0406	190277.0525	209040.9948
Full match	Lipids and lipid-like molecules	Fatty Acyls	Fatty amides	N-acyl amines	2897087.842	3579822.967	3865707.05	2396257.055
Full match	Lipids and lipid-like molecules	Fatty Acyls	Eicosanoids	Hydroxyeicosatetraenoic acids	1533367.388	830684.5495	1207266.653	930548.0857
Full match					1292365.794	2334411.45	2164879.116	2497764.175
Full match	Organic acids and derivatives	Carboxylic acids and derivatives	Amino acids, peptides, and analogues	Dipeptides	963895.6946	954638.0922	1277136.374	1084590.864
No results					768913.1602	952797.9631	846639.0251	962989.9218
No results					3173742.944	3632934.152	3693313.548	1874775.303
No results					610096.8162	688843.302	588609.7339	937646.1027
No results	Phenylpropanoids and polyketides	Macrolides and analogues	Milbemycins	Milbemycins	767354.2567	752607.2414	720085.4376	747671.1718
No results	Phenylpropanoids and polyketides	Isoflavonoids	Isoflavonoid O-glycosides	Isoflavonoid O-glycosides	1489194.991	773179.8455	970852.8342	783461.9621
No results					16717185.16	14398738.09	14707842.06	15374742.72
No results	Lipids and lipid-like molecules	Glycerophospholipids	Glycerophosphocholines	Phosphocholines	1072845.028	1040490.966	10565296.92	828541.4007
No results					671176.9536	474965.0011	538105.5097	581648.1034

MassList_Results	Class_I	Class_II	Class_III	Class_IV	all_YM1_1	all_YM1_2	all_YM1_3	all_YM1_4
No results					34607710.33	34096832.61	22219512.16	35848828.46
No results					311638.742	448886.514	261614.6085	271478.3596
Full match	Lipids and lipid-like molecules	Glycerophospholipids	Glycerophosphocholines	Glycerophosphocholines	10785024.14	10943528.87	9776558.497	9519751.601
No results					8642567.02	3585351.785	8241125.661	7839059.216
No match					648208.4686	800088.854	464327.4536	266887.7754
Full match	Lipids and lipid-like molecules	Steroids and steroid derivatives	Bile acids, alcohols and derivatives	Glycinated bile acids and derivatives	2483821.449	1561321.362	1565953.941	3155309.776
No results					5336206.493	1170173.512	1391142.268	5555111.458
No results					1655375.967	304776.0075	330211.5649	301584.7897
No results					1503909.093	1304944.165	1383839.596	1123017.374
No results					5196381.07	5421634.988	4580246.822	4350724.806
Full match					2716289.078	2397756.347	2332019.314	2352698.686
No results					715056.5293	881958.1556	672883.328	989624.4933
No results					4452509.296	3397769.177	3836341.713	2697000.189
No results					1552507.087	587668.4172	1558495.01	1606137.046
Full match	Organic acids and derivatives	Carboxylic acids and derivatives	Amino acids, peptides, and analogues	Beta amino acids and derivatives	788293.6665	688990.9687	718823.1963	751411.3747
No results					934292.1554	594462.6631	934029.9573	727416.0403
Full match	Organic acids and derivatives	Carboxylic acids and derivatives	Amino acids, peptides, and analogues	Phenylalanine and derivatives	1673756.8	1774919.903	1813552.643	1580397.593
No results					646581.3783	303163.6076	546397.5275	298937.6803
No results	Lipids and lipid-like molecules	Glycerophospholipids	Glycerophosphocholines	Phosphocholines	245068.8135	258510.0415	256903.6144	236899.9357
Full match	Lipids and lipid-like molecules	Glycerophospholipids	Glycerophosphocholines	Phosphocholines	97541.80668	94290.92154	97634.76647	95939.74862
No results					584564.8972	585355.8265	593093.823	809021.4759
Full match	Organoheterocyclic compounds	Azepines	Not available	Azepines	384424.0214	400177.9265	334238.7793	326870.6407
No results					1002009.454	1377869.685	847148.204	1362194.911
No results					15772294.56	16775279.82	15332073.44	16690879.23
No results					1258751.574	1495301.064	1093953.412	840702.2911
No results					7659009.184	7456839.152	565886.0675	488916.3375
Full match	Organoheterocyclic compounds	Azoles	Pyrazoles	Pyrazole-4-carboxamides	4266494.623	3823232.628	3881641.324	3647341.422
Full match					1732044.6	1767902.601	1742712.26	1629037.002
Full match	Organosulfur compounds	Organic disulfides	Dialkyldisulfides	Dialkyldisulfides	2683708.694	2481876.955	2488379.462	2079864.057
No results					907549.6586	658967.6808	719209.567	715410.2264
Full match	Organoheterocyclic compounds	Biotin and derivatives	Not available	Biotin and derivatives	1925843.381	718228.445	665330.7918	969659.3071
No results					1062462.554	1123223.041	790162.6023	939447.837
No results					586416.7156	586126.4041	665197.2997	632878.1517
No results					16740393.84	17086965.21	16843497.9	15744814.53
Full match	Organic acids and derivatives	Carboxylic acids and derivatives	Amino acids, peptides, and analogues	Dipeptides	3079009.248	3241420.25	3516930.087	2534469.814
Full match	Nucleosides, nucleotides, and analogues	Purine nucleosides	Not available	Purine nucleosides	3463013.979	6302175.359	6248536.779	8157957.156
No results					354462.1834	310786.492	276809.126	317519.2098
Full match	Organic acids and derivatives	Keto acids and derivatives	Beta-keto acids and derivatives	Beta-keto acids and derivatives	2524991.073	13537363.61	1414989.579	12557382.86
No results					620992.7305	771411.6894	823621.2055	905322.233
Full match	Organic acids and derivatives	Carboxylic acids and derivatives	Amino acids, peptides, and analogues	Peptides	696729.4717	810170.2007	702524.49	706084.6439
Full match	Lipids and lipid-like molecules	Steroids and steroid derivatives	Androstane steroids	Androgens and derivatives	4075369.364	622828.64	676516.4165	628801.6872
No results					417918.9313	563839.8134	350061.0708	613517.7789
No results					1305459.267	1307282.309	1243633.329	975292.7494
No results					393321.6092	337421.7916	336737.7688	436931.2004
No results					132996.1388	127834.6912	117103.3445	108771.1445
Full match	Lipids and lipid-like molecules	Glycerophospholipids	Glycerophosphates	1-acylglycerol-3-phosphates	297672.5171	447222.2532	394774.1786	373572.5645
No results					9971882.472	9830470.988	7808697.488	7094372.159
Full match	Lipids and lipid-like molecules	Steroids and steroid derivatives	Androstane steroids	Androgens and derivatives	149801.5381	141921.9896	145453.8895	147942.8791
Full match					570149.0058	649744.6307	536522.2815	502545.9154
Full match	Lipids and lipid-like molecules	Fatty Acyls	Fatty acids and conjugates	Long-chain fatty acids	2524404.899	3363098.708	2568149.801	3127956.54
No results					1195030.937	1787278.338	1473811.786	1571043.209
Full match	Lipids and lipid-like molecules	Glycerophospholipids	Glycerophosphoethanolamines	1-acyl-sn-glycero-3-phosphoethanolamines	118778.6666	117666.3246	112577.0425	99231.3911
Full match					440607.5167	696503.243	369178.1289	560689.9052

MassList_Results	Class_I	Class_II	Class_III	Class_IV	all_YM1_1	all_YM1_2	all_YM1_3	all_YM1_4
No results					1006396.322	521439.8706	1096199.991	322233.0568
Full match	Benzenoids	Phenols	Methoxyphenols	Methoxyphenols	387289.2341	443652.5097	328575.94	323888.4763
Full match	Organoheterocyclic compounds	Quinolines and derivatives	Quinoline carboxylic acids	Quinoline carboxylic acids	170164.9242	187303.9448	145998.1955	121803.3374
No results	Lipids and lipid-like molecules	Fatty Acyls	Fatty acids and conjugates	Long-chain fatty acids	865076.558	939833.4092	1143426.539	792624.1043
Full match	Organic acids and derivatives	Carboxylic acids and derivatives	Amino acids, peptides, and analogues	Peptides	481351.0652	375265.349	684952.1712	558869.0966
No results					223957.0608	212275.9119	401234.5382	184787.5336
Full match	Nucleosides, nucleotides, and analogues	5'-deoxyribonucleosides	5'-deoxy-5'-thionucleosides	5'-deoxy-5'-thionucleosides	921485.5194	751190.7135	830420.3392	851991.1003
Full match	Organoheterocyclic compounds	Indoles and derivatives	Indolyl carboxylic acids and derivatives	Indole-3-acetic acid derivatives	383083.9235	440524.0822	604122.9343	449601.4818
No results					550344.2643	330512.6376	376390.8997	239124.3259
Full match	Organic oxygen compounds	Organooxygen compounds	Carbonyl compounds	Cyclohexenones	387316080.3	1189766071	2138179610	1826450744
Full match	Organoheterocyclic compounds	Indoles and derivatives	Indolyl carboxylic acids and derivatives	Indolyl carboxylic acids and derivatives	1517219468	1559261838	1497017452	1512005521
Full match	Organic acids and derivatives	Carboxylic acids and derivatives	Amino acids, peptides, and analogues	Dipeptides	1175149189	1031614210	1063417802	1055376570
No results					621564700.4	565265370.5	625694235.3	540389929
Full match	Organic acids and derivatives	Carboxylic acids and derivatives	Amino acids, peptides, and analogues	Alpha amino acids	1558983645	1520345377	1527794230	1537834487
Full match	Organic acids and derivatives	Carboxylic acids and derivatives	Amino acids, peptides, and analogues	Peptides	3719002160	3073101320	3091031188	2710316999
Full match	Organoheterocyclic compounds	Pyridines and derivatives	Pyridinecarboxylic acids and derivatives	Pyridinecarboxylic acids	49103694.38	54437290.72	43876636.45	30914495.22
Full match	Organic acids and derivatives	Carboxylic acids and derivatives	Amino acids, peptides, and analogues	Isoleucine and derivatives	3069715386	2852712269	3016594638	2814879982
Full match	Organic acids and derivatives	Carboxylic acids and derivatives	Amino acids, peptides, and analogues	Tyrosine and derivatives	2390138192	2198483815	2463042575	2318410696
Full match	Nucleosides, nucleotides, and analogues	Purine nucleosides	Not available	Purine nucleosides	1956194085	1289009236	2334650147	2069148902
No results	Lipids and lipid-like molecules	Fatty Acyls	Eicosanoids	Prostaglandins and related compounds	8436756.995	10029477.26	10059723.24	9402558.854
No results					217829571	222973717.5	227397304.9	264498094.4
Full match	Organic acids and derivatives	Carboxylic acids and derivatives	Amino acids, peptides, and analogues	Alpha amino acids and derivatives	2559573193	3066431517	3259659117	258536353.5
Full match	Lipids and lipid-like molecules	Glycerophospholipids	Glycerophosphoethanolamines	1-acyl-sn-glycero-3-phosphoethanolamines	66181689.36	62288152.16	77442972.62	84525402.24
Full match	Organic acids and derivatives	Carboxylic acids and derivatives	Amino acids, peptides, and analogues	Histidine and derivatives	268960953.8	278395573.6	294372592.1	294463112.6
No results	Organic acids and derivatives	Carboxylic acids and derivatives	Amino acids, peptides, and analogues	Dipeptides	269467256.9	225195541.8	227767299.2	247047801.4
Full match	Organic acids and derivatives	Carboxylic acids and derivatives	Amino acids, peptides, and analogues	Peptides	153472792.9	95730285.4	133944367.3	152668628.9
Full match	Lipids and lipid-like molecules	Glycerophospholipids	Glycerophosphoethanolamines	1-acyl-sn-glycero-3-phosphoethanolamines	47871464.72	37642155.74	41811585.03	41785546.46
Full match	Organic acids and derivatives	Carboxylic acids and derivatives	Amino acids, peptides, and analogues	Peptides	92962200.22	96856933.17	104127662.2	95980128.81
Full match	Organoheterocyclic compounds	Lactones	Gamma butyrolactones	Gamma butyrolactones	304682346.5	261046024.5	258187597.6	290191127.9
Full match	Lipids and lipid-like molecules	Steroids and steroid derivatives	Androstane steroids	Androgens and derivatives	29133906.93	5605779.945	10623304.47	3741579.639
Full match	Organic acids and derivatives	Carboxylic acids and derivatives	Amino acids, peptides, and analogues	Proline and derivatives	244140461.3	227279264.8	139247327.5	274359905.3
Full match	Organoheterocyclic compounds	Indoles and derivatives	Pyrroloindoles	Pyrroloindoles	601754914.2	598906449.7	500388253.9	489152466.6
No match	Organic acids and derivatives	Carboxylic acids and derivatives	Amino acids, peptides, and analogues	Alpha amino acids and derivatives	15982212.89	12805314.67	14983821.67	11750595.72
Full match	Lipids and lipid-like molecules	Fatty Acyls	Fatty acid esters	Acyl carnitines	187877964	149162636.8	141433945.4	157382262.8
Full match	Organic acids and derivatives	Carboxylic acids and derivatives	Amino acids, peptides, and analogues	Peptides	57493231.39	50051960.78	42569326.89	48060948.22
Full match	Benzenoids	Phenols	Benzenediols	Catechols	5225046.64	6484651.537	5335173.614	3469389.312
Full match					78640089.57	68340130.83	80919618.73	66003930.04
Full match	Nucleosides, nucleotides, and analogues	(5'->5')-dinucleotides	Not available	(5'->5')-dinucleotides	96227210.45	112822734.2	69395576.21	65433657.67
Full match	Organic oxygen compounds	Organooxygen compounds	Carbonyl compounds	Hydroxybenzaldehydes	307196120.5	95621867.93	315320190.9	299296583.7
Full match	Organic acids and derivatives	Carboxylic acids and derivatives	Amino acids, peptides, and analogues	Peptides	243827011.1	254470648	264316810.5	237394966
No results					29240110.9	20248463.07	20765337.83	35534249.2
Full match	Organic acids and derivatives	Carboxylic acids and derivatives	Amino acids, peptides, and analogues	Peptides	6578604.385	7643229.821	6521499.696	6730071.441
Full match	Organic oxygen compounds	Organooxygen compounds	Carbonyl compounds	Alkyl-phenylketones	75851667.36	73494702.2	70838600.67	66323365.08
No results	Organic acids and derivatives	Carboxylic acids and derivatives	Amino acids, peptides, and analogues	Dipeptides	123940902.8	81773665.45	101064799.4	102975157.3
Full match	Nucleosides, nucleotides, and analogues	Purine nucleosides	Not available	Purine nucleosides	1460088.745	2515096.835	2448442.428	1337300.934
Full match	Organic acids and derivatives	Carboxylic acids and derivatives	Amino acids, peptides, and analogues	Leucine and derivatives	81887564.48	86832361.32	56577015.09	75447607.49
No results					44631705.85	39453666.98	39506086.44	42567077.07
No results	Lipids and lipid-like molecules	Glycerophospholipids	Glycerophosphocholines	Phosphocholines	64079791.74	55177457.31	68304157.13	71044770.52
Full match	Organic acids and derivatives	Carboxylic acids and derivatives	Amino acids, peptides, and analogues	Dipeptides	31487440.52	32246698.84	31959962.88	23420078.15
Full match	Organoheterocyclic compounds	Azolines	Imidazolines	Imidazolines	1009911656	1285527845	800042082.5	706610025.4
No results					8217788.236	7840154.104	8611775.463	8035807.348
Full match	Organic acids and derivatives	Carboxylic acids and derivatives	Amino acids, peptides, and analogues	Dipeptides	24733410.15	27185050.72	29537292.82	29321791.74
Full match	Organoheterocyclic compounds	Imidazopyrimidines	Purines and purine derivatives	Xanthines	3380279.589	1973174.162	3553616.29	1683324.703

MassList_Results	Class_I	Class_II	Class_III	Class_IV	all_YM1_1	all_YM1_2	all_YM1_3	all_YM1_4
Full match	Organic oxygen compounds	Organooxygen compounds	Carbonyl compounds	Alkyl-phenylketones	5082961.763	4677302.848	6529690.776	5099126.386
No results	Organic acids and derivatives	Carboxylic acids and derivatives	Amino acids, peptides, and analogues	Peptides	57003800.56	48201306.75	69107331.63	48770500.39
Full match	Organic acids and derivatives	Carboxylic acids and derivatives	Amino acids, peptides, and analogues	Histidine and derivatives	88731583.83	104407114	105973333.9	96817418.59
Full match	Lipids and lipid-like molecules	Glycerophospholipids	Glycerophosphoethanolamines	1-(1Z-alkenyl)-glycero-3-phosphoethanolamines	17050629.53	16577916.11	19956143.21	25818728.22
Full match	Organic acids and derivatives	Organic sulfonic acids and derivatives	Organosulfonic acids and derivatives	Organosulfonic acids	3821721.641	1204439.88	4502770.493	3937963.851
Full match	Benzenoids	Phenols	Benzenediols	Catechols	29149291.41	17722342.95	17487421.91	27852666.37
Full match	Nucleosides, nucleotides, and analogues	5'-deoxyribonucleosides	5'-deoxy-5'-thionucleosides	5'-deoxy-5'-thionucleosides	51671878.94	46995151.05	65244054.92	52347658.44
Full match					80026304.88	64987080.02	108666230.8	78679348.07
Full match	Organoheterocyclic compounds	Diazines	Pyrimidines and pyrimidine derivatives	Pyrimidones	11812626.59	13374945.98	15800918.54	22687981.24
Full match	Organic acids and derivatives	Carboxylic acids and derivatives	Amino acids, peptides, and analogues	Dipeptides	31643263.85	19145495.41	23364231.72	23549011.57
No results					13204518.62	11156240.61	11752258.8	10878430.66
No results	Lipids and lipid-like molecules	Glycerophospholipids	Glycerophosphoethanolamines	1-acyl-sn-glycero-3-phosphoethanolamines	2454090.236	1820720.219	2369500.054	2278605.851
No results	Lipids and lipid-like molecules	Fatty Acyls	Eicosanoids	Thromboxanes	8001972.353	6017842.81	17604466.01	20962260.54
No results	Lipids and lipid-like molecules	Glycerophospholipids	Glycerophosphocholines	Phosphocholines	23008901.46	20994555.49	21132917.11	22529141.76
Full match	Organic nitrogen compounds	Organonitrogen compounds	Amines	Aralkylamines	39451131.72	53019517.99	61371351.16	57382844.79
Full match	Organic acids and derivatives	Carboxylic acids and derivatives	Amino acids, peptides, and analogues	N-acyl-alpha amino acids	38027830.54	34424496.33	29252613.82	33028988.53
No results	Lipids and lipid-like molecules	Glycerophospholipids	Glycerophosphoethanolamines	1-acyl-sn-glycero-3-phosphoethanolamines	5986898.804	4812041.323	5568868.003	7384578.38
Full match	Nucleosides, nucleotides, and analogues	Pyrimidine nucleotides	Pyrimidine nucleotide sugars	Pyrimidine nucleotide sugars	69962173.77	61545998.71	55246638.05	42127188.04
Full match	Lipids and lipid-like molecules	Fatty Acyls	Fatty acids and conjugates	Long-chain fatty acids	5737754.149	4932868.412	6850442.707	4969719.227
No results	Lipids and lipid-like molecules	Fatty Acyls	Eicosanoids	Prostaglandins and related compounds	2912061.014	2245488.255	2024315.233	2609730.42
Full match	Lipids and lipid-like molecules	Fatty Acyls	Eicosanoids	Prostaglandins and related compounds	14033940.55	14984608.73	15571972.99	5353940.661
Full match	Phenylpropanoids and polyketides	Phenylpropanoic acids	Not available	Phenylpropanoic acids	5151011.783	3818715.615	7034530.164	7675596.854
No results	Lipids and lipid-like molecules	Glycerophospholipids	Glycerophosphocholines	Phosphocholines	19626365.48	18461640.58	22557710.32	23732632
No results	Lipids and lipid-like molecules	Glycerophospholipids	Glycerophosphocholines	Phosphocholines	2687843.1	2254560.747	2191915.208	6114649.615
No results	Organic acids and derivatives	Carboxylic acids and derivatives	Amino acids, peptides, and analogues	Methionine and derivatives	1797113.116	1716171.539	2907050.471	2013278.255
Full match	Benzenoids	Phenols	Cresols	Ortho cresols	18760055.82	19607729.81	19853461.14	17824908.16
No results	Lipids and lipid-like molecules	Glycerophospholipids	Glycerophosphocholines	Phosphocholines	14316287.93	13319623.44	17921581.57	18859136.71
Full match					12307735.68	7400172.721	10421660.15	8536480.84
No results					32327920.54	30196305.54	28280292.16	27373758.15
Full match	Organic acids and derivatives	Hydroxy acids and derivatives	Medium-chain hydroxy acids and derivatives	Medium-chain hydroxy acids and derivatives	14443813.88	13963190.21	12966830.31	21742773.07
No results	Lipids and lipid-like molecules	Glycerophospholipids	Glycerophosphoethanolamines	1-acyl-sn-glycero-3-phosphoethanolamines	26029097.92	24021348.43	32540569.57	33693966.24
Full match	Organic acids and derivatives	Carboxylic acids and derivatives	Amino acids, peptides, and analogues	Alpha amino acids and derivatives	87295728.24	80597335.57	70556690.85	29220923.38
No results	Organic acids and derivatives	Carboxylic acids and derivatives	Amino acids, peptides, and analogues	Dipeptides	6126838.377	4220997.26	5083930.812	4766576.779
Full match	Lipids and lipid-like molecules	Steroids and steroid derivatives	Estrane steroids	Estrogens and derivatives	1231008.979	1371861.318	1209925.347	1111774.9
Full match	Organic acids and derivatives	Carboxylic acids and derivatives	Amino acids, peptides, and analogues	Peptides	7125481.7	4550830.441	6278371.126	6694000.609
No results	Lipids and lipid-like molecules	Glycerophospholipids	Glycerophosphocholines	Phosphocholines	16218738.93	13267566.83	15307807.22	16800156.48
No results					6754503.981	6091290.505	6418497.123	6511534.327
No results					23508928.88	22640570.35	27337970.57	22287794.26
Full match	Lipids and lipid-like molecules	Fatty Acyls	Fatty acid esters	Acyl carnitines	34044341.43	37241344.01	34276005.16	32497455.4
Full match					6939672.515	7152347.471	7100028.62	6682476.249
No results	Lipids and lipid-like molecules	Steroids and steroid derivatives	Hydroxysteroids	21-hydroxysteroids	3510521.597	6631022.498	6861664.177	8144559.286
No results					11422008.33	4080114.206	11880271.54	14300653.66
Full match	Organoheterocyclic compounds	Indoles and derivatives	Indoles	3-alkylindoles	6989274.578	5071755.294	6401195.543	4407808.618
Full match	Organic acids and derivatives	Carboxylic acids and derivatives	Amino acids, peptides, and analogues	Oligopeptides	4549679.706	3112379.687	4017408.407	3803143.099
Full match	Lipids and lipid-like molecules	Fatty Acyls	Fatty acyl glycosides	Fatty acyl glycosides of mono- and disaccharides	43165230.63	43270200.05	40251448.97	36156313.06
Full match	Lipids and lipid-like molecules	Fatty Acyls	Fatty acids and conjugates	Long-chain fatty acids	6030373.866	3896963.224	5203891.021	4921440.941
No results					4963208.037	2789852.786	4487453.763	3188622.433
Full match	Lipids and lipid-like molecules	Prenol lipids	Retinoids	Retinoids	2643805.768	486626.9058	799160.0745	530973.0298
Full match	Benzenoids	Benzene and substituted derivatives	Phenylpyruvic acid derivatives	Phenylpyruvic acid derivatives	12711043.79	9031950.003	12409913.09	13148554.37
Full match	Organic acids and derivatives	Peptidomimetics	Hybrid peptides	Hybrid peptides	81898242.06	82496426.69	77848998.1	74372314.07
No results	Lipids and lipid-like molecules	Glycerophospholipids	Glycerophosphoethanolamines	1-acyl-sn-glycero-3-phosphoethanolamines	2437382.175	1312504.546	1582121.822	2884124.379
Full match	Organoheterocyclic compounds	Indoles and derivatives	Indoles	Indoles	646713.7994	3485990.118	3544544.754	4254457.535
Full match	Lipids and lipid-like molecules	Steroids and steroid derivatives	Bile acids, alcohols and derivatives	Trihydroxy bile acids, alcohols and derivatives	184686.742	173572.63	191181.4205	166373.8721

MassList_Results	Class_I	Class_II	Class_III	Class_IV	all_YM1_1	all_YM1_2	all_YM1_3	all_YM1_4
Full match	Lipids and lipid-like molecules	Glycerophospholipids	Glycerophosphocholines	1-acyl-sn-glycero-3-phosphocholines	712653.3638	893617.9443	761240.4673	749828.4893
Full match	Organic acids and derivatives	Carboxylic acids and derivatives	Amino acids, peptides, and analogues	Peptides	3196908.409	3194062.828	3222509.585	2879584.789
Full match	Lipids and lipid-like molecules	Fatty Acyls	Fatty acids and conjugates	Medium-chain fatty acids	1297072.672	1728518.673	8321337.085	10567119.7
Full match	Nucleosides, nucleotides, and analogues	Flavin nucleotides	Not available	Flavin nucleotides	3075670.864	2559968.196	2680366.347	2314020.895
Full match	Lipids and lipid-like molecules	Fatty Acyls	Fatty acid esters	Acyl carnitines	3424758.829	3451696.557	3359514.851	3310894.146
Full match	Organoheterocyclic compounds	Indoles and derivatives	Hydroxyindoles	Hydroxyindoles	959772.8899	942287.3782	1237055.707	1058760.608
Full match	Lipids and lipid-like molecules	Fatty Acyls	Fatty acid esters	Acyl carnitines	10603933.97	21681178	12305838.47	11261157.08
Full match	Organic acids and derivatives	Carboxylic acids and derivatives	Amino acids, peptides, and analogues	Alpha amino acids and derivatives	624142.5764	357800.1758	529864.5168	582158.4452
Full match	Lipids and lipid-like molecules	Fatty Acyls	Eicosanoids	Prostaglandins and related compounds	2002397.877	1767653.425	2269035.477	1016954.145
Full match	Lipids and lipid-like molecules	Steroids and steroid derivatives	Androstane steroids	Androgens and derivatives	941773.5388	297654.5117	323884.3763	487591.7316
Full match	Lipids and lipid-like molecules	Fatty Acyls	Fatty acids and conjugates	Medium-chain fatty acids	1417811.559	1020856.857	1018826.55	934347.2078
Full match	Lipids and lipid-like molecules	Glycerophospholipids	Glycerophosphocholines	Phosphocholines	1393924.468	1848702.83	1372561.127	1451106.848
Full match	Organic acids and derivatives	Carboxylic acids and derivatives	Amino acids, peptides, and analogues	Dipeptides	1103644.814	1478899.672	1130509.343	1453293.223
Full match	Lipids and lipid-like molecules	Fatty Acyls	Fatty acids and conjugates	Medium-chain fatty acids	1518716.336	1096322.668	2116564.961	1035668.664
Full match	Organic acids and derivatives	Carboxylic acids and derivatives	Amino acids, peptides, and analogues	Beta amino acids and derivatives	640872.7657	1948565.208	1349025.128	198453.8166
No results	Lipids and lipid-like molecules	Fatty Acyls	Fatty acids and conjugates	Long-chain fatty acids	282565823.7	215404705.4	220924410.1	277782713.5
Full match	Organic acids and derivatives	Keto acids and derivatives	Gamma-keto acids and derivatives	Gamma-keto acids and derivatives	3248752118	2479494087	2248822502	2593081676
Full match	Lipids and lipid-like molecules	Prenol lipids	Retinoids	Retinoids	321157583.4	30875588.98	50361330	11022781.43
No results	Lipids and lipid-like molecules	Fatty Acyls	Fatty acids and conjugates	Very long-chain fatty acids	29227935.87	21889308.58	27074236.5	45187663.93
No match	Organic acids and derivatives	Carboxylic acids and derivatives	Tricarboxylic acids and derivatives	Tricarboxylic acids and derivatives	1345255353	1140327006	1281889740	1469629227
Full match	Organic acids and derivatives	Peptidomimetics	Hybrid peptides	Hybrid peptides	1287721428	1578677018	1693074214	1739788690
No results	Lipids and lipid-like molecules	Steroids and steroid derivatives	Estrane steroids	Estrane steroids	192283621.5	1093445447	768317072.1	126029136.6
Full match	Organic oxygen compounds	Organooxygen compounds	Carbohydrates and carbohydrate conjugates	Sugar acids and derivatives	286890889.6	218067636.5	378384901.6	287058173.8
No results	Lipids and lipid-like molecules	Glycerophospholipids	Glycerophosphocholines	Phosphocholines	106643612	90472219.87	106515055.1	116174112
No results					378161694.1	303593640.8	316933735.2	349694240.7
Full match	Lipids and lipid-like molecules	Fatty Acyls	Fatty acids and conjugates	Long-chain fatty acids	20678325.72	15808283.33	15113672.94	18816155.14
No results					82613223.13	77413938.94	76559439.55	93040301.27
No match	Lipids and lipid-like molecules	Fatty Acyls	Fatty acids and conjugates	Long-chain fatty acids	58247340.31	38667584.1	39677206.94	54949535.86
Full match	Organoheterocyclic compounds	Furans	Furoic acid and derivatives	Furoic acids	238399342.8	198780154.9	179705649.6	204914508.4
Full match	Organic oxygen compounds	Organooxygen compounds	Carbohydrates and carbohydrate conjugates	Hexose phosphates	96604007.67	109788997.7	182741735.4	144424186.4
Full match					908968.7087	984497.8751	494078.1769	350054.7649
Full match					586184.4686	548703.8821	1939413.899	545946.4962
No results	Lipids and lipid-like molecules	Fatty Acyls	Eicosanoids	Prostaglandins and related compounds	72671161.74	434892448.3	307199393.8	48258013.75
Full match	Organic oxygen compounds	Organooxygen compounds	Carbohydrates and carbohydrate conjugates	Monosaccharides	16169421.45	17194385.79	12698342.98	17069842.35
Full match	Organic acids and derivatives	Carboxylic acids and derivatives	Carboxylic acids	Carboxylic acids	122126689.4	147589905.4	264907115	122651954.8
Full match	Lipids and lipid-like molecules	Fatty Acyls	Fatty acids and conjugates	Long-chain fatty acids	23349107.62	32134792.29	36343131.19	34379300.15
No results					856128.5234	944471.6127	1320756.838	754204.2288
No results					57486040.92	39993330.71	41726587.74	65318794.62
No results					41716879.65	36171177.95	35941756.47	35279322.21
No results	Lipids and lipid-like molecules	Fatty Acyls	Fatty acids and conjugates	Long-chain fatty acids	19413563.46	15220321.65	15470582.57	19237064.26
No match	Organoheterocyclic compounds	Imidazopyrimidines	Purines and purine derivatives	Xanthines	27269769.72	32643693.78	26392146.15	26111982.46
No results					12491527.11	9863125.019	10481917	9247391.24
No results	Lipids and lipid-like molecules	Glycerophospholipids	Glycerophosphoinositols	Phosphatidylinositols	213707176	177938802.7	183067801.3	184085213.6
No results					10400862.53	7456933.919	7642582.351	10234036.2
Full match	Organic oxygen compounds	Organooxygen compounds	Carbohydrates and carbohydrate conjugates	Sugar acids and derivatives	31664241.41	25863503.81	18170049.84	24345797.07
No results	Lipids and lipid-like molecules	Glycerophospholipids	Glycerophosphocholines	Phosphocholines	80399510.71	69211059.03	101441132.5	122993914.3
Full match	Lipids and lipid-like molecules	Fatty Acyls	Eicosanoids	Hydroxyeicosatetraenoic acids	201230.4416	231690.9069	228966.0751	194159.1641
Full match	Organic acids and derivatives	Carboxylic acids and derivatives	Amino acids, peptides, and analogues	Aspartic acid and derivatives	65915128.93	57900662.58	45665439.24	56915185.33
No results					26811784.42	18946078.82	19436162.88	26403484.46
Full match	Nucleosides, nucleotides, and analogues	Pyrimidine nucleotides	Pyrimidine ribonucleotides	Pyrimidine ribonucleoside monophosphates	143754694.1	195534382.5	128164828	132350261.2
No results	Lipids and lipid-like molecules	Fatty Acyls	Fatty acids and conjugates	Long-chain fatty acids	15095626.65	9881095.17	8675746.4	12191265.63
No results	Lipids and lipid-like molecules	Glycerophospholipids	Glycerophosphoinositols	Phosphatidylinositols	115041065.1	91641619.15	84726100.59	95197173.36
Full match	Lipids and lipid-like molecules	Fatty Acyls	Fatty acids and conjugates	Long-chain fatty acids	10474517.7	6828867.423	8344869.425	10114818.59

MassList_Results	Class_I	Class_II	Class_III	Class_IV	all_YM1_1	all_YM1_2	all_YM1_3	all_YM1_4
No match	Lipids and lipid-like molecules	Fatty Acyls	Fatty acids and conjugates	Hydroxy fatty acids	5096431.926	4272071.125	4347347.231	4712036.782
Full match					7716402.001	9853746.313	10037500.39	10914652.44
Full match					22194659.61	19327895.38	21530909.32	29430351.88
Full match	Lipids and lipid-like molecules	Fatty Acyls	Fatty acids and conjugates	Medium-chain fatty acids	25725995.97	27711514.68	24098459.88	21948726.27
Full match	Organic oxygen compounds	Organooxygen compounds	Carbohydrates and carbohydrate conjugates	C-glycosyl compounds	97338754.9	134481812.5	134519739.8	139804699.6
No results	Lipids and lipid-like molecules	Glycerophospholipids	Glycerophosphoglycerols	Phosphatidylglycerols	14759177.06	14719486.18	15826754.55	14433017.68
Full match	Lipids and lipid-like molecules	Fatty Acyls	Eicosanoids	Hydroxyeicosatetraenoic acids	3815376.243	6203309.286	6011697.017	8987911.436
No results	Lipids and lipid-like molecules	Glycerophospholipids	Glycerophosphoinositols	Phosphatidylinositols	23258226.39	17214362.63	19102019.95	18876427.61
No results	Lipids and lipid-like molecules	Fatty Acyls	Lineolic acids and derivatives	Lineolic acids and derivatives	8505899.218	18256919.68	11279633.53	21130440.63
No results	Lipids and lipid-like molecules	Fatty Acyls	Eicosanoids	Hydroxyeicosatrienoic acids	9775881.889	7868316.523	7862024.352	5932586.316
Full match	Organic oxygen compounds	Organooxygen compounds	Carbohydrates and carbohydrate conjugates	Hexose phosphates	19583288.21	17838986.67	25714327.83	19889101.69
No match	Lipids and lipid-like molecules	Fatty Acyls	Eicosanoids	Prostaglandins and related compounds	17668974.49	2472962.396	3081742.347	4883451.4
No results	Lipids and lipid-like molecules	Steroids and steroid derivatives	Hydroxysteroids	21-hydroxysteroids	19713920.46	16749460.41	19981584.21	19851396.97
Full match	Lipids and lipid-like molecules	Glycerophospholipids	Glycerophosphoglycerols	Phosphatidylglycerols	105127594.2	92560620.08	102094656.2	100890897.7
No results	Lipids and lipid-like molecules	Glycerophospholipids	Glycerophosphoserines	Phosphatidylserines	7241405.81	6310065.728	6255239.079	6310485.418
No results	Lipids and lipid-like molecules	Glycerophospholipids	Glycerophosphoinositols	Phosphatidylinositols	20139278.61	14857460.87	14123942.17	16692083.96
Full match	Phenylpropanoids and polyketides	Cinnamic acids and derivatives	Hydroxycinnamic acids and derivatives	Coumaric acids and derivatives	28942343.31	28431542.44	42850290.45	39716136.7
Full match	Organic acids and derivatives	Carboxylic acids and derivatives	Dicarboxylic acids and derivatives	Dicarboxylic acids and derivatives	54385423.83	53056308.46	42469064.27	52178105.22
No match	Lipids and lipid-like molecules	Glycerophospholipids	Glycerophosphoserines	Phosphatidylserines	363613990.7	288603234.6	305965004.7	393703853.7
No results	Organic acids and derivatives	Carboxylic acids and derivatives	Amino acids, peptides, and analogues	Dipeptides	32483780.76	20537645.09	30304651.99	26371191.81
Full match	Lipids and lipid-like molecules	Fatty Acyls	Fatty acyl glycosides	Fatty acyl glycosides of mono- and disaccharides	313744317.8	345360812.9	420619463.2	333407156.3
No results	Lipids and lipid-like molecules	Glycerophospholipids	Glycerophosphoglycerols	Phosphatidylglycerols	54099621.78	40828128.78	33866372.12	41380227.66
No results	Lipids and lipid-like molecules	Fatty Acyls	Eicosanoids	Hydroxyeicosapentaenoic acids	800572.4441	1254089.245	1119104.37	1476399.761
No results					1756802.061	850284.5154	993942.3843	1120285.701
No match	Organic oxygen compounds	Organooxygen compounds	Carbohydrates and carbohydrate conjugates	Pentoses	5226104.832	3577708.728	1648510.064	799457.2385
Full match					9011485.26	2184243.798	1065448.983	3704079.033
No match	Organic acids and derivatives	Carboxylic acids and derivatives	Amino acids, peptides, and analogues	Dipeptides	425497.628	1007857.808	31594690.38	26781519.72
Full match	Lipids and lipid-like molecules	Fatty Acyls	Eicosanoids	Prostaglandins and related compounds	261851.9613	258185.3836	249282.9884	625067.3041
No results	Lipids and lipid-like molecules	Glycerophospholipids	Glycerophosphoserines	Phosphatidylserines	6934270.791	5117516.702	4919104.906	6160494.391
No results	Lipids and lipid-like molecules	Fatty Acyls	Eicosanoids	Prostaglandins and related compounds	6841495.881	7883753.207	8399404.919	11923280.96
No results	Lipids and lipid-like molecules	Fatty Acyls	Fatty acids and conjugates	Long-chain fatty acids	3289140.987	5165600.283	4263240.596	5335849.527
Full match					48984847.1	6817152.941	10817965.14	2427354.302
No results					65264922.38	59501688.41	66399029.91	68781039.91
No results	Lipids and lipid-like molecules	Glycerophospholipids	Glycerophosphoinositols	Phosphatidylinositols	74698779.02	62766360.72	69105910.53	70007016.34
Full match	Lipids and lipid-like molecules	Glycerophospholipids	Glycerophosphoethanolamines	1-acyl-sn-glycero-3-phosphoethanolamines	18427660.98	11262703.42	15552168.73	15598952.48
No results	Lipids and lipid-like molecules	Glycerophospholipids	Glycerophosphocholines	Phosphocholines	3814981.567	2886563.103	3634178.135	3816292.905
Full match	Benzenoids	Benzene and substituted derivatives	Benzoic acids and derivatives	Benzoic acid esters	7758605.999	11804577.29	15824312.17	13001860.71
Full match	Organic acids and derivatives	Carboxylic acids and derivatives	Amino acids, peptides, and analogues	Isoleucine and derivatives	4357523.005	4470119.287	3963449.905	4265728.769
Full match	Nucleosides, nucleotides, and analogues	Purine nucleotides	Purine ribonucleotides	Purine ribonucleoside monophosphates	67451557.81	62924035.98	64023566.75	50848813.61
Full match					5072865.838	5165600.283	5496132.817	5335849.527
Full match					10959986.07	8304428.831	12501774.78	10196918.4
No results					8812029.023	6302548.604	5999619.109	12357236.63
No results					15844859.31	729963.5821	1088981.305	1016480.602
Full match					2520830.833	2478356.394	3129534.75	2542787.848
No match	Lipids and lipid-like molecules	Fatty Acyls	Eicosanoids	Prostaglandins and related compounds	2552526.737	2287640.187	2648232.462	3812532.569
No results					227716.3813	193891.7807	205747.2428	232137.4538
Full match	Organic acids and derivatives	Carboxylic acids and derivatives	Amino acids, peptides, and analogues	L-alpha-amino acids	26453072	23639173.57	26822877.77	28475118.67
Full match	Lipids and lipid-like molecules	Fatty Acyls	Eicosanoids	Leukotrienes	1338350.73	1486509.881	1104579.152	2309657.489
No results					2213443.281	1864095.699	2513811.397	2352077.007
No results					4360720.266	3221237.825	3850516.324	3838917.026
No results	Lipids and lipid-like molecules	Glycerophospholipids	Glycerophosphoserines	Phosphatidylserines	3532922.931	3489037	4796037.236	3706961.998
Full match	Lipids and lipid-like molecules	Glycerophospholipids	Glycerophosphocholines	Phosphatidylcholines	3311751.516	2221187.089	2558718.421	2621686.808
No results					2256561.971	2152775.501	3140273.48	2173764.921

MassList_Results	Class_I	Class_II	Class_III	Class_IV	all_YM1_1	all_YM1_2	all_YM1_3	all_YM1_4
No results	Organic acids and derivatives	Hydroxy acids and derivatives	Medium-chain hydroxy acids and derivatives	Medium-chain hydroxy acids and derivatives	12659907.89	11355105.2	12296464.02	14127642.23
Full match	Lipids and lipid-like molecules	Steroids and steroid derivatives	Bile acids, alcohols and derivatives	Trihydroxy bile acids, alcohols and derivatives	14085777.69	15981126.61	17779736.52	19806079.6
No results					8622094.163	7202325.237	8200462.026	8534739.241
Full match	Lipids and lipid-like molecules	Fatty Acyls	Fatty acids and conjugates	Branched fatty acids	43063741.79	43808345.89	46991120.72	48691966.17
No results					959318.405	576429.9153	698291.4429	964430.3313
No match	Lipids and lipid-like molecules	Fatty Acyls	Eicosanoids	Prostaglandins and related compounds	6803723.95	237852.1569	511566.7697	272036.7356
No results	Lipids and lipid-like molecules	Glycerophospholipids	Glycerophosphoinositols	Phosphatidylinositols	38224683.02	26600254.87	33604009.34	36843927.91
No results	Phenylpropanoids and polyketides	Cinnamic acids and derivatives	Cinnamic acids	Cinnamic acids	18479393.89	16291034.69	18329929.39	16936922.7
No results					17039439.53	16883583.98	15242734.03	13752518.14
Full match	Organic acids and derivatives	Carboxylic acids and derivatives	Amino acids, peptides, and analogues	L-alpha-amino acids	20215835.02	19523548.43	19622574.6	21434416.81
Full match	Lipids and lipid-like molecules	Prenol lipids	Monoterpenoids	Monocyclic monoterpenoids	15842273.19	21838582.16	23102666.56	17744619.37
No results					9634479.966	7665434.457	7603420.244	7718819.888
No results	Lipids and lipid-like molecules	Fatty Acyls	Fatty acids and conjugates	Very long-chain fatty acids	2259505.465	1707635.41	1731181.945	2289007.902
No results					31253086.58	25619049.83	32898006.48	22876172.41
Full match					16044908.14	11842680.31	16840493.06	15973296.42
No results	Lipids and lipid-like molecules	Glycerophospholipids	Glycerophosphoglycerols	Phosphatidylglycerols	8671780.005	6483844.065	6636530.931	9359330.932
Full match	Lipids and lipid-like molecules	Steroids and steroid derivatives	Bile acids, alcohols and derivatives	Taurinated bile acids and derivatives	3153267.863	2858855.548	3639738.929	3633749.074
No results	Lipids and lipid-like molecules	Fatty Acyls	Eicosanoids	Prostaglandins and related compounds	2264101.575	986117.8341	1373002.418	1831093.398
No results	Lipids and lipid-like molecules	Fatty Acyls	Fatty acid esters	Fatty acid esters	19809149.24	22812265.69	25110428.29	27724893.26
Full match	Organic oxygen compounds	Organooxygen compounds	Alcohols and polyols	Quinic acids and derivatives	10995209.51	13007369.31	15296074.68	17578532.93
Full match	Lipids and lipid-like molecules	Fatty Acyls	Eicosanoids	Hydroperoxyeicosapentaenoic acids	1562542.502	1538079.797	1112249.588	2638560.958
No results	Organic acids and derivatives	Carboxylic acids and derivatives	Amino acids, peptides, and analogues	Tyrosine and derivatives	4158793.971	3557197.59	4497527.043	4323967.237
Full match	Organic acids and derivatives	Hydroxy acids and derivatives	Medium-chain hydroxy acids and derivatives	Medium-chain hydroxy acids and derivatives	3764014.598	4869573.076	4144583.07	5448076.916
Full match	Organic acids and derivatives	Carboxylic acids and derivatives	Amino acids, peptides, and analogues	Histidine and derivatives	4008115.338	3259263.603	2850663.588	4674777.719
No results					22180539.29	19154344.87	22158016.62	679910.8726
No results	Lipids and lipid-like molecules	Steroids and steroid derivatives	Estrane steroids	Estrogens and derivatives	2189853.493	2280643.394	1914632.314	2493731.454
Full match					1968265.683	3032842.612	3380882.76	4565866.443
No results					2509714.902	2272247.285	2282594.307	636288.4001
Full match	Organoheterocyclic compounds	Pyridines and derivatives	Pyridine carboxaldehydes	Pyridoxals and derivatives	43293800.32	45400469.45	46976278.04	43019976.85
Full match	Nucleosides, nucleotides, and analogues	Purine nucleotides	Purine ribonucleotides	Purine ribonucleoside diphosphates	19278711.78	15861473.91	18810787.72	13001960.38
No results	Lipids and lipid-like molecules	Glycerophospholipids	Glycerophosphoinositols	Phosphatidylinositols	8807395.686	6569294.937	6496199.901	7452790.721
No results					178879.9059	152083.2871	162752.272	192838.9671
No results	Lipids and lipid-like molecules	Glycerophospholipids	Glycerophosphoserines	Phosphatidylserines	6850314.285	7342558.173	7864391.193	9130356.717
Full match	Organoheterocyclic compounds	Pteridines and derivatives	Pterins and derivatives	Biopterins and derivatives	4731818.259	5600264.543	13274160.49	3097983.116
Full match	Lipids and lipid-like molecules	Fatty Acyls	Fatty acids and conjugates	Medium-chain fatty acids	12035080.34	9631787.109	9841107.285	8530516.212
Full match	Organic oxygen compounds	Organooxygen compounds	Carbohydrates and carbohydrate conjugates	Sugar acids and derivatives	1767608.737	605848.7008	424238.5025	549632.5521
Full match	Organic acids and derivatives	Hydroxy acids and derivatives	Medium-chain hydroxy acids and derivatives	Medium-chain hydroxy acids and derivatives	329077.5051	316824.1412	323693.5412	341741.1439
Full match	Lipids and lipid-like molecules	Fatty Acyls	Fatty acids and conjugates	Hydroxy fatty acids	9876673.435	9041319.195	9035648.851	9041518.179
No results					990902.919	832298.6105	758200.2865	1635563.486
No match					1004344.03	1464739.078	2088901.656	855932.7513
No results	Lipids and lipid-like molecules	Glycerophospholipids	Glycerophosphoglycerols	Phosphatidylglycerols	8012548.147	7591634.686	7299618.204	6700031.827
No results	Lipids and lipid-like molecules	Glycerophospholipids	Glycerophosphoinositols	Phosphatidylinositols	5039943.335	4396699.811	3572780.4	5342713.284
Full match	Lipids and lipid-like molecules	Fatty Acyls	Fatty acid esters	Fatty acid esters	244420.3087	405891.4004	407919.3203	277223.1259
Full match	Organic oxygen compounds	Organooxygen compounds	Carbohydrates and carbohydrate conjugates	Hexose phosphates	2271411.546	2976464.075	3414506.685	3019593.962
No results	Lipids and lipid-like molecules	Fatty Acyls	Eicosanoids	Prostaglandins and related compounds	199002.6144	229125.8512	226431.1861	192009.6232
Full match	Organic acids and derivatives	Carboxylic acids and derivatives	Amino acids, peptides, and analogues	Glutamic acid and derivatives	623272.3067	1006242.612	648961.2982	630030.7662
No results	Lipids and lipid-like molecules	Fatty Acyls	Eicosanoids	Prostaglandins and related compounds	2273580.114	358661.1033	733449.6605	499227.5552
No results	Lipids and lipid-like molecules	Glycerophospholipids	Glycerophosphocholines	Phosphocholines	1486943.531	1582424.49	1783501.958	2354808.689
No results	Lipids and lipid-like molecules	Fatty Acyls	Fatty acids and conjugates	Long-chain fatty acids	13624962.74	10321947.38	15550520.51	9044674.607
No results					4836400.795	4164070.06	4207375.078	4771500.79
Full match	Nucleosides, nucleotides, and analogues	Purine nucleotides	Purine nucleotide sugars	Purine nucleotide sugars	12877700.27	17464387.4	12567063.26	10156709.85
Full match	Organic acids and derivatives	Carboxylic acids and derivatives	Amino acids, peptides, and analogues	Alpha amino acids and derivatives	26666842.17	24572852.9	24566805.15	26095640.77
No results					142009.7581	141975.9383	139421.4633	155517.2321

MassList_Results	Class_I	Class_II	Class_III	Class_IV	all_YM1_1	all_YM1_2	all_YM1_3	all_YM1_4
No results					1989799.832	1396096.263	1473302.214	1959290.474
Full match	Organic acids and derivatives	Hydroxy acids and derivatives	Medium-chain hydroxy acids and derivatives	Medium-chain hydroxy acids and derivatives	4431688.459	4588982.761	5347624.28	5573364.701
Full match	Organic acids and derivatives	Carboxylic acids and derivatives	Amino acids, peptides, and analogues	N-acyl-alpha amino acids	12388209.88	12639972.45	10699095.51	9866831.979
Full match	Lipids and lipid-like molecules	Glycerophospholipids	Glycerophosphoserines	Phosphatidylserines	17296570.28	13228108.96	16473135.15	17159493.19
No match	Lipids and lipid-like molecules	Fatty Acyls	Eicosanoids	Prostaglandins and related compounds	455848.3596	428061.5114	449042.7525	470401.0969
No results					898071.9545	1151813.108	1934527.422	2184199.838
No results	Lipids and lipid-like molecules	Fatty Acyls	Eicosanoids	Prostaglandins and related compounds	283798.1402	412325.0467	302210.2985	519414.6709
No results	Lipids and lipid-like molecules	Fatty Acyls	Fatty acids and conjugates	Medium-chain fatty acids	3290713.371	4340179.891	4054984.851	4674887.913
No results	Lipids and lipid-like molecules	Glycerophospholipids	Glycerophosphoethanolamines	1-acyl-sn-glycero-3-phosphoethanolamines	181311.9829	165868.9677	165307.8237	184028.6411
No match	Lipids and lipid-like molecules	Fatty Acyls	Eicosanoids	Prostaglandins and related compounds	1700288.176	380416.9418	396475.9081	390853.7661
Full match	Organoheterocyclic compounds	Pteridines and derivatives	Pterins and derivatives	Biopterins and derivatives	382613.9917	734755.0557	1656255.47	366426.7967
Full match	Lipids and lipid-like molecules	Glycerophospholipids	Glycerophosphates	Glycerophosphates	7956524.237	6395338.658	5526293.963	4561874.551
Full match	Organoheterocyclic compounds	Indoles and derivatives	Indolyl carboxylic acids and derivatives	Indole-3-acetic acid derivatives	403728.6598	525866.301	728533.5126	496161.1206
Full match	Benzenoids	Phenols	Benzenediols	Resorcinols	386765.8544	346942.8812	361588.7695	340908.8399
No results	Lipids and lipid-like molecules	Glycerophospholipids	Glycerophosphoglycerols	Phosphatidylglycerols	287379.9239	331142.665	322353.4473	323645.0348
No results	Lipids and lipid-like molecules	Glycerophospholipids	Glycerophosphoglycerols	Phosphatidylglycerols	373677.7426	390269.702	456082.6081	325711.8911
No results	Lipids and lipid-like molecules	Glycerophospholipids	Glycerophosphoinositols	Phosphatidylinositols	8473020.172	5461893.301	5145088.372	6050653.699
No results	Lipids and lipid-like molecules	Glycerophospholipids	Glycerophosphocholines	Phosphocholines	1114195.048	1301047.86	1433857.989	1385993.325
Full match	Organoheterocyclic compounds	Lactones	Delta valerolactones	Delta valerolactones	1610662.975	2050168.453	2398124.327	2120543.902
No results					708410.5771	677473.2937	839308.569	2017282.212
No results	Organic oxygen compounds	Organooxygen compounds	Alcohols and polyols	Cyclopentanols	1498610.855	1545391.45	2022709.926	2059474.096
No results					512163.9812	437903.6137	423214.8809	511974.4622
No results	Lipids and lipid-like molecules	Glycerophospholipids	Glycerophosphoinositols	Phosphatidylinositols	3375125.161	2792709.031	2753076.74	3595692.96
Full match	Organic acids and derivatives	Carboxylic acids and derivatives	Amino acids, peptides, and analogues	Gamma amino acids and derivatives	16879254.27	17601112.37	16252761.85	19521220.52
No results					5938159.909	4886670.612	5972772.763	6280383.826
Full match	Organic oxygen compounds	Organooxygen compounds	Carbohydrates and carbohydrate conjugates	Sugar alcohols	4587839.068	3009581.627	2100255.811	2569978.258
Full match	Organic oxygen compounds	Organooxygen compounds	Carbohydrates and carbohydrate conjugates	Sugar acids and derivatives	7707811.27	7996863.55	7688387.568	7788871.773
No results					9788523.504	10187807.74	8612967.622	7375721.019
Full match					633986.5063	619497.0013	1155293.353	343670.7372
Full match	Lipids and lipid-like molecules	Fatty Acyls	Fatty acids and conjugates	Methyl-branched fatty acids	351043.1403	323250.7727	867150.1599	330669.8663
No results					1427921.358	1214295.99	1001746.574	1107637.83
Full match	Benzenoids	Benzene and substituted derivatives	Benzoic acids and derivatives	Gallic acid and derivatives	309740.6549	263883.109	269430.4219	306059.1931
No results					2777302.698	1574705.499	2073468.495	2410075.909
Full match	Organic acids and derivatives	Carboxylic acids and derivatives	Amino acids, peptides, and analogues	Oligopeptides	755354.847	794523.2724	936898.5888	1077229.697
No results	Lipids and lipid-like molecules	Fatty Acyls	Fatty acids and conjugates	Very long-chain fatty acids	430747.8422	405200.6231	419018.7185	437068.0968
No results	Organic acids and derivatives	Carboxylic acids and derivatives	Amino acids, peptides, and analogues	Dipeptides	3454727.306	2597913.1	3777724.241	3377386.394
No results	Phenylpropanoids and polyketides	Phenylpropanoic acids	Not available	Phenylpropanoic acids	968107.0924	892838.7341	970382.7687	1178877.469
Full match					172458.5325	168901.2563	168675.7271	181173.8769
No results	Lipids and lipid-like molecules	Steroids and steroid derivatives	Bile acids, alcohols and derivatives	Monohydroxy bile acids, alcohols and derivatives	249462.9696	162179.7449	252454.0858	254684.4994
Full match					1340595.792	1668738.75	1939062.313	1501493.145
Full match	Organoheterocyclic compounds	Dithiolanes	Lipoamides	Lipoamides	1773862.06	2312483.521	2167630.511	1990033.807
No results	Lipids and lipid-like molecules	Glycerophospholipids	Glycerophosphoglycerols	Phosphatidylglycerols	2645607.438	2420161.132	3913221.788	2268255.176
Full match	Nucleosides, nucleotides, and analogues	Purine nucleosides	Not available	Purine nucleosides	753295.9459	532561.8043	483638.2191	468099.2274
No results	Lipids and lipid-like molecules	Fatty Acyls	Fatty acids and conjugates	Long-chain fatty acids	2340821.804	1822542.714	2306985.596	2065894.95
Full match					1331218.48	1536888.244	1113927.451	2490399.882
Full match	Lipids and lipid-like molecules	Fatty Acyls	Fatty acids and conjugates	Long-chain fatty acids	1103366.372	710294.5897	700064.444	771139.287
Full match	Lipids and lipid-like molecules	Glycerophospholipids	Glycerophosphoserines	Phosphatidylserines	2037401.284	1984749.691	2684415.039	2469782.843
No results	Lipids and lipid-like molecules	Glycerophospholipids	Glycerophosphoserines	Phosphatidylserines	1613577.938	1353963.443	1780962.928	1785284.174
No results	Lipids and lipid-like molecules	Glycerophospholipids	Glycerophosphoserines	Phosphatidylserines	2628587.141	1527814.061	2018362.592	2804021.917
Full match	Nucleosides, nucleotides, and analogues	Pyrimidine nucleotides	Pyrimidine deoxyribonucleotides	Pyrimidine 2'-deoxyribonucleoside monophosphates	23361470.77	20658163.6	17871134.8	17315724.89
No results	Organoheterocyclic compounds	Azoles	Imidazoles	Imidazolyl carboxylic acids and derivatives	7897630.89	8069596.732	8712640.016	9156998.455
No results					297488.1617	286955.9869	288868.8766	314146.4135
Full match					323662.3676	307593.0132	450166.825	325946.7821

MassList_Results	Class_I	Class_II	Class_III	Class_IV	all_YM1_1	all_YM1_2	all_YM1_3	all_YM1_4
Full match	Lipids and lipid-like molecules	Glycerophospholipids	Glycerophosphoethanolamines	1-acyl-sn-glycero-3-phosphoethanolamines	248186.2641	151274.8961	174640.4595	214415.008
No results	Lipids and lipid-like molecules	Glycerophospholipids	Glycerophosphoglycerols	Phosphatidylglycerols	396195.4555	327274.1194	332530.7783	429565.3685
Full match	Organoheterocyclic compounds	Dihydrofurans	Furanones	Butenolides	9353867.73	11787098.73	7553464.622	6865139.868
Full match	Phenylpropanoids and polyketides	Phenylpropanoic acids	Not available	Phenylpropanoic acids	184418.511	186065.2571	197968.3457	215506.5601
Full match					238173.389	215000.5196	241617.722	379376.9785
No results					270857.6538	705888.6356	778710.7499	276070.6416
No results	Lipids and lipid-like molecules	Glycerophospholipids	Glycerophosphoethanolamines	1-acyl-sn-glycero-3-phosphoethanolamines	187901.6762	231799.5747	216119.9033	198333.8455
Full match	Nucleosides, nucleotides, and analogues	Pyrimidine nucleosides	Not available	Pyrimidine nucleosides	1659289.412	1751857.036	1721772.487	1425000.72
Full match	Organic acids and derivatives	Carboxylic acids and derivatives	Amino acids, peptides, and analogues	N-acyl-alpha amino acids	982049.0346	1018541.935	1248468.284	1478871.908
Full match	Nucleosides, nucleotides, and analogues	Pyrimidine nucleosides	Pyrimidine 2'-deoxyribonucleosides	Pyrimidine 2'-deoxyribonucleosides	340283.0128	452405.7805	325474.8521	380453.2234
Full match	Nucleosides, nucleotides, and analogues	Pyrimidine nucleotides	Pyrimidine nucleotide sugars	Pyrimidine nucleotide sugars	752753.845	678737.2554	1081581.502	412154.2888
Full match					1125894.43	1091371.5	1356126.72	1179020.022
Full match	Nucleosides, nucleotides, and analogues	Purine nucleosides	Purine 2'-deoxyribonucleosides	Purine 2'-deoxyribonucleosides	4479312.961	4559094.192	5114352.078	5346874.616
Full match	Nucleosides, nucleotides, and analogues	Purine nucleotides	Purine ribonucleotides	Purine ribonucleoside monophosphates	396480.7576	381198.6024	382777.6057	260514.2183
No results					228440.8543	193854.6337	204792.8914	215322.1381
Full match	Organoheterocyclic compounds	Quinolines and derivatives	Quinolones and derivatives	Hydroquinolones	1449345.905	1307742.394	1263351.557	1483845.794
Full match	Organic oxygen compounds	Organooxygen compounds	Carbohydrates and carbohydrate conjugates	Pentose phosphates	4999265.3	5677590.222	6417320.776	5274500.742
Full match	Nucleosides, nucleotides, and analogues	Imidazole ribonucleosides and ribonucleotides	1-ribosyl-imidazolecarboxamides	1-ribosyl-imidazolecarboxamides	1046147.43	639909.3187	1010846.209	964617.5671
No results	Lipids and lipid-like molecules	Glycerophospholipids	Glycerophosphoserines	Phosphatidylserines	1402234.153	964087.8598	1543325.728	1398076.908
No results					2027728.601	2351345.774	2106977.021	1715270.902
Full match					92283.94983	88135.88631	88141.2188	99867.22287
Full match	Organoheterocyclic compounds	Pyridines and derivatives	Pyridinecarboxylic acids and derivatives	Pyridinecarboxylic acids and derivatives	409332.0676	200439.8041	274139.1486	338547.1611
Full match	Organic acids and derivatives	Carboxylic acids and derivatives	Amino acids, peptides, and analogues	Peptides	365196023	391079713.2	351681245.9	333426905.7
Full match	Organic acids and derivatives	Peptidomimetics	Hybrid peptides	Hybrid peptides	6300325821	5775325435	6795180017	6830254272
Full match	Nucleosides, nucleotides, and analogues	Purine nucleotides	Purine ribonucleotides	Purine ribonucleoside monophosphates	9562475301	9168086570	10809573132	11818848434
Full match	Organic oxygen compounds	Organooxygen compounds	Alcohols and polyols	Secondary alcohols	596304680.2	613035315.8	556658564.5	552858372.9
Full match	Lipids and lipid-like molecules	Fatty Acyls	Fatty acids and conjugates	Long-chain fatty acids	9604510.117	13992711.76	8944497.768	9262935.876
Full match	Organoheterocyclic compounds	Imidazopyrimidines	Purines and purine derivatives	Xanthines	4790923888	4737147356	5302211293	5346325147
Full match	Nucleosides, nucleotides, and analogues	Purine nucleosides	Not available	Purine nucleosides	261948.7231	365356.354	774157.2578	248436.7272
Full match	Lipids and lipid-like molecules	Fatty Acyls	Fatty acids and conjugates	Medium-chain fatty acids	122126689.4	147589905.4	264907115	122651954.8
Full match	Lipids and lipid-like molecules	Fatty Acyls	Fatty acids and conjugates	Very long-chain fatty acids	285934193.4	213993018.9	264441246.2	306059350.8
Full match	Lipids and lipid-like molecules	Fatty Acyls	Fatty acids and conjugates	Long-chain fatty acids	72413849.99	49726665.92	52454605.12	76850549.71
Full match	Lipids and lipid-like molecules	Fatty Acyls	Fatty acids and conjugates	Long-chain fatty acids	686891716.4	561738938.6	677220428.5	701969811.5
Full match	Organoheterocyclic compounds	Imidazopyrimidines	Purines and purine derivatives	Hypoxanthines	5252503740	4817497287	6020787724	6568003143
Full match	Lipids and lipid-like molecules	Fatty Acyls	Fatty acids and conjugates	Hydroxy fatty acids	150759991.8	193924431.9	132517804.2	126589339
Full match	Nucleosides, nucleotides, and analogues	Purine nucleosides	Not available	Purine nucleosides	46168134.71	29874346.03	37284116.4	27457804.81
Full match	Lipids and lipid-like molecules	Fatty Acyls	Lineolic acids and derivatives	Lineolic acids and derivatives	9779306.597	5490140.567	11964819.41	13686342.85
Full match	Lipids and lipid-like molecules	Glycerophospholipids	Glycerophosphoethanolamines	2-acyl-sn-glycero-3-phosphoethanolamines	9061312.738	5618400.382	9535132.302	10475436.16
Full match	Organic acids and derivatives	Carboxylic acids and derivatives	Amino acids, peptides, and analogues	Glutamic acid and derivatives	34934653.38	30939428.31	29728813.53	29942669.46
Full match	Organic acids and derivatives	Carboxylic acids and derivatives	Amino acids, peptides, and analogues	N-acyl-alpha amino acids	84321294.21	81987135.09	100875252.5	118212397.1
Full match	Lipids and lipid-like molecules	Fatty Acyls	Fatty acid esters	Fatty acid esters	4680271.26	5326427.291	5478813.421	5320967.801
Full match	Organic acids and derivatives	Carboxylic acids and derivatives	Amino acids, peptides, and analogues	Phenylalanine and derivatives	45584611.06	35083772.7	30517926.9	39905824.15
Full match	Organic acids and derivatives	Carboxylic acids and derivatives	Tricarboxylic acids and derivatives	Tricarboxylic acids and derivatives	227819234.3	284974710.9	326908933.8	381930279.9
Full match	Lipids and lipid-like molecules	Steroids and steroid derivatives	Bile acids, alcohols and derivatives	Glycinated bile acids and derivatives	98342874.57	77452770.76	93511913.47	62244434.49
No results	Lipids and lipid-like molecules	Glycerophospholipids	Glycerophosphoethanolamines	1-acyl-sn-glycero-3-phosphoethanolamines	9153882.681	8045704.372	10542889.65	10645956.59
Full match	Organic acids and derivatives	Carboxylic acids and derivatives	Amino acids, peptides, and analogues	Alpha amino acids and derivatives	312371202.4	266513379.4	360866538.2	291763299.9
Full match	Lipids and lipid-like molecules	Fatty Acyls	Fatty acids and conjugates	Long-chain fatty acids	1156900707	1083931362	1135369917	1601831475
Full match	Organic acids and derivatives	Carboxylic acids and derivatives	Amino acids, peptides, and analogues	Dipeptides	49981101.94	48778235.2	8505954.049	5842341.309
Full match	Organic acids and derivatives	Carboxylic acids and derivatives	Amino acids, peptides, and analogues	N-acyl-alpha amino acids	49981101.94	48778235.2	33185085.05	5842341.309
Full match	Organic acids and derivatives	Carboxylic acids and derivatives	Amino acids, peptides, and analogues	Aspartic acid and derivatives	4761965.285	3572159.807	5555854.479	5001164.656
Full match	Nucleosides, nucleotides, and analogues	Pyrimidine nucleosides	Not available	Pyrimidine nucleosides	196853990.1	222435415.8	280734576.5	290569734.2
Full match	Nucleosides, nucleotides, and analogues	(5'->5')-dinucleotides	Not available	(5'->5')-dinucleotides	704060182.4	593346214	459858982.1	413727638.9
No results	Lipids and lipid-like molecules	Glycerophospholipids	Glycerophosphocholines	Phosphocholines	45955644.33	42283700.45	56023675.16	68716427.62

MassList_Results	Class_I	Class_II	Class_III	Class_IV	all_YM1_1	all_YM1_2	all_YM1_3	all_YM1_4
Full match					1569493.262	361324.7403	550526.344	457782.9296
No results	Lipids and lipid-like molecules	Fatty Acyls	Fatty acids and conjugates	Long-chain fatty acids	18592803.71	10198873.97	21447896.34	8778496.038
No results					12324702.69	9879169.418	9723692.929	12309197.23
Full match	Organic acids and derivatives	Carboxylic acids and derivatives	Amino acids, peptides, and analogues	Alpha amino acids	5077552.958	19537563.88	26533172.26	16008234.67
No results	Lipids and lipid-like molecules	Fatty Acyls	Fatty acids and conjugates	Very long-chain fatty acids	261968623.5	180714014.4	225759847.2	264075040.8
Full match	Organic acids and derivatives	Carboxylic acids and derivatives	Amino acids, peptides, and analogues	N-acyl-L-alpha-amino acids	5662356.187	11648373.05	13451338.65	6340895.625
No results	Lipids and lipid-like molecules	Glycerophospholipids	Glycerophosphocholines	Phosphocholines	677278739.5	440535377	566013706.1	607873045.7
Full match	Lipids and lipid-like molecules	Fatty Acyls	Fatty acids and conjugates	Long-chain fatty acids	1395573.688	747746.6779	968062.4107	1190573.795
Full match	Organoheterocyclic compounds	Dithiolanes	Lipoic acids and derivatives	Lipoic acids and derivatives	26247929.51	23455082.9	18768180.66	23184935.71
Full match					35154034.61	24106048.06	33095842.28	30063934.65
Full match	Organic oxygen compounds	Organooxygen compounds	Carbohydrates and carbohydrate conjugates	N-acylneuraminic acids	20112605.76	38335445.81	22207862.92	19968412.85
Full match					239650.8577	242010.1332	231734.0041	249453.9093
Full match	Nucleosides, nucleotides, and analogues	Purine nucleotides	Purine ribonucleotides	Purine ribonucleoside monophosphates	37361456.25	101910711.3	84187135.4	16528252.14
Full match	Organic nitrogen compounds	Organonitrogen compounds	Amines	2-arylethylamines	58977123.93	49889392.89	56946469.88	67002970.86
Full match	Lipids and lipid-like molecules	Fatty Acyls	Fatty acids and conjugates	Long-chain fatty acids	230510683.9	187814538.1	198231256.9	243552604.6
Full match	Benzenoids	Benzene and substituted derivatives	Benzoic acids and derivatives	Benzoic acid esters	113243198.8	109163068	112592914.1	127988681.1
No results	Lipids and lipid-like molecules	Glycerophospholipids	Glycerophosphocholines	Phosphocholines	4732584.493	3439372.588	4633008.818	6345915.432
Full match	Lipids and lipid-like molecules	Fatty Acyls	Eicosanoids	Prostaglandins and related compounds	2583835.396	2216669.826	2946170.638	3456225.23
Full match	Lipids and lipid-like molecules	Glycerophospholipids	Glycerophosphoethanolamines	1-acyl-sn-glycero-3-phosphoethanolamines	8695515.917	6858219.555	7103168.17	8594995.221
No match	Organoheterocyclic compounds	Imidazopyrimidines	Purines and purine derivatives	Purines and purine derivatives	16997310.85	15482036.98	11746444.98	17263039.36
Full match	Benzenoids	Benzene and substituted derivatives	Benzoic acids and derivatives	Hippuric acids	37359941.7	44670154.26	41596176.45	43936691.89
Full match	Lipids and lipid-like molecules	Glycerophospholipids	Glycerophosphoethanolamines	1-acyl-sn-glycero-3-phosphoethanolamines	7526853.122	6112532.367	5458137.687	8632925.701
Full match	Lipids and lipid-like molecules	Glycerophospholipids	Glycerophosphoethanolamines	Glycero-3-phospho-N-acyl-ethanolamines	47417191.25	32536461.16	39100999.99	42995518.35
Full match	Organic acids and derivatives	Carboxylic acids and derivatives	Amino acids, peptides, and analogues	L-alpha-amino acids	12216024.52	10794079.73	11525028.41	11644182.33
No results	Lipids and lipid-like molecules	Glycerophospholipids	Glycerophosphocholines	Phosphocholines	8782034.917	7054150.252	8985908.605	9858498.647
Full match	Organic acids and derivatives	Carboxylic acids and derivatives	Amino acids, peptides, and analogues	Proline and derivatives	54103137.68	56492193.25	51612414.78	61884091.51
Full match	Lipids and lipid-like molecules	Steroids and steroid derivatives	Bile acids, alcohols and derivatives	Glycinated bile acids and derivatives	21930210.15	16150344.36	16892222.28	13089001.49
Full match	Organic acids and derivatives	Carboxylic acids and derivatives	Amino acids, peptides, and analogues	Dipeptides	2738191.631	1765749.213	3102529.969	2255102.741
Full match	Lipids and lipid-like molecules	Glycerophospholipids	Glycerophosphoethanolamines	2-acyl-sn-glycero-3-phosphoethanolamines	25207345.42	22006058.44	29317953.12	34706454.58
No results					23592914.4	19709238.47	20171675.22	23886832.05
No results	Lipids and lipid-like molecules	Glycerophospholipids	Glycerophosphoethanolamines	1-acyl-sn-glycero-3-phosphoethanolamines	2879160.594	2316004.556	2608846.127	3000932.121
Full match	Nucleosides, nucleotides, and analogues	Pyrimidine nucleotides	Pyrimidine deoxyribonucleotides	Pyrimidine 2'-deoxyribonucleoside monophosphates	11424188.39	10836633.43	10417402.89	10449826.05
Full match	Nucleosides, nucleotides, and analogues	Purine nucleotides	Purine ribonucleotides	Purine ribonucleoside diphosphates	117636628.7	38168926.89	47419358.23	92072869.9
Full match	Organoheterocyclic compounds	Indoles and derivatives	Indolyl carboxylic acids and derivatives	Indolyl carboxylic acids and derivatives	14621182.14	18908129.76	17904561.2	16715327.51
Full match	Lipids and lipid-like molecules	Glycerophospholipids	Glycerophosphocholines	Phosphocholines	2203576.912	2157532.579	1040409.741	1237744.584
No results	Lipids and lipid-like molecules	Glycerophospholipids	Glycerophosphoethanolamines	1-acyl-sn-glycero-3-phosphoethanolamines	2040519.085	1595027.331	1863710.083	1959269.152
Full match	Organic acids and derivatives	Carboxylic acids and derivatives	Amino acids, peptides, and analogues	Methionine and derivatives	117148731	116528216.1	147438795.8	123327846.1
Full match	Organoheterocyclic compounds	Indoles and derivatives	Tryptamines and derivatives	Serotonins	41494.10514	40196.59705	39653.31915	41082.48574
Full match	Lipids and lipid-like molecules	Fatty Acyls	Eicosanoids	Hydroperoxyeicosatetraenoic acids	15503100.91	7172126.058	8199759.049	10980840.85
Full match	Organoheterocyclic compounds	Imidazopyrimidines	Purines and purine derivatives	Xanthines	1248192.7	1665008.95	1638964.63	1484573.863
Full match	Lipids and lipid-like molecules	Fatty Acyls	Fatty acid esters	Acyl carnitines	43690435.39	36196704.79	31343548.48	32606868.77
Full match	Organic acids and derivatives	Carboxylic acids and derivatives	Amino acids, peptides, and analogues	Asparagine and derivatives	11780552.27	10917906.54	12370597.18	9116289.419
Full match					2879058.103	2064949.699	2925923.948	2992537.535
Full match	Organic acids and derivatives	Carboxylic acids and derivatives	Amino acids, peptides, and analogues	Phenylalanine and derivatives	476859163.7	416534432.5	1163230.934	442271960.8
No results					696577.6799	975530.7233	1168812.365	2284798.064
Full match	Lipids and lipid-like molecules	Fatty Acyls	Eicosanoids	Prostaglandins and related compounds	764628.493	763658.7118	755881.771	827460.5093
Full match					1994673.795	4213865.79	11249647.21	13101727.11
Full match	Organic acids and derivatives	Carboxylic acids and derivatives	Amino acids, peptides, and analogues	Tyrosine and derivatives	189173.6266	203863.7607	188758.0774	183867.9387
Full match	Nucleosides, nucleotides, and analogues	Pyrimidine nucleosides	Not available	Pyrimidine nucleosides	3644187.485	3899858.864	4260934.659	4158159.166
Full match	Lipids and lipid-like molecules	Fatty Acyls	Fatty acids and conjugates	Medium-chain fatty acids	10496192.43	12070970.6	11884722.47	12002977.08
No results	Lipids and lipid-like molecules	Fatty Acyls	Eicosanoids	Prostaglandins and related compounds	686430.4506	566528.0371	800166.8337	464256.3936
No results	Lipids and lipid-like molecules	Glycerophospholipids	Glycerophosphoethanolamines	1-acyl-sn-glycero-3-phosphoethanolamines	44044471.36	40310627.26	45425140.77	51436535.92
Full match					9056320.995	8921356.671	9380489.085	11796530.47

MassList_Results	Class_I	Class_II	Class_III	Class_IV	all_YM1_1	all_YM1_2	all_YM1_3	all_YM1_4
Full match	Lipids and lipid-like molecules	Fatty Acyls	Lineolic acids and derivatives	Lineolic acids and derivatives	1337566.716	840304.3133	1333212.557	1465936.155
No results	Lipids and lipid-like molecules	Glycerophospholipids	Glycerophosphocholines	Phosphocholines	6499841.113	5309570.177	8390711.327	9964890.344
No results	Lipids and lipid-like molecules	Glycerophospholipids	Glycerophosphocholines	Phosphocholines	1498703.62	1299309.522	1707552.34	2084497.869
Full match	Organic oxygen compounds	Organooxygen compounds	Carbohydrates and carbohydrate conjugates	Oligosaccharides	19985759.46	2845202.302	4732541.321	966788.2438
No results	Lipids and lipid-like molecules	Glycerophospholipids	Glycerophosphocholines	Phosphocholines	2202052.408	2145859.672	3038083.904	4233386.495
Full match	Lipids and lipid-like molecules	Steroids and steroid derivatives	Bile acids, alcohols and derivatives	Dihydroxy bile acids, alcohols and derivatives	1739105.167	1985079.821	2632585.835	2922960.742
Full match	Lipids and lipid-like molecules	Glycerophospholipids	Glycerophosphocholines	1-acyl-sn-glycero-3-phosphocholines	1296491.142	888278.8312	1011081.008	1081339.12
Full match	Lipids and lipid-like molecules	Glycerophospholipids	Glycerophosphoethanolamines	1-acyl-sn-glycero-3-phosphoethanolamines	2862451.413	2228320.616	2993445.002	2961434.019
Full match	Organoheterocyclic compounds	Quinolines and derivatives	Quinoline carboxylic acids	Quinoline carboxylic acids	1431589.073	933810.5097	1063871.131	1325572.913
Full match	Organic acids and derivatives	Carboxylic acids and derivatives	Amino acids, peptides, and analogues	Dipeptides	4187754.691	2839831.04	4109164.414	5381048.733
Full match	Nucleosides, nucleotides, and analogues	Pyrimidine nucleotides	Pyrimidine deoxyribonucleotides	Pyrimidine 2'-deoxyribonucleoside monophosphates	3160380.089	1477733.267	1515038.009	1402546.198
No results	Organic oxygen compounds	Organooxygen compounds	Carbohydrates and carbohydrate conjugates	Pentose phosphates	291686.424	301554.3002	328672.7146	306764.6873
Full match	Organic acids and derivatives	Organic phosphoric acids and derivatives	Phosphate esters	Monoalkyl phosphates	10884000.83	8862461.641	12924918.43	11219466.61
No results	Lipids and lipid-like molecules	Glycerophospholipids	Glycerophosphoethanolamines	1-acyl-sn-glycero-3-phosphoethanolamines	2195408.695	1910031.462	2496625.052	2458275.083
No results	Lipids and lipid-like molecules	Fatty Acyls	Fatty acids and conjugates	Long-chain fatty acids	482455.2287	460706.6615	497362.3109	516270.3642
Full match	Organic oxygen compounds	Organooxygen compounds	Carbohydrates and carbohydrate conjugates	Hexose phosphates	468608.4431	880408.5569	1152093.53	933313.6179
Full match	Lipids and lipid-like molecules	Fatty Acyls	Fatty acids and conjugates	Methyl-branched fatty acids	45596853.12	54654701.82	58908364.3	45730034.13
Full match					16084959.71	16122956.46	26075301.12	24034621.25
Full match	Organoheterocyclic compounds	Pyridines and derivatives	Pyridoxines	Pyridoxines	1354951.534	579194.8493	1035076.866	1440718.809
No results					166090.8448	153984.2598	159128.8495	170813.6054
Full match	Organic oxygen compounds	Organooxygen compounds	Carbohydrates and carbohydrate conjugates	Gluconolactones	828389.3071	903750.2408	591124.4625	578061.2954
Full match	Organic acids and derivatives	Carboxylic acids and derivatives	Tricarboxylic acids and derivatives	Tricarboxylic acids and derivatives	475774.573	448148.2014	465617.5222	510032.0021
Full match	Organoheterocyclic compounds	Indoles and derivatives	Indolyl carboxylic acids and derivatives	Indolyl carboxylic acids and derivatives	125368.6628	123084.6682	136681.9585	136047.9992
Full match	Organic acids and derivatives	Carboxylic acids and derivatives	Amino acids, peptides, and analogues	Dipeptides	6089754.354	5794556.327	5329601.738	6094366.108
No results					844206.314	2781789.431	2070449.064	756543.1508

all_YM1_5	all_YM2_1	all_YM2_2	all_YM2_3	all_YM2_4	all_YM2_5	all_YM3_1	all_YM3_2	all_YM3_3	all_YM3_4	all_YM3_5	all_YP1_1	all_YP1_2
26663711281	34816575491	35939787467	34415676354	33269760896	32873726427	34896814955	32031117545	33972175955	31881897316	31828257503	145299235.3	1038166108
27319350381	30357783426	23918867831	22178869803	27228049632	25603324530	20837518274	27658959821	26934233926	21011284366	22554188384	29086624175	55747807718
6839535659	8852776668	5785984930	5478221545	6359709345	6192813368	5550243395	7414853568	7368895129	5126500436	6716584815	7235231913	15774174293
7434985784	9634672750	11038454108	9462486814	5606746288	4146562896	4222775802	5097042042	5762069876	5030831697	5214707169	13746401384	12846139002
5265433376	6160699609	5756071233	5008142265	5708597791	5665837953	6916335688	6544059791	6751943304	6913469339	6572262187	11742637419	11049391960
4975076627	6240170893	4961649041	3812401734	4819484131	3607038818	3905437301	4530659554	5591524261	3355264186	4081768462	6665793025	15039793083
2918436.057	2511691.788	3154573.095	2770587.832	3038935.086	3136985.967	1658835.299	2883210.864	2292486.127	2510330.422	2386540.439	12866132.39	12662622.26
2928367290	5010324838	4318333268	3114579730	3291272587	2882836429	2573076310	3299691258	3856254881	1872320907	2661815909	4916284276	8629600112
143209842.7	425490966.3	238680350.7	202469994.6	292812526.8	71874756.44	199894047.3	204237005.7	272842264.4	267339138.8	91635097.4	920236860.7	547001596.4
1522632349	526234192.6	545930904	675583782.1	724017071	1080239838	116203306	558332466.1	416516715.5	529478469.4	745727263.3	2857610627	1526178583
91228145.24	115698346.4	116113669.3	136534542.1	79383207.73	56506927.45	89718047.94	119115360.4	115999942.2	115629396.4	93564902.22	74424135.49	113278868.7
1131766521	435537139.4	565026481.8	787245443.3	1256209595	1446592854	385870049.6	753994230	819635176.2	928596658.2	1367041672	942395562.4	1136365165
1472525576	343792892.1	547104066.5	907450037.7	1644398725	1856467381	248902684.9	818945986.7	765456995.3	894040135.9	1586275204	944683272.1	752468450.1
764159965.1	1004432344	1135573299	1089418415	3152790041	1882721605	1434670058	1514078108	1489081375	1562770340	1583618054	29438103.28	9696014.577
525635395.1	509052993.4	635033611	471138466.9	463619270.3	339632945.5	574606874.8	522068077.4	733723683	536362963.7	343930432.2	461252660.6	536933205.2
105434644.1	225261074.4	65404874.45	142586769.4	110071462.5	56440085	93551165.57	133902363.9	106437289.7	168794917	100651827.4	382267374.8	117265019.3
491334921.9	342133133.8	298677276.6	326073012.1	308673916.6	277211066.9	938384403.7	368360505.3	455318855.4	433734870.2	314237553.4	32978880.5	19677402.98
2825190933	3605698717	5920215024	5626170175	5264951861	3624843391	1498459769	3290256405	1470196900	1647718686	1718750260	26455252405	14272850198
2667690336	2363945161	2482781642	2594111729	2600066843	2547071680	1832178734	1945381415	1996405616	2415127430	2732951989	5326329998	3103175452
173340.3211	3549603.463	3922368.173	24070987.92	168656.3631	169633.5144	8957651.284	8280466.163	5685289.213	2215120.421	156274.188	28055112.78	17205501.5
558211832.1	687782285.2	281481676.5	657661033.4	610913385.3	582636265.9	1266185721	314063750.8	265546952	293942322	369599925.4	1011775725	1099114662
537189091.7	431808311.7	528357832.9	544141828.1	432870139.3	565684466.4	632918537.5	644124643.8	615023231.7	620896221.1	796390453.5	444429568.3	955391224.1
499882846.6	1647303.445	165186356	246987687.8	435305114.1	464383388.7	68661706.56	186541395.4	4346886.926	261479956.4	339037926.4	522112903.7	410756507.2
557302231.8	724672952.8	799808279.4	928349810.4	1563904173	926904926.4	752413793.8	919517916.9	874480119.9	943199834.9	963886756.6	135358984.1	109256591.6
333943905.7	46704514.54	132861154.1	171147727.8	352302362.8	282056649.2	29517317.58	95291900.04	110609002.2	153806978.8	205672363.5	829006983.7	694825523.3
407799985.1	354822896.9	461958117.1	405448686.3	427623870.4	387619910.8	336617547.2	471757468.4	425935768.1	327486370.2	282183744.8	1146969395	1358215597
671181992.4	1239796779	1240357666	1214882367	1202452846	697386502.9	1051484680	1170978229	627168091.1	1187533748	1170301900	5212160.573	10047905.86
303476000.9	224135647.5	215003683.7	179839666.3	300475536.3	213783651.7	197470265	239344904.7	297409610.5	217993705	233640014	285201645.3	673753643.2
258916319.5	259273884.5	248965063.5	263367094.2	232058840.8	261159131.5	234658241.2	256149624	306084129.5	301631510.2	284837018.1	7503983438	5971545077
43436836.67	77339509.74	58786298.87	41814066.37	50652270.73	39589058.39	37544903.41	50904298.93	56546028.09	46009847	39958685.04	61430506.68	236457792.2
296989438.7	354020069.3	278431114	311249984.7	281304202.7	268498906.1	307070720.5	336861297.2	354210498	383830438.6	277701358.9	263090314.6	239654923.5
28019057.6	38015518.63	31299495.85	28019362.93	33371444.22	14589263.68	38947016.19	28805415.09	42056185.83	42817680.19	31293423.82	5757301.985	3177398.189
64883281.78	48660246.28	32817441.84	33113709.73	48143871.56	45589148.11	67047655.37	59296349.24	69394336.69	94899077.55	135449065.3	116032070.1	137563980.7
81713067.21	70581776.32	58428368.4	62439136.74	68665911.52	48862652.85	213099691.9	87339618.1	97192551.01	83750701.67	79896342.92	227991813.5	218632917.9
293075976.2	282402545.9	236510241.2	228516806.8	278580507.8	236174172	163431517.1	285114537.9	233647514.6	259570756.9	279583266.9	432329938.1	519216671.9
1462929853	1258543098	1337612678	1402495850	1292197498	1805839753	2277491488	1985213889	2029163944	1536239884	1417080910	7034718393	6813390179
26795342.73	31390090.98	33681699.85	35979348.52	23773364.66	16369861.42	25176915.31	36111752.72	32287043.71	31585761.33	24370066.95	19557567.26	21851628.43
193269029.7	289849674	365156594.7	320441183.1	290229411	329438410.5	182672766	193878594.3	165345673.8	198132328.6	200222412.5	236433595.4	163680235.8
86184791.87	99691440.05	124994344.7	114555058.3	289393197.9	193020516.2	129194037	165290883.5	147805264.8	166473176.2	176346600.5	408291198.4	465307916.5
255780523.5	250469118.4	324083520.1	865921351.5	598296327.5	954946252.8	1060681571	1230417467	1134894577	230598829	1016887206	258384794.1	273159918.4
205273587.7	90587958.99	81113812.9	115047030.5	173327784.5	209327363.8	15255029.99	62079804.27	42279570.9	77718228.97	91457788.77	145485720.7	188111191.1
594893278.1	738984615.5	528809009.5	981991443.3	360444154.7	680404677.2	1092921930	831447737.3	769992860.3	961133089.9	743803755.7	2627842563	2371509609
2270450.764	4475465.365	6045579.366	6470289.758	14809270.48	14155177.2	10159256.76	10671387.21	2353307.37	8206959.232	14269694.63	37008522.35	47753483.36
19945331.85	39682271.13	31263021.25	30409859.28	24263598.19	16251187.51	30366449.34	30949061.34	35731058.62	17930512.57	15006906.92	25833805.63	34973244.59
127926303.8	22922658.35	34739237.31	65263587.45	92798412.63	85056352.14	4337333.646	27053018.57	19096731.8	26378311.48	43124256.65	278237433.2	78279055.85
127800907.8	156891197.7	131692238.7	143758915.3	117546688	119622108.5	171456489.4	134781954.9	145575762.5	150134187.8	108053400.6	128333032.5	109678900.4
67064317.76	145088175	103637600.6	150805034.1	105929452.7	73038558.51	118274760.5	107303247.9	126488685.9	66881276.09	63162796.82	51875732.4	50080065.26
92577620.46	131867022.7	129969159.2	311081987.2	222768113.5	158959850.9	160351041.4	182596513.2	172082204.3	215991008	196524565.4	1241930.701	1092091.754
93599511.87	117932154.5	88888072.5	118495277.4	104803765.9	124558407.9	96944853.78	123709692.8	127232680.1	97786361.9	97485821.23	244589374.3	435312692.4
64832987.08	72629264.7	139863414.7	85228708.08	91138538.29	86366105.44	97009326.27	124398449.7	119983457.3	57488331.41	211733679.7	1223364.469	1181580.572
61193716.92	82570519.15	68259526.06	52672882.12	64986921.64	61574636.62	59559677.69	74241525.8	89130459.51	77325145.02	71202295.52	48135148.9	51866036.88

all_YM1_5	all_YM2_1	all_YM2_2	all_YM2_3	all_YM2_4	all_YM2_5	all_YM3_1	all_YM3_2	all_YM3_3	all_YM3_4	all_YM3_5	all_YP1_1	all_YP1_2
45382941.79	32229217.3	22619663.23	30241300.85	30637685.12	26639648.27	95451154.92	40239867.52	39433605.61	40213910.85	36511619.73	3476450.611	17363325.78
29159339.1	12529866.57	16200777.79	14098490.29	24093328.56	23922342.35	8083371.526	47129020.9	31855877.41	22347326.68	36821646.09	163307982.8	58009681.56
46253667.56	36757242.41	35518468.17	39116326.78	37444887.28	40771165.31	41542351.24	45708576.42	43198709.54	50096597.28	48324037.75	28237859.4	21881177.85
209063941.1	305395680.4	364629816.9	246939270.5	342094121.3	264379765.4	233733025.1	242609829.9	262653822.9	235550049.1	232274030.1	24639285.64	20622555.41
1862839.094	674124.2529	1279752.571	833175.9987	999914.7873	2202885.997	743465.975	2325442.551	1596291.084	2292041.586	1997277.66	94437723.02	96515036.73
255904075	278621637.5	255430959.4	274975322.8	293287658.3	281183416.3	317477310.1	338051813.5	288332637.4	369590967.9	330809934	7464377.821	6325710.575
38957988.04	48954572.35	48176140.08	45012594.07	42015841.77	47776174.66	94539725.84	63640825.17	64017729.55	64237860.99	70581074.9	58901322.86	66022161.7
59693399.08	29624448.94	39371775.82	56708043.37	95682783.87	99614799.61	14347508.56	44291947.83	42442744.28	40650983.83	93929106.22	80193997.17	88242320.68
56311843.31	102709618	112209877.3	92355512.56	53297653.48	67564905.06	59357048.02	54589645.82	82971286.28	69096402.79	54920859.7	115412893	328297349.2
39939203.53	59162847.45	43926432.14	47036987.28	49070793.13	45243347.21	276319391.8	126271478.1	135447768.1	104194805.1	107332750.3	66722828.09	151804986.6
43695448.57	118846792.4	84880757.58	85483975.53	61445805.66	43956462.94	76670244.37	80137882.97	91122787.97	48874783.19	36415072.67	1601648.654	766003.1861
72246611.41	85267930.48	92703036.02	96899014.14	77138572.21	84310374.25	63716153.32	84504718.82	78110964.8	79318518.95	66457652.46	169643075.6	77175162.2
3015851.983	10152754.84	8069262.935	5398167.746	5726470.889	4683701.739	6794684.62	7701112.289	8849612.578	8066403.553	6027906.55	4183154.946	8918691.806
103623446.1	20444438.96	23858543.08	41962058.31	73994540.16	81359054.29	33289409.62	48286054.41	56642872.14	57086208.01	73040537.65	818242287.2	977414923.4
14436634.12	19283932.01	23685031.47	18801212.72	16495482.32	5637205.646	20405749.34	19737244.1	23438565.29	17451821.75	12490857.79	1247263.028	3514438.585
348087.638	369580.9821	376138.2897	371899.0875	371623.6664	359797.9128	344027.7935	347037.6887	369374.6459	299187.9077	339648.3154	915251.7248	997003.968
10100969.09	7540460.094	5190364.94	5606400.835	13266125.92	8564283.207	5002840.166	11867333.77	7493846.18	12285161.36	15212377.95	8054222.238	4103295.983
33118894.04	41484675.22	48870252.27	59124041.2	52944408.05	37530805.3	45930272.76	44172765.85	44678935.86	49273581.49	62501185.02	10017660.99	7239742.493
27717467.85	32806194.68	21765108.22	30844498.26	30684997.29	16943054.59	15066675.82	18504304.08	29431307.73	26229971.47	21524095.7	2576495.505	1776125.21
25617171.9	29166253.38	25784416.68	23502916.16	22781736.31	16061301.66	31005391.15	35497310.83	37009193.17	26485960.42	20384802.2	20516642.51	26329864.96
4888930.645	80730145.31	72895368.59	50717646.33	57539667.59	48262995.39	35689644.97	63653744.95	58269739.14	3883301.5	59115059.88	12044582.93	16628516.02
82358265.58	33298273.06	81073319.41	78888979.64	76608715.99	45193817.6	29539062.4	82615644.01	32959121.25	43643126.38	19929170.31	35976259.77	26371270.83
28019057.6	75993021.18	49483085.02	43027944.42	58422462	14190437.65	84901248.38	39511382.55	78397014.9	61494003.87	26545089.54	100663089.7	56754967.56
63914032.34	93671396.19	86217386.9	68734282.94	77884503.05	76669650.52	72333988.95	86846352	101765289.3	92705669.71	94644844.02	542091326.5	485396695
145411854.6	134387370.2	126015750.9	149058106.5	246819174.8	185363302.4	197505169.6	159469756.4	165456492.9	387837497.6	146356322.4	664097873	650162884.4
90700492.14	53200227.8	58640247.96	53986168.9	86779516.66	93078530.02	38637806.74	64223249.24	27617562.8	38973633.92	30420249.17	25939676.67	35470165.2
13753373.06	5261037.735	5580267.779	4426205.74	14163603.67	4748793.836	7921335.055	11004483.61	19330875.87	17673339.86	4375053.701	30442101.6	38306494.7
33097848.66	75650903.09	50546840.13	37667379.7	50454800.34	57050825.39	47686300.29	41024539.51	57356780.99	35866143.2	37217112.91	40194232.59	66974871.19
59900163.53	63589447.48	67704907.49	55020458.17	62130087.45	73652091.66	76474091.57	73114004.31	65018806.79	69305026.77	69114747.18	17389294.5	17763323.64
39154299.25	25840189.3	35787078.74	39546749.22	29587318.57	39813470.14	30932210.7	39507504.04	33516322.5	46223036.66	59876169.08	362222004.2	165014784.3
64841489.5	27440074.75	41559612.93	50496532.22	70732257.04	69125804.52	5795765.983	29044248.38	29790710.24	34620288.27	29711111.96	63594664.94	80116059.77
44985997.97	57470048	62255465.07	60625216.53	104449353.6	90698945.66	66375731.7	80545624.51	70445610.32	74264325.48	74157787.4	64160826.86	60998324.12
12172432.94	22447167.31	24988304.93	21182345.19	22345364.81	21552870.97	17951028.38	22211967.69	18459615.15	27349340.82	30489869.57	1950510.027	2461210.7
27169338.24	30929753.45	43586735.2	32527377.97	46743581.17	51841513.42	34851642.81	46594227.45	48008787.2	39929927.24	60913824.4	118243808.7	116634047.9
27010728.1	14530095.16	28186926.92	27535183.26	22705596.84	26044300.36	2141804.975	5982079.627	5481218.227	5099203.822	6986580.712	170386501	75838301.12
42728930.17	46820067.47	108627542.5	61513166.29	73389320.64	50695813.56	59929305.47	60600480	61659838.08	80888735	55134352.63	177698017.4	263011742.2
56016847.48	60684628.75	82150445.63	45249794.2	56096631.93	49391279.62	44448439.33	54204521.5	60241219.68	42353381.81	48083166.99	47813322105	84388859413
509279.9496	529579.6369	951273.8058	668774.8105	482261.1375	486035.9345	622600.4326	691911.4931	525334.4031	524620.971	394397.7032	8084454.641	3243355.169
5867358.827	4739853.191	4922427.679	4306178.231	4400370.985	3168817.666	5751550.964	7987273.89	7062929.886	8157352.3	5949476.373	3427780.594	5251506.911
110099022	54611794.94	87049971.63	59912151.25	74114900.67	73638407.69	67278205.97	81171719.27	85885578.25	93986416.13	107883105.6	5673449.491	13971623.05
47513614.39	62284218.7	56513615.7	61118572.62	60802816.66	51594858.79	55994815.09	51706510.69	53991277.47	41011783.14	54118615.6	52841277.77	73965885.39
31183066.84	45036999.28	39648728.74	46145841.61	38101770.79	38086283.98	35203418.74	41201007.2	47953856.21	48999712.96	40508826.69	17623678.63	13664539.31
246302947.4	240948617.8	235155367.3	256442165.2	261711273.3	477166684.2	359558402.5	342692926	64806167.99	345123801.8	363823100.2	548889777.7	498600805.2
28753363.57	31486795.36	25994189.56	31237334.89	30331586.26	32872949.01	24120963.72	31622081.69	30933854.95	37459197.12	33823778.42	25225849.32	31438951.59
161485625.4	151773025.5	137569857.1	135503729.7	142966985.5	154209828.5	178988125.8	174759100.8	176798795.8	183020925.9	176584841.9	68889037.01	70451301.39
22228287.29	22052057.09	32285819.24	44737551.9	14357543.72	14243537.96	20686851	36951083.54	25675661.66	32342096.88	12081352.68	456173.2122	3919641.518
17501841.68	12052159.5	15928440.56	13588705.09	10577072.52	9069052.21	12959022.82	14508682.97	16294952.36	21157192.53	21210917.45	47821326.88	44922916.92
145066194.4	189731340	167503563.6	60445249.85	55506550.25	49945959.38	88368540.15	191145299.8	128613339.5	133296211.1	165027296.7	11148530513	9987192184
64741433.64	84186089.56	87959432.59	86072676.57	76544460.74	88622305.86	113512065.3	100599799.2	95828422.36	109638663.9	110994017	141811154	125300707.9
39974085.19	49918928.98	61217760.29	49882817.85	98848300.43	72954708.24	55272064.46	72385571.57	68225363.32	70881959.52	78361716.49	739868438.5	670351247
38457594.84	14808997.65	42374483.74	57390727.98	47559643.06	30979641.69	1521742.621	23390558.32	12619772.17	19606556.85	16320073.55	33978333.94	6016197.856
16199556.48	18880763.29	20119742.98	18701333	15338785.98	21188695.82	12801264.6	15971197.17	4990726.092	13785919.36	14788656.69	67159634.35	42156780.6
15388076.17	23438738.05	15891019.58	19263604.37	15700434.59	9793145.738	17679747.39	21396923.65	19333715.56	19873636.23	15481569.25	15208203.19	8448804.094

all_YM1_5	all_YM2_1	all_YM2_2	all_YM2_3	all_YM2_4	all_YM2_5	all_YM3_1	all_YM3_2	all_YM3_3	all_YM3_4	all_YM3_5	all_YP1_1	all_YP1_2
2297165.657	3121643.033	1371515.764	1583150.797	1831119.825	1295170.008	28801471.53	9809914.748	11306504.43	8571162.619	4278943.379	2720371.507	2207989.786
1978512.376	4641644.243	4234663.757	2935565.67	1963040.11	420887.2584	1870210.702	2805167.011	2490029.479	1320113.35	1256788.34	5030277.939	12445320.49
1803022.391	2116047.108	2002539.289	1833731.446	1867508.674	1695377.517	1575883.301	1789866.398	2035940.864	1498398.943	1783448.063	3327784.205	2147248.585
4221177.461	7457736.262	6568379.218	6094171.372	5170258.346	2606997.498	6579360.37	6815631.335	8823848.097	4107603.853	3181602.109	5721758.963	6904159.79
13035547.84	18782428.53	21983949.06	18514369.51	48633805.48	30103900.04	23809665.22	30918907.23	28797809	27575451.62	30466894.61	3927154.815	4040477.656
33517872.12	5003220.842	10540982.13	15626378.45	30124869.98	35464374.22	2836625.49	12713409.59	12006741.39	15853629.69	27523836.04	36190654.62	32995201.82
1296923.034	1142361.036	2609956.649	1854961.217	11805038.94	6153001.459	2175519.216	3581940.836	3879967.221	3927797.374	4830801.752	1089659.855	1684851.414
105837380.7	138457117.3	108788603.7	187178364.3	172557514.1	102119055.9	246083264.6	161785800	159171168.1	171820110.3	161505364.3	274169211.5	314204084.8
9861570.126	5517845.156	5701369.135	6352097.23	5668901	4765681.672	32764496.34	11113266.67	13047190.78	14080529.44	11928859.84	26441719.14	44332389.56
45358446.54	52642955.64	53031869.32	46319760.8	53417172.29	59542119.02	37587373.95	39831107.22	47764609.08	45009036.65	39571710.46	70234153.48	68577299.95
34002366.78	21618985.2	21420185.43	21928261.76	22433447.7	27564059.95	14013369.4	24604553.45	21386027.06	25242973.85	27460973.6	315112264.1	236134495.3
130444718.6	108327598	107789374.8	116330385.5	109661690.4	109988621.7	95344610.85	102525156.2	97468430.56	118980656.7	99217713.8	57368997.39	51237496.16
29261568.57	12429273.21	22635636.16	23200814.27	26192773.56	23886893.02	10345084.55	25433910.87	8695413.672	11594630.04	6302830.557	49250901.21	39252418.87
20263410.5	14191203.76	13063783.77	12885225.53	9100865.57	11789998.84	16159119.23	9970207.442	13177787.23	10189206.7	9403394.728	22763922.94	24481400.87
45043082.88	98751859.62	104062770.9	73906269.06	463223161.7	91262904.24	99715539.9	72957150.77	72752993.84	76262352.4	76260083.93	616643451.8	560225718.7
136794318	134305079.8	146909466.3	128017800.7	128810819.6	132336007.6	119500250.6	139308234.4	128696333.9	120088343.7	128402170	5005626.914	45412369.15
42598078.41	28776121.23	29159833.84	29254687.8	38037781.84	28754788.01	14913520.03	21605897.05	19047825.67	24719331.41	13381650.14	77436270.9	44441599.69
6963052.39	11485335.11	13721363.01	10953932.63	23805600.03	14174682.88	10951461.95	13448355.28	12901230.84	16755503.3	18100513.01	217764881.8	163637447.7
372249.078	402640.9644	405300.5087	385126.8154	522860.5414	475320.9954	437404.8643	453083.408	444894.3014	443798.6203	467996.4912	773128.451	893971.9043
2225307.297	1161352.834	1652422.486	995904.3144	1981250.644	585995.4065	1299425.146	1788688.844	2467357.676	1798129.427	1445059.906	26964755.41	41698619.31
27919252.53	12857214.61	14533624.08	15921811.68	16515479.91	19882869.64	32013019.96	15679709.82	22870929.67	22107020.68	13787000.72	25030106.92	32621054.35
3595724.099	4617105.426	4133452.765	3218748.645	3582164.522	3646172.496	5078883.931	5223318.18	5696867.73	5588183.609	5092976.306	2869092.935	5211616.709
2375729.638	4599574.516	4940921.155	3451630.53	1936003.329	497572.6015	1871891.396	2812694.36	3232404.136	2114802.917	1869349.486	736623.0305	293708.0186
127763886.3	103616964.9	112077518.5	139765751.4	99050634.32	212774719.4	167669615.6	125076047	149391629.1	134150539.6	150849174.3	735646004.4	690689603.3
414331.7785	613615.6363	341140.1115	494878.8937	190261.5126	206147.3135	112321.8933	297788.9953	260680.6566	127631.4455	230063.7551	922694.0589	920887.6709
24618215.88	64062524.59	37494881.49	46139105.24	44619519.73	33382701.37	117635909.5	56739224.98	58052119.92	62151031.37	56177151.62	3969073741	2284444414
62811600.11	81839373.15	83194399.84	88465334.25	78681228.92	85282481.96	131948665.2	101010548.6	103188975.3	110203007.5	120114857.8	1629335.206	949314.8915
23904193.59	8254258.919	18303762.91	19198556.71	15154253.31	10384308.19	8313027.548	21753008.88	7824379.195	12784665.78	7521819.039	13017796.44	23384494.22
67012826.69	32068875.42	51428879.85	72745208.64	76972201	125498027.7	109476307.5	112157655.7	65379948.49	91945488.45	97153531.11	56042953.02	64218750.46
4266028.33	2936627.093	2421117.284	3180494.595	3497143.552	2336676.448	1649619.628	3668418.977	2272803.771	2370760.808	4459897.559	3726937.144	4142750.971
27696278.47	9482770.72	13371838.06	16103608.31	30492074.87	39517601.89	4499560.332	16749386.49	17795300.5	17400742.48	26458361.56	1170932415	1699026806
149917671	165984431	172649236.4	151675678.3	160386332	165229933	174833859.4	166689437.6	188911518.4	179838009.1	164640218.7	451637419.4	432086076.8
11103395.23	24383593.17	18893979.11	22589771.73	14658517.8	17008041.44	21322591.61	19038070.34	21112643.44	7106172.336	6662787.668	55900309.5	55331287.76
162030519.3	128435272.9	130007424.5	82794094.02	128699980.4	140368746.2	172526686	155857509	147215210	171579542.2	174118008	10347424.35	7413149.475
1641694.628	1865833.569	1913392.373	1863488.411	1904009.837	1831224.791	16457227.7	5757288.148	9241604.404	7745161.74	6218538.408	9025795.847	21619106.04
1568240.457	2219761.505	1733429.269	1688218.997	1724929.201	1658989.915	15541106.69	11241901.75	11012188.15	13283331.29	17006383.83	3284114.003	3209934.036
8814403.344	10329618.37	9837336.786	11074645.46	24627990.07	14318020.01	10718938.6	7671967.037	8139577.425	15165490.71	11593520.34	6782679.533	7101911.959
2109673.513	29773147.52	14068611	8406912.73	4270420.856	5655558.58	9107580.324	17434190.91	16948315.08	11215994.13	9108272.541	2647956.556	4985716.07
1228610.539	1273018.639	1608828.14	1336158.514	1357481.551	1332497.569	1231848.691	1381171.289	1339074.563	1191072.989	1118740.698	2913278.839	2937159.547
13180691.76	5580037.958	8065156.22	9791171.217	20740582.23	26124659.17	1911769.242	13308256.03	12349980.43	11452834.55	18283580.84	63622186.13	65482549.03
3344744.332	14045883.91	19970243.59	24945682.41	11980544.14	1653475.854	2360978.321	4543701.918	3647903.107	4075549.987	5747471.46	5314267.583	4737564.666
1039716.002	1869297.922	1532147.846	2722407.72	1327606.335	406105.0156	577043.6929	894930.3592	1033163.383	470453.5472	495869.2075	1073464.36	1247405.533
22169147.97	44798747.96	34758780.92	34748096.51	39616425.7	33872767.06	110001038	73178552.17	15101479.33	99995626.22	84163103.15	2813707.315	2101077.994
26366545.28	31382704.6	37506366.56	22759003.36	21506248.63	31898038.19	23776384.32	48677639.69	42073502.67	30615278.34	42054631.38	146071782.5	137983024.1
175079372	317131343.6	321140702.6	312794746.9	332624681.5	235253338.1	296579401.6	321910715.8	318770369.7	335884051.9	348749044.5	1164671955	1738073457
13065257.71	30649814.67	32576288.67	29813137.03	29676269.8	32811522.21	15179452.07	15894995.77	15313985.53	15951496.62	14142203.16	16327893.37	12283621.34
14225161.43	7841070.625	6210606.966	8530707.005	6670555.618	4577183.758	8046090.279	8628538.897	8046687.148	6717414.255	10893037.93	39304182.59	59410598.42
11810192.14	28094276.21	24415548.82	25954606.36	26860001.79	24039507.32	56661014.38	40788326.41	43896054.46	46044131.77	43129672.28	15006068.07	127873582.5
16989338.33	14573709.37	14628964.89	14544190.06	16390234.55	17541463.65	15256584.59	16183719.3	15003881.94	19903111.68	18230787.72	4204056.502	2761074.887
8008344.05	14772569.25	10781576.12	14573788.98	7053018.552	3703939.536	10145958.55	14080183.78	15558947.88	5171997.108	6073325.388	10254748.57	12897311.1
29522326.17	21220548.69	19716088.49	22673056.19	22880591.84	27256542.54	19768847.6	23590129.68	27032694.88	30488336.33	29622422.1	28649749.42	51300968.42
979526.8604	1553071.261	940087.9238	586566.3319	1178191.465	852351.5534	5703850.215	819555.6772	3411902.666	1063331.33	565586.0095	15128371.75	29042949.19
35936519.45	31601961.11	31903508.47	36025211.88	29187362.07	28980653.44	28889156.31	30996370.97	25290699.55	34494888.07	34126211.98	50216881.9	27305994.16

all_YM1_5	all_YM2_1	all_YM2_2	all_YM2_3	all_YM2_4	all_YM2_5	all_YM3_1	all_YM3_2	all_YM3_3	all_YM3_4	all_YM3_5	all_YP1_1	all_YP1_2
8325607.001	9137231.07	11226529.2	8972922.853	7525159.155	11816624.39	5904413.193	6697333.852	5972759.955	5884386.878	7239948.618	31781925.38	28023397.84
7481637.598	17176773.17	18702727.81	17417439.38	14215021.37	9405451.661	7114625.259	10790461.14	9445066.667	13278293.66	6426733.767	23617311.82	7230722.927
11450646.08	14414076.25	16459677.65	23541275.94	31071204.54	24978206.07	16478526.16	20435212.7	17454738.28	25509289.85	17050752.26	189702751.6	154574447
16244152.1	2743986.371	5904635.899	7380620.945	14534468.64	15344619.92	2215763.83	4271929.647	4546125.69	6528735.295	9227733.144	118932389.6	215360936.9
16585693.07	17824809.55	15409954.36	16192356.78	17491063.32	16130782.75	12025008.66	18419988.16	17018774.64	15628854.73	13741207.44	1083385.317	1733856.904
14919628.04	12300438.61	10321417.9	9116662.855	11450894.81	14104431.03	26142696.41	17044120.56	17753106.11	20001625.69	17604766.25	3069257.134	2913001.741
38710831.14	35556322.27	30657664.97	72319786.69	52499592.71	76955484.84	74725718.35	54019829.42	69627751.13	64573107.52	74972630.05	251910845	250376557
272883.936	274341.3619	292914.2977	281182.9153	261604.4976	240618.0603	252472.5013	269097.3442	253325.0369	252437.9745	273687.9732	17458915.92	8422082.049
3677101.556	3933502.503	4163103.948	4148993.117	4716020.228	3680595.151	3651050.007	4810883.507	4238230.369	2788261.186	4886814.296	49179532.55	48999412.89
1489743.409	4780110.337	4232211.261	3662362.669	2731459.202	1356022.594	2987440.862	1848545.332	3715386.193	1684686.181	1370062.778	1523388.767	2663952.293
19322701.27	25248749.13	28362868.98	32409552.29	30139266.62	22249659.65	28387433.93	23588584.81	25544532.69	29487919.94	33034375.59	126165203.1	82284730.41
2554782.421	2875396.414	3330894.941	3005924.983	3322226.144	4165849.535	3683648.617	3579333.632	3871790.879	2243176.406	1645331.66	25714017.15	19109172.45
443467.7574	452634.9093	392199.2285	339708.3878	370101.2226	383591.8934	288583.574	394075.5022	416241.0408	349914.2709	302934.0724	1002362.685	1159036.48
22577257.53	31428412.07	22852930.17	24760619.15	25698423.51	27348768.22	28878616.26	28128634.46	28174630.91	29977031.77	31196236.3	10141371.63	11854268.57
85029472.57	59630952.16	59120458.47	67577883.91	63033460.41	78636285.77	140933946.8	54021714.24	52172876.75	46121420.25	56959646.57	362859810.5	310960902
7754531.401	9358970.359	8659410.176	6279264.176	7670755.421	7057125.215	6645850.069	12425424.74	8904533.404	9134380.009	7739125	9507983.538	17894817.44
10141385.77	6305607.675	6027691.401	4569689.399	4365807.242	4999975.498	3914706.133	12001600.33	4305757.669	4259839.173	4052650.319	8091456.149	3233894.748
14761031.01	2441984.461	3344273.952	5359931.114	9666773.322	11303946.94	1489473.635	8223061.541	8637948.126	10521429.91	12282794.65	34603785.24	126410861.2
966988.5503	1268308.461	1190603.305	984865.1661	1052176.874	912988.0722	865814.9033	975525.1626	1107458.961	706649.5008	772839.2169	2573069096	2507200565
62459055.16	27837545.3	31623965.61	30455075.34	28796731.57	29203055.52	35068202.33	59531722.63	45414574.35	62023828.21	64890596.05	7604474.82	4552957.616
11735712.7	17271267.01	10839646.84	25243174.52	23133623.34	13425391.97	22508799.18	18673144.29	18870096.21	25308054.41	22964432.17	37776221.01	33119006.31
5882657.987	13449967.92	11984085.17	12821796.55	13718329.08	15406921.8	7156614.095	7315129.859	6769787.258	6842089.501	3925833.7	13231478.26	11060070.47
4491057.386	2421766.254	5004528.087	1894905.685	7308946.937	409653.8937	3248555.44	3358929.554	20221924.42	312943.7348	625888.8128	2720655.666	18340466.79
10840640.4	36514953.4	20131667.28	15650606.84	34260895.91	15142245.59	22219937.01	19089277.35	17626465.95	15496919.65	14968084.95	119021876.6	120810240.5
9535003.073	8285829.069	6627271.431	6970338.001	5449057.542	4012155.952	6071300.968	5719821.776	6786796.328	5084440.555	4799459.793	109237632.1	171509451.1
8757999.503	14685452.27	10106161.96	6886629.996	10971310.3	10889839.38	14236443.82	8094489.085	12864845.31	11218983.64	10217370.21	16028717.51	17784533.29
4687972.605	8055147.539	5970396.473	7619204.561	5991203.137	2377409.227	6246889.067	6697825.497	6277777.764	6720227.392	4177133.327	2206377.586	3805579.124
2254055.031	2459840.941	2331666.959	2981306.192	3305035.929	2837498.765	3202554.448	1011046.693	2809832.906	3384425.456	2872123.186	184752510.3	177993133.3
1299229.671	724029.0458	1011665.006	623879.4742	1892124.851	633165.3524	1080693.012	1020066.913	1499065.446	2707661.345	999093.5517	328937.0517	348208.3383
249091.7271	700149.7643	417052.0243	356738.6922	408481.5121	143907.1536	12017995.45	1326057.298	2662527.726	1754370.56	563225.1871	4126675.154	3935313.955
215730.6952	5703594.348	9466955.458	11123733.52	2144138.333	177953.7673	251401.7906	705273.9568	564210.1916	287591.2533	270340.807	249150.4323	186819.869
3221282.96	3055644.466	3770776.462	7831138.515	3594315.357	3409801.299	2566793.655	3936029.554	5207454.048	6887479.203	6601614.533	786840642.2	875438732.9
2406072.128	3021266.476	2478613.647	2413344.105	2530078.708	1480312.139	2902256.212	2222634.446	2448626.498	1630328.983	4368143.959	35874093.45	33717520.55
3311626.283	4288325.916	3463003.838	3818442.159	2461968.414	2529730.2	2337834.704	3118079.513	3679291.387	2506748.024	2079635.667	2231857.491	1475294.645
9689543.478	8154793.866	8224522.477	8847652.074	7007172.306	9149382.538	7213753.523	6809907.119	6658967.987	7929257.502	7456350.509	15525480.81	14719688.88
6827321.196	4965156.853	3995610.086	5068293.692	5230676.021	4192246.179	53752433.72	20881120.1	24092771.66	20475666.67	43985587.72	18634206.16	53363061.72
9203682.667	1213628.788	2796825.167	4441854.754	8792105.547	12595140.48	1079209.282	4084858.175	3734071.039	4232865.959	9420198.226	13056189.66	38961103.22
13986414.23	9784860.874	12909083.37	9975758.612	11472036.75	10082680.13	10401426.84	11034810.4	10859101.91	11566008.82	8547500.411	15319782.21	18511227.91
1495552.126	606285.3018	580900.6324	103560.5054	987812.3508	1022982.969	1079597.28	1501744.042	1414416.155	1735534.418	1696783.234	6319809.767	2842640.935
18923626.88	7378684.456	16633326.69	17898111.62	15319746.63	19440813.64	3346687.665	3533546.078	3709876.763	3754112.059	4015195.831	77633160.62	33740807.26
1169295.638	1166548.062	2760212.719	4189088.449	1392559.152	2724506.384	1094612.604	1607645.756	3224691.934	1250954.23	306210.8131	21100216.6	49562939.52
614888.1478	6117677.683	13029813.3	14869781.49	3318079.348	623447.7605	381292.1675	469798.4668	554342.7399	461839.5896	534853.3138	259783.6664	359178.4819
16673990.76	46439517.09	41266394.99	26270578.73	18774489.32	65639691.73	12702538.26	16105110.58	50336204.35	25472286.39	76044018.77	71859867.34	36780603.62
33878602.65	34598409.29	27865329.79	38593212.35	22872751	54914859.21	41342365.08	29305453.32	8074709.036	32860317.06	39874919.02	65465724.58	44309645.03
3049900.583	18845631.67	33088624.59	37101968.18	17801564.86	7052507.896	3433212.526	4984732.537	5592496.096	3949952.259	5135391.182	1842308.279	1172636.274
1146352.917	1264842.275	1178760.803	1058502.67	1194218.15	1212572.366	1111119.419	1085627.331	1165024.707	145883.3153	1368972.075	702576.9684	1559575.992
25692062.15	24384789.36	25866532.41	22874352.19	23243017.65	30045775.24	39820456.56	36926788.22	37724101.93	33809894.47	34469181.19	497088906.5	452580985.4
2415823.312	3020119.849	3344103.986	2793636.387	2343278.393	2760537.495	1790835.884	2958481.581	2841712.845	3588982.19	3368141.361	3893321.032	3856964.861
2755092.679	2489553.279	7517026.595	2266662.319	1863200.409	2196323.378	8083561.931	1720565.349	1945207.785	1785557.305	1239721.867	449225.5959	471318.3129
2314167.805	2331545.515	2269330.062	1797685.497	2308750.013	1890667.767	1977346.475	4809435.004	2133371.04	1575508.776	1726565.619	1769282.478	2903837.144
11065125.45	9187947.122	9615874.251	9680375.865	8427898.456	9496161.648	6636602.059	7160443.731	8376381.128	6330953.263	8531544.209	19159548.87	8374263.983
2452090.565	1425751.409	1524668.516	1662378.235	1332744.852	1693875.115	16947073.06	3836379.602	4040558.271	4096292.149	4571316.935	8360443.93	19396618.11
3931273.881	3037974.879	3155491.057	3488543.125	2578454.816	4264068.069	1846690.804	3438270.703	2517961.761	4027863.897	4589772.562	5093179.579	4195819.998

all_YM1_5	all_YM2_1	all_YM2_2	all_YM2_3	all_YM2_4	all_YM2_5	all_YM3_1	all_YM3_2	all_YM3_3	all_YM3_4	all_YM3_5	all_YP1_1	all_YP1_2
817179.7477	620278.7688	770396.297	848853.8547	796425.9643	965775.8247	662272.6653	1479256.19	1388857.74	1962228.959	2891897.69	2877977.916	3970568.184
431760.3961	776360.2814	614910.0259	499617.0229	520339.3012	439358.2866	431373.827	512320.5318	512764.217	365593.1241	431393.1987	2589265.107	4200922.524
778948.709	807277.9011	856913.4374	1500170.096	159346.784	190013.7086	8016549.173	1315567.712	2984996.003	2192546.904	4391106.691	720488.9142	723513.0939
2867963.806	3704947.539	2763448.979	3124129.411	2842322.489	3237349.174	22982751.6	9312133.765	10497987.87	10203294.4	8536064.994	28307884.13	126539835.1
717363.9045	551390.6956	1317063.887	688134.4004	688516.1031	489923.007	764988.3448	657262.0257	1308401.478	594767.6916	519223.7687	262455.979	409112.2305
6817810.364	7284087.745	6847857.352	7219628.519	7017112.556	7030785.689	9154770.275	7482327.077	8122990.15	8370866.952	5738294.862	5056530.303	3696468.779
514084.7473	708818.7823	799511.5667	2494043.368	546896.2466	763363.2506	722627.1961	663619.6185	919582.2645	647338.3681	532475.6987	294112986.6	490891474.5
1413948.342	1683795.06	2128389.21	1776451.991	1398709.549	1588902.818	1292849.32	1705865.134	1811258.486	2266028.416	1923485.13	2322090.856	1435601.582
1237780.141	1370294.225	349019.2984	2134396.224	546329.2131	1649380.615	2838651.731	1821618.34	2205189.488	1290459.752	1341404.256	15138908.08	9811128.109
96127.11632	103875.1303	109052.3239	102290.6898	104969.7215	117163.3616	105864.8476	107435.5305	122529.2772	111076.6064	120326.2493	22075383.14	27974995.62
1674608.248	1633883.125	2536188.539	2449081.286	1785761.358	874021.9117	1730441.709	2593031.965	2509177.266	1362288.374	1541212.344	2122250.345	6635669.356
7302320.408	11817169.43	8549159.398	7305974.956	8567702.934	6314954.115	6074399.385	9063653.874	7718509.157	8477809.571	7360688.292	15959771.93	36784107.14
3025444.739	3306328.411	4252504.749	3350099.718	2238834.505	2981048.117	1577014.983	1963284.74	2101918.114	1881646.924	1962476.584	11216522.7	15149662.29
243060.0965	248821.716	380265.608	351490.3097	273818.8515	152821.6855	118546.0325	161396.7631	152145.0182	157574.8257	522441.0195	1812036.472	2303949.145
906158.2653	1383241.933	1394978.941	776183.1902	1045658.568	1217013.697	1637843.261	2012760.428	2436649.872	1227809.794	812223.135	87488160.08	123187442
946722.9753	4542960.971	4650294.12	1513576.071	810326.8244	7442080.785	1019295.415	744187.4301	6185236.492	2599029.365	3907992.866	3213228.375	2839811.155
8719416.295	9424058.902	10317423.05	9958561.828	8428197.345	9488003.507	7170190.7	8695311.658	8067385.854	7587153.564	8986094.81	174182709.6	138440682.9
2703329.92	6783166.56	4590697.499	2253909.44	1614436.576	393787.7067	2428593.578	3020368.457	3861259.245	2032641.133	1774322.463	84851904.27	77925307.73
595832.6896	1067333.783	838490.0655	745337.7596	257431.8744	224414.5887	174081.7021	237006.8625	630818.5697	231394.4489	260278.4122	1406537.651	2492411.004
2718673.176	10055844.93	6967056.779	7118889.262	6264320.86	5462925.662	3387274.087	5443850.197	6103777.82	4642246.432	2363248.214	1468643.521	1510320.287
31218505.26	4953960.618	5989198.941	5153925.491	28945801.08	5299259.411	5113465.321	5847248.312	7142168.669	16669635	13557930.93	386064.0832	2581410.239
3458625.303	1968424.845	721275.542	4579187.424	1952734.112	755531.2766	3926414.743	3141971.871	2903255.78	3714083.148	3696420.128	12527731.69	11713678.76
10240474.46	12613189.38	12960978.67	12015218.79	11809338.41	11282330.83	13046885.5	12586526.74	10213388.73	12245556.94	12577810.33	2529964.301	2095152.317
3001274.223	3293175.062	3874569.044	3704423.044	2996744.834	3267747.213	3920260.92	1800580.818	2941595.111	2407239.721	2444186.373	36587886.29	65243392.15
2416462.577	2367658.34	2422748.671	2272206.335	2794437.052	3225246.248	2342581.408	2495308.687	2237756.352	2884983.352	2499198.291	13137630.77	6307395.81
10034254.25	12160150.75	12595454.49	21765155.79	17833436.22	13393301.44	14488514.77	14051443.37	14915205.96	16804511.74	19171618.32	77862140.19	68974979.8
13177208.08	4664504.195	4165063.513	5278906.957	8987402.622	10892090.61	2236848.08	13354432.06	10414482.09	9528516.533	10581081.96	513717620.2	766235393
1746721.164	1671775.823	1746881.853	1905851.446	1335345.108	1875624.136	808886.0043	1501498.007	1371952.446	1249404.812	1368196.789	17581653.18	18745999.07
4029319.548	5466434.184	4642085.374	5593042.514	4934974.574	4672577.762	4534213.563	4918036.313	5213579.776	5315210.551	3719829.265	296431.966	513945.3367
613066.6878	677558.7038	707244.1184	494791.4723	666485.8542	623762.218	492635.4297	1167822.701	624848.551	1503317.886	1678990.218	3848482.076	3414606.737
1697675.973	995170.8044	589755.953	1053708.817	694516.2971	263268.6901	7477131.817	800218.3445	893131.983	1721924.159	1029590.003	3340577.285	5102248.296
4335702.083	6450584.039	7067543.15	7548069.908	5400933.596	5771714.951	8610093.528	5684676.676	5551631.502	6441617.252	6268524.952	1438258.317	2000569.212
156864.1487	704744.1707	2297146.266	1595605.033	319787.6019	265377.7302	160497.8435	199460.5769	191794.1606	181994.3942	195500.5977	3219532.723	1683536.215
5926333.139	8015271.06	7223982.98	6701683.487	7231090.056	5927494.934	5597935.31	5801558.574	6143700.659	5535825.356	5202213.855	2279563.98	2221230.378
2760506.414	5656065.155	3327548.634	4578140.51	2550051.042	3410685.837	2500929.864	4035775.522	3205860.937	3509263.722	2900681.924	1362611.477	2329303.238
3448770.376	3787340.683	2039014.63	5298540.886	1597452.246	755050.4812	2188907.578	2073571.146	2647062.325	2302654.242	2039875.631	8502846.316	2403670.662
1590395.69	3651179.714	5752080.877	6219810.915	3499583.672	1520355.569	1525403.596	1697756.052	2235423.909	2006693.798	1527777.129	4959927.751	4145577.51
43118757.36	48261182.33	30618500.94	27770021.26	29958474.1	31091109.1	29090005.72	31505073.12	31210815.57	30014749.56	30492911.99	28574031328	22873355318
5210581.644	5637724.861	4511139.051	4881827.634	5656883.488	6163284.48	3981032.872	4100722.233	5248316.085	4638916.177	7278931.584	605587.1394	942147.5308
924220.0951	1348337.321	1236717.841	995232.5774	818708.6704	996192.0851	1184386.149	1151818.952	1738805.648	1054841.133	1244903.42	3666694.098	6244475.752
2713613.006	4347706.729	4386562.027	4038367.67	2524001.158	1962255.605	1991381.539	2816741.89	2355161.227	2333626.099	2373802.682	71832786.39	30372828.45
6526396.671	6692253.21	5298291.427	13937122.28	15650313.52	10449525.07	8230855.445	10234500.06	7345650.01	8233377.819	11700827.4	8878416.927	9427094.827
4192673.114	2367157.502	4017468.082	3954580.026	2791643.518	3986398.197	3682087.392	3855585.07	4510402.214	4431077.707	4500503.811	1281153.864	1209491.575
876468.7058	5276265.562	8697876.779	11261547.87	3526518.06	820878.5883	772736.3369	920268.629	915013.3355	2197809.376	821461.9775	9998690.843	11699948.12
29250503.48	40461820.95	25191906.44	56184094.46	52030323.77	33250218.8	67016119.19	60642471.16	54710951.03	74839100.9	75137671.77	126260485.2	250405363.4
1423521.421	879581.8152	847984.0668	898561.4532	987683.0596	1074109.369	5936133.352	2151609.018	2589113.538	2337773.778	4393449.892	254606.7233	246102.8502
5568881.712	2776018.882	4183184.822	3925334.805	4084147.982	4671849.535	9603810.868	5407415.507	7936684.479	6774554.87	3638090.162	5289968.743	2547346.958
1232583.721	1788508.378	1273184.888	2331669.601	1544723.549	1036221.38	1612730.563	1756211.63	1933856.022	1283271.997	1335630.563	747179.4845	927875.3284
3410069.147	3121716.733	2042576.113	2024416.962	3781615.94	2303587.79	2953480.681	2794801.742	2994454.132	2955701.921	2945441.787	5900608.978	4986307.288
1891988.548	1189672.034	1069921.502	1064725.853	1302732.81	959608.8602	1196210.187	1345795.682	1639162.961	2005528.013	3315246.435	690579.8128	779210.8903
8922018.922	11415559.05	12278707.51	11287479.8	11029320.9	12235774.38	11499478.99	12736032.38	12818307.73	12760549.07	13497826.64	37180160.9	41707607.95
9343108.235	7852329.558	11485830.44	13029200.82	9727325.912	4404054.297	7286398.396	12524769.26	8384127.713	4647200.995	25989153.1	15370454.95	22324145.82
502521.5795	1282584	442892.6001	408432.6547	315276.8386	361202.5742	320258.3605	330194.752	491639.784	330093.9832	342554.4271	38351642.02	35980179.63

all_YM1_5	all_YM2_1	all_YM2_2	all_YM2_3	all_YM2_4	all_YM2_5	all_YM3_1	all_YM3_2	all_YM3_3	all_YM3_4	all_YM3_5	all_YP1_1	all_YP1_2
22091888.87	6841425.02	9712398.305	10561136.4	8598385.012	8876780.85	8865113.731	16272355.85	12183044.7	14260497.26	9915032.766	19196558.5	2136724.24
9554423.495	7684124.989	5998014.105	6708466.405	7514763.126	6400996.226	9376979.866	10064129.77	9326514.26	13855651.5	18745012.48	18189880.12	18123330.96
150648.3699	142065.3948	150786.5823	159105.6626	139883.5771	146484.9253	133249.507	132526.2801	136609.6337	154667.0296	138664.1827	122934.99	118482.2064
7756034.889	11997525.49	8075078.161	7573623.669	9487258.92	8773809.779	8023321.153	9076875.853	11342779.1	6699601.475	9633755.753	20746285.7	18549004.44
17262782.58	18843141.17	16951840.78	16587816.04	19665398.24	18277406.2	23466547.78	21381863.14	19729017.96	24128116.23	22420578.74	13844740.58	9266262.123
3524081.278	2474639.703	2216877.741	2968883.882	3929282.036	2374920.342	3667774.039	3953289.469	2449755.985	3080008.864	4367028.176	2812150.314	2944413.81
801242.509	2431172.594	3363902.022	3680843.752	1074829.096	754025.9617	1162477.33	1738687.848	1359211.028	1328150.034	1057452.872	1237425.258	1295174.364
1401225.606	3923774.202	6252470.509	7270920.006	4434913.237	1142488.932	4739964.438	1735565.631	3205713.003	1669927.489	1553456.375	820916.45	783483.7681
6990845.976	8470182.9	8687773.627	15272395	11311808.27	8285837.761	8835038.306	8672461.658	9361159.246	10553893.78	11599729.21	124027833.7	31713598.79
1698108.23	580619.1012	683195.0691	728777.2534	832116.4726	440889.532	4234776.489	1576454.438	1338107.615	1082060.764	1481835.367	4668497.974	2594098.903
1431307.014	1680943.119	1520401.28	2208962.928	1489411.878	1030296.311	1681983.738	1685382.321	1796850.238	1552564.438	1182458.165	999653.2035	1161798.97
4273168.064	8760001.028	1160408.651	5868388.769	1257312.806	821522.3759	1573770.05	1529112.977	1915793.449	1611755.253	2034588.25	126165203.1	106575568.1
22575307.7	25826525.59	25891003.66	27189624.01	23497064.89	24539716.91	22240394.03	21337023.13	17254710.41	21755598.35	20967356.54	1052984.37	1470706.592
2780462.198	2561270.733	1630875.982	2002935.597	1599375.337	2220363.473	4608203.046	2151127.052	2761451.526	3314667.354	2773343.475	165220.3926	708866.3865
2363359.402	4266763.841	2769398.14	2369281.281	3072807.39	3320854.99	2884567.723	3010808.656	3658620.458	3058919.012	2210721.731	5835165.423	9620843.72
6063710.768	6219200.678	7768168.387	5809688.837	4857569.623	6068505.519	7117481.942	7106302.27	7701691.413	7518727.707	6392755.535	3331975.01	2918735.343
1664061.703	1876788.485	2319179.011	1988941.409	1346591.26	1760186.769	1612916.271	1575911.605	1691165.931	1617942.941	1563269.053	2717179.892	2742475.592
3032197.843	12886940.43	5048307.293	3705560.525	3068593.402	1328802.372	3508068.498	4858869.193	4067578.665	4253450.382	4253137.372	10748607.78	10774853.13
1667835.399	1926878.783	1888756.706	1510457.862	2003628.897	1685514.115	1649901.579	1914678.54	2190360.086	1554815.617	1454327.655	1841993.658	2081000.667
1224839.342	1282091.745	1790879.154	1570459.197	1324963.49	1640498.259	816417.6851	1211343.866	1144251.576	1401176.645	1274704.165	5755887.783	2630724.851
4174633.73	3717448.746	4376826.699	4994851.119	5319989.746	6498175.531	1574052.344	2897115.154	3743392.362	4587213.581	3705815.487	12474495.72	5393742.531
3115078.369	3259823.272	2537354.536	3713135.232	3740143.101	3107271.341	4680291.4	4050562.099	3423420.318	5231925.224	4143827.679	3202526.424	3046893.218
11281027.44	10725358.67	11606601.09	12101900.6	10549135.03	10042287.19	16982166.89	11752194.02	13114097.28	14141249.96	13918516.05	1353747.992	2067397.703
20896984.16	28504141.16	25658541.85	23356697.3	29667914.33	27960860.35	21754022.76	25632927.14	24208116.56	24913455.59	24933024.85	986907.7729	794501.2206
163919.8876	205574.5108	161735.0305	289160.5461	176934.0785	194708.7211	164801.8365	216276.7162	338077.387	178911.261	177624.9298	559250.2933	506428.9574
1880241.687	11063388.2	7432050.158	2131139.236	1168562.399	9304456.754	649202.9942	1280579.628	8795749.22	2959478.021	19736251.01	5186157.629	6443183.29
805942.5084	1543417.397	1660094.147	2663299.909	1207951.54	240337.8472	570684.3144	921030.2969	1572840.427	777447.7483	1045911.084	5586899.106	3980419.335
4162516.753	3779338.774	4860631.822	5530913.4	8191008.873	8663288.22	7632953.529	8098163.978	7529741.741	6601516.075	9520176.282	593593873	771607239.6
859299.1606	928961.2876	1338504.587	995677.5426	902626.6422	1248698.444	1261441.462	860360.6813	992710.4448	1228917.762	1056712.211	291158.8906	309166.8484
4865151.662	1256507.341	3790693.717	3817080.404	3144567.453	3199477.885	1381425.311	5130238.04	1417190.747	2795392.148	1375014.25	7684229.912	5140028.524
124208.2395	157422.1607	177440.6845	208989.6396	148435.397	135620.1789	127976.9807	128043.6483	140542.8048	117497.3351	123510.7975	7018014.797	6673255.416
2629876.628	1996821.399	2029804.184	1722746.477	4499262.441	4425661.84	1150025.177	7820873.043	3877015.337	2654801.155	2636659.216	5786067.383	21580144.27
3618225.107	4734604.988	3115198.366	1020727.634	3172895.702	3637455.462	3826754.522	1236459.287	3415038.428	3851867.136	3155651.688	2107867.162	3647539.888
728183.5357	1300459.154	1086884.638	1149495.025	720960.5626	243760.2358	660094.1741	1543757.62	1453818.767	404480.5124	449584.0041	7060669.528	10206411.01
474690.1608	721888.9946	1072012.19	1168845.979	551228.5351	522539.6261	603543.7398	530491.5421	487507.2572	586081.2075	524212.9355	4706945.046	2773610.667
4534206	7074701.93	6742664.333	14036840.69	19281463.87	8909257.323	7598152.017	8577936.292	9952293.478	11670057.57	11670981.88	288137711.7	164455442.6
3504938.014	1717693.242	2648075.528	3532643.161	3069285.954	2561351.465	1691653.927	2484914.829	2078340.987	3021915.815	2341439.462	8868403.506	5699871.143
41546847.61	34258170.35	20850008.5	26493868.35	33502772.46	27310550.62	77017193.74	55761526.01	41063726.96	51170679.67	43014399.11	84145208.21	101793627
19901494.23	22729992.83	19961539.4	12781287.17	12480452.77	12043060.51	8968718.064	21419747.23	18498739.53	14196972.92	21396931.56	460617362.5	478136164.1
1802204.57	2932763.426	2350570.284	2928379.736	3044280.287	2789572.987	2201410.302	2459408.144	2882089.453	2688140.049	2059969.625	2190695.094	2896188.695
222318.9083	222373.5252	225402.8736	210730.4004	220871.5848	201746.8906	233706.967	182731.421	234710.8664	199554.0203	242586.3816	1816377.701	1547064.027
3680749.185	2403993.326	2498817.699	2204330.201	2847621.475	2362296.506	293988.3977	2584033.41	2221440.086	1168328.494	2804977.703	4403342.642	4129095.528
1137017.585	2484370.122	3741167.018	4189785.777	3037808.496	1748211.545	1175760.305	2206069.356	1213158.833	1531050.492	1756067.556	10992941.61	10937650.72
2481741.811	2590243.268	6317891.491	3941647.868	3652803.393	3567730.004	3633155.751	4764559.898	4400543.049	5731404.714	2376252.962	48972180.46	65073166.93
1106763.482	837145.6186	976557.0389	662856.0423	806022.4018	889543.097	1439259.22	1038917.397	1093344.114	995068.8877	1034592.688	192060.3046	181568.8602
1015844.039	1231085.861	1165048.64	1066838.658	914557.8628	719409.9834	799959.1715	1035883.739	1558830.787	852631.7542	623055.6204	17717644.21	7377873.053
3162173.653	2318004.592	2357193.084	2704810.898	2638795.458	2711035.436	2741508.784	2079877.097	2068027.117	3702181.191	2279493.502	21323164.14	33594889.18
1073281.84	3333078.91	2246845.407	2915235.098	1288316.133	664391.5287	1332720.383	1977089.714	2870331.327	737852.1043	737969.9349	901671.447	1588040.061
793763.0531	860089.845	816203.0733	732933.3738	826906.1223	839615.0344	769366.5094	751715.1587	806691.8616	771764.2481	745684.619	7659434.018	8498675.373
893622.5041	687003.1083	1201888.905	1135017.125	956560.9715	1260552.054	1802844.164	542842.9911	822058.2522	706808.3456	799635.1761	887582.0628	506899.728
19269127.47	18266109.83	10948585.66	7930339.396	7955732.263	10791022.5	6982773.46	16837444.6	14386729.28	11400130.69	16022210.15	93787635.43	90571552.96
810128.472	862961.7797	875083.4233	11899335.97	1052397.747	1023798.613	937460.0781	920036.7071	940027.4292	958204.6102	1010450.179	2215078.641	4233030.122
639948.6369	1902245.194	1628120.578	1340107.711	1615155.607	1081263.354	423786.7891	1211607.653	893975.0764	486748.095	624387.1041	5627784.493	2074303.453

all_YM1_5	all_YM2_1	all_YM2_2	all_YM2_3	all_YM2_4	all_YM2_5	all_YM3_1	all_YM3_2	all_YM3_3	all_YM3_4	all_YM3_5	all_YP1_1	all_YP1_2
35164940.45	14481158	46948052.34	40838619.11	44614462.99	48005541.45	42042685.69	36270432.57	10790087.22	33571069.14	31578756.48	1945238.639	2526039.229
724659.752	1151859.054	949380.3655	902425.3103	275553.6108	242580.6453	280006.7097	263169.7893	296806.0194	242116.7633	354286.2929	35732162.95	34523055.97
10390791.99	11324424.09	12813100.27	12476342.41	10933047.51	13033005.79	14621872.96	13717473.76	14545899.72	14652888.38	15853167.82	3383118.443	3485157.171
5652490.754	4840372.802	2261937.594	7683480.281	6262846.739	18116702.43	14264339.64	11892807.86	10351896.63	11527906.42	10476650.57	920118.4521	731549.3031
1904993.778	983809.3871	2169484.067	722674.8119	20353210.74	6032086.77	1072319.489	4017460.485	2050720.758	4151966.746	3088970.243	499822.534	508239.8644
1835769.325	375756.53	482906.4148	920079.525	2870173.654	2372523.124	120132.3312	759903.7557	398346.1491	871308.1014	1344797.865	2723068.549	1790240.995
5932044.629	6594672.316	6126178.726	5162282.746	6572465.639	5399578.54	5242068.334	5894704.379	11986222.92	8285734.18	5455485.483	682102.8527	527552.828
326560.5788	1965855.042	8762822.063	10151353.56	5656573.012	369261.9698	270052.3196	567108.1946	406656.5367	465140.6385	335055.9768	69335.93892	67594.06968
1207529.936	1198167.141	1111006.531	1439649.054	1552324.394	1546385.552	1139148.275	1476055.774	1553166.714	2084510.293	2070401.41	345711.1184	3983733.94
4528600.123	3740834.52	3386038.427	11133111.71	5693857.365	7229757.563	6525192.012	4267353.973	6038385.981	4740010.497	6430507.554	139409609.9	143098138.3
2631305.578	2566469.582	2608445.113	3461168.747	2498436.044	2689623.551	2303291.136	2081527.736	2313195.442	2527892.196	2357739.768	381327.4767	2088994.656
873523.9154	839259.1904	829121.0429	889896.001	984431.1141	1613844.567	850122.6574	1045614.525	1233267.775	971655.6838	673649.4588	8948506.967	13170670.52
3833670.099	6425915.163	4998670.708	3984242.722	6061824.11	4509336.161	6022430.546	8173084.192	4906692.059	7070838.138	5595010.96	4900643887	5174019699
591356.3236	1087968.841	1792486.852	2902757.009	2160072.433	1713328.862	1905412.27	1125720.547	970114.6608	1359812.55	3507046.263	368622.7998	345358.5205
646394.9072	502703.5394	486428.8186	468043.2217	564516.3081	569980.1963	344907.3422	471223.9025	488419.7099	520395.131	538925.9639	280402.124	2138698.871
962856.678	1637229.032	1561355.685	1271652.715	605940.1961	601259.1092	585342.7018	858447.6072	1019758.574	716074.3876	1105632.74	34799512.07	13657585.35
1785454.827	1463915.486	1415903.663	1449959.135	1363737.217	1497006.304	1839454.874	1609640.429	1628455.204	1596177.329	1338084.002	2153187.947	2123605.212
544546.7182	383107.4532	484812.1209	370063.8177	491748.994	825947.8416	329572.4669	548085.1265	329479.3197	759925.8157	699608.0142	1700372.815	3279254.428
266671.2678	282259.2511	304761.2656	297276.4097	283401.2902	298204.6211	292422.3331	305869.664	337450.6616	282272.3742	533487.115	565068807.4	537267035.3
101863.6208	82765.54377	92296.1107	98199.67474	99274.23865	99484.12301	77946.96217	86284.78869	82040.68516	88719.61637	90912.42078	352804.4349	316719.7446
935447.7667	1375215.61	1346067.559	1342957.078	1089834.046	300457.067	986226.8574	1555433.494	2213474.234	786141.6479	637915.4195	754552.2281	1608236.083
373995.4699	330325.5102	352583.5999	318403.7748	363647.1714	433335.1625	360376.8289	186827.7361	218774.6861	291109.8845	273801.5084	12022621.74	9427633.172
1745574.501	1849490.477	1951762.827	1806067.658	1299129.404	3085191.3	2331419.178	2587880.305	2875796.725	2031957.566	2256086.91	1268194.456	1332336.974
17114840.48	18324957.29	11898885.41	11050341.47	15971462.57	13094719.02	10646979.21	19518524.07	17813652.54	14423996.96	20179505.2	99092204.52	82041813.34
1110494.38	2142065.427	1812069.827	3688125.905	1991423.757	1312538.608	1351241.294	921977.4549	414902.1791	2060427.643	1955603.557	21477495.36	19540836.07
3685020.393	5851824.203	979168.8453	2377260.315	3346815.482	4026899.223	458941.8651	626708.3865	661958.8114	556477.6468	481763.8312	479427057.8	453757264.7
4630012.505	5592357.648	5197353.208	4853969.848	5237639.862	5469396.139	3447012.076	4396145.844	3863166.147	3392370.061	3479000.669	21084089.71	18971402.71
1776138.796	1131914.036	1058036.344	1160813.086	1243342.066	1340174.649	1915019.655	1316520.89	1223574.289	1386844.617	1565762.016	6777084.787	16134009.49
2312173.748	1760959.977	560097.1784	1971718.467	1762133.324	548377.1661	1910559.289	1709827.488	1619225.23	1939057.834	1817349.532	2221823.116	1697927.199
883615.8457	1244405.502	1493244.905	1205305.702	1038438.877	942909.3045	856265.5863	926867.1046	1040831.653	790351.1937	714790.5335	3236450.542	3685130.624
851395.452	1051090.772	529110.964	720682.5902	475149.5494	803292.3983	1577317.856	1382432.93	1107981.48	1257421.56	293335.27	356286.0968	344386.1292
1010703.499	2124027.953	2883915.402	2385221.038	2189437.402	2135380.282	923006.8114	1234978.952	1025200.946	870136.395	1510204.807	1436031.811	993871.2295
879468.2851	712355.6411	728379.4311	608728.3047	728670.9777	1153745.763	589807.2204	637437.5831	744912.1749	1541469.185	773696.3102	6977297.358	6969082.237
17166568.89	10940068.61	10226032.91	11219380.98	12017032.27	12952929.41	9525393.591	12724313.34	11825974.62	13403999.57	15133255.11	155641.4726	150252.6603
2663939.468	2218010.012	2668198.838	2526962.653	2138543.922	1814258.181	2412563.431	1867550.081	1897086.459	2778473.397	2156830.987	745331.5996	669811.3273
6479393.056	6127145.117	5578793.241	7435372.653	5426880.014	5769514.711	4876324.481	4855767.328	5140350.574	4431373.601	5018228.466	28373364.54	21173630.58
453539.5521	462103.583	454960.6191	322746.9957	343824.2608	342623.4831	496841.063	369995.8761	488633.6461	352445.6586	334030.7574	7315116.803	14498322.15
2495003.178	14532488.57	1433088.614	2024220.768	499834.4013	17213111.46	22832522.71	1718388.621	20198352.82	23266036.91	25719554.93	26062829.44	29134771.29
623193.8665	919651.2586	1069548.037	1138721.854	1396682.146	1294516.492	544044.0323	669763.5766	769470.7358	864170.0339	3738311.528	402718865.6	396286727.7
777690.536	805323.1585	938851.3387	969095.749	851257.621	936514.6097	1670708.49	1138859.768	951164.5373	1429396.02	1268886.24	356622.4977	444192.4741
679616.0871	1601043.421	2388322.76	2997262.423	1608295.643	701441.2149	554631.0231	632690.708	818986.3006	830373.5137	763337.7373	794869.8866	735363.5534
735810.9362	938358.0988	888023.217	788274.8047	568934.3866	1183353.348	319677.0458	306912.171	517297.4842	472888.7949	563991.0628	1069254.858	1477979.954
1107077.715	914688.9499	1220250.575	931494.315	1060959.327	2227260.598	1662961.198	1346799.763	1766427.291	1224765.336	1251007.835	586645868.2	557540616.4
393595.797	632158.2023	548376.3366	359764.9731	456538.443	308942.9046	296630.8154	449725.59	374387.8436	286869.2572	336931.6958	1542504.068	19077387.34
130162.1218	145976.0467	155282.9144	148650.961	140255.0397	111267.0007	119278.236	120487.5247	134000.5963	122896.5493	126152.6481	1606194.229	2134782.869
417818.0231	575649.9674	1088403.867	1675501.557	433881.7329	483970.6123	252635.8542	277898.8876	341692.1196	326253.5366	375173.1673	10705935.16	9238183.903
7032367.158	9891188.499	9420908.469	16280544.66	14809084.65	9143612.132	10798104.74	16779425.12	16278928.64	12978461.75	20436450.68	1952079665	1949271137
159600.9883	406740.486	631445.7358	580664.0857	181196.1189	172741.448	153470.5574	185375.2327	186759.5698	175922.4353	171208.3164	1568540.196	640799.5538
550083.5905	661991.7192	661098.1356	519986.0344	677009.1213	528151.5433	559131.2711	695185.4301	776358.9717	456650.9338	488957.654	828955.8385	2812789.863
2437561.686	569882.2413	1584672.759	2656952.592	2420315.917	2719115.439	2036625.756	2541585.699	2333056.451	1823639.795	1693383.554	8790845.518	6712423.056
1610250.537	1739156.673	1355950.765	1324981.65	1640885.239	1381628.492	1130901.049	1609819.103	1209106.813	1358372	1223995.312	609630234.7	588566984
194340.0198	106399.878	111443.5471	127751.9111	128877.9334	160834.746	97855.61768	120845.1098	119043.4686	121721.5961	137109.2762	328375.8631	1776880.002
435150.8568	738993.9512	1403914.151	902430.9779	756760.8741	376683.8468	1121512.188	1698027.721	1119399.466	1277581.721	1838851.142	5395455.121	5435634.065

all_YM1_5	all_YM2_1	all_YM2_2	all_YM2_3	all_YM2_4	all_YM2_5	all_YM3_1	all_YM3_2	all_YM3_3	all_YM3_4	all_YM3_5	all_YP1_1	all_YP1_2
330568.5809	283529.8544	280899.0345	124228.4558	453290.7521	141259.7215	90033.85295	621379.3917	105791.8179	220175.6003	148157.6067	48908220.81	126411982.1
377872.7661	1705408.957	1881185.467	461944.8263	1091824.898	1507699.211	626886.4376	501461.734	461927.7009	581192.0213	493887.461	617385.6998	600973.5091
149099.9939	99355.44888	108688.8581	107952.4717	104350.6341	110272.5792	120071.3691	124622.5069	101834.9424	121378.6742	126698.2555	1372512.197	971497.9287
757510.1107	553116.2426	937309.7402	908087.4067	458118.8109	364329.2176	544387.6858	679759.2994	697629.7059	696751.6164	849230.3081	11005215.94	7454368.209
592084.7334	599751.12	726207.5432	612341.8465	589328.6836	455199.758	574143.3852	506337.2821	304338.9582	377819.4817	350057.2944	521463.5539	502206.9907
171338.7527	184319.0953	540398.7107	181305.7648	218667.7296	228286.8098	192848.9935	200629.2787	204483.5988	372577.8644	371951.5101	269613690.9	287892820.4
774284.5645	756435.8877	730133.0489	981040.1977	785603.2496	664558.3117	2403199.932	1657584.136	1576385.129	2134488.768	2042998.655	21572561.67	10417087.36
599309.9435	379487.115	407986.7797	219895.2092	405350.0472	463597.9324	452552.9928	484300.3172	343864.7421	448105.7522	676737.238	21544448.31	24100008.09
441703.5696	680246.7209	529510.9912	1177558.369	777375.9165	330599.1831	457804.0791	294751.1103	827448.8136	542896.0574	1186078.057	8142594.028	3738330.476
1319485731	2526672380	2511984673	1395978715	287417964.9	527567112.5	2520714051	1539303322	1939213982	1894837574	1333946918	55286259.31	110934602.3
1571533332	2126059740	2019971547	1928350686	1772115263	1812573511	1974111432	2009256518	1694546213	1985942540	2096931178	1670411358	1650095837
1025971339	1556603693	1133791991	1198325087	1229051642	1117749630	3232290074	2528121485	2121865282	2660473756	2737039848	1218636348	2062886283
698998294	808184130.3	788580098.9	636221274.6	705879123.5	750526572.2	742093319.6	750397087.4	1098943311	931538378	866933991.3	10478181.47	18905312.04
1496800724	1609856071	1769933542	1678947810	1584247221	1600617171	1410416298	1468146319	1529126313	1384478652	1470008804	7579351281	9956203612
2771944144	3614024248	3635519132	3951641900	3881975666	4495151241	4151928678	2434132891	2080940475	2372040790	1808977978	8540423802	7982402349
57263474.01	109268669.2	500248874.3	402286691.1	79287167.49	245109187.6	1226954855	757925985.6	748700396	847700130.6	705312983.1	286394636	213813689.2
2516990868	2507390333	2741505739	2829192062	2563467141	2641911708	4199360190	2742190578	2920378141	3454569864	3652336135	6096465855	4370117122
724016836.7	1188145677	1112408406	2578595946	2379541088	840302980.9	1773902587	1285716413	1046342263	1158281748	1205432398	3824500218	4110929807
2278544862	2687463651	2801287121	2738960673	2918628374	2115369966	2641374913	2722990991	1694250582	2096166082	2684927589	5658510248	5138615796
12727120.58	7890799.236	8812820.683	10791614.89	7578635.63	5496492.492	6215470.157	9462527.806	6957256.902	7437829.715	7114345.891	2286186.805	3589889.203
288979075.7	279000966.9	323212467.8	249295713.9	257854732.4	286679244	239165405.5	365233137.4	301318649.4	319826646.1	383261568	80070199.69	439127264.9
3476014992	3654472276	3693533302	3618919346	279741656.6	3899644579	3636147695	3594942646	3985256282	3712177239	4039887261	7506513252	7361627553
95981269.96	106018527.4	115980412.9	109795425.6	90730879.16	45826384.76	105704842.6	126043787.5	128755736.3	98133762.48	71979088.31	51870251.69	91624977.64
298122302.2	370595422.5	376551932.5	358402973.7	307101343.8	320967149	264715165.3	310941928.9	353159648.9	244755041.6	261610266.4	477707644.8	428142200.7
207197935.6	263452994.3	197981728.1	210651808	246239232.9	217844428.7	688847484	582262284.4	490721741.8	556517002.8	560394300.1	313839729.7	495200239.5
113332658.6	99870009.86	77755590.19	95992812.13	78535276.23	87428823.62	432903890.4	143794230.2	189693688.2	187138819.8	116895540.1	49758947.81	74772053.55
49049756.2	58370249.75	47784862.86	31456411.79	43333819.42	39400691.19	45326904.89	66503392.17	73067809.22	60499060.13	55228698.63	77257487.33	155358108.9
98509321.2	119335106.6	104902578.2	105674621	86003688.7	109309953.5	323207279.4	221550333.3	195477115.5	224822296.3	222835621.6	70117291.7	111853483.1
265833273.2	260818240.1	286997186.3	278215222.9	253109663.3	286254971	346001579.8	290585319.8	323835147.5	295337490.8	278589526	6096465855	4370117122
4483480.589	176788636.9	299480297.7	386496635	64417933.55	6022157.146	6290917.786	9165639.496	8696356.62	9569264.836	8254467.279	500697.9375	849986.2618
241465677.6	252565396	232858691.4	241109062.6	257856090.6	289631256.7	290087934.3	279426641.4	312866728.2	336387766.1	336240438.5	569056819.5	668765557
524456362.1	443336671.5	352584731	452650969.6	432174835.6	399253659.5	313932329.3	319141396.5	301825167	354374897.6	310532929.9	1459337153	802544475.9
16384824.94	19270478.82	13649598.41	14492913.01	12127274.26	14340900.51	9926182.781	9709005.704	8349856.048	10706372.55	11182976.63	311134447.6	433290616.3
168991597.6	186923277.7	210317023.8	149338916.9	179173705.8	196486010.2	203427910.6	238027255.2	291302966.6	235330962.8	240152097.6	15235493.08	15364858.56
44048196.18	63007705.19	47929117.75	54412988.8	54825927.16	43371432.12	118796252.5	74041502.08	81346591.24	78569880.81	89200672.41	96320661.57	131138800.2
5538909.181	6835770.009	9196985.334	7927566.525	5565208.686	6618541.983	12896642.05	10561295.72	10646188.43	11887596.32	9881209.39	366071.3127	196521.5881
73569034.47	79451365.13	75068710.9	98995311.36	68368334.7	86072997.06	68658024.17	67363883.13	72300732.95	59135014.04	73577387.25	210693698.4	186641563.4
68318051.58	19341425.38	33022258.88	53300059	54923777	55624593.64	1474315.575	19658105.82	7340177.507	20518382.67	46544254.48	111745295.8	294524456.3
113498427.2	143499423.8	131838856.5	323164826.9	96006907.77	101376379.7	210392603.5	119557764.7	268320419.9	157246662.4	311158627.9	552444290	585100858.2
242652714.7	388867684.1	372848268.1	371611395	357498338.1	371405897.1	225437669.1	264281085.7	236122625.8	291907379.2	262817497.1	24241469.77	49175524.24
32409511.33	47166119.11	61281189.63	36540911.16	40049064.13	39495462.28	31368430.03	50933092.35	47892288.12	40677503.73	60774419.72	13126198.18	61897276.57
7554065.453	14164145.51	15609692.44	15942216.99	11457783.73	14485401.93	14486993.48	7704465.182	11317576.85	6389508.496	6763906.395	40886459.84	48072038.93
72246611.41	85267930.48	92703036.02	96899014.14	77138572.21	84310374.25	63716153.32	84504718.82	78110964.8	79318518.95	66457652.46	169643075.6	77175162.2
85155919.98	98249789.67	85897917.22	100959673.1	101753316	77769062.97	384110236.8	186693077.5	191892108.6	201435033.7	208034007.1	121254264.4	277995807.1
1458059.006	3355761.087	5485232.942	3998970.623	3535660.743	3770443.665	2156574.313	2584019.846	2329867.366	3245601.739	2985711.584	25214624.28	4656669.951
91208698.19	68270512.39	48506555.02	52084353.31	66736742.51	103588185.8	94181411.78	78367402.98	64122805.97	66844552.08	102507162.7	115789910.5	116406932.6
45038030.61	44959585.18	57011274.33	41897330.36	52115734.09	48197831.9	44042076.41	55364140.73	56408697.4	59779127.99	68766180.34	43008501.6	73806776.92
77134257.83	64154753.86	65577529.39	61033654.06	59861257.92	37933584.25	81695243.71	91066655.4	90070351.47	71812677.45	57870311.44	142065669.9	178487839.3
28217701.74	25939022.33	27910163.49	31908601.03	30475344.63	24798366.44	31083229.89	29980256.16	27495104.07	35835317.13	39514886.45	160829706.6	199328305.1
1095374242	827283454	1179174548	794647703.9	774855263.4	791093908.1	754351603.3	715526235.5	725364347	1149345125	783385620.7	53179997.96	58929938.7
9327615.978	10959666.2	11101058.26	9035411.901	8851951.944	10074872.51	10365702.24	12246416.02	11770003.07	11366270.87	10711979.71	10115154.18	13574542.54
30044954.87	69699067.94	88869727.67	69951445.19	60101770.81	80547311.14	40148962.62	31369343.04	38609637.55	28000988.09	24849262.51	45908938.88	36790784.72
1976501.646	24773959.5	74032748.16	60811139.65	17993442.62	24809158.88	43389040.73	15482119.24	5436956.946	765444.4921	1102906.411	16713771.39	8095976.255

all_YM1_5	all_YM2_1	all_YM2_2	all_YM2_3	all_YM2_4	all_YM2_5	all_YM3_1	all_YM3_2	all_YM3_3	all_YM3_4	all_YM3_5	all_YP1_1	all_YP1_2
5772733.79	12510146.01	8849908.136	8161397.71	8942158.07	5333222.714	28464256.39	7929119.768	14849355.47	8113422.368	6430513.879	36684598.18	7978665.133
55665495.21	39938614.12	46616973.76	50855333.05	50127993.97	47815048.35	118073714.7	41677797.7	51492656.73	43674700.24	40088515.16	501031303.4	299069348.5
99948733.56	114482250.7	102972852.7	88497836.23	86619155.34	87753652.37	90965033.69	128744014.2	127298768.2	142111496.2	102259871.9	7334353.077	7152529.501
28087472.06	25828887.07	18977988.31	15090050.33	21616322.47	27919020.74	28273207.99	32819347.12	39051269.55	36143710.87	32222955.12	2466645.353	5726219.033
6343855.62	6053652.155	4136475.104	3182344.932	5881955.579	4316079.475	5528580.187	5365204.061	8170226.778	4969886.311	1503508.845	61753464.65	10808222.35
18116065.45	30850811.54	21994624.14	21750295.08	22201571.11	10450040.27	57353298.97	33691894.75	35168931.45	35575396.18	27725627.17	1116520.917	1269487.483
61442557.68	59071546.27	52379878.35	54963862.37	55543609.66	47719450.19	113872172.2	34020209.13	49130656.84	39784583.29	31276440.43	196282017.5	254812884.6
72138137.12	50207623.09	42999410.85	50931027.84	51132270.27	51041745.35	133947414.8	67200729.58	75810653.17	77434376.41	71536525.3	151529915	184268304.4
23747253.88	29145187.2	31418212.7	44625425.53	23335835.79	9081396.467	28792045.14	26463726.73	25508984.21	17343135.62	14612668.79	286394636	230018170.6
16481586.47	10252408.77	8554206.624	10947667.62	10088994.39	12402355.21	120111236.5	46967471.71	40909316.04	58735974.71	61554299.14	52870854.76	111613150.3
13041039.98	12763890.22	17220135.53	13329434.19	15456616.17	15950120.96	14670425.1	15923949	17115777.32	17586770.38	20492060.26	9889658.894	23222580.59
3564006.53	4475654.334	6136810.271	4667777.226	3901582.104	1934660.219	4330373.657	5250262.918	7538855.356	4567857.708	3074229.567	1655388.694	4300239.348
20121952.53	30370701.47	31121166.5	20076902.58	25408509.2	12122710.26	28896160.78	18383580.18	27473407.24	37824826.3	9102560.291	664916.4353	7541543.725
25715658.7	29028996.21	40555844.67	49574958.53	30918217.84	26290192.67	30832615.5	39954146.39	40056007.54	38099779.79	29570327.95	106154457	181847499.3
53684554.42	68601314.64	53425798.81	57743885.73	61415671.43	43643526.25	218492761.2	118030264.2	118640080.3	127746464.6	129835908.1	17890784.97	24607282.46
36712811.49	35095496.5	35766345.2	36232846.14	29255788.48	31115460.8	44852964.65	36392997.02	43521959.97	34871597.36	38662234.33	77630589.54	50791863.26
8814704.725	13315005.77	12584642.05	12173379.88	8999091.173	3785713.602	10341579.69	11399706.27	12434815.21	8662316.547	6551787.556	1004513.049	1276497.692
56011990.2	49099297.07	50957309.71	52395666.35	52227949.89	55924936.04	43463336.4	33275954.64	39276679.12	37120821.47	43337819.18	20962617.25	13370707.9
8845649.41	5149614.005	5159516.785	5850927.944	8517095.194	5415371.223	4869412.715	7847702.662	5774591.81	10434612.83	16537840.48	621477.9775	617047.3899
3858540.059	5116356.004	6579876.391	8501287.802	4532617.972	2684223.663	3137555.195	4016035.576	3822836.063	3279140.978	2946654.146	6519071.14	5049073.381
21686590.25	35575468.9	9788340.203	51420793.35	26991263.46	13879490.57	18896854.17	24692048.06	22982457.74	4609320.457	18569221.66	2964244.909	1875978.554
4910378.892	14336782.05	6569714.665	3325284.688	7155411.464	5685116.229	21042381.11	10671529.56	12228225.6	9802780.277	12747716.05	13760551.48	18450167.29
26795342.73	31390090.98	33681699.85	35979348.52	23773364.66	16369861.42	25176915.31	36111752.72	32287043.71	31585761.33	24370066.95	25726943.67	36651674.99
6728372.517	809705.707	6104222.08	8923388.911	4783324.577	3076008.437	290957.6595	6648850.877	7611623.74	6359711.594	4276973.193	35315674.23	51879907.11
2109306.78	1588891.975	1665087.77	1740665.245	1378977.025	1653813.995	3855593.581	1863366.496	2237917.174	2095532.182	2074071.754	1690466301	2149965628
17261754.47	3142464.101	16651872.36	17265331.56	20586105.31	19403775.98	21851388.76	20432471.45	22962713.44	20373272.35	17803387.96	39558229.81	32594586.39
17339591.17	18848462.95	19280997.19	22530694.87	15457304.34	9619413.036	18960791.38	26872060.26	23211336.25	20435376.47	15943803.86	14626435.69	21414695.26
9535003.073	8285829.069	6627271.431	6970338.001	5449057.542	4012155.952	6071300.968	5719821.776	6786796.328	5084440.555	4799459.793	30374201.22	51097846.6
26731290.45	36771107.02	31295063.36	37027660.71	34584506.25	33196920.81	36060767.07	33065425.5	30479098.01	37917781.41	34409587.83	88164349.79	67081758.04
13050451.28	10794521.82	10578233.95	15040556.29	14551334.01	12963348.14	8763696.81	8783070.594	9222225.152	8303150.412	7802303.029	6956786.074	6029315.951
36296936.65	56168490.56	43952830.95	52861325.74	38704835.47	20051145.7	49399673.41	47010868.06	53668511.38	32363531.54	25006873.52	9954521.064	14494075.09
33339081.68	64589286.67	29584028.14	61098265.63	29774131.78	62025930.28	63429620.62	70462605.96	64960665.24	34500731.73	68941491.69	24470063.91	18651613.49
3454201.57	2928408.986	3355640.751	3924537.556	3707469.521	3626259.037	6078094.73	2862496.698	3300099.731	4605690.739	4325674.984	15183275.38	70070055.46
1286881.766	2568471.43	2991526.634	3904795.185	2565599.685	1544788.98	1051737.093	1555191.87	1989598.94	1642845.387	1407770.106	3326482.069	10592301.35
5339768.239	5906200.162	3163978.648	3704046.501	3622911.495	3041667.352	21808836.29	6613089.21	9111471.131	7853583.219	6790069.334	10501691.33	51179820.74
20932803.82	47640401.53	41744717.74	24245324.21	39418357.79	30197940.77	29188307.6	31028125.26	38269309.32	29662897.8	25089679.9	2462969.576	3568431.3
7194253.375	7338016.978	8386409.563	7546689.886	6480789.663	7617433.067	6851043.826	8458375.263	8101227.709	9163401.892	8797673.556	4090341.718	8129043.758
21338520.29	31167493.57	34235279.83	41266393.39	37283212.56	27905749.82	32984606.74	29193532.28	29295979.52	34320025.67	38178525.72	44622242.83	28526321.71
35628091.22	35801045.09	35221002.05	35387764.31	36303791.92	36367534.18	44203817.88	37793209.7	36304123.1	49037434.35	40546838.61	39115343.64	42009105.68
6959430.302	7437153.882	8114150.069	7827658.506	7183443.265	7600426.632	6554345.61	7366240.872	7828693.243	6717023.823	9070788.237	36860170.14	54378246.48
10010396.29	847348.0878	3168273.776	4274692.331	7973904.666	6447266.992	1069740.853	2714745.164	3854024.507	4132751.024	3457370.644	182726415.5	158698691.4
8546050.596	14077558.31	18209549.78	17332289.86	13921467.48	13891078.23	12248382.01	16050305.43	13622592.62	12411793.83	4732680.334	12541756.43	21024091.11
5319758.349	3778794.811	2103387.28	3434713.055	5225972.691	2027957.127	6472025.673	6584030.862	5407538.182	7711134.712	8000744.088	3284346.091	3315226.404
4106531.596	2428744.81	2143416.726	2450641.014	2192208.855	1896418.511	10121474.44	3593547.977	3813159.704	4676931.82	4475925.777	13002433.43	12923164.79
39923640.9	44236133.96	46596377.28	46886074.55	45980672.16	44422007.01	41954253.98	37601546.5	35377516.75	43647128.75	41274665.04	63404724.86	58050445.21
5491789.754	9000495.453	9851373.699	9157932.215	5084616.673	2966366.074	3928126.638	4477682.044	4825833.55	4454756.05	4386168.361	32218687.67	27217017.82
3603933.039	4543121.459	4158484.131	3099572.112	4135307.706	5724679.643	4112039.837	4892511.127	5706373.988	4201356.097	6805604.328	4185795.801	3593857.843
676239.7055	6617233.053	11579624.22	12475184.11	5645443.108	2836698.928	706501.3551	1387472.866	2332128.438	1721391.704	2122784.23	415350.2414	414537.0968
12264098.98	10790650.25	10986372.94	11622723.74	10102881.79	10068501.04	22937537.67	13028712.04	13110601.97	11919842.07	11827560.02	18370253.47	18054207.52
76494617.92	64151774.01	60530389.94	59644749.63	63143954.13	64533239.48	71492392.13	69886109.2	68003827.31	70442803.56	71914376.21	4555415.794	3304718.98
3861449.972	3297644.034	4290741.571	1958012.324	3783888.179	244296.9945	3539066.23	4165394.844	4064582.814	4300811.131	2632337.106	4452678.744	9096536.099
1712825.301	2651681.732	1462705.397	1414456.016	2110076.478	2493576.968	1927213.83	2501851.31	2776218.389	1701047.597	4895962.123	7558188.938	9614379.619
151023.3357	185863.2178	307021.6855	218035.3938	168601.2306	165817.0234	175942.8373	151929.2122	192850.4186	152769.1255	222545.8805	12240915.21	7873835.434

all_YM1_5	all_YM2_1	all_YM2_2	all_YM2_3	all_YM2_4	all_YM2_5	all_YM3_1	all_YM3_2	all_YM3_3	all_YM3_4	all_YM3_5	all_YP1_1	all_YP1_2
793685.1106	927022.1651	808508.1501	691926.2419	849975.7858	842043.2353	702792.5401	808107.7492	884644.4795	687599.615	720701.6238	1653743.449	4566622.36
5398236.039	2683722.269	3151040.437	2696118.926	3260443.787	3693282.657	2487715.545	2839675.661	3336468.45	3534237.038	3871916.192	5821947.6	4929493.627
2552633.987	15026382.94	23529766.25	25795855.47	11000380.48	1927309.506	3120357.383	7668588.926	5309849.278	3989680.026	6266817.98	141970.401	138765.7032
2413747.317	2139151.162	1855577.609	1951821.889	2120318.91	2206412.236	1321500.428	1848225.583	1833472.667	2027486.07	3340461.255	41973791.44	46266042.36
3415715.232	972680.3506	1069835.471	982219.628	901187.8716	1254468.059	567344.6915	831012.5348	904246.2227	1062256.213	1160917.376	9507303.886	18489625.46
724972.2659	1607752.215	962847.7969	1352980.515	1487003.018	1453679.952	6703083.243	4313665.296	4255744.299	4427684.084	5032260.579	3262550.484	10009388.53
12721330.75	9121236.945	8399329.858	8862404.495	8839010.63	9466262.276	6439895.996	10724846.97	455662.3915	11599457.96	8402615.433	9384885.297	30699730.81
401911.2953	373132.7933	392387.455	379944.1579	390003.1987	381850.4098	1671877.312	444812.6874	682614.21	682569.8858	696725.5177	4652493.975	8607522.646
1852659.323	1344182.965	1699315.334	1670202.586	1773383.966	2344068.407	1073512.048	2374496.191	1587218.287	1293067.584	1113022.23	1287978.593	1499853.995
340655.1226	1963165.037	2761030.817	3560030.724	1889711.389	367240.1131	325684.4118	788999.7259	312637.3352	578813.0297	385898.5502	278928.3854	296490.2484
1260246.616	679543.5413	1267689.803	878959.0905	750458.4449	898150.7133	4959054.755	1895870.484	1669044.161	2508209.976	3063623.128	19130651.95	25258496.87
1549569.374	1722661.053	1600281.105	1348492.087	1716860.223	1410478.521	1369333.687	1539815.349	1822783.56	1308133.336	1425082.539	896173.4128	1711238.075
1437479.639	1289724.34	1267838.733	1140252.365	1419448.829	1171587.373	1928663.207	1226795.985	1419186.52	1516196.563	1466016.359	12278854.01	12416189.6
1780874.501	1001813.437	1145396.82	1600634.426	1329991.38	1154618.575	590571.6164	1976306.569	889074.6794	983821.9154	612725.8162	244236.8325	242469.2059
679765.9341	167031.3144	431467.5434	716426.9201	538249.2343	390114.9422	317533.3814	5156846.465	170644.8365	397456.6237	113760.2384	3095513.421	4014994.332
384398441	436658588.1	275749328.2	369581323.1	442189266.2	346043008.7	266910846.1	596233753.9	471767848	447025416.3	581005023.1	3555807011	4217295717
2174150431	3075578635	2517936071	3058237009	2346322233	1821243822	4259138302	3167611703	3942509537	3299628648	2012001753	7192316.186	2879274.017
33267186.66	1351682873	2168670063	2519860782	836334025.7	35886685.47	44693028.83	140301068.4	47995462.82	93038747.2	107056422.4	62171976.05	55011561.15
39121022.27	67665534.49	45495916.7	31069190.48	35785821.39	35869293.51	28568297.64	55893360.24	53754356.6	31414999.41	52514840.25	15253392.79	48171850.41
1201467782	2531817275	1778120252	2115710078	1600164153	1061401352	4125613683	2870395074	2983546173	3024626838	1616886615	17139397.47	34865162.95
1773939416	1116722933	1046502088	1210049418	1225678307	1341216408	1438394865	1409309133	1097417520	1409616621	1421094439	2053393811	1713206745
284959295.5	1036143589	716530396.6	359427045.3	179202919.2	1388213293	144935110.9	191908176.8	867729365.4	459464298.2	1725297686	851596685.6	550854173.9
245886142.1	224353768.8	185515948.3	237782802.3	201937242.8	153901330	302932196.8	185353403.9	249809048.3	183966312	125722249.2	18508263.3	33955324.17
126897154.3	102167128.5	110671907	101979322.6	94506277.1	92612208.62	118117869	125727415.4	122180325.6	127386346.8	119403336.6	117820040	181145176.5
373456539.4	351332009.1	323389073.6	267454890.5	333547186	344922147	338649823.4	417133887.1	429849332.3	360274736.7	354871640.6	318600688.7	790486997.3
40744563.41	21886206.64	17833395.72	17536855.63	28733719.96	16443774.99	12403803.89	28461854.39	33839576.47	23196864.73	51131157.22	22148650.17	44492805.89
119891965.9	102824808.6	78585659.21	59816332.2	123009425.7	85167299.39	93799011.16	124860678.1	152656132.2	117356539.5	111887867.3	98962361.69	179236099.4
97690689.49	52697019.43	39204116.18	33963529.25	64562257.99	49058451.53	38322462.07	77284053.36	96314279.4	59036746.51	137220040.3	60616378.93	25595667.44
171084715.7	245205700.8	201354934.3	239937427	184267669.9	143297396.7	348434048	259565162.8	325515219.9	274598923.4	160750526.7	61102787.38	29646107.38
161212732.9	215390480.5	139863390.1	169100648	217470396.5	137740651.7	350803747.3	135813002.6	206513202.1	149705637.7	47907255.43	4660636615	5535862537
977689.4131	307069.188	455249.9636	798012.3028	391456.0363	489362.5933	97465400.5	3407654.849	1241437.811	2349714.7	4211705.54	459202717.6	241794661.1
561311.1859	436517.2527	437082.0159	468404.3379	459009.6395	503578.0533	487745.8304	432560.5104	404037.904	437805.758	415879.0342	2661342.711	2577217.765
101014991.7	289684104.9	246940319.9	87858978.86	44279411.91	578979927.6	42851959.25	61250720.24	361912202	144073117.5	447520899.5	217675126.9	242579579.8
10669218.02	15909342.41	6026108.621	28611503.1	16117081.12	15909453.17	14539299	11159907.55	10217734.59	10008951.18	11696440.07	844193.5353	8176976.35
185535441.3	211737943.1	187954330.6	227768625.6	184925042.6	261766417	118650042.8	262098995	182211681.3	155462411.6	148330786	26388285.05	17253212.53
41636319.56	53903752.7	43126094.76	51394393.93	15553466.66	12755676.84	48569463.18	58142242.89	58987649.76	68014033.85	24231966.56	10044549.42	20908906.27
658292.1511	2031831.116	14373026.74	15925244.46	2120497.187	7113014.803	43284668.34	25764426.12	33724544.87	25853849.33	45714672.19	5624302.076	23435932.29
70520458.46	106874027	65973744.6	63850589.23	105742768	68234321.2	41724143.27	124013466.2	90708090.85	74305904.97	129863844	44238569.88	51143498.82
36713167.01	40772115.81	42683734.35	33452322.45	43388263.3	38543086.69	39207511.38	48912572.23	50382801.78	41561516.97	46515586.14	33998993.93	77270613.69
23311713.67	20916555.42	17992679.07	16891000	24113331	17164514.79	14485509.5	23145679.82	22432903.65	23503241.65	35459807.38	74866943.54	56586078.67
24686364.82	38499812.18	74243112.69	65117841.75	60350177.67	70322172.1	68229183.93	95421062.85	76132422.5	91898995.06	92516905.89	32409862.16	46469319.38
11442600.77	21454861.07	14914001.72	14565246.18	16569482.37	19027927.12	15346064.9	27035270.03	28746199.24	23089470.81	22784200.67	2910710.94	28585691.09
185910887.1	162946931.8	141399779	96984324.43	139220301.4	128804254.2	136685307.5	128252977.4	138330767.5	127647124	140335829.1	200641125.8	336103939.6
14328447.22	25178773.39	17098246.27	18338650.89	27508298.39	27981440.37	15980229.53	32727084.76	29183129.88	20709076.12	27345959.33	4492988.171	10717085.34
26075698.58	68884472.51	256076171.7	176520074.6	59106409.41	59616791.83	236317178.7	386492851.6	196607997.1	175220111.8	125524311.1	3295664.171	38138235.9
94260223.07	115017697.3	103365835.4	88316694.98	76444266.54	96937647.69	121629168.5	124674747.4	127660341	60329296.03	42271747.73	69491135.66	82334103.21
191386.4935	544144.8005	842845.2275	1122034.075	523070.2145	228627.7066	163613.6299	171633.4953	183497.3638	170947.0991	228720.6885	786455.4065	1052778.141
56704222.22	84351835.14	107001893.6	109416260	134041031.6	107245615.6	75624007.72	83299358.41	87114560	66753364.88	80128638.37	115127322.2	263708502.1
39364497.33	35749296.47	21949184.92	28689155.7	53900560.63	45513419.03	32147907.75	73279148.4	58511259.34	57744459.77	69584628.63	16533069.82	30153152.1
99556665.88	90387600.29	77106252.44	115722502.9	91681026.15	88356380.38	51903186.05	48909231.59	48457437.84	40752307.96	39292043.72	451731805.1	358548935.7
15079826.8	20797573.75	18644096.98	16078808.62	20828803.61	24408494.04	7773747.833	22302471.52	14954736.04	21163076.22	35153530.68	4455093.971	11796629.66
89578308.39	57418364.52	59643451.11	45423855.56	58079338.15	57366425.68	58188420.4	80457285.83	75139531.17	78613676.35	96323779.19	136281210.1	197728248.6
14108104.56	18450511.32	11277651.78	9364748.796	15460651.48	11241757.48	5258629.866	12198778.47	11771476.57	9459299.952	15071133.12	6970605.702	8007756.073

all_YM1_5	all_YM2_1	all_YM2_2	all_YM2_3	all_YM2_4	all_YM2_5	all_YM3_1	all_YM3_2	all_YM3_3	all_YM3_4	all_YM3_5	all_YP1_1	all_YP1_2
4385409.161	3738038.017	4246995.542	4114551.395	3835958.782	3780511.933	4780470.52	4136874.649	4756955.198	4207216.344	4018082.009	1075621.055	1315492.395
16174001.26	17235719.75	12281731.79	16987332.16	11198904.25	7887875.257	10251480.58	14607071.32	10067830.03	12090035.36	8782835.829	92468730.31	61882724.94
27307257.37	26644345.1	19545791.45	18607823.36	20973530.8	18126693.54	16693984.66	16215114.72	19330504.83	14713603.71	19420053.39	6771068.545	12548851.3
36185029.26	42143680.71	42457424.44	40064096.69	30361681.78	45174058.81	22388849.51	37744369.13	29292905.18	29981131.78	30877084.88	3587162.067	5880027.265
101024739.2	118498910.5	88999023.46	101990942.1	126006042	95042622.8	110303442	82478219.29	84833347.14	72657755.92	55344690.95	1342117610	1361086458
15490908.06	12613109.41	10443871.19	10107292.1	8669678.026	9254002.24	11842181.66	9011809.361	8844264.764	9354339.57	7663696.696	10283700.44	12437923.15
7014193.31	4828692.079	6348672.855	6661215.994	5879038.889	3930461.382	5358932.305	8914571.111	6985603.374	8509218.95	5155805.034	2737512.019	6086733.879
20206408.96	14493007.17	12964235.23	10841826.86	13046341.53	13880955.53	15044756.99	14337575.67	13358946.3	13905557.79	16227671.68	41116444.06	58939113.13
29809852.92	21167365.32	25784447.71	26076021.02	20914732.32	10943368.47	23563519.61	23440694.47	20592489.87	20480940.54	14349274	41722286.71	32050581.72
6052595.366	6412991.748	5669425.823	5682721.615	18460114.2	13433403.13	8682229.872	13115094.35	12406015.33	12683325.97	14398764.08	6437425.777	11115004
20499176.85	18793775.91	27068566.3	21389634.38	22585522.66	23244208.89	21572733.75	31094094.95	24989751.5	33560194.63	47721745.23	19496327.96	32344265
4666822.302	39115568.49	74205695.94	118331932.4	43206142.52	11800145.75	3795578.054	10536134.7	7924239.325	6785254.966	6984506.969	8713540.802	6964811.507
20890749.89	26843159.35	18417078.33	18982224.39	20333741.35	20378311.82	23263748.14	27887705.56	27834022.19	25196831.31	24345698.89	9578093.557	39405426.3
107469770.7	63196166.93	78459396.03	67387705.29	80075200.82	67136572.01	71231520.88	70246223.39	74002171.31	70658759.38	57332575.59	137568253.3	167913156.5
6043041.1	3881597.188	5413245.178	4909313.906	4697317.865	4565835.757	5235311.784	5437511.341	4606884.235	6441127.048	5846575.243	4968370.816	9203548.067
15694900.27	13144805.28	11896686.33	10386552.83	11889027.06	13227139.4	11704133.7	13270880.63	12389258.12	13557376.19	14668363.71	19190851.05	42724726.72
35105478.15	17203407.6	12118834.02	18259694.01	13293992.52	9819562.701	60635388.59	16507501.32	24381778.07	16689699.44	11428121.31	127742778.6	165039795.9
49183309.74	26866923.48	17735063.07	24894810.89	33803186.83	35045644.75	14768625.22	32801109.29	28662632.77	31848410.54	38361371.97	317498502.4	264204867.9
381480679.7	473826263.3	366945681.2	258547230.3	420982153.1	480318470.6	466856013.7	536319678	11765310.59	387606072.5	393332585.3	26408103.12	46457355.73
25460125.14	14033302.11	10565866.96	12556455.56	13132834.99	11836104.08	41753257.91	16597919.6	20741117.13	17980906.93	14759479.44	123539297.8	129307002.6
415919137.6	327714974.6	372464601.1	379630661.6	437884538.7	188180818.2	458375121.4	188332806.1	160716715.8	180982995.8	343884233.3	995706226	1137367849
39380323.65	23207725.65	25952188.02	19281145.55	24883804.74	28452368.26	21984389.69	33233923.15	32146271.01	35120090.28	48357984.57	27918079.9	35777658.52
1520407.961	1825502.413	2364420.508	2521083.733	1650531.793	995456.9853	1339719.403	2207880.125	2260401.024	1956662.819	1693124.726	786587.8523	1080986.654
2144702.831	1086629.911	563148.9564	499966.7574	2800297.808	1224462.831	778424.9099	2932879.276	1528413.044	3271956.004	6060399.472	348811.0654	1216960.288
3041343.106	3880668.33	17764801.83	10032700.39	5176966.168	8402561.958	31565374.43	17900696.34	23757788.12	25300705.13	14049539.04	135726683.4	137746066.2
4365922.907	4010089.383	2054410.279	4413945.451	3936654.83	2115780.938	1788161.333	3482892.05	1951117.961	2044763.314	4297050.919	5437726.51	3392744.333
87000696.13	15353414.11	37952146.96	17642030.7	461501.6487	55861755.77	22355027.56	24373426.85	20808712.13	31164594.65	24013765.02	167460106.4	59893718.8
396844.2508	997858.0865	1154124.806	1325471.492	783142.659	439565.3921	387806.4991	798485.1864	939486.7017	698614.3569	379441.9769	4086507.527	7253153.578
14864929.51	4243597.316	6737922.806	4033858.446	13713094.52	4647239.874	5609287.169	6061749.263	5860334.803	17077784.35	6746872.418	7128326.121	13593080.51
10335074.68	16310877.96	5219319.237	18034903.71	13859969.73	7123447.592	12979632.32	15102556.76	14661594.74	14153568.82	9519891.254	4794802.959	4142266.755
8419695.274	8972542.252	9142072.574	13136308.81	6262287.308	4493952.367	4511941.595	16504560.1	9879218.201	7696143.32	7755237.495	3350916.775	3314778.74
6771057.949	66752846.87	75979021.07	93296898.63	64694844.44	6528975.547	7941965.914	21405833.28	7939237.313	16069914.93	16638912.07	5225363.936	4352741.052
58611540	59877323.15	60927771.91	54019863.91	57940135.04	59724650.52	53052238.41	62628932.83	61372543.67	55352802.84	54201991.57	61577417.63	58206830.02
63548413.58	55995684.12	38028279.41	40068679.95	41455443.01	50968986.27	59383779.76	47587514.2	48620325.09	47949924.51	46659303.79	47048412.87	67944605.02
16320122.52	18608296.09	18271228.09	3286645.375	20718285.75	19060640.82	17823158.99	20175766.37	20190141.06	18932247.68	17050386.34	104476921	171614764.3
3703641.484	5583946.502	3614601.727	2271181.289	3678256.741	4672176.342	4433515.528	5346137.794	6456218.455	3788234.156	4094298.185	3231962.244	10391928.24
14807886.2	17504561.87	30645470.26	30429242.03	19248277.44	30476590.43	12498723.96	16536632	15094803.78	12000648.17	12820708.25	49340352.09	17624785.76
4929797.739	3604620.876	4212005.015	4507164.95	3822956.536	4706147.222	6498466.588	8439440.613	6574978.115	9608198.429	7974793.776	12923457.4	8855960.64
50696567.14	40570837.18	33751337.56	48219851.64	40519352.02	32571032.58	30131584.21	35659720.07	32673333.35	32472313.58	28102210.09	461723589.8	377576481.5
10533831.05	8972542.252	9142072.574	13136308.81	6262287.308	4493952.367	6729553.828	16504560.1	9879218.201	11901315.66	7755237.495	25844600.59	23103161.13
13708091.44	16733932.55	12050251.71	14934553.14	14577788.58	16580591.44	10849105.41	11077107.55	10416882.12	12189441.65	10692446.28	2995620.637	3167731.259
9020806.36	13264254.79	13660745.89	8018873.767	11422812.33	12470254.09	8864545.152	15198764.92	14121874.3	8288481.303	15529957.31	3041958.81	11162533.87
966120.1537	8333067.149	25870693.35	17856853.65	15521838.08	1424294.959	1214846.243	1736156.434	1648779.372	4349611.356	1472486.14	2628788.486	4015245.032
3225006.178	3929362.389	2742379.702	2588067.555	3188336.988	2318262.11	2064569.062	2217895.12	2320235.95	2665510.661	2111001.337	728070.9709	2067154.516
2706018.27	7083415.962	15412239.26	13266678.99	4623673.545	2181042.817	2969761.228	5249394.087	4265766.22	3907318.705	3163057.457	1691827.19	5043305.354
348100.8552	842042.4695	519303.7473	363185.926	646913.8006	259032.87	153801.2095	836865.0835	565392.4464	315013.2881	966961.6007	895835.5559	1256568.324
21447621.22	33914519.73	34636546.68	38995042.71	35095257.09	36408847.15	28874894.35	26204787.86	25658668.29	26009169.84	28696972.22	23379816.84	31191918.86
1829053.493	2553205.677	3424744.269	5135002.12	2733047.967	720968.7721	2364397.247	2446414.314	2905897.47	2724594.454	1360076.482	3804438.878	6378129.336
2494141.76	1615092.592	1925094.583	1518648.998	1915368.096	1587698.325	2194689.619	2529178.297	2416096.612	1989826.335	2006663.422	675110.14	1097822.748
4022498.799	4025864.164	4086719.045	2991774.864	4398410.856	3586914.803	4094180.149	4481151.184	4721158.615	4401048.483	4196197.492	1946483.362	4315542.562
5384899.064	4399634.963	2926020.726	3507056.331	2957836.72	1981969.17	3528391.84	3292618.059	3340769.162	2915675.2	2132728.485	4510783.758	11498735.42
4224360.065	2476496.31	1725656.617	1601817.936	3057491.076	2156253.589	1783314.798	3458080.244	2911242.196	3833852.146	4700700.85	10807020.97	11780023.74
2340835.971	2762249.959	2781957.739	3101804.586	3050364.18	2372900.063	2550896.526	2312699.568	2903062.533	2382922.135	1665342.368	644840.544	1128167.977

all_YM1_5	all_YM2_1	all_YM2_2	all_YM2_3	all_YM2_4	all_YM2_5	all_YM3_1	all_YM3_2	all_YM3_3	all_YM3_4	all_YM3_5	all_YP1_1	all_YP1_2
15962915.05	4668189.734	6851810.714	8304282.632	9045936.281	11069646.72	6073128.904	10520956.07	9353682.683	13377306.6	19181493.38	3351976.711	2928313.798
19171838.55	13204324.92	14060200.19	16055264.33	13093958.17	15926623.97	12612547.12	11514427.8	11135145.22	13896022.77	16242289.72	106222632.5	66340767.04
8743146.74	7550045.304	6264380.519	5598113.567	7464961.069	6135358.282	9160860.701	9568658.808	11351946.22	10053228.73	8053064.334	9473079.896	23244500.45
51384539.95	43353285.81	49991135.75	54252889.6	45859701.09	44014989.75	38845582.73	39281350.13	38186072.86	41360815.16	38102586.74	972301.0338	1225413.894
1252944.55	3115586.272	2783182.973	3065619.084	1939435.483	1850657.114	1310694.145	2504270.533	2432208.644	1854764.201	2084107.444	314460.5676	3182298.15
417689.1477	14708387.03	35338683.35	4164359.179	19502816.23	1571300.71	774550.4641	1652209.878	1235224.669	1274272.977	1223053.965	752078.5965	745131.7756
29813777.93	35016917.77	25864584.09	22046907.03	25358073.27	23436494.42	26430248.56	25461827.99	31898103.99	25432535.15	25608768.13	3862699.098	8417554.245
14578665.81	13471309.39	12784098.29	16810989.33	15400175.54	14859284.38	25770607.03	18836667.81	17417113.4	20192417.57	18982003.07	11675286.83	10055986.75
17224053.27	24165715.72	44769187.91	46213600.17	55290387.32	10253780.89	30704934.57	32946603.8	32249910.33	13330104.61	17804155.3	4573200.558	8049666.129
19365611.81	25280428.83	27140631.44	31690971.86	27608090.26	26976936.11	22905428.58	22399499.3	20183140.3	27599593.22	24365279.76	37583731.15	26408870
10939754.41	11010912.11	12446832.88	8732701.216	12607309.35	14305300.13	12078063.93	16455772.96	12656988.64	11044473.58	10443358.1	18439925.25	18354834.89
8379006.147	7091204.698	10175246.42	8428065.794	10262669.39	9392393.738	8785209.284	9274333.459	9792980.951	9541397.73	9983827.701	197550706.6	424904388.7
2493462.285	2642104.559	2788212.816	2298401.37	3191877.146	3684184.964	1407701.056	2986495.146	3123565.445	2548413.022	3918236.224	1214796.261	3548977.131
20622861.61	26075226.75	12996339.55	18360401.83	18026294.37	16900730.92	34749118.47	20069698.01	32424052.97	19700833.33	21000909.43	21601435.14	172004362
15911224.93	12880854	12092586.45	16990306.45	12357347.81	19279878.57	8816308.181	9945595.063	12397224.22	8417395.909	9993259.863	10508687	10385578.99
8838487.134	10201610.56	10108252.22	7131603.134	10704341.84	12602588.85	10794793.34	12914518.89	14272763.68	10232430.31	12456843.88	5638129.189	10074478.26
3969921.701	2009365.565	3139887.99	2515828.296	3035646.723	2483539.184	2408014.999	2656350.334	2460638.312	2914547.676	2288543.586	4731568.827	1319854.752
1442507.451	6336013.618	3446943.945	19507487.83	4558447.671	1690727.778	1627947.283	3034357.858	2749315.533	1968235.431	1732940.735	3361607.88	3667091.903
24021411.07	31776059.68	18926478.21	29525026.82	31969696.25	27056031.53	23389899.84	35554702.56	37136736.81	27893510.82	32644164.1	205300120.6	318762226.9
13770525.78	11854753.2	11684891	11532114.98	13023286.04	10843424.68	11904698.57	14581329.15	12357778.1	12036783.42	12960031.03	12299410.46	18793054.37
2142730.687	3214267.644	3879900.834	4740311.74	2936096.109	545757.9896	2131141.081	3387139.398	3025949.592	2921190.595	1983961.839	183310.0098	211268.3007
3889867.449	2828973.961	2073250.582	1799506.401	2224298.711	1972390.599	20563355.55	6519445.758	6897645.407	6391619.806	6171960.09	16374404.05	43906204.06
7442215.462	6160333.145	9110823.444	9956699.014	4732719.56	5613099.236	4621131.29	7298723.257	4932859.246	4741094.343	4602099.353	8930190.368	3622448.073
3668003.782	4119244.349	5094612.41	5418784.552	5074568.776	4448529.69	8157217.462	6840751.137	7331172.067	6859293.65	6153980.557	7773529.961	10751151.28
21332475.67	15861335.95	15905935.74	13993715.46	16765734.78	17844213.36	21705172.71	20229328.93	22727783.73	21402128.26	19260029.03	17861771.04	24159152.08
2122060.791	2190860.38	1349634.95	2320024.281	2826960.186	1274059.648	11304561.07	6870413.839	9131354.032	6370563.097	3443273.941	265916.6949	294545.4046
3616506.556	5163954.706	4689820.285	6335160.934	4221973.344	2022146.34	4590356.646	5093665.339	4838462.548	7546076.943	3643234.572	2295361.503	2566914.996
704166.0337	539190.0486	845915.634	1365971.571	1696058.482	1995723.785	1655415.127	641186.6467	563662.1499	1669607.966	1512281.456	26596494.55	19984809.53
44434679.52	26317095.86	27735289.5	37542756.26	30187289.96	30224286.81	32794818.27	33530890.86	27613131.75	38001215.79	35956053.13	80797142.25	40099965.63
12327019	11825943.56	9994147.18	12359247.83	12663180.82	14445397.42	12558532.5	9008196.994	6422628.298	8269046.977	5912369.209	250660803.5	275482318
6298287.237	3954686.155	4419173.791	3855734.618	5086318.949	4584625.622	4261816.704	6872861.853	6516980.987	6389696.379	7403318.058	6784913.09	13423148.1
188006.3619	200767.4602	188683.3492	204725.0358	159848.1434	146398.6653	127764.2444	158930.9036	167666.8469	121494.6277	138059.0243	165653.5261	175068.5982
9386444.604	10138794.4	9210557.87	10308964.09	6885297.391	5011206	10283956.95	7544026.59	6972879.849	6203209.97	4774010.739	10157350.48	23584124
6935718.317	6041686.584	2995996.078	5870084.181	6340802.674	4789358.922	7034727.392	5314636.599	5827139.049	6668232.465	4115205.237	6187448.01	3121383.365
8571202.546	774175.6262	1410158.333	3201591.009	7239909.496	6803099.481	407423.5067	2338398.559	1633595.671	2641247.635	3798944.854	22223303.7	11305403.5
649913.2199	367710.3744	352602.298	412995.9852	391478.5638	446141.0431	457285.8676	373982.6642	358445.4661	437822.8694	914968.4029	47105494.42	30467446.82
328748.5909	266381.8983	270913.4579	302525.2272	298789.6915	303904.1479	288786.6272	283620.6243	289099.4677	281113.7415	266790.4763	3305381.521	2312501.651
10124613.29	7097389.258	6071726.781	6366102.667	6631114.048	6844150.083	6628973.689	6202059.147	6128797.802	5950236.106	6006802.299	703812271.1	500523944.2
1678260.847	1514401.933	1326023.981	975680.3151	2583746.53	2294131.158	1289644.428	2984626.434	2411757.203	1166622.891	2520366.338	294357.8088	1212084.771
1273445.328	1790865.665	2042113.742	2137631.518	2513853.546	2126664.445	1849875.853	2403951.06	2316415.374	3072934.692	2477291.522	7813708.439	9720441.278
7150657.092	3548786.666	5002235.64	2537421.849	4161392.387	3743560.808	4233594.969	4961337.774	5413809.231	6150708.986	5416677.211	7785821.142	9975248.132
4982375.355	3927417.635	3857992.816	2212633.289	2726985.894	2551342.071	3082805.899	4170656.18	4362235.366	3462507.377	4005269.626	5803035.95	8626942.162
696716.937	248533.4364	304168.3389	471733.1957	563141.7926	419811.0996	294274.9739	512865.7863	487564.3392	441075.1355	1022660.985	5383962.088	7044492.019
3062715.137	2242029.089	2813522.783	3946710.145	2827886.096	3225494.811	3798055.485	1560483.908	1772694.684	2353899.551	1952886.012	17481714.22	17990213.53
189267.649	187197.5422	184873.9352	179943.0625	170194.6677	226096.5637	161802.2594	169733.3366	181465.86	169054.5396	226188.5161	36563997.25	56466413.06
717523.278	513994.598	919357.8212	798305.6674	696545.7837	426469.8185	528725.0346	875128.8309	802245.8463	791947.4547	727252.2186	5194391.96	5027496.519
835617.6135	5838857.644	9304188.537	9431318.062	3954110.833	1983930.732	616491.1583	2410640.353	1660360.546	1217030.677	1847916.449	448028.4657	2128016.014
2306326.983	3575611.968	2680975.2	2940938.908	2051056.625	1377363.945	3120392.083	2285460.577	2645257.133	2388339.037	1201764.54	2740025.774	3501181.891
8874449.302	6994989.417	7129868.065	7282684.12	7904457.601	7481735.397	3813894.987	3891740.174	7753932.838	6335188.153	6672847.784	1250489659	1373730830
5288507.653	4753279.803	4509274.331	3767159.66	6172639.46	3932754.908	6214771.277	5173953.281	6449926.84	7243814.269	4992040.233	3889356.985	6253745.413
10903537.39	3426512.024	7786613.236	11507161.91	10577745.76	8950911.678	888118.5509	7377574.514	3775967.38	9805138.409	8670227.569	61342080.87	28501058.76
32839010.6	17281998.43	20904781.06	26441863.87	20188426.15	23337257.09	20683233.76	22760778.88	20042224.46	21142490.76	22288176.41	215136078.2	218645619.1
147575.6796	119689.8362	118313.9125	122880.1984	128343.8536	128077.6028	120061.9521	118948.8081	123450.0901	115792.3287	110520.5063	240628.3836	258827.6557

all_YM1_5	all_YM2_1	all_YM2_2	all_YM2_3	all_YM2_4	all_YM2_5	all_YM3_1	all_YM3_2	all_YM3_3	all_YM3_4	all_YM3_5	all_YP1_1	all_YP1_2
3203014.208	3093741.202	1691375.647	1928860.945	2654991.743	1703325.844	1430813.311	3555344.307	2667001.415	2515758.341	2999027.03	3667219.655	8337511.674
4749976.017	2694154.117	3026926.734	3274128.232	3729379.463	3202332.161	2050118.063	4474078.582	3361146.742	3528498.127	5073043.306	7964370.015	5566092.268
12066568.34	11730963.17	12773875.46	15067003.92	10250809.74	12610517.7	17968947	14619990.22	15505291.46	14505799.67	14364505.55	20997005.43	92302214.15
17140631.06	16802544.35	18068035.36	15334888.94	14854367.98	15385376.41	21227584.78	14133288.64	15517466.23	12892242.56	10279316.77	13579266.88	53587758.7
445738.3364	426306.0836	431514.5538	1112096.693	412272.0919	404772.8865	390864.4773	393050.1725	410220.3054	372851.4679	358604.0535	7738755.596	14091845.2
2370798.563	1930462.849	1071844.453	2846304.431	1504820.784	745243.2726	2176613.02	1832826.46	1530298.141	1579095.167	774473.0831	1466734.939	2718212.977
393127.7537	351126.4877	354596.0055	278410.1758	304732.1435	270724.0675	286977.8683	298917.4517	514881.7903	346465.6388	434554.4097	1918595.474	6218849.12
7179672.574	6312040.631	7009378.794	7567484.564	4375118.17	6544142.423	5541188.711	5434036.962	5159638.451	4478284.871	4837790.171	10640605.25	10823831.55
1624040.904	289295.6586	401714.3081	191086.0464	2073311.08	326599.5747	154983.8921	514177.83	248935.3839	2714305.349	256924.4605	344353.9471	594600.7627
390528.4834	2931683.418	5126761.553	7570953.311	2690816.707	396320.43	314368.2756	624355.2426	722174.0375	1122373.034	511728.4966	11823663.36	11063663.47
619568.5009	1827158.847	1345678.263	2332953.21	1564105.922	1310399.906	1723180.397	1430492.813	1509846.451	661472.7164	277651.9447	312315.6333	313163.2132
5128225.392	1396977.028	1882867.195	1958214.886	2587330.051	2541479.415	1002707.278	2893564.152	2443017.227	2139327.441	2474576.981	2311617.727	10320015.23
675768.6847	1239394.228	1057672.054	1061160.78	908460.0108	814466.7721	3880554.333	857280.168	1375758.47	888533.0484	590075.6635	9546595.197	3494836.721
356164.6771	432948.5391	11715861.01	1245848.657	3633671.956	304603.8988	323572.2561	304317.6878	324409.6601	283041.3439	290718.023	798678.1602	431980.7636
318186.7075	288109.5771	544021.3398	407787.7106	279368.399	299558.3505	322301.5337	272796.6588	307174.7784	286625.1243	269450.7952	482208.8808	615288.755
320856.1368	340092.5254	322733.9439	312121.093	313048.2949	457343.0235	286619.1863	278830.3218	364640.1874	347052.3391	340590.0344	303103.3403	297240.6817
5862702.26	1729619.636	2396072.515	1964015.465	2881185.941	3323797.613	2951331.853	3514985.136	3484290.499	3923444.814	4631142.925	12257413.91	12312352.46
1572153.254	1281997.444	1587422.311	960978.1955	970834.0794	977956.3964	1444813.702	1529096.55	1799568.138	1190403.802	1073085.138	1299480.996	2382300.332
2499319.421	4323256.643	4970258.497	4251324.678	3685737.845	2882123.046	4376499.829	1907153.128	2624803.444	2221390.7	680572.5467	1872109.972	3420913.235
1697410.638	2730162.522	2387693.869	1764382.776	2177843.83	605504.8326	1959326.215	3020499.907	2856846.788	1170428.198	610751.4117	327379.168	608109.1447
1484241.374	342514.4402	424658.6385	533093.2785	513271.0211	457804.7673	5665543.299	2002113.894	2161095.993	2396318.578	2442170.173	467976.1917	453017.2917
443528.502	1513395.159	2312502.453	2657531.743	1371054.834	1562151.452	367298.4108	2232690.625	633953.1924	403929.6918	1772295.497	498356.1568	496659.0682
2921663.082	1852497.379	1640812.535	1595773.384	1587319.679	2198459.487	1877295.847	1690753.048	1775525.949	2640121.558	3076335.666	6101899.041	10120468.15
14293052.63	19115024.81	17788278.78	15564913.34	21756680.4	17269043.72	14371545.97	19712093.9	19542142.22	15656110.69	17493787.93	46028816.9	55734127.83
5889621.543	5230496.974	5533068.902	4763114.433	4737600.735	5541292.193	5688633.596	5522934.807	6355419.073	5485101.117	1012755.519	19001550.68	24198718.05
2683685.332	3550197.326	4451270.844	5282132.443	5556914.178	4764596.234	2246025.396	5913978.482	4260163.895	5234400.698	9135592.818	198523.0219	158920.0839
8308389.294	6892200.884	7458065.569	7940131.287	6997435.262	8219430.004	8057325.351	6663243.838	8847149.386	6184556.254	7570090.811	9844329.495	7864076.369
8361718.231	5593429.004	6545308.168	6317800.935	7200203.511	6712951.763	5617430.139	6631819.254	6055033.084	5846793.38	5886076.139	18938866.61	25019079.56
1094073.096	1290817.818	896334.9465	857534.2346	1249651.146	752284.3521	2296416.191	725003.373	1334804.6	1012397.022	478422.3777	49700962.84	42505884.62
355530.3322	254560.9227	255445.4699	286412.3493	269472.3236	318962.5952	295096.6699	273980.6115	255914.6046	277302.9123	258339.9116	8331712.613	8143519.948
1026224.715	1518074.444	1261621.258	1008698.083	1996440.654	2210637.493	1932561.976	2138071.874	2325443.608	1338384.787	2123832.691	297555.186	781509.5484
276412.0614	226119.009	230316.864	244917.6074	242078.6952	250173.8266	250083.1611	250585.7833	239937.4121	230391.1579	228684.9506	228770.8758	2678373.911
2084822.274	1674018.375	945831.8171	1205255.497	1828505.512	2440556.7	3276792.664	3302797.313	2874449.574	1100938.008	854778.4678	39242614.13	48845143.12
1146501.458	818695.4474	727376.6271	866440.4402	680462.8373	626081.9222	1114413.03	1047193.93	909301.9045	1190524.354	1083843.907	3278600.044	4163640.335
421194.5102	345772.5999	366812.6017	539856.1868	389571.0278	388676.6634	947985.7788	371333.9643	369742.293	359528.9891	341210.322	257318.5373	1207439.296
2594624.498	1724023.532	1881137.206	2159270.255	1876367.369	1811418.026	15281394.63	4546321.348	4532089.443	5284852.773	5232446.209	22946287.21	26081014.17
1596432.879	1405323.985	1447854.349	1713520.464	1427775.791	1499080.51	278408.9982	1099971.656	921702.8619	830609.5578	654542.5986	224646992.1	308813950.8
177500.3335	156292.8516	165577.0329	169433.9916	165885.3812	148328.4388	156626.9543	160086.258	156145.4624	140151.419	144295.7783	992220.068	1471901.134
307380.9285	358492.4853	321536.6701	239378.838	635641.1458	446675.3037	172261.0533	581866.7236	455448.7166	495855.7353	714099.7164	167081.8697	239619.1172
1332337.72	492127.2405	1158953.778	1597337.043	840806.1274	979164.5407	886535.6355	498005.7289	619727.7531	639283.0139	615776.6066	564520.0285	380055.1512
2222575.076	1634812.131	1379085.202	992017.498	1960210.595	2083495.198	1327850.646	1333865.918	2448654.612	1398183.433	954823.3518	2383842.528	4857328.328
2550465.899	1696482.981	1559498.332	1502678.429	1450060.958	1618403.153	2208565.263	1527063.464	2305467.382	1663308.744	1451068.442	861884.4046	702416.733
410115.6637	284145.3911	446459.5586	239077.6169	339734.3137	609075.6851	556056.1929	787581.2862	728555.1101	1176258.033	951438.7296	5210473.593	4632175.954
2000461.94	2024551.64	2353156.657	2619212.771	3056410.154	3313156.465	1651236.035	1612089.704	1492572.262	1735516.018	1324945.03	5013730.435	10619778.91
1699138.887	2273037.083	1512073.997	2018157.736	1044272.315	1513815.85	2900615.354	1858811.996	2541280.299	1964087.572	1958140.203	2603877.668	1292186.155
1245508.743	1094275.227	1021483.445	1080557.554	1215398.287	1032016.972	636945.6019	1298038.425	1184574.21	932453.7586	1555248.89	19412928.25	14336829.47
2733943.19	3000809.011	2334043.881	2466437.47	1857947.441	2551831.486	3876557.288	1954296.737	2196830.454	1080360.083	973270.6315	1987674.285	9066327.86
1884410.832	1876139.361	2266534.602	2861732.299	1790767.586	1270494.762	2611517.224	2297546.167	2268412.264	1551381.023	1186390.356	5924093.713	11963027.09
2368681.638	1689767.413	2544748.038	2705505.42	1775945.192	1703375.236	2773151.412	1939349.787	1999608.893	1806545.711	1064147.453	1018360.206	3497909.245
16318395.66	6598154.202	8687236.24	14078951.01	10277754.3	9970543.554	7052280.649	6223657.165	5782635.703	7010017.741	6553640.207	72405318.61	42809688.52
8604895.379	7483522.147	7312705.285	7877581.676	8207553.048	8255619.689	11674158.05	9913576.964	8333671.709	9554859.137	10269831.43	15707171.59	27844958.54
298540.0835	238976.8771	519552.2309	470019.9559	284270.6082	259969.3542	258363.5735	259760.5298	249011.2546	237144.3262	233474.3219	920010.1761	572066.9191
411379.7748	561257.5338	488816.5269	360406.0991	444553.3097	480948.6807	324323.3127	477252.11	448999.8347	320112.1936	430735.8219	654740.7285	852210.2272

all_YM1_5	all_YM2_1	all_YM2_2	all_YM2_3	all_YM2_4	all_YM2_5	all_YM3_1	all_YM3_2	all_YM3_3	all_YM3_4	all_YM3_5	all_YP1_1	all_YP1_2
474998.2382	363757.6919	278039.4022	119735.032	1238918.285	412525.8488	204648.1716	319627.729	493533.5752	788240.7067	250799.75	242508.312	260044.4783
675316.3041	274966.6831	357554.6661	298649.1782	291070.033	275356.7372	260354.983	278358.2528	273134.3416	257058.7547	279226.9086	3060623.06	4171812.204
10927856.44	5888993.923	5526605.64	6657719.484	6202888.013	6364984.454	6428250.633	4982876.632	5405552.738	6852697.575	4658801.622	10992259.22	5170368.029
203284.4179	164351.8584	162039.9271	179694.0051	174277.4219	180419.0422	164898.9788	169744.2158	172795.8717	161028.5587	154419.9493	3730768.612	5533462.138
400158.2424	183222.4904	210574.9894	205569.5314	204655.7844	204250.6517	191287.2651	192829.6638	202231.5538	201679.8596	215804.8059	1211242.429	8176287.349
395760.2149	855600.4017	443045.6698	1051709.102	776266.4182	241153.8864	435454.6194	234418.8536	235980.8891	455749.2661	466245.6673	2457023.813	1474133.346
204717.8999	213368.6762	172388.6401	180172.7703	176861.7081	200371.9256	155805.0202	176317.1115	193716.5957	161993.4905	228020.3823	457100.7024	562001.8396
1705163.334	1351046.591	1235095.94	1047785.579	1428845.224	1344276.326	1495362.318	1336604.297	1215404.872	1327177.849	1451882.98	81422743.26	77601506.47
1365653.238	1332336.296	1360767.752	1495812.885	1393343.297	987145.0823	1228015.058	1340491.822	1105752.078	1015417.683	950590.4272	1962295.608	3433663.437
419516.8413	190568.1744	210111.125	125338.5346	220019.1044	267060.9985	471762.6252	255022.8534	239345.0301	292060.3504	399305.1327	571225.4432	854307.6144
618047.7167	371012.8783	487548.6044	569492.4341	538642.6671	360633.1824	342215.5931	348347.0135	336838.9506	327362.5589	516009.433	317615.569	304212.7961
1645595.739	664431.8612	624868.9115	4153600.819	557587.1364	4043201.246	772182.4928	869445.3469	906269.6546	958934.7163	883433.6435	1216285.968	18005229.37
5064325.098	5307318.201	5382562.907	6237539.612	6822757.294	6041371.277	7902979.338	5591430.505	5487992.665	5894231.344	5146832.397	15292619.25	29712278.44
468107.9158	58918.40935	59909.15349	65344.72704	66669.85133	64654.00791	62023.23968	58820.02744	59947.699	93559.83712	126803.5913	8989608.814	790605.0437
214745.4784	197530.2356	186287.0204	192539.0914	218555.4488	184648.31	185426.4033	217448.5655	202933.4579	205981.4121	169007.5988	337289.6336	589727.9305
1111077.231	643151.3712	652437.9467	840556.2445	1163009.351	516674.4522	209970.0691	1165282.062	1076361.114	1046882.035	1213334.086	144850148.2	147908588.8
2346747.675	8872515.163	5416720.755	805224.7284	7899994.323	8132881.457	4978649.636	5590234.305	5183627.217	4689764.379	3751766.387	7993209.801	7675235.102
1197406.84	399048.6657	806987.039	913744.2417	901959.1983	1164980.706	922005.0725	691890.3586	696786.6307	752212.0772	808160.6839	3048668.672	7106354.891
1682250.599	1280309.34	1546568.122	1214134.59	1250887.363	1099249.072	1590143.656	1500681.966	1444046.742	1054949.903	886475.7905	2564915.241	5702890.234
1971950.295	1571390.379	1290242.829	1996868.142	2208520.663	2587915.259	2498558.278	1834483.952	2006596.364	1185138.578	1752562.829	2826261.117	4471637.97
92732.41367	72670.71802	76965.66547	81178.25825	83137.77879	84195.38521	75696.74253	82042.80619	79207.78721	74568.19831	75915.82553	236152.863	1404169.497
255381.526	529173.0001	477151.1064	216546.8756	476081.259	224146.6393	111595.0852	202614.6658	433200.0563	402665.9128	267912.5437	463961.5487	556207.0855
373900331.3	235853959.3	278428102.5	301532338.7	294965726.2	357119237.4	272595376.8	240689850.4	262424518.9	307466504.3	315838463.5	1862724111	1419608540
5786661108	4875950162	5332320475	5766106449	5635466742	6591020010	4788627773	5236702898	5489634938	5302954662	5419818972	22431527025	3585210177
9567721283	8646449871	7764694067	9408079335	9337668948	9636286954	8773427509	7757648200	7339975587	7720652995	6722746639	1768843571	1565847601
568456249.6	253839541.9	255447538.9	298756628.9	315056038.9	319532324.4	449186381.2	442873794.5	368870772.6	509390976.4	506032828.4	1665639786	1321238726
24616347.43	8351871.926	7852210.516	23254176.65	7406062.032	8034922.898	6009128.86	22093463.57	7385795.904	5935568.209	8996827.562	499639587.4	234042040.2
5661088767	5880316562	9601176687	9608613001	6177550941	7996666249	10580831182	10471121173	8807247333	10841917506	9794398378	2358493854	2752614808
245841.4246	946062.3786	690989.7785	625709.0794	257276.5966	820953.3585	333133.3026	289861.9324	316329.8308	289869.8744	773691.3773	680691206.2	719900869.5
185535441.3	211737943.1	187954330.6	227768625.6	184925042.6	261766417	124302061	262098995	182211681.3	155462411.6	148330786	318443696.8	356542887.7
323276110.4	459548210.7	350375468.1	299101706.6	332325698.7	300035276.9	264651020.2	357429590.3	373494357.8	304394324.7	257892778.3	6253659.477	31015861.31
76631874.98	90710562.69	77462365.41	71405773.91	75406840.25	76038456	57262768.37	94062111.92	94000401.37	85367499.37	106125623.6	13452091.79	59094548.12
706869674.2	1012097193	1118841864	1114803716	718695233.7	702801929.2	726233862.2	836908533.3	893456758.7	984405118.4	706718549.5	136455625.4	451461763.8
6093292757	7941959665	7592078108	8687171262	8105131079	8594113267	10127557140	10670670727	9850885877	10830112749	10083210649	30709052537	43788200167
163924296.8	69770578.3	82620642.74	82807943.38	73586809.19	102255394.1	105097852.9	107519598.6	96021266.38	111327664.5	136648824.7	362222004.2	165014784.3
45006225.08	32043575.18	55142245.07	63785170.02	88776295.78	46533170.06	49048605.25	50245052.29	48928688.73	52714362.19	40416222.72	192005501.3	293124944.4
24982000.39	11977004.23	15978568.71	30179149.15	20316883.3	7557948.791	26028313.74	27669847.68	22348838.59	16877798.54	11544028.34	54863082.39	18880602.06
10721599.36	13286005.01	11619124.03	8150899.272	12397493.65	10253357.11	12041025.35	17814237.78	17351191.33	15122793.89	14616179.87	2425322.693	6084367.98
31659786.88	22284634.53	56074955.25	26953045.93	27271905.7	28807209.88	46339978.36	41817245.92	40678343.25	37890084.17	36465616.19	42395093.51	170080917.5
108238642.9	71595061.63	94528773.53	92545023.96	150442.2288	80089256.28	52413218.81	70275487.51	74749559.49	74431194.04	76679357.55	234818301.8	228084203.3
35893212.86	7685603.923	4410337.851	12234943.29	5836029.044	4141865.347	11632306.05	8861669.182	4522714.782	17720371.44	5278594.054	863798.9114	332282.1096
35636504.96	30855314.65	31120087.62	30414012.42	30548028.22	25935820.36	33377328.19	39682071.59	27938010.29	40586865.09	31454610.78	179993652.1	130526871.7
239527863	666631365.4	453472006.5	561672136.9	421044641.5	327528081	936142364.8	647711117.3	648118001.4	773040208.3	367582012.6	3569668674	1865643258
89654305.9	38015004.34	71001223.38	69245246.44	64801907.13	54310602.13	56236012.17	70210319.44	64383048.39	71802896.65	67774286.39	44884438.81	18642729.01
13448866.99	12434147.09	12766546.93	13875961.94	10827601.43	4617588.086	10707954.02	14346850.61	13741179.49	13498260.55	9124447.712	27158249.88	34183252.73
313092396.8	274665702.2	245411297.9	306516057.7	287908403.9	287831362.8	233768976.6	252801811.5	248555205.8	260612027.2	255900534.7	1.47667E+11	1.35631E+11
1854048298	2053382502	1480939530	1241431096	1813266713	1569677763	1387172011	2085749889	2516240776	1507778321	1491345701	504360571.5	2432266071
34735836.09	6555665.088	41198386.68	46127599.81	45860836.44	33371632.02	50000079.03	43549170.36	42825382.12	31947455.92	39907840.43	17873496.57	30526350.75
34735836.09	33217387.17	41198386.68	46127599.81	45860836.44	33371632.02	50000079.03	43549170.36	42825382.12	31947455.92	39907840.43	60045669.65	127873582.5
5592833.12	3139640.202	4087748.433	2973912.396	3095448.336	4990756.688	3147844.998	4270333.264	3931283.772	4891839.677	4944108.082	56011759.09	53819567.81
321017610.4	254694280.7	282749670.2	247268806	275754559	382482648.8	352340360.3	211795365.6	286493915	211191246.4	132634915	48969037.24	159522373.3
409698242.2	86073876.2	165570479.9	268037300.4	237209184.4	422910064	32847851.21	173515006.9	77272193.24	181782414.3	407772231.7	81970606.15	215502331.7
68171531.52	81367258.06	58361271.81	63876788.37	65481753.22	48580675.37	88953974.42	93017390.11	95731717.14	57130771.39	39113881.05	7301352.027	10932964.34

all_YM1_5	all_YM2_1	all_YM2_2	all_YM2_3	all_YM2_4	all_YM2_5	all_YM3_1	all_YM3_2	all_YM3_3	all_YM3_4	all_YM3_5	all_YP1_1	all_YP1_2
776152.1306	4389014.071	6231997.327	5492609.152	3351919.928	2768151.742	606244.3208	1070514.936	1943001.343	1188790.249	1505451.046	4627944.704	4674342.834
24956578.88	3837551.827	7540876.737	12183097.07	24271245.75	8334090.096	11491544.64	15614439.48	10178757.47	21938837.2	10761798.95	25575420.99	21558596.03
11871037.27	9756135.32	12724430.37	10351706.81	10966919.3	9748171.879	8639727.567	11208878.93	9478736.106	10589558.63	12541487.25	7777951.386	36180306.17
29594687.49	8902834.486	13927987.71	13772597.84	14228631.9	16980988.67	17267781.47	14293745.37	12307462.46	18094146.16	12088614	22234900.61	12689183.04
401486948.5	500188772.1	353725744.8	328125448.6	358192404.5	401667751.4	363630975.1	465887195.1	671540933.8	371588097.4	356571994.3	44163237.7	382194709.3
14238015.27	6388209.597	18713963.16	15554036.09	15566539.59	19165954.49	7109624.564	7342343.675	8834545.05	20366866.64	15760643.24	196668845.6	118086853.8
603285422.7	674459035.1	685467146.1	496440435	730696726.8	645086377.3	665178663.6	772050098.6	755699542.4	675020875.2	614727750.7	22932696.54	38252450.77
1279236.866	6597208.212	7698935.119	8346978.328	2426426.383	1174316.347	1106824.071	1641443.416	1443236.509	1232073.341	1395065.92	941246.4264	1901275.206
21918415.53	9013082.751	7504779.181	9567086.389	10824424.43	11335028.61	12431917.83	14597328.04	13607957.5	16507725.72	19817032.57	152790892.1	56817690.02
25826538.44	18995131.26	17499090.37	23929391.09	20126874.54	20715793.22	73873194.27	34936196.98	33410483.09	43186152.75	46225751.37	52641086.7	70723548.71
22340942.06	22224369.6	19775496.81	24849566.51	25527053.9	23730301.63	26281527.76	24815530.41	23720831.3	21697624.03	21175611.16	10203920.88	23076604.98
234546.3064	192782.8656	197441.5423	213039.1626	210196.2446	209480.3413	208659.1355	200632.953	225488.2176	181432.8262	175113.931	671544.741	670549.6992
41190575	11590472.35	21408042.35	62549083.83	95973231.74	37229712.03	22557677.63	15588692.62	68399909.28	86315289.54	99481334	26058710.34	11021017.11
64176528.58	52920868.57	50063795.04	57191985.3	54205536.74	57919588.8	53645790.7	77697708.73	46580077.21	47105246.96	42460403.44	60005294.36	114671333.2
305425955.9	410062407.4	315892027.1	307609538.6	417706819.7	419210663.7	304890142.2	471089924.9	419232862.9	349712673.1	391134434.8	166406299.4	247953108.6
102192530.8	90745765.07	85907884.85	102044932.2	89891645.16	89958639.86	87661316.78	88959212.99	79252083.38	73473816.66	69622294.78	234307283.9	243798142.8
6492656.89	8162047.991	7317628.856	6450239.411	5624235.41	3696326.666	6974290.197	8040040.656	8639354.311	4739118.015	3552254.48	394572.6768	1042123.847
2905687.773	6013442.64	6669730.489	7800319.71	3870919.964	2499998.728	10400424.42	4494213.324	4885301.986	6797010.27	3563581.108	2695197.083	1600637.694
9287709.101	8391252.145	6142155.537	4190822.817	7303864.597	7087575.566	8764562.817	10234636.48	12725732.6	8056820.028	7390387.775	5649762.751	14654553.39
13323470.13	12985024.48	17511214.35	18218372.69	16048518.11	11268083.09	15959300.78	14524635.08	16484636.26	11979416	13828012.2	4724431.664	23806631.32
45059467.38	31729929.8	35118378.93	43520092.58	33968246.52	31875293.82	24785704.39	25792527.49	28823411.31	28499526	27035187.75	181591288.5	249952475.8
6222393.199	8730161.776	6322703.654	5145335.182	9136598.745	10114789.82	5642044.135	12458259.29	11586736.35	9638871.685	10501790.52	360800650.4	587330836.2
50198695.58	40245749.93	36247292.64	26206938.41	45127902.29	37345849.77	43869301.12	62378886.23	60097625.43	50806270.5	41605740.27	25940384.33	46884977.4
5162679.17	16810699.58	10627133.94	37924197.43	16223286.63	18915865.58	15538372.49	12649485.45	11792139.37	6966207.331	12619986.22	290721161	227627479.3
9894583.214	6837961.791	6729555.335	6282947.063	6689294.432	5293950.429	7395524.772	9717910.555	8881481.41	10437646.51	7649448.712	38629691.53	54764276.43
52324578.36	49014073.48	42295304.64	52928790.51	40656862.85	47822923.76	91159008.36	59723391.97	62999019.94	72557776.8	67089916.51	3515723.652	873772.1627
21911194.64	6480222.949	14936456.84	14313180.6	13366216.06	8328127.088	12881053.14	8103389.028	10632712.56	13993473.78	10275058.84	7272161.729	5868066.343
2670201.629	1562982.097	1394587.593	1539036.623	1311604.081	908147.8328	3515324.038	1071405.581	1322489.553	806713.7731	570254.3374	13672810.41	10354538.28
32091602.49	30975627.1	27420707.92	32300824.76	26906319.32	16528750.54	26224840.97	27240890.72	36331421.04	21942754.76	15627160.48	4100696.981	786437.5091
25656313.52	24278463.56	22971160.08	19007906.16	22282154.8	19959909.43	21367137.16	23151508.42	25840318.04	22930741.27	23270974.02	2829131.731	5058591.762
3775677.817	2681907.265	2558873.85	3191191.057	2064843.61	8716473.934	4408683.43	3349358.918	3207215.469	3148399.282	2522446.151	6309756.368	18616977.13
10125154.34	9977802.268	10610773.52	12549540.16	10736686.98	11163987.3	10040665.12	8362605	8692745.933	9355582.687	6788454.694	15562905.37	37604415.46
38169874.25	103477404.2	82263477.6	35318508.51	32794259.91	70091440.25	73914238.51	102082052.6	57986497.55	19576653.97	26079338.44	165046214	369614264.3
18509334.46	10480922.6	18018204.55	21624148.08	14574456.03	16987422.03	12175483.18	16290556.83	12915256.9	14681520.89	9659359.336	46736502.02	55534372.16
1037666.845	1020401.018	1219053.987	1215372.035	3070638.993	1704707.639	954226.5078	3182970.426	1277092.293	946863.4837	756638.4664	1707036.305	3008728.116
2046466.035	1273279.188	1640212.514	1063750.187	1421202.068	1252831.554	1490781.054	1755264.373	2081379.827	2327845.613	1622680.661	1272226.867	3136081.497
107987766	59104611.53	42384021.25	55773956.19	47527358.43	45072682.49	143165079	70952867.38	84099405.26	80835197.96	59554514.25	145316360.9	210573594.7
41598.46835	31313.52581	33999.47291	34944.65587	36071.75906	37065.14424	38747.77742	35974.68409	35260.67273	34000.113	33001.69156	45957987.3	60916125.24
12268728.89	35061400.48	48941020.54	35022847.05	22309609.15	10572789.35	11824531.58	11779633.92	13544161.81	13686390.32	15263405.26	8173062.241	11023333.89
1507438.728	996607.3259	1067433.273	795856.5683	1380118.811	1356332.309	1150959.317	2128858.257	1670450.332	2773434.367	3494463.334	448006.9751	479225.2526
40426183.74	12994237.38	11746785.37	15468490.33	18135631.66	20348133.87	5157242.177	16473571.3	11923667.55	17585425.32	19679137.34	97820275.67	74826885.25
8324287.948	9369176.753	11731119.21	12766304.74	18241988.74	4121860.739	16481554.96	13986720.9	7995503.165	9924686.352	13208089.27	18187850.38	18270707.12
3490778.08	1607164.63	1868649.374	1607408.641	2345598.131	1362236.931	2779881.859	2874406.901	2862141.391	2093852.318	1372193.862	2947427.51	1619933.911
406048284.8	376380815.8	348578034.5	454669320.4	387447296.4	383172352	670564984	505181043.8	461668188.6	513601649.5	501996659.8	79617566.24	74588760.1
2023985.852	1168518.202	1539060.014	1939764.956	936357.9764	334822.9158	889571.2138	2390958.399	1265505.884	873920.5676	1199402.751	6768148.732	7302782.133
770712.3776	967967.1923	781254.7655	822967.4828	717792.7139	707119.1735	679680.0401	1361337.396	798207.3721	793091.514	644152.3265	85314364.01	63562592.72
17222778.04	4229733.007	13536700.84	3585744.871	3914454.184	2741475.935	3838167.348	19683454.55	19782883.93	13569716.36	2885965.705	6905825.85	15065540.15
188051.4762	153652.706	155802.577	176854.991	168872.475	157686.5625	156250.0604	178474.3434	159366.1725	159982.6018	158848.5661	534674.8832	737790.2719
3457264.825	3681593.784	3938797.402	4407987.577	3745795.39	4254486.851	3867855.569	3214321.403	2558873.299	3301265.049	3084916.873	9357476.368	7863707.513
14359534.34	16899124.98	17655435.62	25081632.42	17483683.47	19864953.06	11737870.01	14603056.72	14557255.47	8529328.2	12147209.03	43961001.54	23458159.76
1086908.795	1721292.034	1937789.401	3239852.063	1705833.222	399982.5776	2091491.142	1086639.75	1643722.986	1703221.13	655084.6911	13508977.91	10517076.76
66734781.74	41069307.13	42149459.43	50598164.93	37844879.83	22788829.78	33686982.6	53064334.36	46359565.68	50375278.02	38998148.03	41078860.91	60074223.78
9242177.049	7106386.282	6571456.839	8763868.119	6818665.133	5073836.089	21938831.68	15248034.91	15684142.27	12468017.69	17284687.26	45845859.06	92327959.48

all_YM1_5	all_YM2_1	all_YM2_2	all_YM2_3	all_YM2_4	all_YM2_5	all_YM3_1	all_YM3_2	all_YM3_3	all_YM3_4	all_YM3_5	all_YP1_1	all_YP1_2
1413076.313	1633537.891	1207769.873	1034896.594	1092615.071	1286006.181	1377572.815	1735221.847	1772006.533	1665943.528	1622505.949	122553.254	152557.5311
7902655.941	15577544.73	11590418.27	10041093.83	9733650.915	7456726.678	11305284.09	9729935.168	14976698.52	5709381.629	4798344.455	6560347.431	14267186.95
1860856.078	1178386.45	1582372.652	1095063.123	1262564.954	1291624.891	1603999.6	1822922.408	1547457.638	1117902.426	760841.9213	3421244.404	3774556.444
3070225.446	9425887.214	22910253.47	27426841.41	22940739.68	2540804.118	3492393.43	9155955.943	3573674.972	6696014.095	6659778.678	18297052.19	15935466.78
3645677.444	4871135.754	4396407.769	4964953.609	3960953.714	2647833.559	4294970.041	3744501.891	5149098.272	2711887.905	1935746.401	1878221.123	3160147.217
2008954.973	1605659.725	1489822.619	2295915.297	1291201.613	1774383.882	1539701.591	1609787.756	1505568.552	1882888.246	1463987.232	25054036.13	7314354.896
1305129.186	1599505.643	1263982.405	1015663.232	1675008.724	1295659.033	1504357.906	1829676.881	1958360.692	1546381.115	1639814.165	5353271.176	13622975.64
3467130.248	1597640.54	1804457.621	1670278.416	2103768.931	1236835.04	2698479.55	2776072.025	2589156.585	1976104.575	1372193.862	7138725.224	7076099.547
1001797.658	465983.8225	330213.6034	501247.1574	557983.0559	328634.1978	183466.3117	950645.135	968960.5958	1353127.387	1565285.102	3998596.518	1913051.779
3789189.962	3238785.86	2608174.063	3174154.651	2634161.734	3127861.04	11950527.45	7158385.766	8472097.33	7271333.448	8656002.544	12618513.21	10394407.03
1468279.184	950767.9639	875386.2847	1185030.284	1033119.388	1121066.916	1030526.714	973980.7041	772419.6394	922559.9795	1236632.673	57737252.49	59692842.63
285255.5111	228126.6362	429955.6896	519391.1173	1241043.442	253257.8852	248477.3066	324160.3911	219315.1589	223139.1072	217547.2916	6931527.092	3103662.102
10348359.51	10750437.33	12325364.9	11840671.63	13481910.48	14155974.68	10687620.23	16013109.91	14644680.64	16899635.34	27919466.55	35214509.89	66813178.8
2606904.182	2901285.037	2239695.318	1138834.964	2249936.289	3414110.867	4931122.619	7551172.598	4866960.039	3386856.131	2162746.48	7944485.632	10233557.44
481991.8932	396704.8642	404083.1326	445115.7565	450494.3177	444795.492	417439.19	432865.1056	427730.7561	404872.1106	405187.7841	191989.5507	194337.3435
847007.7664	1411876.113	1313883.525	1507378.354	3089293.989	1138694.61	3454116.42	1606178.606	2305561.511	1868779.065	1373271.099	692864.8911	1553923.111
52898926.98	56792517.49	52917280.12	52730054.78	40681402.15	47659261.31	93691053.05	66622387.91	77631640.13	77217291.35	47731975.24	29838534.03	41891894.17
27520742.17	30058478.09	43763581.98	37747386.96	24574525.51	24495309.13	73473000.48	45014727.61	58809030.34	10572134.24	40642958.46	125595831.8	49667768.81
1332299.462	1015520.827	1176942.598	1626919.463	1276610.566	604698.8251	1894821.503	1336648.668	1270574.269	1497222.434	1624073.339	121254264.4	277995807.1
163329.6448	126198.4741	129161.1663	145319.6689	138760.5292	139623.6227	134976.1212	146650.2717	132116.9183	130289.8148	123367.0292	146704442.9	334640970
1070594.542	947583.5873	5079157.597	4534093.264	1524868.287	2237676.145	11219283.7	8675380.12	9952428.826	9490404.158	6796866.385	3480427.317	3480775.182
493561.0476	379827.6386	377082.2767	421469.18	413457.2265	414176.696	413678.0222	407071.8629	413016.6134	370573.2827	370468.3127	2383018.908	1730730.572
128041.682	101549.3274	105742.8681	113493.9176	115515.6381	113675.9637	334271.7025	161660.6584	163313.9543	244185.053	238036.5453	1922887.581	3495465.552
5306288.931	5648698.042	7904562.054	8118455.684	7800019.264	7967392.762	21160615.24	12521499.31	12541598.92	10718214.61	11660119.65	10713407.17	47401387.45
1540767.402	3833151.921	2901069.225	1480545.559	1158782.041	5810933.791	622114.7909	1043560.819	3160831.788	2164744.118	9218218.052	8618494.428	8682226.598

all_YP1_3	all_YP1_4	all_YP1_5	all_YP2_1	all_YP2_2	all_YP2_3	all_YP2_4	all_YP2_5	all_YP3_1	all_YP3_2	all_YP3_3	all_YP3_4	all_YP3_5
845262531.2	921034050.8	986705277.9	991014686	1117704193	1131614477	858626796.5	747887728.4	992002761	657177872.2	750231539.9	737602471.4	623689438.5
43150212100	42026642631	41128478792	42151591139	35434215113	42626029480	52001908904	45922205850	33214102549	34392201743	38924855347	45220060855	36973354072
10554139008	9350388529	10442598289	9787161863	9064674719	11672794337	12758934088	10678002869	8328559682	8408652528	9026281524	12392972000	9207874177
13854109166	15260664952	15813007886	13032036948	16741954316	17047487778	8918899143	9522289432	8882028787	10481367847	8252301220	8427589648	8717712413
10190544046	10571982869	10220832863	14489040242	16399958218	15131119763	15392082877	15273112157	18305774442	10839129453	16218171082	16470588556	16187281405
8893054638	9711862660	8810049881	8263954341	8720581789	10539154860	17015134387	11383069478	7722832075	8898858112	7620199480	7886564437	6300170647
13219723.98	12300405.45	11225361.4	633085542.3	165927723.9	14217218.41	23760909.21	10674757.03	209008198.4	272482425	328421551.9	848575142.6	284584207.2
7737662129	6580560774	7231110814	7261423389	6303337207	7420977464	10648458247	6968273192	5857114404	5879540543	5794762343	9474360982	7127328951
1579800490	1628622273	1658402551	2052125888	1854858173	791997609.2	739509762	1379165965	397003984.4	1602538443	294529159.6	310957377.4	427950436.1
3440124408	3326874271	2304438079	165621814.8	179390680.9	186148096.4	150926900.9	277690155.5	175049657.6	213607414.2	179888453	161588850.9	177152207.3
108414059.7	162105638.5	86627229.93	219873716.8	159836270.4	137891683.4	86031068.24	155287976.4	285315131.4	1428814602	199918176.3	237835949.5	376585310.6
1013966565	815494725.3	1330002287	474525274.3	284928370.3	223332617.5	437368865	305605061.1	175794986.5	293763484.2	200183141.9	174655442.2	201712829.3
1223939544	1132641642	1106447507	327920645.4	254887365.4	117748313.6	317056396.8	213108841.3	66459490.81	120747361.2	96804403.88	86190446.46	79065469.21
24306114.7	19454829.92	11664483.29	7267346.787	35380430.13	8972343.897	11633120.5	7569789.937	40779120.36	77486653.96	26779468.61	24303362.48	54963182.94
1119168750	1464115509	593559957.2	1304126991	1008643640	602282233.3	569289570.1	811607324.3	1162304119	3973648523	645661138.1	1511082508	1272467472
30846702.39	34869771.83	84070168.12	357294509.7	323515964.2	195484488.9	83215148.28	290062793.4	545273467.2	407907763.8	166690445.2	575840156.8	673300234.7
51337605.69	58148462.74	22625834.04	86210565.58	83202770.12	71812976.84	59483569.12	78123287.95	146310737.9	49590574.62	110054192.3	127951805.9	113521455.5
21668102111	20941640911	22672970668	13105776273	12417978336	9641179559	12217629613	10064967023	10904593779	10838106387	10273087615	8526324110	9079480556
3528512712	3277689257	3288519785	2128331379	1708484017	1365371028	1606381671	1583057924	1612484205	2695725187	1601985559	1260661474	1574385174
10739648.89	14741758.9	8500376.346	54150013.33	35679518.09	26130688.17	11537649.56	72728233.2	77286414.02	44493602.8	26687315.2	125202070.9	99573377.32
904829620	998840719.1	929728493.1	1389556478	2281545811	2284013360	2171966635	2010001599	2601220063	1581086601	2148230305	2137826222	2436789068
329591612.7	322865580.5	406220631.7	556805530.6	663266829	1340036865	656765666.1	1281221566	1069478727	439238056.3	829732575.1	1068426118	868633684.6
334506603.7	329839658.8	518449351.9	147962914.5	94544154.82	50747219.68	104348993.3	74332628.34	37392322.62	68689448.25	38920847.17	33163879.82	40780901.32
103003388.7	144883800.9	108567435.9	129556900.9	110491941.5	125448278.8	150616578.9	81312083.89	131623677.4	200532938.8	151334115.8	138975098.4	180120379
310020651.5	338363605.3	636694642.6	122191489.9	91788216.01	31132908.61	76082180.29	53133765.12	16628656.64	20795925.95	11295582.76	12689795.14	14739366.83
1035862755	1090056656	1074986762	956048345.2	1162607383	1225930897	839796025.7	1077130870	844170527.7	813583291.3	935812259.7	977409708.4	1001099918
4981800.164	6367260.458	4203419.951	23843812.53	26052383.75	27563945.33	24010639.82	29408767.09	41958650.32	29828992.28	35828704.13	34287449.59	39900020.08
392099084.1	367213288.8	386589512.4	407419404.5	353399281.8	430815288.9	576788003.7	409080280.2	307423883.5	324906569.6	389300521	422926013.3	390010154.7
7151856467	5863554217	6817465362	5497895632	5531693218	683458967.9	6941909509	6428497402	4580755781	852199561.3	7277293357	6017396378	6253136170
123263821	157528089.3	146087051.7	490864052.7	422926040.2	327512045.2	322093106.3	252364019	319700910	514391977.2	390386311.3	305937790.3	440296710.3
357873069.8	423408937.3	255242655.9	254682681	120501514.7	99563183.65	151951292.6	107854402.3	99610523.38	169358196	164359354.2	61152241.66	98326672.45
6707943.002	5032920.368	4333192.434	10780279.79	5877350.243	5905620.884	4492839.102	9954502.033	31481644.44	30401441.48	20071804.86	18521097.43	39458553.47
103906696	148222534.1	64812932.64	111966758.3	121825209.6	102519316.9	83327190.25	118208067.4	104930201	79440677.26	131136336.6	158297544.4	112415998.2
285839753.4	343246169.6	174548822.4	285413079.9	295828427.9	275181869.7	236281575.7	251597619.2	204877396	78403525.43	155513234.4	195514700.4	154593716.4
781336243.1	833219212.8	914956310.3	1081750967	1214141783	824041175.4	910539115.8	945351172.8	703152586.7	1381242932	656629282.1	670613326.7	716570969.6
6393995778	6484318993	5919859511	4496764978	5178234822	6062351889	4728465814	5467393221	5595053863	6167814731	5629844049	5068553194	5775686381
26343286.2	39625408.81	18339923.31	54352854.09	39586436.71	25267494.54	19049932.21	26783774.55	55141840.31	368845926	40680931.55	42166377.13	73460221.35
227823773.3	230187938.4	276413152.5	165774503	137800774.8	116328834.2	142373861	129184412.7	154522476.4	194248946.8	148281786.3	122937207.1	133610300.8
440782091.8	219144001.4	607730498.4	460558273.2	260458854.5	328435289.9	353594635.4	432959474.1	214789824.2	153216538.6	571908661.6	687103021.6	860623773.7
295109601.3	226608740.8	251209553.3	288396479.9	271347996.5	218938565.2	257679282.3	310659331.2	276750862	202517916.1	279631844.4	281723971.5	242576942.6
180793806.3	160258259.8	138471070.8	7130623.784	8247162.653	8041228.094	6393988.744	16567556.73	6216422.913	9256954.998	6335776.118	6062564.114	6015956.382
2630604645	1597428206	2799456082	1796790563	1939963332	1531625000	2101058062	3272674894	2228926369	2819597861	3016840882	2722248677	2855343454
78005096.66	58488742.66	24492219.18	16523533.71	14950598.32	36846403.24	29729907.1	31395680.87	72357769.93	22773780.19	31622438.82	37555688.37	67854194.31
57512142.74	58349982.51	24921646.2	56888136.7	32103800.83	39120082.71	33534977.45	60981146.09	62793991.78	100273209.3	31369249.09	97343299.99	53459083.14
195100599.4	209887923.5	233581564.8	11888996.07	16532626.56	4321997.652	12924623.19	18216569.37	3231609.397	5742260.121	2928720.736	3374828.306	2933606.886
144894326.4	198666410	136583267.3	90612677.1	58252213.82	54083529.74	76418210.94	50557560.56	39704936.37	56235046.37	63394790.78	24996051.83	37410724.77
85408249.24	137621832	55324234.65	77430906.05	85434045.42	73847892.9	63096789.04	113983128.3	75687415.9	81244531.22	49234623.81	98116310.05	81113998.88
6167899.069	1299318.722	1156971.029	1074318.122	1102958.077	1753341.203	1100119.505	1080834.712	4854279.118	1344726.564	29431066.99	5079298.942	1024964.345
259190157	211118671.8	220555628.3	271559866	169274772.4	310056599.9	398310790.9	309648461	227740475.6	173053208.2	305575433.7	205381183.9	216872193.7
1232200.567	1221755.388	1251558.556	1114141.683	1096166.101	1117359.612	1142290.943	1271763.137	1215043.2	1153642.918	1095844.342	5928590.645	1092011.328
80653945.45	86487298.11	87841990.98	238121015	261298747.7	93340540.54	106223393.6	90584689.03	90999510.73	95587399.7	53633349.81	103969704.8	108979131.4

all_YP1_3	all_YP1_4	all_YP1_5	all_YP2_1	all_YP2_2	all_YP2_3	all_YP2_4	all_YP2_5	all_YP3_1	all_YP3_2	all_YP3_3	all_YP3_4	all_YP3_5
1908067.221	1556334.608	4872357.415	4942836.014	9868124.661	7498873.697	5118142.352	7916253.26	3385722.716	1692529.613	3109765.206	2395828.696	3224007.494
208901897.4	259794428.2	103553616.9	170706015.6	87915707.46	16552764.48	82957018.51	37987059.96	25817436.62	18908612.75	17302560	46772310.1	24336974.34
19996711.26	19778386.49	21971472.74	18523210.9	23365093.53	15091919.67	18790953.36	13609570.5	14408081.58	16418094.76	13487298.52	12601372.34	14304112.73
26476894.05	32981384.67	50403983.81	26878270.57	17079919.26	24082635.76	48325810.18	12976717.19	22832415.51	27307951.74	13828865.14	24912263.76	66164920.05
209291386.7	251267937.1	78518582.84	162477955.5	96056516.78	92796561.27	85423247.23	146866732	160859368.8	404895120.9	99170363.96	233333383.4	147770041.7
9248730.142	9978015.988	5136795.051	14510548.57	9035478.88	10823342.48	10608716.98	12245874.3	27776401.22	19146034.72	18906989.66	32878277.32	30905483.29
57083746.67	72305648.16	46094073.04	90841323.41	85717386.92	96567009.35	70714735.22	96365359.57	103873774.6	104856806.9	97782634.78	89446641.71	98410554.3
111128649.4	96567381.06	116311504.9	53948452.59	31210189.15	20841134.32	41789095.66	31002938.62	23758144.3	24132988.65	22873604.31	22761117.91	22630549.49
115571016.4	136149830.6	226804537.7	122836428.3	149583934.7	360376471	173180065.5	277604588.1	196444950.3	228146629.5	212213548.9	222573570	254812441.7
103273155.6	135360335	58371947.89	356540625.5	457870180.1	496426639.1	403350058.3	468746217.2	644550066.2	218231633.5	468758251.8	539376220.4	509691735.3
715032.1174	1286448.489	1689978.01	595991.0275	1428857.054	1746347.493	879820.2929	2291394.243	1369914.276	1274201.714	1275724.12	551300.8179	1653896.899
136235327.1	151097591.7	153827194.4	132848150.7	144152953.3	69651952.67	132128226.7	67695986.83	83833439.94	127495814.2	96766267.38	83305388.21	95092042.36
15039029.94	18518665.61	9227411.701	21285799.92	19962150.9	17613047.93	13340486.78	19040443.05	17737485.25	21593945.62	9766790.154	20353630.42	18836520.23
892257257.1	1126064774	864843226.8	483788168	397600208.7	475649076.3	475111383.5	551825823.8	375577726.6	474712692.3	522513359.3	262465315.9	370481610.8
1666996.984	2300705.493	2714399.791	2655452.036	2688653.114	2640647.237	2118815.842	2916480.299	2527532.041	3356452.111	3020477.286	2067678.072	3044151.396
1036644.083	921632.8696	918894.8163	846999.2821	927828.3256	847932.2047	735490.6313	842521.1852	830446.5673	958102.7531	879226.7524	815075.1576	875306.1054
6164320.639	5075974.875	6405560.679	101312475.8	58472595.66	16784384.11	11138877.83	29378506.83	8738625.341	37440304.09	7602187.99	9305601.419	6251496.832
11198370.85	15408686.55	12700202.97	5077391.223	9094584.702	9083151.506	5938342.697	8150217.979	8845966.78	7360163.765	9036399.554	7060159.393	15580307.95
1770318.201	1573908.992	1569233.11	10489508.36	11584404.45	3568860.406	599524.2446	6148464.656	5776031.026	8565317.45	4921331.582	6587492.13	8210191.798
48272890.34	62499622.77	19136837.09	45361957.64	32318981.71	23243613.4	22808021.27	37803565.21	43412770.73	158459885.8	26445931.93	62154046.18	46325750.91
28981511.86	41177627.68	5576568.52	20881922.35	8777664.204	13988287.21	10450362.03	49730270.97	27071774.41	8524688.876	10104785.84	73205399.83	23044347.12
48475710.56	21505957.39	16116786.5	11829371.65	12119802.61	13105325.07	14626600.45	13439933.88	9602642.321	8099869.168	13939061.5	27042514.24	10912549.86
165059218.2	212463124.5	162914937	155190671.4	127084064.3	63514819.35	67170181.5	92380059.48	38221439.91	233792466.5	31014004.08	38645750.4	40150393.08
850275061.6	760756175.8	646636750.4	441572860.1	296412770.9	42480350.69	146010305.8	131986796.1	35353417.11	28067545.88	25937002.93	69535693.81	23565186.15
671135968.2	360631794.2	730717878.2	513734865	477898405	508883906.2	672098777.2	849793801.1	543695030.8	641169151.3	798788250.2	660927960.9	739021467.4
22584548.35	20771542.46	19304347.16	19285801.33	17172465.16	23373954.69	29033924.32	27505034.37	16674759.23	14028386.78	25947783.86	16841695.4	18135335.27
44705566.63	61715427.06	36077580.66	81631706.72	69129242.46	50417030.07	44398385.31	53139414.19	71265301.56	492745449.9	52365529.69	65382452.15	86873997.4
77885865.75	76093757.04	54268734.62	54796930.61	53550166.08	50403249.6	52867002.53	66850518.06	58005293.91	52648322.83	46046475.48	73362967.05	50808886.65
26688098.77	19021718.41	9971126.588	19437493.44	17807790.22	18779148.61	26170707.79	22991616.73	17716058	16361117.14	19937872.41	18571416.93	20629908.72
306643806.9	320310727.8	407548336.1	207452965.5	262420344.1	128023839.2	260069310.4	121884134.9	108616126.7	125002172.7	94942046.22	97891921.91	113282994.1
36919126.82	37614753.92	54344521.59	23155866.19	21570834.88	12691340.08	9909520.289	15062863.87	7083853.48	4341720.796	5857383.372	5669582.252	6531473.77
32035150.35	27976128.19	46516907.38	23741002.8	44461715.71	61872302.09	29001605.06	22274286.23	37980081.98	43712831.45	51293916.61	15348129.02	24272969.38
2158041.126	3427314.367	3898016.052	2948182.749	3999159.348	3413181.729	1747576.898	2266057.976	6144988.435	20530927.55	8080571.747	7647916.944	8745998.218
141257350.1	85801617.88	173884784	107655523.6	97786481.86	85538269.38	92294070.04	176658841.3	122872760.7	158899881.5	188174933.1	156536473.3	177598173.8
144193787.9	149804308.4	158785355.5	25686033.02	26004787.4	37203764	33026387.71	37701462.15	13815418.11	107019173.3	14535956.14	13884334.16	17421727.12
144962031.3	144065358.8	170730063.4	395922983.4	372457103.3	461676050.8	341839197.3	455449210.6	712119535.4	310581394.8	544238298.3	630109469.8	470931690.5
67681420832	65785396428	65355078462	60853024917	54126130110	64091057425	76144597276	71182588622	53270084194	24476182136	60142705326	66664811841	56178895503
1692273.356	4092224.762	6490315.091	710964.7297	1185163.335	310211.3475	534357.2555	535700.9425	1133352.081	1169144.252	820591.2196	474532.5857	1639853.722
5963807.626	7564867.856	5056902.101	12415882.4	8338094.263	6243993.654	5897542.742	7277845.888	12157942.35	129157778.2	8522547.96	10498574.96	13065136.49
3614473.054	4738646.204	5731840.003	119249349.8	55525737.45	21005062.61	13913826.22	42154219.93	7354311.62	15019076.13	9134006.309	11781283.75	12461198.06
59367496.34	65295980.5	68970482.38	45769010.61	54965130.68	60486907.04	46286508.45	54835924.38	44338731.2	54128835.78	61510363.4	51470753.26	59485518.75
24415173.03	25838752.96	22586433.95	22549699.07	10716894.49	11131032.72	14209106.87	10038580.94	7287070.669	10288257.12	11279882.38	5111420.173	7999573.516
441704465.4	173207609.9	556029846.8	702548167.9	328286074.5	238782894.6	357139008.2	673512932.9	394405084.4	466272065.1	658908611.4	528172924.7	567840819.4
39127520.17	45914757.93	26470817.86	25738426.92	13979332.29	14242118.68	18203712.96	15883119.39	11292325.48	18678386.67	20690169.75	8896242.11	11503622.9
55345729.5	54609919.91	59899064.95	80821930.43	97406119.38	93036818.45	91781990.78	85995459.28	113068183.7	111367593.9	120442287.5	115950093.1	122901446.5
4458314.59	5482685.48	4736343.247	3627714.942	4343633.553	2115793.622	2803214.422	3169326.316	3030323.818	2050773.204	3310733.01	3630553.748	3942074.949
46101429.58	49460165.29	38178401.42	31988002.54	32126033	32562551.02	21508471.05	29082913.15	39691782.68	92183877.18	51288627.78	37437487.58	48774587.82
10645168014	11134361696	10122713555	16991530016	19779983138	18301527940	19712733618	18096943959	20505992228	8674542273	16130057993	18591873054	17296434965
137841654.6	136098112.4	127361658.7	156212022.8	157750739.4	150725861.9	150717009.7	151252416.9	173494129.2	67219537.2	137794179.3	145433128.8	45780892.4
517197098.8	961001455.4	665722410.4	591876593	496653440.3	911670296.1	862229771.9	617383590.8	1672112878	888014123.3	607633581.8	644317775	588516382.9
22541359.31	10739969.44	16367261.64	4701363.202	4389055.818	6453389.905	4793106.073	11802448.07	4018306.945	14278317.03	4345781.791	4477569.917	4211634.443
57766425.95	62927263.52	72351536.83	55244633.91	62598930.54	54768078.98	57942424.75	47605352.93	38951983.34	31719720.63	35275146.13	35482399.47	37503602.75
6964533.758	6548781.869	6460529.352	22179827.57	13636746.97	6113365.188	6791867.967	18713169.81	31553729.45	18204556.35	7204320.843	47130110.64	41989957.35

all_YP1_3	all_YP1_4	all_YP1_5	all_YP2_1	all_YP2_2	all_YP2_3	all_YP2_4	all_YP2_5	all_YP3_1	all_YP3_2	all_YP3_3	all_YP3_4	all_YP3_5
615014.3377	1119624.162	638704.834	12591990.22	18289342.39	33769361.43	15952808.54	31410892.86	19794485.88	9779564.048	18785489.13	16930235.51	16908189.4
21841108.41	19024911.29	2922456.757	24048977.35	7123976.446	28167549.15	10363401.43	44306326	82154760.98	61582506.34	23846780.13	148898385.4	93502817.85
4077944.042	4723766.646	4089953.721	4504849.683	4918241.759	3335780.215	4382331.782	3676997.141	4164899.022	5491213.039	3209457.129	3499795.629	3757312.768
13932204.8	15849619.59	5268459.384	13055393.72	9594229.113	7547987.135	7177020.615	12410289.19	15017169.02	31896176.42	7020553.841	23099600.4	15663791.67
1301488.424	8253924.638	678726.4225	1208389.223	953809.0448	5635352.66	5218673.137	6695632.886	7463762.54	15652598.17	5660155.052	587450.0974	11018922.92
36708318.4	35291168.61	40137590.6	6929415.088	5229612.023	2330857.394	6055481.798	3806662.325	1952165.994	2969477.289	1743503.451	2221416.287	1813711.364
1182671.256	1223370.361	1238979.28	1420518.435	1080073.452	1759965.319	1514142.374	1198250.663	1016948.18	1225166.313	1641601.772	1591605.951	1384967.179
251853929.8	265259913.7	267059494.2	353887930.5	608963821.6	575016758.9	549017098	501907767.8	651981034.1	422656717.8	517491935.3	517126184.5	600319119.8
46421991.39	58908643.66	14898524.79	65786434.8	69404884.05	91875061.26	70211523.6	77413406.6	50454444.33	11428452.24	34229627.97	42365329.17	29982167.82
81751486.36	73817252.91	85973997.99	60190139.36	67089683.88	69377552.05	67906328.03	65422277.81	64698947.23	71424170.99	60627751.83	49782876.86	63888173.67
361843604	356909384.2	225200633.6	26313186.09	34824090.89	32842684.5	23604864.64	54553477.82	17752260.89	33418623.15	18726226.62	15535753.43	19597330.08
60974967.85	61583268.59	56735791.56	36685940.02	37217505.81	41449421.38	31591856.73	39530213.22	3769020.593	4230543.812	3620141.783	3812054.743	3525712.754
36525842.26	30032433.67	35485337.1	34772707.88	19402579.8	35756310.81	42940334.39	38304205.35	35568657.37	25495385.49	40183437.02	27575346.42	19056464.94
30131298.92	35143409.76	21528999.39	14111446.73	17666660.15	18053702.19	16476451.14	17612436.79	6200331.881	4631282.007	5294253.695	5897577.845	5834368.104
764477499.6	800900278.6	653363732	925088388.7	1077557587	821614069.5	1019067254	1018030207	979417352.7	166798873.4	721033319	855888412.1	463938304.3
8928449.181	19800893.71	241235848.8	1547563428	3528257244	1780112740	128134449.2	1905992910	1187829916	1269208025	640999461.2	1005017770	1246859168
37454327.34	41077819.14	60921715.89	83367661.8	91929898.83	59390166.55	69573773.36	53429690.76	10139958.32	11145298.48	14600042.57	14824014.93	17848292.21
163897678.2	124884347.4	205355293.7	176257257.1	144606019.3	85379467.7	117355578.7	160083616.3	121323778.9	149879569.3	134952523.5	170307685	122550433.6
897946.2372	761542.6384	1101012.366	916113.6676	907826.2836	952169.7597	731942.8953	986458.0299	5255848.635	1000651.344	913833.5512	109769293	53487989.96
22012993.04	33671553.22	10919026.85	31766661.11	15823349.02	27156407.93	16462834.48	24072732.75	21519243.83	13977523.38	21660803.21	24343865.88	29917749.69
26610971.23	42256123.82	18856044.68	6792959.483	7128238.924	11049526.17	14818949.85	12455978.02	6197456.846	22176261.34	21295326.15	4031899.075	8896636.875
5164167.898	6612329.803	5091111.678	10026197.43	8530552.596	7632814.087	7119252.793	8312065.621	8248653.758	10326009.82	4955101.409	9444512.834	8644583.633
498360.3737	635899.9085	523895.9792	931392.7035	506668.8843	844555.7838	463527.2131	633963.3222	4166348.57	3166362.706	437194.8131	8093436.26	6567042.704
756702989.7	443618045.6	714760784.7	600302578.4	509752731.6	479843926.2	559129073.2	750373576.1	622635698.1	769719644	788379845.3	648213606.7	656923799.5
855189.3564	928424.2717	861064.5491	3279449.075	2763656.913	3134501.102	2443759.566	2875643.575	6466196.847	14264459.64	7284768.317	6859049.001	8418882.737
4149924015	4380691593	3117309878	2898956107	3171637502	2077604152	2794705382	2519155623	2608669112	2813693504	2266230227	2166428674	2314027923
1240366.909	1271004.908	1020769.703	1002927.825	1096814.88	1247613.278	1029344.045	1181892.482	1330581.603	1241994.666	1013572.717	1202326.423	1068075.081
10110179.65	9488726.904	10865126.58	9833992.921	15398489.68	8543089.857	9519877.395	7569744.233	9169344.361	7339800.023	9404210.27	6102387.761	18594333.08
58425834.51	30821708.49	70805465.35	47344581.58	41550478.15	35454519.19	40610575.36	80529547.01	47581096.56	50170079.35	85831196.32	70479979.6	80338821.48
6645141.421	5223910.71	5789064.787	7204829.434	6648819.995	4729741.285	6515292.238	5822269.514	5048431.043	8138313.894	5483907.133	5111315.657	4633776.296
1846113041	1770268754	1940962915	613996211.5	467701726.4	122008011.4	333814704	326415953.4	119360519.4	88836531.68	95898830.32	205938687	98416518.07
470781935.2	440376382	457242864.7	414326047.8	346606101.7	399342494.5	386516368.2	428781712.5	383157200.2	283996668.2	353617080.4	363052703.3	335757844.9
39413452.08	41767988.7	63540801.8	39798712.68	41938645.36	51486277.09	43470585.99	43634719.43	44738162.55	47772932.02	40886553.5	37967677.36	41485851.83
15821004.74	15169539.18	14677504.02	13172300.56	15208578.59	10152042.79	39214613.42	17315614.07	11760309.55	9107910.275	13666926.83	9688496.048	10211373.36
15152953.28	16039350.82	8376832.451	16151723.8	21453349.71	25125028.41	18366520.02	23828422.05	14549712.47	6849094.2	13954401.27	14151340.41	11788954.32
3363330.06	3443806.443	2985952.264	80979230.84	91221671.6	99036536.97	98617072.18	86743582.64	37302141.46	3683348.877	2929030.605	35801815.43	2810343.289
12866011.47	13977323.05	2657280.06	8187942.115	4266361.657	9136926.29	3359417.009	23274457	18316728.27	16489719.57	10544220.66	800966.5204	20066360.42
3159878.204	3089238.783	3172676.876	10207645.58	9674411.941	7456450.15	9225741.551	5710615.299	7488748.028	3464354.737	8882563.98	12964910.7	7894570.39
3053938.764	2666512.393	2707051.095	10595316.29	9178342.948	11467712.24	7612418.612	10523673.91	32710299.58	49240337.44	32671777.23	47511014.93	57428601.79
100934440.6	70516679.27	84260347.62	61077841.43	47433073.31	15113369.82	20693016.68	22554138.56	21931602.16	23391107.85	18935659.26	25267873.84	16820972.23
3864244.38	3762541.71	4411087.014	10169919.71	2820989.594	5927331.896	2879398.535	17345586.06	5167755.585	5414836.438	8824620.44	4712073.494	9177790.836
2933057.976	3050545.193	678472.2964	1963733.26	2014108.068	2475744.844	1156520.719	1729662.314	1789768.744	1605694.473	1105198.07	1349572.291	1241698.628
2968773.676	3021156.874	2408165.543	992093.1727	1203796.183	1232735.719	1110290.827	1353673.044	9125569.288	3282184.261	3517142.675	7589487.922	1912894.82
163827021	113197236.7	121902064	153894911.5	140172436.7	106574008.2	144797964	154858545.2	147476163.9	174958651.5	128137166.3	116814459.8	118259183.3
875772715.2	792221927.5	1286262678	913689000.2	1017106866	1081751284	980604784.2	925068319.8	1515513490	1545606584	1374076603	1217970493	1408935059
9940594.144	11759656.46	19076151.05	7700724.654	4802860.233	6868333.939	5848578.975	7720036.857	11033156.81	11336488.27	10051443.02	9330635.274	9280904.477
33116365.13	25451375.87	27822315.77	22823076.55	44803729.48	23747080.02	23869827.9	21313840.74	27931410.64	20541364.44	27115982.58	17181972.92	42793061.36
79683359.72	21133533.88	64710309.47	72205457.04	81563583.97	160279382.9	116424047.5	159366862.1	89083985.89	77323711.02	93604374.94	101101183.6	75523889.27
3150106.246	3059957.207	3914495.149	2827240.825	3315674.116	2193187.674	2673879.285	1716875.462	2328768.286	2715231.722	2263441.533	2327037.273	2633872.814
40182641.18	40129568.41	2402079.965	23950556.65	6793946.543	9689796.903	8610280.386	36652700.78	17267309.35	8940260.061	5602932.923	48579411.48	20110897.89
34462160.98	36759852.15	35179889.35	17165626.98	12227753.21	13381083.77	15963419.31	14839084.67	10154829.55	14098785.72	9988302.107	8400184.484	10303253.39
20694914.35	29379196.66	17180555.91	81507286.31	61079918.55	32122560.97	28682631.68	28539119.38	9926990.32	6748294.889	8559605.356	5487001.611	9738761.671
37638025.43	35335023.07	33157474.23	26671074.06	27624685.57	33700177.51	24196111.34	31157869.11	28684142.55	57558529.73	26626103.22	22255042.1	30663294.25

all_YP1_3	all_YP1_4	all_YP1_5	all_YP2_1	all_YP2_2	all_YP2_3	all_YP2_4	all_YP2_5	all_YP3_1	all_YP3_2	all_YP3_3	all_YP3_4	all_YP3_5
20801028.08	23001455.79	41864382.31	26564927.4	22347659.86	20896668.53	23903177.41	15199285.36	20456399.48	33444183.67	18497537.01	13889256.11	17230140.48
8685820.917	10227973.61	11609924.94	37795979.2	29098122.36	11183311	19129774.73	10610421.82	3245480.664	3586144.927	1592001.416	2346785.134	3376938.617
98155857.32	218110801.1	138381863.1	124654992.3	132827007	172579658.6	222333740.8	123828978.7	155257894.3	221690276.3	140471415.6	166174543.8	157139878.4
101898638.5	106557410.4	120358109.7	65760909.89	83044730.78	112528715.3	73582708.89	105134390.9	122978443.5	119419396.8	123297751.8	99928969.86	117224687.2
1561436.177	1435794.021	1819246.026	1838877.146	1470097.65	1369996.29	1475725.222	1599295.966	1479279.958	1375494.601	864077.8805	1614722.397	1268131.902
1695759.52	1740507.612	1231884.238	1896916.285	1864468.8	1422142.258	702306.0951	1896809.873	1618273.475	752213.0965	876700.1531	936477.7333	1303048.35
261719397.6	222367736.1	244870319.6	290925319.1	217571059.4	195910214.9	282518344.4	292579628.3	217145946.4	335653183.1	346821111	285853617.9	244903957.4
2259668.51	5456525.92	2653471.28	11651665	12427510.19	12845075.94	876775.3844	28557192.43	9370292.187	13040130.62	1066077.313	6007331.425	4775717.416
39661960.28	38310806.16	47123164.28	44361331.61	53893488.01	51083382.43	46697170.49	51582439.13	45897816.2	32214070.87	35509494.72	29212444.99	32623896.69
3913846.764	4685024.164	2073027.364	4812050.492	3824431.997	4134745.935	2610372.887	4247758.079	7827877.964	14453745.6	2934406.829	10872645.58	7973930.069
85523714.71	109913335.1	145266803.3	152553860.1	90527808.48	122641695.4	85777183.91	40383617.39	125316566	108498068.1	75107370.05	95407826.85	81482984.93
14945597.64	26750014.3	13759369.56	7392316.862	5627228.259	6027811.65	8806838.503	6192541.921	4986324.816	4322401.454	11398887.38	5126291.387	6566635.894
1164189.211	987341.6543	1427464.879	1187743.323	1176998.712	1234490.123	1040409.801	1278944.939	1120069.023	1297346.601	1184787.147	117867830.2	32551526.47
32261958.03	42133201.06	2411398.663	15098376.04	8439776.041	11436145.67	9105218.882	59481241.08	50321698.97	4300975.284	17563187.03	173374065.3	118858827.2
353734652.6	394201800.2	337194944.5	247707582.4	283805196	321598927.5	246723794.3	248400865.2	214138920.4	141042812	176779477.8	174997007.5	184847722.3
27689209.01	40106981.77	6004012.55	28559580.27	15683313.55	15852322.93	9947200.933	43110454.75	28643791.1	78131123.11	13124960.28	67566377.06	32690435.09
4923650.526	5167696.599	3860509.653	2221892.692	2827894.582	3217451.249	2867847.108	2805715.266	4817425.863	3456768.622	5356904.531	2578618.184	2104826.959
39111735.77	36971650.49	54491132.6	8469939.495	5979818.125	2861583.516	852159.2784	5436540.962	3130441.239	4162839.489	7318333.416	3037437.856	3157908.597
2727877461	2165902398	861508132.9	3435990913	3054118810	2148473712	2736328626	2951571147	2448346933	3771050433	760541362.4	2561654162	391435002.5
8544335.033	10037134.12	7662236.274	2645886.299	6194344.539	4708557.272	6452748.859	3469599.631	2200031.215	2217192.252	3229764.094	2491522.214	3788692.137
33855070.27	29110408.96	35450485.52	40869697.28	38230679.56	26663311.42	29622133.08	35437654.39	68355200.78	50301364.21	40772182.37	37029435.25	33887438.37
13559600.12	11171480.04	18198981.94	6932715.457	4451921.637	5604045.55	5063720.036	6447795.404	9155735.728	14514124.36	8501436.896	9188028.104	8453424.179
5073290.186	803349.8863	3240340.527	2588448.059	2075544.767	1828705.926	1205083.971	9751676.771	2510546.257	3003512.412	1562979.221	2077571.497	21346006.67
122228189.7	125117349.8	116305365.5	194982192.6	202734115.9	187918341.9	174043506.5	190996594.9	184752037.2	25200103.22	144534597.7	187669926.2	98457577.76
151919110.9	210793794.8	116195472	490058603	351925910.1	207231542.2	120115723.7	203217291.4	94079554.49	73943152.07	65229572.38	71793346.53	91231644.47
16337155.19	16365488.24	15106784.79	13837367.9	14078734.93	18185105.99	14307580.68	17757433.08	15480861.61	13651404.03	14474878.72	16443270.95	14381980.7
3675722.875	5290846.793	2535430.807	2206761.76	1217931.663	2248580.079	1926771.487	4924589.153	7410860.224	6353541.147	3734895.219	14210048.61	9924919.749
183295571.6	98765454.15	187995402.6	190398602	118727428.1	130116861.7	162467452.6	160898402.8	123773524.8	171533698.2	162615975.8	149704698	150148920.2
383116.8659	420732.394	376932.3487	2792492.492	1251603.215	1126813.085	1460466.271	1938850.636	4735931.08	6319860.134	2312351.284	5285394.16	2798386.586
1604860.133	1511835.233	2461874.099	6551576.414	7061241.051	12974459.39	14429199.2	6341826.268	14468700.15	7323486.063	8882567.6	8203918.58	11474713.77
206757.9019	185381.6973	202211.3295	7031778.835	2741443.498	943596.9078	415641.4906	2094042.309	187025.5197	284863.4342	173941.6569	181808.8798	173883.0962
591176151.8	1040708019	734914251.4	686667555.2	513187368.7	859622175.1	671064874.5	591198148.5	862122719.1	1019263247	863813912	197452165.1	213997020.9
41480557.6	39011766.59	39638027.92	39995267.89	44032242.77	44860176.49	29431926.39	28010989.59	26406360.7	27028678.23	26369258.98	30047481.84	32359117.02
688900.9313	390924.9223	1075918.998	10664766.01	9320277.477	5539166.316	1442883.363	6655062.427	2179515.327	2894130.737	2608273.885	2418970.363	3488669.368
7955054.219	8148214.051	11417701.9	9687622.322	12518399.25	14914760.79	12261130.25	14116350.72	10906036.48	14039778.81	9603762.822	9299255.232	9282710.418
26461269.29	32423270.75	14919107.96	197472568.7	223461256.6	132747283.2	206451392.7	125071469.8	171169449.3	147149286.7	143774105.5	150110628.7	154560272
15796093.36	15757238.77	21477859.9	5985868.586	3935759.581	1472275.106	3215441.084	2876643.14	1816426.376	1490307.34	1657212.908	1655667.816	1151259.193
31028749.33	31465462.59	23859296.97	33266108.71	13655826.3	7173506.828	6113751.549	23789137.37	13012990.94	38177232.67	7964609.648	8886771.779	13528733.98
5448839.19	5521233.272	5004222.992	4818676.48	3687753.628	1275783.603	2839191.633	1373403.796	1905586.574	2598714.992	1686215.287	1451356.807	1776075.362
90162333.36	101192818.5	69611703.02	10150775.03	10817125.18	15943100.36	18500720.85	20060661.23	5464395.038	19197070.79	6007690.428	4440195.992	6744198.63
3481223.172	71827887.62	7020263.075	3612602.186	2749004.832	3912241.234	13088580.6	4139222.512	455406.8628	556923.0747	800883.1131	2799977.118	1672204.324
369091.9904	452116.1238	282659.8293	1512722.156	981103.3353	565192.5434	428741.513	364487.7252	358890.7658	494149.0144	277315.1952	426171.9072	375837.324
45262391.98	29836832.64	60690074.24	49542523.15	40491534.27	39173671.15	33116047.02	45281336.13	15461045.23	27273574.96	21705632.19	15475882.17	18614939.08
55841585.76	41789626.56	70203464.19	51510214.82	49988556.42	22044170.55	33159334.34	59609558.47	77094685.5	164331542.6	41043477.1	42376944.93	16360476.93
1802496.832	1789427.493	2226754.275	7733422.054	7484631.652	2424528.339	2029032.548	4111808.924	9940488.515	14338882.78	4989776.621	11479596.57	12241983.04
814788.9591	820782.2962	596625.6094	14832418.59	16620403.22	10087671.67	13186299.53	11200827.32	17143827.25	6557239.585	14656107.88	18156015.55	15407209
551676008	413919694.6	437234922.4	438107098	497231827.4	404051278.7	485610258.6	602131256.2	524909351.5	509682884.9	550303841.6	506305119.5	483170939.6
2723937.83	2999910.823	3510372.308	3636961.254	3956536.234	2604182.825	3641699.183	2453596.234	2595665.847	7722265.566	3688561.625	2690061.832	3639448.68
473623.2048	475591.6272	486288.9179	510458.7171	581591.1669	452404.0575	476375.1603	510157.9672	436644.1203	2431962.431	456162.5899	504157.7622	501852.4094
1897058.984	1161444.146	1588405.742	3219666.264	1843671.132	2358985.198	2418367.529	3973909.03	1256859.406	1065243.642	2668947.769	1320051.834	1486690.713
10363991.43	17038980.84	17437680.45	16090104.85	16038215	14643949.37	7064969.024	11219476.25	10360172.11	10132775.08	13814806.5	6411754.656	7701749.708
23531627.07	27807004.46	6932989.432	30924052.89	30864918.67	40245434.32	31714660.64	33258713.93	18146923.04	6972709.491	10498509.35	15847859.47	13109463.46
8139484.785	5683543.159	7440330.644	4597361.703	4713955.409	3834585.489	3666403.658	4851334.675	5325364.756	35708961.54	27707557.43	5989244.172	36846395.85

all_YP1_3	all_YP1_4	all_YP1_5	all_YP2_1	all_YP2_2	all_YP2_3	all_YP2_4	all_YP2_5	all_YP3_1	all_YP3_2	all_YP3_3	all_YP3_4	all_YP3_5
2536360.354	3326361.721	2007678.375	5854636.478	6346207.106	5519032.62	4380662.192	4999361.09	8175511.575	22133173.53	14450174.62	11682395.5	11685862.55
6118469.308	11736304.51	3493223.826	4907994.276	3107398.442	4524881.483	3258224.169	18025478.53	22932454.37	22263055.13	3501880.851	49555606.49	31254279.74
1012405.161	947331.8389	931729.1336	878459.4903	818284.2373	757621.0199	649676.5364	708412.9401	7701791.744	1324914.765	6332754.32	4011290.21	3610354.891
96245440.86	112283919	23960433.81	265164467.9	286779919.8	254799729.9	265983602.2	258491085.2	200313838.9	71998209.17	148617764.5	182029267.7	142225378.7
478895.702	767159.5232	355655.6043	917997.9081	828387.1157	460770.7463	508522.2856	575785.761	1701926.505	4593102.237	1033525.844	1633690.749	1779273.597
5351632.762	8034589.671	5586086.519	3647750.543	2084961.133	1586992.321	2708395.054	1660288.9	1296099.299	1777208.949	2146963.874	667084.9389	1206127.019
625409770.9	788641654	574742068.5	1286090159	1161001098	824407823.3	815826028.6	899329629.9	849428490	709306501.4	528138606.8	1008896306	977423438.2
1487232.646	1393271.612	1665199.482	2434078.527	2712805.385	804521.3286	1864158.812	1047953.548	1669058.129	11008417.86	2697044.019	1969457.862	2825214.537
15365433.74	15497325.75	6571387.608	17435436.7	18903241.71	14624071.36	13248613.91	8411236.154	9584733.113	8937544.345	13834688.49	5554453.849	7865989.8
24105219.55	8672369.005	28166538.05	17913212.96	8997199.942	15001990.44	17644056.65	40496841.02	9769449.312	6032957.009	45390254.06	39078120.96	53721596.55
4596424.741	4940652.134	992768.4905	4473472.104	1491705.413	6671447.552	3293158.905	10863272.47	13573839.91	14739729.55	4772055.277	18429926.35	15621032.12
21427471.48	26697529.48	24401320.76	71746840.64	78336130.27	50945464.53	51066761.34	56503092.17	60867988.09	107930987.7	49514974.09	62712106.16	73780503.23
2184400.134	2101718.414	5498387.745	17701652.38	20218534.78	13847682.98	10298845.21	16053542.25	16766310.4	29230109.57	19367688.88	18565788.59	25951526.37
4358823.264	2565026.118	3878089.406	3142081.609	2370063.21	2515245.557	1408638.688	3047896.647	5203799.289	3106797.397	3903876.308	5852220.858	3858819.679
164000191.3	228212995.5	145657789.1	357908779.8	314852106.3	162278547.8	164235103.1	181397593.6	318870465.4	2460168106	220908554.1	286032113	340771832.6
7060494.236	867039.7914	3853246.068	3253656.947	968336.673	940746.8088	2805126.525	4636284.15	784702.1476	824714.926	942654.4623	526972.7296	975491.3035
210302535.5	190932094.3	210692688.9	257992279.7	268095377.6	194099545.8	243880300.2	215283923.1	372121898.3	403936487.5	324973131.2	314859974.4	329038591.3
84504879.16	36285492.01	92005856.97	48166289.79	31680443.21	37433820.78	58305650.29	118653131.5	50710821.13	44749890.46	111079851.2	100147328.8	91028563.38
5790427.739	7181993.725	2361108.981	4120641.351	1841140.805	2566986.134	2095245.784	13392786.41	11083186.54	9931253.406	2901030.194	32122160.53	5548439.998
1090053.157	1261516.37	1073153.531	6034016.312	2810939.197	13157311.72	4617706.339	3704851.762	67420143.05	3672154.236	23116636.43	29371857.41	33620958.09
2500135.963	3144035.963	1973230.942	4650789.164	3309347.909	2219625.716	2425216.25	3841407.402	4309979.643	9793150.239	3193136.255	8521597.798	8643453.74
10600386.89	10440024.74	11674961.31	12390203.49	19467830.32	14461046.5	19959084.61	9779589.917	7761092.606	6665088.918	7472546.61	7853505.991	7602678.763
2264148.545	2488841.208	2656625.121	1344474.162	1690422.594	1824498.347	1272730.875	1995990.314	2050622.262	3358841.008	2269974.134	2247722.237	2619252.563
33809344.32	31038690.63	37561999.39	37008006.27	51956001.07	59651964.01	60424574.21	61741595.28	14494686.59	5929946.293	5942068.543	23224780.72	14485661.96
8932503.992	9024351.848	11583906.95	8864776.708	8946614.49	4567246.988	8570543.571	4215401.895	5542299.063	6002996.067	5655455.632	5211024	6300436.667
69011795.94	93830396.78	112414450.2	99814812.53	87301075.85	112049898.5	84807635	58846751.03	90040873.02	165232718.6	73271892.5	82942288.46	63262187.14
718128394	592701285.6	840211560.2	144034649.7	78344759.52	15914639.12	50713137.29	63990959.55	17375744.99	15633532.22	18181882.45	26429280.63	11608706.61
5425335.575	4749721.193	8686683.63	26736140.19	33306324.41	20986048.75	16148547.94	23730831.25	25943137.71	40726525.39	27316757.65	29667671.84	34517275.86
269321.7642	290272.0101	283183.72	240450.3698	239183.2879	233236.7979	221930.9531	269331.1459	2117215.33	262609.9042	217398.3077	288113.6272	1326786.341
3640943.818	3625773.208	2948047.057	4893802.638	4623159.382	4229594.357	3944765.279	3983617.341	6825313.891	8840721.161	6851229.295	6773161.218	7367544.97
2658025.672	1710864.936	3971335.706	2517175.334	30292459.11	26784266.28	7358759.915	3580552.823	5608628.79	724922.5091	1619533.526	14355569.07	1060574.385
1999106.964	2012252.713	603618.479	2757560.261	3290353.873	3976442.749	3030319.373	3337782.956	2789139.761	867734.9429	1643257.838	2379367.363	1807156.773
3493086.558	2323581.613	2679552.335	3193644.461	2347437.213	7607415.48	611585.8996	873523.5693	1776147.618	954769.9103	1030810.165	1191826.314	1066200.913
2055281.908	2352780.274	2351486.368	1650207.427	1497166.096	1706736.199	1539745.136	1701803.734	5138452.057	12277472.64	10706848.97	2733203.534	6121582.842
1867631.626	1603940.17	1485113.37	3925197.026	2679879.652	2080419.231	1976168.667	1821613.775	1341180.82	1538238.554	1671111.663	1635951.026	1734857.059
327912.6497	408418.2797	1443958.668	3027522.2	4833509.218	2357917.65	1072805.638	2578635.831	9180825.289	7170540.481	5008878.346	13519866.2	19534710.53
5763601.133	3832181.836	5259576.361	17319123.76	12250546.14	11977989.85	8515642.361	15058785.42	30783040.64	42943893.69	24363754.78	50862437.75	42932755.21
32232912262	29870291583	30236150517	5939381788	7458852051	8063738301	5176161463	7956943092	22724550091	43580879816	35494167317	18059846388	32194131515
652293.3713	736101.1597	843491.4565	560427.1026	2395213.843	587872.4574	486646.0836	546693.8012	549474.8029	633940.0296	581750.7899	336363.8563	579156.6474
3903117.3	4793941.305	3516438.174	13892000.68	6167505.607	9114394.046	6878806.534	11069224.59	20587735.23	87871415.81	18154730.34	17727436.48	30230404.92
63389566.71	70157836.35	71576809.1	56317454.61	67153389.17	63003338.48	28748342.3	35224412.43	33646384.49	39530941.3	33490376.37	48554097.49	43524942.9
8705059.703	10123968.69	11670741.67	8943247.43	9931146.189	11366206.68	7591253.328	10104132.28	13675326.21	13739058.96	11639158.47	11527928.74	9066065.026
1611847.443	1682400.384	920601.4648	1294042.85	1548616.029	2878034.194	1676330.861	2544499.878	4467795.52	8177334.107	4853375.875	3520854.906	3592195.969
4637240.439	5933780.706	4425731.356	98189607.87	68695373.87	28020432.33	10019508.89	41026711.79	35781609.93	44151202.16	26894456.91	47928254.24	59891636.93
179998290.1	81248053.99	232018099.1	137870058.8	85571425.18	159840304	143733425.7	274919024.8	72851278.48	200904347.1	340816449.9	189639891.3	172670179.5
268376.3371	281571.3528	231432.3198	1756260.906	1191300.744	492848.2624	434580.7529	567652.5937	3578241.581	10184663.08	2676787.518	2480030.718	3876204.994
4976753.734	8628514.321	3484612.524	1263822.738	617054.2973	934836.848	1586588.175	699319.62	307452.9074	2027559.252	1530250.107	278273.7083	350029.6452
1381861.223	1822956.426	520652.6823	1628113.607	967378.5379	1064468.266	609552.865	1726607.883	1506107.37	3362226.253	722734.6238	2613128.966	1669331.091
4161366.544	4596118.83	4849673.132	4868351.509	6525969.787	5463220.617	4511846.568	5318348.844	4391395.927	5534383.775	4833265.864	6677372.825	4583304.3
507855.209	502749.2184	560699.2817	503460.6722	511531.1038	493192.7499	675569.825	683915.881	638610.2562	1028863.012	815921.296	433624.7269	442954.15
36630322.78	34044487.76	36142265	39731248.65	39036959.49	45810046.66	36788021.93	44894207.66	43120889.58	24897711.51	36809577.33	35572347.19	35424495.86
38344640.66	7994942.059	15822685.27	39220523.9	21296924.31	4427204.546	6359153.567	40222116.71	41621440.15	72000721.68	3659301.827	66108693.7	6819020.828
20930294.51	37256467.15	31265807.6	24269077.82	30861082.3	32001633.78	45901740.63	7118501.638	34417055.24	20719854.44	39551034.17	22762772.46	30946220.71

all_YP1_3	all_YP1_4	all_YP1_5	all_YP2_1	all_YP2_2	all_YP2_3	all_YP2_4	all_YP2_5	all_YP3_1	all_YP3_2	all_YP3_3	all_YP3_4	all_YP3_5
12082343.24	13277392.27	12978396.33	2746236.333	2066263.785	1895101.508	1938558.665	1722016.75	1979829.643	1898942.241	1937948.6	1756903.12	1933119.251
14898516.45	19415338.83	12128427.4	18272718.98	21090354.75	17469419.4	11920674.94	14735902.22	21609879.92	38205283.82	29527154.24	25727717.46	26217495.19
156174.3548	136184.1526	128801.6176	126347.5367	458453.9839	377800.9276	351991.6289	1131714.085	1072620.56	204896.4672	168095.9623	4156505.568	409928.2025
9471262.651	15153267.12	9074326.628	49381217.03	24799731.44	26262486.86	13373331.98	28902942.32	20941670.82	27355381.38	19109427.15	21423360.76	26483684.48
10313391.34	17947958.06	8784861.546	25489207.21	25227900.61	13570488.52	22755637.63	11962588.22	16994718.74	12375034.91	17978897.48	16511327.32	18906919.64
3694230	1937935.174	3916122.823	3695197.019	4009621.129	2940411.898	2954872.678	3219278.842	5558452.746	5819494.048	6051785.09	4640965.727	5056125.421
1583296.404	1163099.466	1359544.336	3048532.459	3109418.116	1513362.773	1166558.944	1552306.615	4259050.277	11855817.44	2951418.359	6557894.288	5363677.894
750377.7491	837810.0153	757606.2449	853315.4838	596338.7802	1494584.427	1169271.139	2192938.094	6937127.488	3216238.986	2894663.184	11039090.74	11681322.87
96278235.67	134358448.9	67922115.38	128387803.8	147053968.7	61418237.47	15609105.86	57319851.16	153391835.7	156321431.4	88054228.66	93220983.11	82818253.55
7468240.487	9168818.398	1938994.586	8051571.423	7050630.682	7406535.822	5181137.015	5219017.649	3647644.309	1392877.605	2003916.118	3929286.657	2274238.663
2507157.702	3046712.415	811055.6441	3242968.045	2077305.484	1507674.29	1679800.345	2513932.28	3089848.062	6262312.378	1434937.316	3846884.954	2303410.377
92653071.31	111219317.2	188575845.7	152553860.1	95034453.64	154882819	92840684.78	66276438.16	140300513.9	117174083.3	93281342.02	95407826.85	81482984.93
746425.3113	936812.2475	962203.8508	430234.5957	1254694.866	1009594.855	495300.1415	853616.5487	421826.6186	486669.7757	848658.1846	1160197.83	1402559.946
1007049.458	876092.1237	241046.1576	3667316.802	3468530.971	5021598.535	4429209.996	6390741.666	5306347.85	7324617.136	3470411.027	5049280.516	5364485.305
9141818.308	8443735.771	8356327.438	9592831.456	8040494.071	6496990.63	5699457.328	9420702.813	6693085.73	5150632.482	5784249.461	7480442.27	6070425.541
4445032.86	5317465.613	4934806.463	9991694.173	10511257.08	5112045.229	5967377.758	4012763.025	5197692.061	3941954.303	3792356.946	4200867.587	5717592.819
2927590.17	2844172.698	2650885.044	5100836.844	2098841.186	2798804.763	2736290.794	3248586.713	8121168.084	20736563.4	7313368.813	11667568.98	16458419.51
4076380.551	8063197.248	2189331.957	8135525.821	3958997.942	5030070.976	2363479.758	4580725.471	6401034.227	4655564.988	4355623.091	4784431.804	6688667.577
1531589.677	1998623.672	2601768.396	1401458.899	4435750.042	2617853.949	995680.1129	1676288.092	1179701.937	7978750.123	3197155.768	1051575.353	975571.7598
3621738.956	3036750.821	4318152.758	3523761.565	3936560.575	2478097.999	2862603.727	2291835.129	3076887.124	7348081.936	1960140.969	4543732.601	5861698.863
14775181.46	11184634.31	16036867.87	7589775.857	5753647.763	1117720.139	5261314.317	1961852.042	2276251.293	1925622.061	2069932.6	1438377.9	1947286.921
3180672.027	2572433.56	3670773.616	3268472.314	4037957.428	4082960.084	3086726.186	4042105.657	3448862.244	3241587.127	2774225.253	2955116.54	2774290.44
1267310.616	1533243.844	975634.4542	6816824.514	8987750.296	5947943.606	8045790.362	6603919.408	8160127.22	5010661.61	5630000.426	8493913.555	6356724.824
382814.8256	354591.1515	745217.3897	619070.117	343318.386	360190.0318	4567529.129	749922.8448	353262.4483	357579.8611	398332.8919	359645.8741	369960.6091
2014075.445	6153286.663	501225.6487	573408.6691	546431.1486	502065.0385	579584.3734	5551995.466	473661.6517	489520.9186	498084.2949	15527939.97	586345.7374
14510685.02	2124849.018	11724324.6	10430123.56	4641892.678	2390866.31	5717439.989	10787912.99	2164705.256	2070523.892	2260466.475	2282205.565	2035119.682
1386053.54	2352701.98	756292.3372	7520033.771	2153396.054	3863988.659	893607.9392	4447198.099	11472479.2	9398606.842	869610.6854	4093655.976	3230343.433
1083183786	1298339381	515817938.1	1262741563	883451218.8	799831829.9	697949882.9	1373211930	1250684130	2492226740	684176340.1	1778620060	1283380960
414628.4304	550479.485	311784.0343	872404.1867	663138.8294	610368.7213	584448.9227	533720.3573	957562.7739	2145658.073	368034.3058	1507727.072	926241.4643
3584404.296	3438177.802	2256087.753	3494309.746	4234165.531	2887239.932	1974310.906	4464339.646	2066846.606	2698492.542	2328823.775	1775036.088	3771771.071
6117353.694	6958276.363	5452511.129	5511912.57	7161110.861	6222178.359	6054062.465	5055316.157	5079196.979	4405980.625	3804090.955	4966728.443	7219523.341
17597925.66	10782195.99	9930135.027	13555929.72	8164113.451	4695410.691	11380116.59	6697452.12	11316555.96	11155960.21	12586234.79	14445803.65	9152925.946
2172641.907	2036604.831	2287080.048	2133772.88	1865723.54	2392990.699	3426983.113	1923423.824	1848523.387	1911586.568	2717277.868	1904646.601	1884435.075
13013031.23	14962238.3	2072905.514	13871537.39	4812522.012	8942434.088	6622540.886	19865988.88	17736795.53	15876957.17	6321215.148	31958618.67	24298711.54
2118257.049	2005141.749	3058265.989	4311562.499	3952575.16	2301912.737	2554251.573	2432692.63	3617261.164	9077435.046	4134176.871	2525660.345	3592724.464
143169441.6	371963156	309266828.5	213024706.8	209402098.2	201857902.7	309738587.2	84803291.46	189795375.5	205807658.3	249499722.9	155682070.8	257454506.7
7041111.669	7502559.534	5219737.408	2396096.261	1380757.388	2308824.012	3392891.243	3876506.661	644514.3214	4130664.392	2793062.004	460403.7757	1035510.869
91999929.17	102531889.5	122267085.7	83873392.76	98138569.49	104744152.7	93669888.16	118381328.5	70790292.32	52054161.08	137865910.3	112348228.6	103376874.5
479201054.5	369354489.4	532874179	545666910.7	394757876	370201047.3	385449145.2	479484248.5	306689042.4	316991633.3	581775470.8	566880342.4	558780766.5
2298390.816	2025460.048	2616417.693	433707.8685	1493674.248	1750482.379	1407689.633	1669621.047	1829730.504	1852240.016	2003136.891	1687285.189	1709770.214
1056041.876	1065452.657	1601188.874	3838985.349	1612456.748	1734398.783	1770582.541	1091023.981	23207094.7	357047775.3	84508618.87	22819349.61	64755133.57
911768.5586	565359.5891	2120943.782	6433856.755	8562189.392	4919430.67	3711423.632	5457599.085	6081083.452	9662591.098	6693071.217	6835042.934	8532026.519
12475805.03	17609737.21	11169616.69	7723983.591	4975587.528	4903208.873	6168623.865	4394430.944	2937834.763	4850209.04	5822812.916	2164341.105	2713588.614
91419666.64	142074600.6	37488179.25	95828655.02	68667417.76	78469023.85	60724288.44	139211311.7	108121346.6	222618806.5	67102137.22	179265258	114180066.9
172345.2821	173717.0846	170594.2577	218743.1474	215869.653	217334.9803	221400.1534	246912.5878	224430.9237	606527.5567	321090.9284	215220.3425	816638.1601
5894455.126	4143866.991	9453651.24	6255902.005	5431144.029	5691785.876	4711287.527	5201809.465	3729734.242	4079057.228	4399776.991	3071182.191	2961500.04
20181030.58	18896384.11	16651515.5	19015388.51	16319794.58	21326203.48	29782320.16	19984653.94	17153584.84	16267271.17	22533069.96	16000720.79	17961007.48
8841377.903	7155994.713	1315176.507	5233446.093	998828.2819	1684270.376	1215240.761	7377117.924	5367787.121	976184.6909	1074638.559	9342679.587	4415128.859
10927104.7	13799881.48	9179638.959	22332151.85	21736484.33	14255077.25	14955346.84	13296008.99	13796194.96	11447305.32	9180367.999	15011244.12	18398951.35
505587.7567	540777.0276	706904.9554	1503044.823	1258612.929	563301.8869	858495.2743	584568.7119	2277402.142	6558579.142	2808756.272	554059.3941	2652244.608
81454973.8	89886868.09	93877373.67	101149912.1	70357159.82	94547360.11	116498825.3	83336915.28	71378717.87	175039625.9	85979321.8	78949311.51	75139990.03
3375654.485	14089399.06	3459545.649	1705474.375	3135426.591	3756796.398	2865157.826	15492921.88	6287134.683	6821850.174	2735704.028	86634922.83	8499417.153
7713299.694	9169417.83	2520012.355	6113196.409	3350395.466	2207637.536	2865988.967	1677474.403	2499592.262	2801273.166	1585784.805	4672552.24	1858036.985

all_YP1_3	all_YP1_4	all_YP1_5	all_YP2_1	all_YP2_2	all_YP2_3	all_YP2_4	all_YP2_5	all_YP3_1	all_YP3_2	all_YP3_3	all_YP3_4	all_YP3_5
2467285.284	2034306.352	1939011.327	1806020.356	1934190.599	2912847.633	2158763.89	2409789.96	2191392.764	1773323.178	2044406.594	2620071.165	2089147.931
26320808.64	29940661.95	39951771.78	32577339.1	35462027.25	35349909.08	35254977.26	32777040.01	25622496.49	36363989.12	26733175.18	10031480.5	11125001.7
2171841.852	1725086.394	3371981.406	3531359.998	2488796.411	2757199.096	4434439.939	2391890.576	4842425	6841208.825	6801335.043	5419958.636	5267891.415
938692.7456	895591.6986	736885.9106	797739.5132	832482.6905	699087.7587	843259.5593	693792.5682	1736016.3	865707.9457	700181.9336	10334959.53	3303511.527
480610.2975	459819.2232	436137.925	2638972.139	350843.1109	8724982.797	596509.4855	4111963.888	559456.0542	683935.7207	461977.9633	1683862.724	646538.9864
3028998.712	2841553.935	2888186.169	2116993.783	1849046.444	1269934.57	2164855.161	1231544.021	1147392.175	1283603.896	1226931.971	1077840.684	819270.1396
640392.5945	668908.5451	577593.9569	674843.9852	657587.6019	518272.1566	632857.4692	576567.9199	693280.795	670879.4795	478772.4993	10123762.05	1156981.88
63499.14633	58193.91402	114492.2683	127633.9522	56126.51633	63278.83595	41649.09517	72734.4487	59865.84592	54226.54668	75672.37786	55775.27212	51076.95124
302745.519	339400.8773	296495.9117	321173.2555	302528.6795	1064833.472	286071.1509	1107395.85	2005407.404	2704007.081	2266797.85	1288801.365	1905163.89
146796192.8	108917764.4	138340833.7	165567433.7	135620173.5	112806672.6	146330676.5	141807209.7	136296390.5	97215804.96	166301601.8	143563800.4	130646365.4
995413.7224	3366487.999	273579.0033	19112635.07	19676920.74	15702082.4	12917334.51	18053731.5	20407672.35	22965255.12	18172007.15	19928282.74	18478505.45
7619568.336	8981508.856	25377051.83	35466307.78	9867124.869	25156437.2	8782786.524	30445713.93	19932631.41	33427063.93	2271608.84	11788414.47	3012288.13
4977021718	3700467613	5178752837	4574832340	3456972244	1733357680	5511761598	5544697971	3780161020	5675746739	6384988046	5065690004	5420087176
392705.6789	372041.2504	4116538.801	385984.5178	498470.4968	909328.9383	332206.9269	1149580.606	355962.4087	344339.7528	286710.485	375736.2459	343856.1862
287718.6021	571138.703	433229.9405	1921047.78	2273711.282	2056833.614	1329564.277	1985495.477	1744516.767	2804705.895	2042787.23	1990687.02	2143115.756
60211863.38	13480873.64	13653212.66	29596319.07	14686835.61	15241859.49	17192869.33	40146591.3	34311939.81	17270242.35	66733646.81	45639461.29	13447958.92
1823520.076	1995272.991	2060718.385	2223010.1	2063590.509	1547947.931	1770516.112	1611454.306	4179572.093	26173080.82	9284912.836	4536924.206	8958334.165
1500536.917	1293720.469	3264161.936	2237858.181	4203563.284	3539722.338	4208107.865	3800783.296	1182221.843	1263730.456	1199395.548	1390484.035	1284269.978
355150921.6	583996981.1	720199978.9	477560609	548959078.1	808286506.9	482433109.8	508553451.6	634604038.1	500782201.6	460761676.2	453402755.1	470035964
297521.5014	334022.8094	303768.3222	326674.0558	330015.5951	350950.5002	271064.2068	357922.4414	371671.6382	2857285.236	306951.7678	352223.427	342910.5843
1509040.33	1885688.995	659328.019	2009186.333	734253.6643	1462361.804	864322.8264	2415807.942	3870884.998	3249482.614	1093140.154	5405043.238	5396617.319
11832532.91	12437400.29	14269654.13	13487750.18	14103242.22	10672115.49	12084038.52	13509583.61	19375358.09	21514653.36	16798870.56	18081508.75	17889266.4
11405818.27	1307825.288	3836675.825	1206610.914	1194455.942	1350405.137	1073419.196	2728335.087	5513927.893	1202300.984	1138103.78	6900985.431	3535844.38
88903607.4	93122070.05	75854742.87	105538295.7	96879397.71	97539092.16	91815088.08	82600749.87	111809055.7	106732178.4	90359902.6	82497214.18	76655599.72
21002003.78	24315039.99	25900843.88	20359949.34	26913257.52	28140005.07	25722063.63	20568976.63	28719867.2	23928952.51	23550048.9	23327388.41	26315629.65
570631975.5	390241108.2	16922721.73	431359238.6	459049607.6	423630336	462589434.9	564908027.8	33611458.84	416815739.2	523462945.4	445295953.7	450144239.8
21293625.54	22094856.91	19185390.13	14455030.55	14925657.64	16718067.24	14473695.39	14987431.36	12021962.16	4806773.182	10224724.06	13514148	12130192.27
4686159.183	5731979.489	6762150.792	6035739.851	8790348.159	4501665.892	4774101.692	6917319.874	7671096.728	2485399.517	6563373.498	2685135.153	5381574.352
1309468.547	1753525.096	1567423.826	2003362.938	1655098.829	2430138.059	1690760.894	2379553.236	7602290.199	5266336.233	5984541.754	9319027.434	9199151.044
4107610.023	5436706.406	3689546.434	3645992.121	3573586.451	5056718.849	2434242.925	2153342.107	3300271.603	2186949.043	2444007.171	3035901.914	2936933.631
375554.7236	394019.2818	358223.0872	630245.7566	730109.668	707723.5148	608134.295	900340.6307	806550.099	649353.4594	1188157.285	2935949.547	9949984.448
387445.9193	803230.2235	744582.213	878670.8163	918781.1469	893984.9624	494222.9236	685697.032	516912.1291	416165.9389	443229.6398	453144.8356	470745.7132
6916026.69	7246864.031	5917452.979	15077958.04	14887389.96	8278621.622	9240614.731	8338296.192	23986824.33	341918946.6	75233210.13	43754730.2	90844453.3
155518.8595	165911.4685	159895.6457	909148.4809	923350.733	680544.8417	538078.5336	785345.8698	1049659.93	288019.1416	806052.2594	1002831.291	566040.6481
303295.3055	224944.9084	209980.4834	2006763.336	2328652.481	1285719.864	822940.6494	1431570.254	2085719.849	2798264.899	2756957.557	2984918.024	3113459.469
32464538.04	32449143.63	50016579.54	25029154.72	28411148.68	20198540.77	27687376.14	24640490.4	12105442.25	44055199.52	31595785.74	28697905.1	34181792.34
7434519.459	3422539.403	7472874.807	5283894.6	1815842.89	5042035.518	8767458.26	11621026.48	1181322.365	1368511.8	10831214.21	847793.0376	3407909.186
42547402.51	37915767.5	26152354.8	49650552.08	70233091.48	41786706.97	61944426.72	62760186.43	314192407.7	57892626.57	63845894.97	74521781.24	67276031.73
330575600.8	393749752.7	504090555.2	242832035.9	311440301.6	420986712	297433852.7	259972305.2	317029973.2	276723766.9	314375192.2	242224348.2	271623378.5
1152945.911	1083103.626	347206.2444	1197225.784	1393571.621	377800.9276	1658813.484	3013490.697	1072620.56	763477.2166	1617620.724	3286181.887	1652510.311
1472713.61	345463.9897	1532235.645	1237954.678	315822.3036	800437.7947	495180.6653	522294.9848	732178.9583	276818.9846	605994.4568	628801.7313	513654.2275
1218879.267	1205148.495	1214738.383	1107774.002	1165651.837	1367669.604	1221065.813	1153875.924	921546.3451	985304.4186	860916.1177	3665131.858	937044.9427
586713162.7	456903917.3	550772183.9	657798175	523274974.2	383545342.1	462274694.7	526599247	505384988.4	641045991.4	537854989.5	529978330	436991554.9
13075555.41	2130886.703	15785306.09	9023370.968	3674386.188	14017999.74	7506175.944	19338711.69	2236578.74	8576966.46	22392909.42	11848571.01	11248957.19
4958063.709	4969965.598	1686385.234	4241345.995	2495608.719	2415744.719	1836526.001	3009314.303	4524016.159	19545922.8	2704116.009	5092839.111	3972040.852
962218.3787	13257181.88	9330090.429	13012724.63	11732223.83	6227340.558	8359415.353	8173354.186	7202523.259	7774676.329	6167367.746	5064695.559	5967324.178
1938935639	1169665581	2401799460	1277651014	1171324713	985905667	1326029565	2753708354	1733130147	2105970982	2914846394	2233530972	2312289576
864754.3949	782176.6876	743773.5945	665618.2615	510815.9021	794065.3201	616830.6886	505378.3861	575704.1814	500139.2091	604438.9668	582625.122	637790.786
1986844.626	2571331.032	1833246.417	5710829.478	5608852.722	2711066.397	5388716.189	3505661.574	3743777.521	2877672.254	3376231.416	4971425.51	5350581.071
4426938.858	2662084.117	3896637.31	29492621.32	21747724.03	7822246.408	5601485.642	10581788.72	3896738.693	13878424.87	6136747.827	4194588.921	7543689.932
606959862.7	510349343.2	704835780.2	856041690.2	573058427.7	386275951.4	470964870.4	584647600	447000962.1	490508554.8	690750912.1	698584943.8	657723955.4
342920.1869	361984.201	311638.8895	1259926.712	1311076.576	1949982.539	1717286.478	1551856.337	2583837.283	599165.3897	1638232.419	2137737.313	1997868.463
6215437.971	12361652.35	4642553.81	3441566.536	3631493.662	4844683.724	3904536.416	4749525.704	3061462.841	2110820.282	3425543.638	3180524.254	2392291.157

all_YP1_3	all_YP1_4	all_YP1_5	all_YP2_1	all_YP2_2	all_YP2_3	all_YP2_4	all_YP2_5	all_YP3_1	all_YP3_2	all_YP3_3	all_YP3_4	all_YP3_5
78158967.98	93695888.72	138224262.4	90672376.85	120402364.5	93839938.42	99358713.17	116464687.4	69462226.38	123861617.8	170499972.4	65554578	78015062.88
632595.2201	683378.653	498992.7938	691073.2291	696873.3031	770390.6061	733717.4678	733722.4072	3082057.604	1028866.593	1683896.132	1038348.949	1071036.848
1084892.583	1014407.736	878531.0191	2308861.02	1918028.433	1415243.617	1086486.067	1264539.103	1196174.211	11290686.55	1415230.324	1267772.201	1347822.854
18972742.64	2347363.364	9063310.25	14508215.07	10613657.39	3440432.334	5713337.908	10947742.74	1115939.431	1831382.051	1874577.257	832827.4941	2111674.435
549274.2564	600257.2751	510699.9015	340096.8456	332975.2652	413244.5611	371910.9681	395364.3785	274803.9196	3088273.979	314814.5208	288767.9098	324382.066
228145155.6	317939043.7	367641095.3	266564903.3	154754241.8	243940351.2	264866279.8	215458051.1	197197499.5	234032866.5	253789603.8	222349614.5	190715021.3
18888622.41	18881942.4	23486696.87	2204222.58	2204001.225	3371702.87	2921876.315	3347704.631	3491316.086	1739590.844	3228585.138	3429415.342	3020418.285
17780406.84	32225076.67	11412163.44	19943928.75	23030871.99	18015855.14	10299430.18	16002513.43	24669274.47	28178046.65	34106591.43	29552484.13	29353068.62
3667048.869	9674735.866	4009789.544	4126461.215	7809333.108	1381624.623	4775570.282	2086283.476	7131558.603	9402446.001	3136387.366	6126846.205	6297449.751
87444052.71	79902792.49	82428355.33	81864247.83	69180275.63	84197335.37	114161313.6	54731223.53	61401432.67	66718550.19	78449075.53	96923476.12	73079474.1
1502194999	1524795056	1636762997	2304849109	2609147132	2427619950	2237841398	2343995812	3024526894	1412879004	2511792498	2680139574	2577306466
1289088309	1383579520	1071860371	5229925857	6538237595	4944463612	5462733433	4490710586	6136947399	3229007792	5451607897	5847567140	5443534832
12603406.55	15424951.56	14691605.06	31476136.9	32831042.93	25897357.44	19084174.41	24969929.97	32807341.21	21986922.1	20672345.46	34295841.06	34875108.48
6404160061	6458410370	6898020205	6294709739	6605962554	9352088610	6441872647	9974349533	9267634044	12249298916	9054215875	8442444754	9307660204
5451075123	5648476709	9367665539	3968840146	4508152456	4823911048	5643031292	4229722832	2268661461	846087321.8	1636861089	1644614703	1349288845
159993528.3	155187036.9	167318174.4	2216619184	1368463426	469876346.4	503029107.1	473102197.7	5737802669	10912188369	7358549447	4106908033	7062438707
5415453491	5541693003	5968591469	8640970810	9808423909	7828070872	9706433822	4581164105	7941312156	3451969587	6414640322	6638772855	5354175982
3302683315	3598558638	3292466419	5406701764	8982860858	9067614375	8666808975	8024216944	10424406948	6247007441	8715980986	8527924870	9895750319
5727668525	5600597605	4867372887	6163012663	6616287495	6397322491	6885262500	6503609599	7434366508	3221657739	5810640423	6279634465	5800404078
3646083.022	3758337.307	2067721.093	9708879.421	6433933.877	4210445.687	2562617.585	11504390.67	11385828.41	11823115.82	6076392.956	23602327.96	16480564.29
224716449.8	271284232.9	189689529.1	716589968.9	575631578.4	697846286.1	412455200.7	780313241.9	721898983.5	545424470	435908501.3	1112964079	849061833.4
7714600673	7657723782	8356110885	7435035895	7567923773	8249338636	7910031403	8705788155	8127167247	8848216794	8213205610	8355295703	8300596797
76838300.34	93825743.5	42551984.94	112377792.4	78041123.05	81837940.84	58668881.87	108090830.9	182353100.2	256236188.5	78948717.4	186241174.6	196627850.8
389761932.3	369747245.6	445350476.7	456668166.2	443916533.9	412046930.9	389331689.5	423805577.3	540016979.7	922742060.1	546591827.7	603987709	492066452.7
316180767.1	337744536.6	239081717.6	1546500272	2037319080	1671526941	1852206972	1518240192	1573362538	310110955	1165772799	1664418940	1243293013
44808017.36	55272282.7	45750088.04	211853317.8	239129710.8	239311086.9	223466858.9	220615669.8	354443429.7	89572765.04	278406335.4	326891438.4	268190248.3
178163713.4	238444335.5	125098038.4	199794708.9	162849608.5	131805538.1	108394462.1	139261266.4	285976029.9	881285198	211787844.9	256340311.6	351682210.5
79740819.24	86435544.86	63499977.64	330933655.7	386406752.1	418345083.5	341900008.8	384580985.3	410401196.9	184467196.7	361002958.3	387210985.1	366085410.9
5415453491	5541693003	5968591469	8640970810	9808423909	7828070872	9706433822	4581164105	7941312156	3451969587	6414640322	6638772855	5354175982
701487.6988	572124.1485	663101.9227	50447960	43716276.91	773444.9679	895977.3747	760673.7552	1996426.561	29173202.29	8970158.755	880377.8696	20804230.45
621038483.4	631699336	555849889.9	703582925.1	892486311	731662942.8	758533020.4	789052246.6	1051834591	506497612.9	869682207.7	1034369074	893896304.3
1065961740	1044103779	523557580.2	285106725.1	250151525.7	510045670	281527642.4	448717022	11197350.24	15203606.28	11774220.28	9913719.235	11704597.21
288073565	265113011.1	325641806.3	263283997.9	233669245	252926394.4	242651797	327630015.8	396483779.1	88763198.29	348238544.2	349992841.4	313046878.1
15439975.4	15719024.08	19206789.91	3741990.732	2934176.509	1200011.409	3451904.556	2340024.615	362457.3241	1215927.272	1201287.669	802573.8737	434465.2601
110827084.7	117644556.2	89916021.55	258152330.9	295334694.7	219680671.1	255930138.6	205441544.6	228704841.6	173297601	200549236.1	201296438.2	193648369.2
255889.0531	215569.113	302344.6061	197413.3668	212146.9955	230052.2354	243730.6963	201226.1157	293852.8735	189779.9332	470880.6575	393104.405	395628.5201
173844725.4	172100351	216366736.7	158012136	160670099.5	156249185	179390758.4	183947994.2	147542936.5	152097623.6	151802407	129905234.7	145858625.7
164494935.4	137704882.5	216236484	81915202.27	71145616.91	152277645.6	96327525.22	139371816.2	74664090.53	69112796.44	62587603.97	57051374.62	60941596.73
488598330.2	531084701.3	504978951.1	731715566.4	1128346322	1147802906	1074845015	1025788509	1304182907	852874298.2	1076461275	1083285520	1239040940
47619273.93	63690063.12	12964511.02	54299987.88	60075284.48	141771862.9	64991478.99	107648336.5	71605741.34	11081284.93	46035727.18	72313207.19	53255945.98
33099872.2	40068127.04	27113531.53	119969555.3	111192272.9	124554737.8	80664863.3	151368502.8	151028106.6	144823513.3	72151153.81	211659133.4	167397363.2
45212655.13	49485464.6	45734629.7	27976995.18	30417881.17	55436662.87	55001299.71	56323467.03	34943085.74	25619700.76	38139791.65	39470549.29	33205563.54
136235327.1	151097591.7	153827194.4	132848150.7	144152953.3	69651952.67	132128226.7	67695986.83	83833439.94	127495814.2	96766267.38	83305388.21	95092042.36
149308053.1	155418587.4	114642365.4	876042678.4	912626749.4	917012933.5	903297097.8	850003193.1	694906804.4	149665196.7	433726160.2	615959045.1	447129724.5
6973248.741	8028727.593	23943622.97	10665049.05	5222141.567	2421540.185	6694264.662	2800881.919	4762877.354	15867536.67	6320159.937	3870596.768	5098314.56
119823363.8	120923573.2	112496683.5	182819016.2	196284470.1	55572074.64	183731063.9	168656831.7	174910080.9	50972736.47	141946298.7	185530707.1	47387519.17
73708849.82	79174262.1	78648423.05	253362261.2	236527330.2	140196814.1	130040227.3	144871707.4	160934212.7	139127599.9	82525970.81	202503746.1	181161776.1
315474235.7	364606410.7	114136022.6	199898328.9	164943118.4	130343424.2	120788210.7	227620788.9	217221059.4	528041805.7	149551323.1	298656851.4	206631239.2
204734523.8	232919983.8	118216797.8	287735673.6	350849459.9	370627523.1	355069548.2	366769915.9	227712180.7	164215910.6	236496996.5	232679367.2	211856906.1
64882406.48	68193183.28	1704599.255	63221023.93	69124996.28	68111076.19	69110316.9	81956575.37	78834851.85	73002194.82	72325986.25	74146873.2	75556617.62
22328835.98	26132958.3	18152619.49	47489485.2	44563737.79	25078489.36	16434444.39	20552335.81	24172577.42	21036563.55	15399459.03	25873171.81	32440676.33
43607822.15	33830289.49	59246186.93	23451054.57	16775945.13	19225897.54	18752939.44	23319616.36	30652532.66	50580305	31075542.72	33857674.02	31419520.98
8588081.578	8193770.044	50294566.59	12250300.3	9155396.569	30479903.75	41715750.2	15608859.09	32178141.78	12048725.86	9776502.458	8199687.121	10770005.86

all_YP1_3	all_YP1_4	all_YP1_5	all_YP2_1	all_YP2_2	all_YP2_3	all_YP2_4	all_YP2_5	all_YP3_1	all_YP3_2	all_YP3_3	all_YP3_4	all_YP3_5
14394717.79	19806587.51	20010564.27	119007368.6	119487150.3	42391549.79	129804436.7	36837980.2	71693802.55	39712909.9	63306153.77	64657044.57	62673408.23
473008372.1	541792177.1	302072653.6	85107462.4	90692178.72	134443229.4	118855641.7	135547011.8	57287117.15	15966661.07	34794367.46	56572271.09	33123288.48
9207572.312	6791846.507	4449005.297	7272994.555	8777712.036	6336101.418	18037265.55	5744340.43	7200521.93	9825973.392	8021750.356	6685472.139	6654471.022
5686192.249	7021026.157	3134216.979	11262506.43	6522870.312	5750346.686	6857324.584	9373018.187	19517696.56	27656546.65	11743539.47	20063166.59	14557314.4
159563784.5	134385629.6	56098699.81	29801855.77	24146314.57	5092153.847	24270510.26	14854013.31	7756188.16	10881895.76	8707284.118	5800066.293	6902962.428
1074891.614	1210626.224	998472.4022	2212939.566	1999770.415	2345348.22	1247934.078	2353257.483	2904332.019	13692529.34	3233911.563	6784215.528	2948847.473
157290378.7	164446051.2	160559854.8	125373027.1	215731460.9	318980718.9	275383640.3	293271695.5	360744385.8	84724305.82	253824981	295928391.6	288235827.7
249285985.3	316201749.3	112137251.8	178731765.6	206632137.5	314168564.9	181934965.5	274473778.6	188450653.4	48562137.73	139214190.4	190269950.5	148523980.6
174326446.3	155187036.9	179655679.3	2216619184	1368463426	469876346.4	503029107.1	473102197.7	5737802669	10912188369	7358549447	4106908033	7062438707
108703504.3	132694122.6	43570737.07	333143361.7	362026308.5	394472039.4	345331697.2	359801179.4	413342336.1	143811217.4	368059487.2	428751979.9	384546598.3
17094810.14	16885403.12	12796052.41	32013580.85	29437951.01	29935745.35	20347777.04	29783356.3	41452874.71	23583176.2	24028485.52	45626233.17	41902986.55
7747369.259	10402312.54	1513043.873	6743040.562	10455981.97	4558922.627	4741353.619	7315951.37	11231774.11	19846511.29	4187738.812	13432249.21	13482164.41
5273055.657	5893316.421	5191304.903	38205946.35	31657735.5	34473489.46	27810247.69	38092086.73	117068325.4	158633845.1	97203049.97	161086236.6	156565073.9
145080737.4	224628295.8	114818057	261968130	208135901.5	174867784.7	116153126.6	195604438.8	338153779.7	1950501530	278847211.7	308718016	457336811.3
14745152.2	15815766.35	16147488.71	50369255.21	65165045.22	51828332.52	64377338.44	44889877.37	64545939.24	51821040.02	64931578.78	62235775.54	65392062.91
72650550.41	81588930.07	80058008.46	49888439.06	52510326.28	27115048.59	58022685.1	30932574.71	52999252.47	164931521.5	78000753.54	48969913.28	73907261.62
1130741.373	2419866.514	1135285.886	3069184.151	1747574.149	1343046.144	959415.4308	1612440.621	5604878.697	54186422.39	4932919.6	4281716.649	6945645.048
12954254.55	14482006.71	9442179.415	9253104.999	9057410.084	8355751.473	7250357.923	6249093.615	6489580.436	12134574.65	8213779.078	5027559.186	7339626.2
3214771.07	1822222.069	668597.0357	2358600.702	3280911.608	488040.4532	11436845.35	4969343.186	2931561.112	2866928.343	428803.2038	20143326.88	1669635.28
11919362.7	3154628.399	7330790.451	8049101.355	2252956.537	4699195.153	3883490.194	6192720.102	4865221.585	2425820.302	3521329.157	3584282.644	3606258.798
3067000.631	1217946.415	4740531.207	4300162.492	819419.8325	3012913.489	1178008.79	2430512.769	2486911.205	651764.871	1520675.28	2973000.139	1941923.058
14776750.11	16582229.89	12142435.29	38999320.55	45111954.72	37287292.85	38961178.25	36922036.4	53050757.07	28552317.14	41655966.92	45459481.54	42992653.15
38008764.53	59602402.08	26581414.57	70323547.05	53514512.86	36714916.98	24713081.74	34840974.9	69461339.52	353674564.1	57254278.77	53964972.42	93855573.56
79442132.71	114834300.9	34063440.21	67285286.52	46369514.5	49974879.12	39283396.04	83020074.15	71309054.25	112692643.8	41969463.89	119464526.9	79368814.87
1788949258	2256910950	1822594405	5447401525	6216406401	5578004913	5401175700	5212879918	6442724050	5131612598	5206528517	5722536563	5707122856
26673431.78	33712582.05	31734572.68	46151629.07	50059310.87	45666205.74	46825799.54	47857904.91	53965325.83	34320223.99	49892158.77	49450813.31	50489282.01
16857518.17	23244183.98	11763864.14	28637866.55	24965013.4	23352163.35	14255186.34	25584692.27	45433665.8	338449710.6	45370691.04	35174551.28	66914391.81
33141504.3	46017235.72	48626166.19	199638396.2	141509738.9	69878201.36	41446823.94	66797287.96	33717707.35	74400413.91	35125405.35	24004947.82	43480993.9
63385147.36	65291544.93	57831749.4	58312547.81	54755266.3	60546408.9	55938555.63	55121802.53	53057238.93	83008517.8	55967399.19	45709587.32	54428278.58
2066754.651	1983064.147	1511115.431	17614272.44	17568336	10449822.12	6335676.857	9712887.835	22646608.88	33792942.01	19319196.78	37152027.43	28018660.24
11401988.08	22214904.77	10572264.05	23858483.78	16611194.04	12488096.32	6568025.99	12277288.87	38271261.03	298569908.7	29492435.47	20788013.93	50403528.48
27528911.21	24604343.92	11781440.06	33058042.64	46903872.58	16292121.18	24478686.74	14578661.32	14975979.52	18588386.05	17344317.83	14836770.78	13572523.77
35515180.03	28746482.64	14574006.36	36402727.74	47024841.57	78238655.24	84142597.32	89670327.28	38393345.68	17459768.75	42250754.65	42631095.95	31765651.92
7605153.178	11342934.42	7435682.841	6074467.954	8324865.896	13435674.48	3156476.996	11199340.06	6638373.089	935409.1209	11113562.67	4297785.83	5877727.402
40726317.03	58923998.51	9216133.435	81796587.51	93978317.78	150110421.6	119477649.8	130252371	97223814.6	33916660.4	73497192.25	89189252.87	68533559.82
9506540.412	6882099.364	5157660.793	5803991.601	4080798.597	7383332.132	4158071.642	4801237.833	4552639.067	4505081.08	4003141.547	8500137.175	7564727.027
6336385.532	7595738.445	5417764.126	14990047.88	13270067	8599334.848	8837411.72	9446610.345	12511653.51	8174153.499	6937123.723	14168488.15	13019449.23
58088288.9	58564055.26	54922562.56	35441475.64	42862974.19	34011831.69	33038909.58	39761017.18	59850421.57	50166317.93	59784358.1	47269257.3	80804273.81
49029358.67	52349298.99	30537591.2	35496527.51	41994914.23	53436852.16	39660835.49	48433015.78	31194155.16	5297797.198	20440469.87	32026676.46	21416218.84
53636653.61	46911804.97	35979653.35	52019842.69	61720531.96	56964848.95	59690864.86	64896259.75	18750005.63	13088274.47	23519446.46	39100997.22	20221203.62
77791024.67	93650041.83	94291238.73	61574182.23	50991621.72	30314394.31	47118110.62	39841380.72	10518617.38	20068071.36	10204581.05	6509178.683	12243676.68
17679437.79	21950641.61	7556450.778	40868136.19	46581837.21	50380827.72	45397027.51	45505899.63	36989705.74	12776388.22	36608433.35	44082539.56	32858009.11
1130119.15	1088037.279	1481113.891	2044139.356	2155867.215	1808507.676	2088406.198	1391689.401	1240181.837	3536374.248	2352095.303	1255437.942	2219284.448
18324236.48	21016152.28	6729775.497	21622398.48	21031116.45	23387099.1	21979794.23	21531023.71	5772495.697	3285782.698	7272950.124	8022035.508	6015068.159
39487729.11	40545620.48	54272420.75	57439983.02	60419132.16	51139935.1	48225314.74	48177251.69	69511274.65	69949506.31	59449017.5	60171960.78	49445271.58
31635895.74	35518014.04	36603833.38	30111482.99	37671559.83	43298433.38	19781345.08	23545112.98	20945570.05	28023147.04	18840847.72	22237371.18	23826627.04
5154380.589	6027993.902	6635058.133	15216947.57	16196096.37	7057056.589	7994734.493	5842284.32	5197617.805	3239329.756	4200476.818	6776004.963	8151253.168
395704.1412	417929.6936	410197.9221	1676981.703	1634140.116	959929.8889	637686.8596	1940961.261	2936548.038	3227714.166	2818703.615	5804905.481	4847216.894
16036571.46	17069484.06	16866838.63	45667440.56	29764510.36	17373824.69	18235529.63	14313939.34	155142127.5	131372944.3	112192624.5	66547566.97	160922736.2
5251468.622	4052443.599	3982998.178	3868750.146	3856679.135	3225230.28	12323134.31	4553204.769	3278695.147	4267025.001	3208365.505	2636454.409	3199847.69
5027492.371	8402091.938	3683034.783	22965255.78	20579506.87	13242468.45	6954735.966	15426301.96	33704960.42	330207226.5	36363139.18	25712616.75	59250362.76
7580430.766	11554794.66	5135876.327	6776542.695	8896480.986	6832787.675	5407215.701	6408827.863	5746700.038	16920801.14	12175610.24	12539830.44	10663778.54
12459691.69	12213977.27	12712282.73	6940357.578	6122070.384	5380416.246	9100355.317	5661474.218	5105984.007	5215293.127	6577470.042	5344963.143	5453416.664

all_YP1_3	all_YP1_4	all_YP1_5	all_YP2_1	all_YP2_2	all_YP2_3	all_YP2_4	all_YP2_5	all_YP3_1	all_YP3_2	all_YP3_3	all_YP3_4	all_YP3_5
3807552.462	5322873.661	3271110.668	4361290.096	8009510.345	5624248.648	5012924	7133833.823	5797889.863	4736699.177	2729394.242	6733490.608	4645126.655
7232554.718	701112.8165	9025247.21	8687488.516	7303132.853	4969674.588	5502029.663	9424549.582	574553.4145	1012966.977	1042081.139	616616.9155	603336.7176
144102.9947	197070.0207	129998.124	269173.1309	474432.0203	299927.7386	275396.3224	295045.8255	372577.6794	263744.6458	430831.5857	321033.0597	308823.6904
38411479.59	41001336.79	34531624.73	25850588.81	26674238.83	23995130.1	22078637.21	26234176.79	21323785.43	40238084.46	27662089.72	22626568.36	25948913.21
21153349.05	22724026.66	27869082.7	36224296.8	9996852.323	11729453.66	13924219.68	8042043.494	8459550.688	37492859.47	13758868.55	21758916.62	8564992.782
2484325.132	4844015.192	11448623.06	4903146.841	6517239.414	9340606.907	9791404.087	5508975.184	7817168.296	3817163.906	8193320.071	5358655.453	3300868.27
10221402.8	10077951.6	9344734.221	1865018.658	1681812.505	13932442.85	1879551.687	12523911.08	7266884.533	6941740.577	7697456.923	6511804.296	7186371.357
7534047.075	10203764.23	6005652.988	8590207.844	9294630.36	9608454.718	8265524.914	9069594.847	9215265.562	9582523.231	9056340.215	8133319.449	7768094.206
1483679.755	979893.1407	910693.9291	4047398.782	3089864.623	2471004.631	1642803.051	3584432.721	5350554.781	6157249.373	5094704.443	9794808.259	6558681.872
318596.8593	289919.6616	338038.9275	530076.8149	227871.0373	545268.0156	259689.1236	1313760.287	3124033.712	1135030.452	1140862.785	3939728.553	3232915.306
4738533.142	4269644.035	9153556.489	22631503.49	27074994.11	12516751.19	9388857.002	20087351.62	20640570.52	36407996.59	21384616.29	21172870.68	27203359.85
1914080.275	3145200.87	1589953.075	3551318.148	3347574.275	2219988.483	1777774.995	2231841.41	239444.3011	34617818.63	3090218.533	3142836.878	4871085.025
16422349.54	12810332.69	9562424.059	10270175.63	13043614.99	18520620.23	9279896.738	25023225.8	9286361.737	12653120.29	8104132.947	12642515.18	9910744.224
235552.176	229542.0102	256159.1717	3024300.07	2176289.027	388141.2417	211460.8546	665919.9727	220877.8318	829337.231	240464.8167	215013.2073	240436.3837
5200559.713	4975101.332	7604065.049	580927.8901	634287.1247	767054.7017	756233.146	717773.3661	398157.163	860981.1485	381667.5847	367108.8651	387246.5254
3709019900	3584315476	3738197652	2918128321	3595118022	3897198688	2765045116	3335762996	3063163896	2433192293	2861263582	3427862017	3415906492
3562265.593	8497110.131	8123710.789	59091547.86	45508668.25	17803451.75	27943425.21	12970052.14	27088068.05	26261788.18	34466103.51	20954672.8	32348055.38
77694385.93	60051544.77	186987589.4	58232632.32	26293516.83	81966670.79	48974247.63	38583419.07	133682095.2	40781155.92	63467385.02	34484132.1	99240982.37
30107693.61	35054895.27	30825106.12	124486120.5	85086858.26	58651264.39	51411331.92	45894388.2	57165470.63	170109839.5	67622054.5	74679398.27	87425379.44
39026136.57	62193045.47	35650623.44	230899349.9	227923023	111632611.8	165742522.2	91049222.92	197597761.7	195414800.6	304830603.7	240559621.6	234323361.9
2167314411	2435151831	1751709858	1207560116	1252128601	1154988700	1469028914	1164887196	878543373.1	1084667783	928412536.7	917332588.4	900171964.1
827033286.4	182937899	836791699.8	1458469452	233798540.2	441128495.1	475360858.2	806096012.9	153598477.7	247974458.7	247477188	150833856.1	247807132.3
22836592.12	25869997.07	13177004.54	107721831.6	131191912	120472713.9	136269547.6	103244400.9	127165689.5	196687859.1	194396072.6	188057734.3	203847326.1
243895741.8	295389861.7	193719303.6	371001632.4	354978497.3	211148480.6	213861987.7	208571156.1	385204342.2	2230489179	299631478.7	298454159.9	470757669.4
544358367.6	650212910.5	467268202	1125195806	1095753537	893665617.7	696168419	891203484.8	823007458	605033097	570190987.8	1044264810	852927111.3
42583923.52	33405096.43	43314227.68	108174572	77012380.01	63313994.87	63670403.56	48406954.55	56648496.41	102512892	56754816.68	91239401.7	84548060.72
150755608.2	158110805.9	184983344.3	492246456.6	422166124.5	225624508	233745588.7	195524602.7	179259951.7	160329990.6	139069978.5	241132240.3	196186379.5
26187534.27	27452943.62	70054175.72	142235192.4	105121806.4	1928187.118	192004748.6	184800349.4	490016058.1	596981136.5	251389406.2	612258247.8	657057746
37988165.43	62786221.99	51954059.56	269485482	251947453.2	145340789.3	198217757.4	123839045.3	210492307.7	203627314.1	257607570.7	210579886.4	242419249.6
9663387713	10077778276	3101969982	2940908551	4025886933	3654313457	2692836824	3558766823	2866182992	49814226.85	2352021360	2749930610	1856315362
322987087	325569940.7	405373003.9	311126790.3	343044118.9	178340376.5	340027040.8	159662797.9	206210766.4	287316695.6	247507367.1	209050631.3	242919890.7
1608406.568	2925430.228	2587817.175	2392958.501	2273113.254	3613726.448	2108907.707	2436948.638	1580916.722	1526492.799	2307332.45	2056942.225	2130189.664
425126961.2	70022588.36	388585793.2	305880102	121988779.7	93609435.42	203347027.8	392942411	58464622.46	87519364.82	95010401.24	56301620.81	95523102.04
850303.7984	872018.2375	1383638.455	1048284.649	2046431.825	6952991.629	2352561.749	5197806.563	6740176.796	16980788.39	9439691.735	7055395.373	9811759.09
55184361.94	25723267.72	34469232.5	48125712.42	23948393.85	29472185.7	15965128.93	11171941.64	14783876.87	10830306.71	14113218.37	18986511.37	11111240.67
6207269.564	14533403.32	6773758.316	45630442.83	28640179	30931495.5	20339951.29	53586043.05	124208986.7	82290798.2	63507128.36	172943683.2	164224004.6
10305259.56	15007966.51	3013397.707	19809481	13471168.52	30739655.84	11652200.17	27533084.7	359912075.1	539097592.7	475347650.3	237212082.8	337292924.1
96669530.6	106866912.7	94391024.88	241845576.9	191383991.9	82722333.36	118491957.4	117368842.5	71979249.92	419131319.5	87064309.05	130087221.7	123992457.2
58123264.23	63345703.72	43211611.2	130619500.2	125120458.7	108116742.3	75915773.71	104485775.3	94182907.89	75795155.07	57757546.73	135947674.5	96010232.88
13789542.11	13533177.42	26881926.42	121916602.3	109945756.5	44579161.55	46810368.5	60991989.62	20809865.67	66623797.29	47954128.25	60785650.23	70210405.42
24379716.27	35973682.57	21815943	327276820.8	263759894.7	147948197.6	179551538.2	114413424.3	2580249849	2361977280	2404648477	798844705.4	1904439108
6877918.203	8271126.963	8203890.564	79241909.99	62843123.35	47312433.54	30248646.98	55130719.41	39700877.93	117623593.4	50454989.69	52455865.5	63645579.75
253215237.3	265674944.9	266737993	435877794.8	415559757.9	235695544.6	180709262.3	213333003.7	264112071.1	1888187014	238648586.9	211805879	263704358.3
9915610.097	12225293.75	16100988.81	37440927.12	25106997.13	11804203.54	17369229.88	16042459.92	12826128.14	65553111.09	14886370.43	29256213.42	17214347.9
11244898.76	12821315.53	9877538.218	40634851.52	49323505.8	56527876.02	43959070.9	46447894.17	61144201.11	47987871.54	64679818.49	73187246.08	63094737.82
124490349.8	207383736.4	61023254.49	108899347.1	71658089.96	67673121.66	75336103.63	134480860.7	80683620.51	106014393.7	54614671.4	150900037.3	82124906.77
1360461.334	1995811.401	2130797.232	2437654.449	1115624.546	1513307.396	846824.0219	7706906.333	4006516.656	4446989.735	1950650.905	11677788.52	4764150.26
88803444.18	75039805.41	122066628.6	120595337.3	123443814.7	200586842.2	124711675.2	173895650.7	191752008.9	247052591.4	183805203.7	180198297.1	162586308.1
38553484.1	30228411.68	35561798.91	104761032.9	82157991.31	54973961.3	56406243.56	48489839.54	57742099.1	171742402.5	64980058.86	73991746.44	89435755.64
471083209	530424500.6	450184350.7	70742314.7	100685936.6	126359425.2	113742169.7	123940751.3	12755643.2	37414717.37	11703170.38	9954172.153	14348790.29
7323224.902	6903048.932	8963541.873	29641552.24	22924754.49	18189128.66	15747566.5	19025008.84	18030422.58	41287094.61	21901461.06	27127668.47	26849661.95
205052907.7	234548645.9	185683590	389948946.4	325234192.5	145508546.1	125731720.7	165108629.1	186052250.9	1732364644	167207702.3	177479380.8	196446524.9
10904178.6	11097077.97	11462262.01	28739523.91	21717840.85	10968321.84	13471301.78	15140917.57	8772771.999	40662363.88	11803190.93	18268202.35	13696670.76

all_YP1_3	all_YP1_4	all_YP1_5	all_YP2_1	all_YP2_2	all_YP2_3	all_YP2_4	all_YP2_5	all_YP3_1	all_YP3_2	all_YP3_3	all_YP3_4	all_YP3_5
1077961.516	798001.2089	852488.6734	28258239.74	23776922.31	5584624.439	8406148.151	5092635.32	16535403.21	651480506.2	168631938.1	74009364.29	146008082.5
20861325.86	34970869.24	24527586.37	84683846.37	74822053.6	82446448.99	18772199.4	154799252.6	96732027.2	92546831.16	42454006.43	212282264.1	113618313.5
9225179.481	10317966.53	11232606.57	28354521.33	23276795.91	20109531.16	15838046.07	16307608.47	16199166.14	24447426.95	18433056.6	16595582.3	21571833.03
7269877.72	3281092.633	3952799.473	2944786.387	2937587.55	230502.1871	3222362.872	3493039.696	3427036.814	4122387.304	3913619.43	4106931.206	4436137.825
949381968.8	1078504385	1149022296	884640918.3	952890371	1212077149	963338811.4	294784274.4	836859590.8	694463801.7	929451173	853088451.7	803535741.9
18925170.71	22907194.67	14813117.91	18749932.44	14709631.09	7971325.737	4450265.629	8830936.184	7856683.204	60599539.92	8585335.486	7431150.6	9648800.75
1996074.327	3903331.077	2130915.263	55298880.71	43560687.46	12392141.43	9125146.485	20562451.4	28875429	22607545.67	19362033.31	44855651.27	38643492.43
47843706.6	47205928.33	48539695.6	94475714.83	89818349.16	42981714.32	38973817.6	45804600.11	49420633.96	408661227.5	44571418.33	47911920.73	50939543.9
11817998.64	15956854.31	18691805.96	62800244.85	66062137.55	65875600.9	31214280.36	91275042.19	129119248.5	154799323.9	121079313.5	165603813.6	186739852.6
11561780.73	11085385.12	8500071.746	25397921.35	18198440.47	11432742.14	20152499.47	21015117.64	12717129.23	46815933.17	19939532.97	30806781.61	17454634.37
18493352.54	15991464.76	15111999.14	39554841.11	35541177.75	23709719.11	27533297.59	22411358.83	49583199.3	4829062.285	58931720.77	48919606.95	62356327.83
12822407.56	4421663.632	13699447.39	11664590.9	3592414.036	7395014.471	6137975.595	11504526.79	8881945.036	4067429.588	5423783.094	11602404.84	7613354.339
14572953.08	17363092.91	16869813.8	60026654.26	55453345.5	54938934.01	37597901.86	42919600.65	46314159.16	90777896.11	60257979.32	48778320.17	67074646.15
232805197.7	258166946.9	199165312.7	113375210.2	107125429.4	85636773.23	73086665.39	97714294.8	102699485.7	320100169.3	82552017.43	90107366.25	100133857.7
4284668.995	5977535.839	6067197.861	12588250.2	13432726.35	9554225.449	7617493.017	8560952.632	22809399.46	214748672.7	20296361.98	19505936.24	27257183.95
24676556.55	26701536.76	24689813.09	60953059.62	54895103.47	34707710.27	22159411.11	29983779.05	36314412.88	320103929	31677790	32360145.95	36515948.3
201454568.6	243656640.9	93543196.74	172600320.2	178414790	233340105.1	152648553.5	211670208.6	145620603	18194738.73	89344231.23	154102916.8	93031058.31
189913441	272909850.9	534095293.1	209977528.9	261059500.9	138649662.3	151552366.9	60966474.85	19212669.32	20065386.99	21029822.75	24574755.86	23764186.55
42903855.05	41326964.43	41224803.62	124041656.8	102088132.9	56977956.2	93645004.41	86489432.72	127643737	79663440.92	72023379.68	180328661.2	106565167.1
138179750.5	155854880.9	85354186.57	105167054.9	126894174.3	154265534.3	141591071	142230601.6	61811698.6	14597267.2	50414350.2	63934356.45	48743888.3
926458440	1005601552	1074532891	1047263204	1105227690	1186174045	1096201468	1154007005	1103678856	274687911.3	1122255788	1104263412	999950872.2
46714228.71	50110071.03	37500312.35	13915855.86	14112923.3	21665297.36	14374578.57	21833625.47	19357193.3	19305169.36	18790060.81	20340164.22	18114107.38
2425998.965	3108917.499	1087565.97	2893765.488	1579472.339	998824.3541	856099.2473	8523630.798	2643364.706	4033903.495	2014818.575	13450585.15	3577375.414
1716429.601	1543321.911	1359589.265	8841391.666	6098548.361	2133316.378	3209573.902	2519646.546	3482857.855	24506362.82	4256065.779	5562265.519	6805882.484
96731778.99	110994686.2	122679580.1	92288104.22	81076773.22	109264274.3	77323639.93	112273586.6	113076475.2	93697599.07	137837003.5	116008547.6	120170466.3
3665749.284	3234369.239	2870709.199	27454998.8	22734429.25	2269655.079	5862919.651	3475807.451	4058159.089	11806148.91	5488752.734	10930388.34	8006876.395
165856052.4	201467708.3	133575103.9	77934607.2	77419483.09	35602373.93	65179957.16	42746220.68	21465236.44	24671630.44	21953941.51	14876714.08	20747894.57
5142046.424	7540154.674	3106018.93	28833655.66	21757807.45	24736747.36	19329621.34	39019200.86	90330672.83	101257308.7	76447047.23	142012289.6	114840774.3
11613562.28	9871056.502	10274426.41	24086707.13	20035560.69	13522956.09	15146259.28	14483029.55	32988189.58	192197829.2	25128395.38	27928974.23	30143281.64
6412486.443	506516.7721	7156188.52	2906370.5	477467.3597	461492.5219	3515067.256	5546626.206	530025.9043	588767.9447	524691.7106	543241.9986	537050.3322
7077046.225	674402.0626	5663729.079	4891406.167	663626.3325	704390.4756	3135335.838	5480458.677	331808.8536	722869.7995	984083.8773	497221.5168	646507.4612
6176932.334	4805754.729	15327386.22	4913327.481	2355436.759	8020081.34	3707163.439	3453494.936	12635424.86	2342923.356	4976863.699	2802655.219	8046490.935
75785448.63	93035437.19	87122643.6	155443678.8	181761894.1	75051944.85	100344446.6	73211442.8	72566985.53	63806839.82	49940597.43	74555261.71	79844953.43
47737457.66	78942395.62	63000719.97	102549114.5	79026789.63	48869839.9	47292367.77	42101957.96	49912040.72	292650828.3	46011731.11	40689812.26	47959977.52
173300229	224156786.2	168270399.7	297386066.9	273286961.4	213742294.8	218146111	239134624.4	209127704.3	117082172.9	148867881.5	221412735.6	209573798.7
6780418.413	11228007.68	6055939.099	15584056.11	14446309.08	11257503.94	11720251.02	14191209.74	12484370.93	9852903.732	6856199.397	15030990.06	12469674.69
23185069.14	13876519.97	19491926.27	15195691.31	14181239.78	12992924.32	11501424.57	18316147.55	7527255.569	10255849.2	10847146.88	7217687.81	10026323.14
10095350.37	10564517.03	10886184.32	15591839.82	15465494.06	8302050.952	14847607.64	8522463.595	43969033.69	81845609.29	59109292.28	28802203.4	45962557.57
429687746.4	467756668.5	447154790.6	218883099.4	258112925.6	329464991.3	224166498.2	310389474.7	35082221.05	63897599.05	22176052.14	10746644.98	22251592.54
14036730.71	11122845.34	26210971.71	172479389.2	62736609.25	22994365.65	19248158.19	83129829.44	93194234.09	164678316.8	79957140.4	81122094.99	122668872.7
1492611.576	2013203.478	1269278.291	3147054.466	3069793.813	1851090.791	1774250.546	1676114.311	947263.0324	1885619.035	1837039.395	2053754.879	2225001.39
7817014.369	8088942.724	4535095.379	16170089.33	11256531.36	16358917.83	8319679.614	21778759.01	24055497.99	17213425.17	13393542.23	37069698.25	23725680.49
7797150.673	2398727.86	5532322.047	13386687.34	6083503.484	4319220.82	3451330.188	13574460	15700372.38	15517089.33	8074676.61	22367460.64	20762487.61
565932.6277	659217.3045	1244155.798	6272615.225	4683085.183	2578098.109	1968392.895	2825769.11	2079972.601	2208723.001	2531966.936	1841155.748	2977707.028
4424061.588	5552709.925	995208.4517	5079434.87	5727274.111	6062134.936	5939811.916	9996865.089	2972235.701	2154540.974	2526933.624	577863.5004	2282974.074
1528786.258	1674705.099	1584189.378	5744454.742	4155466.28	3203496.214	2500529.305	2053032.608	1801474.9	7885098.13	2391058.826	4527069.46	3690337.854
24556313.35	28125405.61	29978945.87	34958092.72	41127751.94	35137091.14	47855955.35	38606778.27	37091262.9	5374754.293	31803292.79	35242993.35	19897688.2
6232810.854	8241489.335	2552303.235	15201609.91	17902947.89	20693054.81	20597503.68	19046416.61	13608317.35	9173573.319	12257157.7	11928157.31	11353587.46
1708487.743	1881649.848	1896078.135	4857217.561	2247555.379	1043050.547	1064038.362	883762.4081	1722617.763	907054.8159	1614481.435	2580545.185	1336654.221
3454750.468	4226200.817	3463379.458	8767466.034	8627293.071	5741070.656	4649631.289	5270406.219	6285835.475	5423919.059	3900469.36	8025057.83	6144277.524
9264158.497	11023352.62	6161695.583	10537937.82	9301577.161	9173543.771	6831698.378	8744260.616	17016174.47	33778242.3	9376209.438	17689988.11	17076119.51
11967573.27	6029422.365	12225237.43	13472608.52	9073636.544	6203873.406	8860496.612	13149222.95	9409251.317	13655012.91	17214862.46	14831646.32	11052000
433670.3402	386545.0257	530103.3414	5446540.699	4495908.013	1422184.147	907561.9692	1215813.376	963752.2261	1004507.066	1022508.237	831955.5369	1220507.608

all_YP1_3	all_YP1_4	all_YP1_5	all_YP2_1	all_YP2_2	all_YP2_3	all_YP2_4	all_YP2_5	all_YP3_1	all_YP3_2	all_YP3_3	all_YP3_4	all_YP3_5
11475426.94	1897174.12	3533295.222	9496159.794	4733557.244	2581215.413	3382311.951	4501058.088	1858231.873	3028563.521	3739653.043	1750199.387	4009301.788
121657335	143668623.1	117051029.1	75089976.18	83142292.61	42040045.83	93230123.12	43803741.58	42243581.17	40901497.42	45518359.04	37661318.52	39852208.44
14698183.18	19429299.19	14093922.18	34491354.67	32317992.34	23184976.93	17237411.12	21895459.49	20912100.28	17493357.03	15181917.28	23573259.32	22456800.13
315717.3898	311914.6657	1244329.89	630136.3122	756853.3618	451440.8494	935780.2731	776133.7042	1068926.588	1638711.994	1251726.706	1172491.257	916738.7554
431959.9619	815465.4596	695380.4359	16806084.9	12237310.23	6864632.13	4726744.358	5133807.06	5659326.334	15573537.26	8482208.374	7769896.013	10529369.87
707887.0124	746522.2416	688878.7034	5640152.203	9977370.043	1623600.653	1120960.545	2161182.539	552018.1483	850063.322	716432.6146	624462.6161	771221.0176
4436488.574	5631346.526	5264502.724	20869190.7	17341455.8	7450696.359	5633859.667	5654528.38	13291539.09	318241139.5	13844002.04	8572197.659	17017473.68
9426920.178	10899256.13	9151356.011	18312310.04	19934178.69	13793244.06	16626525.5	14344611.74	26097657.9	303837924.5	85771656.58	43472591.75	87102066.65
6411159.53	8196136.712	8867070.447	23525628.71	13098789.25	17550238.74	10571744.78	28041568.7	49271330.06	28283188.63	23960377.25	63327837.43	56681378
34747766.22	32179575.15	50338749.63	20804370.12	13020755.23	11448437.16	13113269.57	10677335.8	51160439.3	180394909.2	55571175.63	40145936.97	74093626.06
18420152.61	16282771.73	15746802.69	13340416.99	12757492.79	13403330.32	16049512.93	16343031.51	14443379.17	15660605.23	16752678.91	14515446.03	15899667.23
295693807.5	351324911	268905071.4	871571677.6	792032037.8	455920071.2	485004067.3	553375583.1	410263231.4	389972179.2	319000647	558101114.5	406372854.7
2424013.876	2840392.62	2637618.831	6635600.824	5645367.269	4001610.596	3674101.865	3758049.866	4287292.63	8601373.542	4572136.555	5586215.941	5582736.629
102876273.5	34106073.01	24103670.35	52447920.37	62265882.32	318697599.4	131157219.5	92645338.76	85450256.52	59355085.4	86766577.79	167037408.5	82888527.77
9939799.168	8259563.368	8938914.359	6060599.41	7030069.895	9217440.296	8358765.383	7739375.112	7630488.837	6562118.33	7446099.034	7490976.112	10718373.84
10918943.7	12380542.31	9602943.958	21312790.24	23018984.92	11696879.49	14103569.35	11300195.65	12274436.81	19575956.41	9228842.654	14928282.88	14015136.98
4653735.158	4555470.239	3049373.584	3598536.334	1705140.829	1255805.157	2603514.536	1039571.791	639525.1198	1040083.248	1450852.663	1835562.456	527303.987
6586187.357	3882994.51	4135918.985	10354390.31	5571996.914	5236246.57	5247829.904	11143815.14	21034839.92	20883192.62	15245967.7	34748868.46	24870146.84
348626653.3	303627824.9	345313857.5	749883210	635794330.7	482908897.8	421481303.5	399478022.4	430206476.1	1294392281	464149592.2	579417244.1	630697618
11641210.68	13195651.41	20370358.84	14234181.19	15879028.38	17745049.7	17588423.93	18562821.29	15265117.07	20043672.51	16129004.38	15066015.89	17043293.79
389806.6302	588776.4869	222001.3578	370092.9861	150442.7414	139141.9237	152820.078	1993160.918	329324.8479	2206714.771	187044.882	2604756.696	377783.9762
48093783.36	68612183.74	12787281.16	70057810.6	76323878.85	105434533.7	86642988.14	97478816.22	56546850.07	28075573.46	52655725.61	53741468.3	45815869.69
5406879.446	5315345.202	8266303.622	8959766.844	8586526.681	2375696.351	5984256.067	3317463.304	8437557.864	21907263.28	11043715.78	5729824.453	21168137.58
9751925.26	14659537.52	9436255.461	7268531.96	8351429.078	8997811.061	11512893.29	8402870.007	6462088.626	864099.8541	4739196.978	5235128.617	4345249.753
22484431.01	29833864.03	26328550.09	63593154.17	65355917.94	34733902.86	30098632.72	28775487.94	30057914.99	22578556.15	22573300.96	33701585.89	36404548.9
288417.6412	333928.3351	239382.6417	2580234.367	2406234.699	2137793.659	2033903.866	1844690.639	9226135.852	8707899.153	9874255.247	8569013.152	9688346.401
4876606.176	3561289.308	2363243.34	10293608.59	5512584.961	5686049.862	4194198.114	21393669.99	25764448.92	29480824.36	19912300.35	53973290.07	34757251.73
25098568.23	38259298.92	43613331.76	14396128.84	19388343.27	19949594.24	21901806.54	21097869.89	26188061.54	15362707.38	25410845.7	12338838.58	37722770.69
60332942.4	66749733.78	54574340.12	46106860.95	49017899.37	60579035.93	45868523.25	61824657.42	50587117.83	97100758.58	57201964.69	42355712.7	56298955.49
218662202.5	238081795.3	248564174	43850082.52	40157931.46	55519462.42	35591800.27	56245626.91	26360323.48	18604564.59	18273773.95	16919242.79	16125076.6
12718829.77	12388145.67	7912379.411	18899034.79	14794044.8	9659933.633	6789015.198	10458940.34	14897735.19	111496852.6	10064255.53	11862244.59	15537913.01
148060.7908	159423.3998	155193.0341	437337.3481	282657.3491	135528.2979	143636.3962	216083.2165	136928.3764	1461544.871	144201.78	752593.1261	135770.8396
16823156.27	18433794.27	10587972.65	17992887.53	17062300.23	18386392.26	14852507.75	18451846.59	36937286.34	57273845.78	20210519.58	37024619.19	34979551.72
1763872.55	2222557.439	4581520.585	4643760.611	7685246.951	3076699.426	3668998.003	2300510.976	1388694.405	3477524.226	3241164.145	2995419.465	3365211.213
16713028.77	17000151.83	19073973.04	1357998.499	1550770.297	239139.3508	1018445.713	1297054.692	254204.5323	276961.4251	286540.0576	253924.5	289643.7206
70429097.67	86917593.56	20634996.94	43974953.74	17298516.65	5279419.358	15312518.84	8333617.592	34098865.41	567471935.9	139347378.3	43782229.37	183622964.6
1593972.463	1339990.818	1922738.472	3251862.25	2560489.91	2437836.758	1838847.703	2719002.424	4889619.852	7442248.392	4808771.424	6040614.514	6537641.554
658795921.8	828145883.7	546726595	626415861.7	651969579.7	569778009.1	631132691.3	553856725.5	417960952	442674965	396336736	377603468.8	365494658.6
591243.3058	378358.8456	348755.4234	3722175.189	1598176.829	1935713.087	1505696.562	1687231.405	2654218.938	3921138.825	2560149.483	4071847.625	4447434.482
8057332.137	9599946.592	7305318.527	63795706.57	66025836.44	30701019.47	24009607.88	24306169.52	64525328.44	168000407.3	92505368.15	57254731.08	87566736.6
10382828.4	12657754.81	9842216.134	13504053.3	7066194.752	3900546.341	2591902.273	4032506.321	67231845.42	93029068.09	44982832.54	48985890.05	88973261.23
10420426.33	11044720.11	7461562.596	17179811.58	12871729.74	6377578.103	5189405.316	6591130.148	10406615.28	107097137.3	8775839.742	8577292.535	10286574.14
9721732.919	7654794.541	9319064.312	13894949.28	13185820.02	8259343.673	9167390.119	8411847.555	7685827.139	17192457.74	9858584.525	8713813.258	12339630.3
16330803.3	16394256.18	17260973.78	12673845.87	13043577.22	13114653.01	10220861.26	9568160.344	9098151.882	86769371.55	31904262.1	8482798.976	51600747.84
53431797	70308619.68	25956085.56	42132280.19	47573254.01	44776420.73	52815173	42072822.52	13270433.48	6974226.619	7396472.794	11361055.08	17560804.01
3699464.063	4292228.319	3444645.868	8804985.141	9182420.857	9656293.937	10116678.29	8629512.172	16184219.93	106191448.7	21318524.83	14080930.01	30996152.97
1907338.364	4465660.786	529712.3697	2933713.856	2116504.492	1603619.078	783909.7521	25843416.36	4454968.239	8317552.107	2247070.264	38700050.58	5794539.222
7876319.208	11297751.47	2451602.842	5024320.347	3947817.042	4190036.953	3674232.643	6351931.595	9189240.133	13002251.18	3559234.64	10373763.48	13819499.84
2125784594	2224633318	2108690796	4321269737	3166938918	1966948192	2183984484	2138922089	1525481914	6694265898	1808711152	2520873349	2419098581
6007905.271	7503533.994	6240144.26	16435493.57	14268373.92	6486595.821	8127961.872	6695234.389	6329235.845	5018683.477	5490165.078	6072603.807	6731704.209
43154624.72	38494496.4	40011915.79	11443644.77	14053685.63	16459645.06	13348867.55	18916421.5	1929680.084	7980084.78	5215171.482	3160970.935	3620448.48
273669573.8	220214974	197232967	100165443.9	12148086.7	143496010.3	108737701.4	122154769	130645796	166782679.4	151180277.5	108245673.8	164005572.9
488196.7013	436310.3368	222408.8482	197034.8704	192004.6532	210402.1007	203848.7524	217968.9165	372575.1667	3377061.792	861822.5376	2941484.535	338406.4783

all_YP1_3	all_YP1_4	all_YP1_5	all_YP2_1	all_YP2_2	all_YP2_3	all_YP2_4	all_YP2_5	all_YP3_1	all_YP3_2	all_YP3_3	all_YP3_4	all_YP3_5
8876651.136	8059628.948	8472301.214	16517485.46	18567949.82	15809028.53	10672920.72	9510302.817	10820051.58	25588432.18	11105645.27	12443602.35	14358925.67
5591422.086	3942402.425	4375937.724	12654197.43	11035542.07	9871331.363	7929007.495	12174675.24	23827060.8	18833063.52	19347930.19	23643546.34	30443548.04
54582666.58	43899028.95	27992573.84	76020328.78	95939896.4	261918774.1	109824929.2	250913051.3	127971086	56097108.33	109566366.7	131898301.2	146152995.3
26959926.67	21444486.65	20548444.48	59566709.82	14759976.13	32066176.29	10198462.3	22934574.97	28519557.47	173102932.6	33077803.2	28006872.94	49320008.06
2888969.67	7399241.774	4117110.699	62683470.09	49634502.2	64566480.31	45534611.8	66581641.22	215266863	249296791.3	190092647.4	290096005.3	277159513.2
1101189.204	1687268.39	1138633.931	6331819.397	2135012.856	8239781.459	4110096.755	10271087.4	27271196.92	28268331.53	19178225.98	41921274.93	37739982.39
1607990.451	4201170.599	1632246.349	20645672.84	16532385.08	14557191.85	10469179.66	20395264.98	51617279.03	50773916.58	29408010.48	73642378.12	91733361.4
9174592.653	5205177.576	7776981.007	33416111.87	26126947.58	13032184.55	12253374.76	14324478.13	17386787.82	30501474.11	20247948.63	25456624.29	26177542.84
448749.9545	642515.4063	375961.787	939914.8759	611225.1317	380107.6062	783559.289	767839.8889	4039212.589	5559307.912	1851786.791	2305975.78	1808041.241
15569171.72	15100418.29	11561599.25	9172121.749	7524931.029	8660635.05	8160430.91	11627727.73	7959428.016	9957302.703	7750174.482	12805374.87	8660040.085
327088.3848	325108.0035	1061952.799	418576.7974	776147.5128	397456.9519	978076.106	321054.2429	304485.5695	315143.9992	313614.0871	320429.9717	337940.1057
2045646.897	2173065.294	2204255.808	7567506.203	9110072.397	9761756.184	6712047.469	9702558.026	25940043.73	10000288.71	22923454.23	17556435.34	12977261.72
4207097.076	6031897	7524036.383	31159853.66	31940264.01	11485252.94	38266438.05	11128355.26	18185427.22	7991943.121	17622487.8	17850444.58	16530266.81
410132.4954	447770.3372	566719.0985	666667.3711	796765.43	870634.45	692495.8369	784177.7066	886223.3948	767564.105	922695.2193	850436.4837	836484.6403
543875.1094	443639.4624	543197.884	545144.3248	392488.8061	409455.5309	415134.0105	540564.9428	1272115.975	12483928.58	964263.5288	1096437.677	1751878.923
322812.8532	281953.6474	359588.7398	264118.2124	258476.7957	264019.0681	283483.7484	286300.1674	1106101.537	11393212.27	470473.9452	675820.8443	942423.5864
13752338.25	13901771.14	12702239.22	17356093.09	14363177.49	5647044.208	7042728.073	7050355.688	8206818.831	58401552.87	7304754.874	8234401.002	8976437.55
2762963.058	4803502.383	826704.9664	2437082.226	1578901.193	1957898.452	1033523.333	3816971.078	3121033.94	6114343.108	1822782.551	4901124.59	2926848.189
903101.5604	1153009.272	1900153.05	1046881.931	911187.5622	2393848.435	1042858.528	1996793.704	2371343.942	2233483.399	2852595.291	1905431.003	2054061.355
3001777.622	4456716.965	357674.3913	457744.0913	268430.5446	311326.2834	297705.433	2636536.178	1080574.254	1392809.196	573570.9872	12702607.07	2236263.894
425895.5065	421960.3988	447049.7091	360229.6346	358488.8925	640242.9534	378611.1062	1575410.875	957964.1081	1279004.25	485239.068	4225335.863	1107409.428
635696.1513	397039.1051	1350230.3	892866.8364	403720.3993	474434.7426	536610.1645	1056219.964	2643002.615	1910910.605	1317802.804	2941039.202	6203247.94
5263239.187	5435108.917	6037925.055	14259507.45	11766923.12	7938828.216	6289639.934	6983532.09	8609009.235	32067468.17	3908489.288	7601834.765	5063164.996
52424891.13	50715791.97	42696957.07	45587147.09	54579386.62	16602143.75	47777892.91	136557087.2	60304821.75	37149523.81	23811274.39	52937584.79	43389796.1
30301940.55	21118760.96	13191500.71	10240812.62	8308079.924	13873411.68	15904172.95	16363125.16	13582454.04	2977330.476	9564803.122	15671231.4	11317566.46
169161.2802	180204.7202	172514.028	227849.7429	227840.2077	204922.0231	214696.4788	191011.81	924181.0055	629058.3651	331593.6934	485512.2077	490207.8648
7784616.293	6066103.097	6811405.473	7628263.863	4647251.985	3427383.886	6238648.552	4755486.218	20249787.36	21200669.45	21532181.49	14624799.66	20235156.21
20241853.72	20747447.63	23049876.33	19686915.34	17743843.25	18529906.37	19902356.38	19136068.34	20384317.01	29451091.56	26364560.54	21744887.41	22764060.38
66056379.15	72823686.75	22287343.13	25865661.47	28966755.25	24502217.58	20987583.34	26108509.12	22191975.29	6200708.006	23719993.73	17266426.4	20649129.34
8532681.711	8736848.221	7575283.965	17934922.01	11408462.5	7883645.43	8228920.538	8425239.121	170701050.7	112696141.1	178784980.7	53084655.36	130060167.2
811541.1302	896350.2902	302578.9743	3621652.949	2806599.7	1483022.161	2029045.074	1928656.21	1446341.539	1417283.351	922695.6983	2900270.847	1277736.114
236138.309	268681.7896	194556.1069	231293.8337	229529.2464	1184370.262	249654.8191	846390.9044	686733.8324	1922925.948	1547771.595	1760079.485	734608.8322
41919698.4	35218416.88	24164865.08	25650539.1	17695350.25	31750006.42	36016191.6	40251448	24391969.18	8056600.885	19634451.37	34177318.01	18922123.16
5490104.336	7148710.369	2984932.636	4369921.151	3956955.276	3256757.909	3337290.533	4200756.711	4438884.116	8864488.136	3441298.119	5989017.062	2880679.413
465101.5836	695202.8582	439690.53	2666182.071	2105345.867	1680760.862	1081695.692	1271922.62	1548140.409	3045567.082	1793473.528	1422707.245	1929895.113
32614169.13	33510218.23	16100482.87	53222272.62	72829064.23	55778683.64	52870966.98	49010433	41486097.26	5974742.092	31740376.54	40777996.84	34040008.69
267716956.5	255836503.6	205368455.3	208606125.9	232004527.2	199801513.5	206353528.2	198040904.4	276194260.2	1509203077	539353781.1	349772442.2	553383674.3
446944.6015	802878.0267	2244952.087	3925206.639	4143444.272	4910696.811	1422746.572	7360294.344	15624406.35	10688595.55	7659177.623	17382551.35	20468775.62
177955.4939	555251.4512	287218.0859	1116881.775	1753427.304	502473.794	378783.8004	913500.2152	144800.3354	2298937.418	527835.8625	850885.6413	403353.8742
336275.7211	384896.0781	724549.2547	367129.2204	360419.0415	356366.0778	238476.1984	253514.9594	237527.2294	9476395.537	438772.6285	382565.922	639588.175
2142048.232	2273557.117	3671553.167	1866967.707	2660320.233	4185574.01	2183490.484	6308079.23	4641876.786	3655169.987	4977974.307	6069841.733	4745967.291
716356.4615	3348729.414	486090.7729	1911804.884	1387567.577	714368.1058	298161.165	1181440.794	1097499.351	10515073	947790.6735	608611.7519	1849919.44
4482925.935	4562129.296	4367339.621	3380421.648	3601633.897	1247736.97	1896555.044	1026755.44	1349438.976	15156995.99	3280156.812	2113660.628	3622625.268
4472945.454	4906982.476	4225178.083	2333487.11	3114863.621	4850357.315	3060514.903	4164544.856	4931331.045	4772071.509	5653640.555	4314684.656	4313756.509
2261821.436	2873789.724	2439452.539	1532229.033	1655490.837	978272.06	1892207.177	1560787.293	1260616.413	853159.7918	1033552.611	874634.534	1714023.169
28131667.82	25238396.48	21745620.46	8483458.142	7329044.939	933882.6849	4515491.242	2565250.476	733104.4216	639372.0729	330603.2237	902519.7154	510402.8525
2650736.335	3246579.154	2478912.735	3967330.434	3395303.683	7451453.605	3280529.864	5402915.262	11411870.88	26157140.38	5955532.507	10142323.38	9986572.513
7745994.624	10603530.34	3992192.657	7600657.807	5728125.517	8969283.7	4781423.443	11612946.61	15186253.95	29934907.54	9028912.471	18288366.04	16529246.84
1553621.958	2280069.734	1064454.383	2093119.915	2012491.253	2920523.005	1607219.892	3643147.392	4674193.856	14483621.67	3102365.159	5862660.557	4827807.43
49403451.27	63189553.66	51889280.81	15390986.11	15909468.68	26929456.04	21120002.62	22948506.41	4091329.412	9259933.778	3109978.32	2545156.428	5022368.848
18950519.64	17005891.79	14806032.04	41767905.67	44831176.03	42562072.73	54201180.13	37188032.44	44084385.44	53969907.93	57789422.91	47913760.55	54944036.07
298854.7187	457684.6124	322333.6317	623166.5777	827759.0405	940524.0733	715840.9893	2749880.009	3125492.848	1645838.701	1386757.965	2929115.513	2953761.12
955951.4137	943091.5129	1190203.164	3966549.73	3427565.295	1258311.947	1501834.024	1526723.067	1119827.271	1504570.319	1255223.18	1437896.892	1425958.892

all_YP1_3	all_YP1_4	all_YP1_5	all_YP2_1	all_YP2_2	all_YP2_3	all_YP2_4	all_YP2_5	all_YP3_1	all_YP3_2	all_YP3_3	all_YP3_4	all_YP3_5
258522.2626	239306.694	260594.3011	290456.1286	193247.859	192679.1043	258653.4737	434025.8021	1884426.08	448226.1141	662712.3053	1100219.839	561719.6525
4672221.213	4097210.345	3019213.11	600846.1179	766824.0566	779971.1576	376993.4033	956906.7895	1041446.577	932139.5359	1133101.234	891198.37	1015097.641
4995361.741	6511105.557	8694919.88	4174089.895	3342022.557	2682505.278	3243862.33	3197470.938	2652003.831	7451726.604	4195259.546	2107439.429	3583913.656
3194173.182	6409421.958	6761650.849	5866351.809	6462675.719	6363315.724	15129473.47	8708246.764	7202711.39	4649729.977	7093607.151	5702005.834	3698896.458
1345641.875	8765043.757	10952400.22	1386347.916	1388538.12	215597.9136	910399.1441	777560.6825	246094.2069	223809.3962	286313.6213	236826.9655	263488.6052
1738376.621	1999399.935	387066.8827	7499459.733	11108680.8	4177362.624	1237529.836	6374632.293	2040145.368	2869863.088	3124454.226	4095801.343	6661058.452
507902.8989	622305.5583	475762.3678	672950.1852	426028.3668	429962.6127	374060.6651	527998.6365	1719296.841	12427186.98	445870.2896	557130.1248	1504163.805
50088541.29	61830809.16	68379080.32	76411132.2	80228491.37	86306555.76	11810640.26	69418810.68	44843011.78	3893246.87	31876593.83	52591162.92	35658355.99
1156032.742	1098963.334	1384695.996	2710803.928	2641224.657	1544652.141	3214264.876	2221777.342	3870469.866	2668472.618	3124586.938	3509924.724	4479794.437
794838.2497	811444.8892	553433.8788	1057422.626	644332.8789	649150.7419	567843.8275	396412.1856	598045.1464	625010.1278	889305.6547	549125.6445	691541.9047
145194.9817	144704.6983	395673.4132	533659.9335	116133.1142	114747.8196	390970.5589	117286.7208	152054.9425	370830.0282	129032.4634	112669.6305	153335.0549
16989208.76	1437767.116	17524870.42	12629302.21	13068040.77	14129568.09	1689753.012	13697492.56	12337324.55	3621545.927	13060218.66	12673535.34	4208943.37
13938700.18	13974366.82	19426898.57	18447486.95	19178279.76	18102647.88	17791242.32	16155263.77	31265596.69	85706540.47	43556393.73	23351119.77	31440009.57
2276898.445	3083049.913	1982739.002	1025989.056	1028445.926	985731.4112	949823.6482	1137577.057	1020406.763	3158038.365	1033271.458	947187.0338	997663.1454
302048.0013	411472.846	589099.0619	1044826.163	810051.3579	533477.9798	482860.5963	644067.5921	606351.3358	1695037.493	726179.3541	1185910.134	907592.0512
134326779.6	135049480.5	141852413.4	191885619.3	216551286.5	199972454.3	182981958.8	189135442.3	250349723.3	132647146.3	209019685.1	222313479.7	220890832.2
7226077.324	7257468.508	8109108.363	5520565.188	5185559.543	5999911.348	6170281.951	8156044.771	5059597.486	4332061.476	5324312.124	5122174.983	4551497.98
1804931.474	2066192.065	3584166.615	5965023.274	7008185.173	8266621.184	7903822.921	8221795.084	7722734.451	6830134.889	8766543.695	7943462.436	7717220.175
4638563.254	3328479.593	3178811.682	6598569.726	6489143.593	3932956.775	4426581.369	3555825.799	7527695.444	32624564.26	4785179.437	7038811.331	7877248.373
3593768.554	2979773.603	2961350.993	3443947.527	2839245.254	3226810.767	3611140.508	4097657.832	5391161.313	4532591.58	4593069.757	4306809.224	4355192.281
2458224.052	3655937.769	233844.5743	422712.221	289566.7749	429591.0995	305749.42	3685691.781	1100055.7	915780.4979	480462.0663	6028664.052	1377604.801
2033399.57	610841.7076	1543803.72	672072.3237	497004.2319	493881.9581	809474.599	1010036.338	573182.7057	534160.7842	483917.5121	749014.5759	582248.1146
1445026454	1506214164	1424549029	1024477663	809323001.4	739611336.5	811268559.7	802479264.2	771696004.5	1294010163	935880274.8	699876871.5	777944546.5
19938996630	2039793632	24408617812	14584061544	15074045057	19795151048	18201985156	19687709528	15858926109	20101174264	16434343859	15920689233	16167323397
2066164696	1899151874	1492595606	845830744.6	1087071752	1053010042	953665847.2	1103912790	104543313.5	9197352.526	31057374.18	27312077.99	33346330.47
1105714111	1149635064	1299876248	1206980657	1244869492	900530509.4	1378830230	861137660.3	954220799.4	1254266906	1097179954	887825498.3	1002648833
42546600.8	46184766.22	138428129.7	443145075.1	400126857.6	347964370.5	116917339.5	458537456.1	611347066.4	565692267.8	318459600.6	716752835.3	810527958
2238194222	2278346947	2479522177	5116765550	4992270465	5240089582	4552847431	5003007380	7981864648	9424415292	7188978786	7007201833	7944651660
538651836.9	650988982.1	708673073.6	800665184.6	709371010.3	639609245.2	842499144.4	595917971.9	656228078.2	859755957.1	1020777584	693743813.8	637695036.9
95271115.91	81067632.76	162875775.9	504443861.7	559926018.9	348528708.9	301920160	408039975.6	450708842.7	628344563.1	522403008	542701918.8	639581093.9
14540270.61	18722466.93	14483720.98	58737206.19	57731538.56	49689666.96	31308008.06	30903925.38	37708334.21	63834349.58	57100658.08	36732671.71	57938894.77
24311450.63	30502747.75	31856348.11	137489986.2	123145287.8	78439550.82	56233178.71	59182668.79	67046266.16	135926742.2	76782813.88	88907635.83	89943173.64
215233671.7	292094668.3	323600501.1	1250456170	999794731.7	665759218.3	567888222.6	444705176.8	598524179	870087847.1	733283442.3	485449519.8	804089138.7
24028509128	25569719931	33448264758	56488845113	52558180623	58380105165	45192223778	52910735977	69245949091	91782962286	71025354695	64896057450	76812620603
306643806.9	320310727.8	407548336.1	207452965.5	262420344.1	128023839.2	260069310.4	121884134.9	108616126.7	125002172.7	94942046.22	97891921.91	113282994.1
160003223	168061713.1	215014328.4	439530725.3	273921899.2	525919752.2	426827078.3	500141003.1	531235498.2	320696054.6	676783715.4	579211363.8	461437314.4
9423389.108	14374864.52	7102125.47	34567249.49	25195965.4	44551255.96	12164784.17	70812085.78	66639923.49	45910561.74	24691013.13	97839442.43	80306926.25
4012985.403	4967794.857	3558026.206	7732285.322	7197293.674	4799063.316	4634071.987	4242502.859	10371941.89	48640538.96	7322495.73	8745064.472	10041360.21
76817605.01	74436663.32	73346128.61	223408976.8	252113330.6	218913809	235443905.8	203593387.1	348537977.9	248646021.5	362265620.3	372680462.2	284903015.8
211736768.4	209869296.6	237117126.2	126007222.8	134262989.1	130159856.7	148606138.8	122770740	124918160.7	135638135.9	148273640.6	117674641.9	131340806.2
295931.1827	322944.0734	443067.6437	4732809.829	3721697.815	1521159.18	640544.8471	1825542.563	582529.0094	1620413.671	860434.6253	774040.9294	1302670.077
129201558.8	128753877.6	157541065.9	132663832.6	148451297.5	104065733.1	135695952.9	89350274.85	106177442	159978487.6	133037261	105445669.7	123838392.7
2312837525	3656723311	3205702842	12390656991	12344954035	7205974456	10280670403	6302836982	11115110715	9634819086	12685231649	10426900600	12447353657
52936730.13	51420855.07	53146460.67	27827601.25	29051732.53	11491642.49	35536163.71	12801622.7	10161153.16	13203159.36	11654717.08	9772692.75	10329370.83
20896211.83	86984703.83	26497948.01	61942869.52	42976599.72	31996626.36	29235860.58	51128876.77	59951222.17	204277808.9	35671465.44	83572204.74	61807730.7
1.34151E+11	1.3762E+11	1.34162E+11	1.16619E+11	1.24291E+11	1.27855E+11	1.23711E+11	1.29252E+11	1.1706E+11	1.15785E+11	1.16039E+11	1.19906E+11	1.18834E+11
1258515897	1553736916	1189179414	6835492236	5050791961	4142406489	2669997467	2560467899	2743988690	4879488979	3678613284	3913423754	4332202575
20994477.53	21588388.21	16315044.53	67975953.05	81122838.46	62473096.31	69480596.31	58610443.02	100809824.6	82505675.28	84905992.59	90069695.6	90494200.29
79683359.72	54974396.38	64710309.47	72205457.04	81563583.97	160279382.9	116424047.5	159366862.1	89083985.89	91903772.53	93604374.94	101101183.6	75523889.27
59289968.51	60023715.41	44031044.6	45966922.43	54279033.31	46524451.73	39845902.35	40640035.38	62117539.07	114609548	73048546.11	72936853.1	83879807.2
71825931.69	66762417.2	84312671.18	149903536.5	162068441.1	178549272	216945124.1	182488565.1	89153813.33	53883827.17	84573232.22	111403574.9	79159580.04
119890022.3	119528728.2	171900654.6	57601252.93	58935895.43	113848552.9	64160608.36	123647381.5	55508135.1	48484812.58	53902242.96	45225481.46	44718626.74
10705071.96	17448712.28	8389312.665	19109260.2	14061570.6	11278201.69	8215883.313	12997329.15	21877307.76	117663712	16086753.62	18985008.55	27306908.53

all_YP1_3	all_YP1_4	all_YP1_5	all_YP2_1	all_YP2_2	all_YP2_3	all_YP2_4	all_YP2_5	all_YP3_1	all_YP3_2	all_YP3_3	all_YP3_4	all_YP3_5
4553972.967	4385871.67	4143504.73	8484356.79	8745012.526	5179120.089	4261008.158	8570279.955	17188676.08	34977664.45	15272449.63	22048684.95	22549702.36
20341074.61	21458293.6	15931001.97	20587002.19	28125949.27	19724958.74	16352877.09	25319118.82	19897431.74	25467313.16	20620827.08	20230476.65	23369310.54
18038656.28	21909879.89	18965317.44	41358752.42	38393978.21	64700408.01	30789236.47	60255807.96	64557466.97	51022407.5	37872296.11	18495156.87	68142882.75
27805609.8	25961119.58	25068950.69	35713753.82	35404114.62	16135005.25	26042468.22	25514162.43	58827644.18	53382971.9	40015608.61	51164184.99	78448145.23
109496662.6	147822647.8	123192357.5	600968947.5	419287318.9	791961705.5	295525194.9	426237669.7	467670241.8	695616733.7	650498225.8	552023232.9	785640655.5
171805379.4	165078438	181116197.2	135413998	144036512.9	77230261.57	140156338.7	81256944.75	86558604.29	90483504.77	90581095.99	80504786.52	84920976.93
54770654.9	70839512.13	42119362.86	81211175.71	72485799.7	56624085.77	57101845.86	63650009.77	76141828.74	134087967.5	59883706.82	96961319.74	87941009.7
2224307.348	2947609.992	820391.5888	2199394.719	1335290.123	1621942.559	1195800.66	2234599.247	3809422.408	3809025.495	1284915.982	4286418.488	3691890.425
128755974.5	164105788.5	97264387.7	61416352.83	56382922.62	40522441.34	64508140.99	43465340.34	31120098.27	85865946.25	37086749.6	27408428.7	33368942.03
42566436.91	65466540.07	38986721.45	151231497.9	160610535.9	179971509.6	129660456.6	156526593	186120547.7	153250387.8	214215154.7	172202877	174843071.8
11283455.23	12288592.59	11557865.91	15262975.83	24658640.55	21070913.69	27128670.93	18973772.49	23864002.82	48659660.27	27321280.23	27301770.23	25141545.51
714491.8896	965180.295	729155.6737	1422928.285	570374.6209	825049.1172	492827.5044	823637.3371	1584319.315	9786612.951	860815.7447	1462749.29	2024458.767
31327761.42	24333359.06	19822782.7	9969415.179	9289436.196	9440289.731	18816865.91	11708542.89	11629043.07	34347819.46	10971375.35	10172796.27	12137583.09
110196738.1	92049687.13	80666564.51	38432268.52	32089523.24	101768228.1	35459517.8	105244710.8	129398068.3	186285856.7	124941164.5	126252705.1	139048903.9
271914303.1	269653230.1	352844263.2	503487194.1	429932619.6	288409423.3	359608285.2	311531602.9	292253517.6	745514400.8	329189289.5	439918405.8	343048711.3
154211433.2	196128326.9	263791635.7	163317275.3	173386210.1	221131754.5	207253621.7	222654941.1	186178784.7	253242673.7	196805335.4	190979182.2	188606754.9
21080524.09	1901365.389	629819.6832	1951993.095	1210912.617	805982.9326	484821.496	1407141.515	2476932.84	16108780.96	1946424.188	2765739.163	3892401.357
616356.9888	650975.9709	638932.803	7361822.384	7171800.439	3709067.449	1685625.767	5017355.27	8658320.862	7930085.816	6079546.457	12187548.47	10823626.65
13934811.44	20316738.78	8791485.922	13557196.28	11203742.58	9654463.117	7756965.614	12525575.47	11406668.89	9776279.483	9171285.357	13294901.66	10007224.09
3831901.408	4407826.513	4355936.232	4195838.563	7206783.686	15832747.82	8425569.238	13470269.62	16383104.59	23010319.72	15568504.59	12810520.35	18682663.98
178003890.6	177303860.5	189595916.4	102005585.2	51716433.47	132033280.1	109092553.7	124789565.8	117285559.4	142491077	175980967.7	128591689.8	142339506.8
856751972	1020204229	577664418.1	850140952.4	698216460.3	450617885.4	596341839.7	714940827.2	609864775.4	421580861.6	434583113.2	644350090.6	550558493.1
53423912.1	75652385.22	33401353.78	94239291.3	78983810.18	50396920.04	46436191.64	65255007.4	85288523.7	136380481.5	68773512.42	94176697.49	97564894.94
361953571.9	325966356.6	357028161.4	466252272.7	493232945	373983275.9	437844883	369295369.6	628719985.8	749135663.8	544244111.4	565543563.5	588823192.2
89542041.82	121794127.1	35810997.73	73545623.74	54568369.38	44133611.54	42930413.94	70166718.18	76154173	191065547.3	48012801.02	109559908.6	74959883.61
2833509.587	2577693.933	2439513.325	2385751.515	3375726.267	1421330.36	2425192.947	1332861.575	1602517.929	1269527.397	1581295.159	1434089.078	1403750.975
8640773.891	7863589.443	8579866.212	4633448.292	4431779.343	3363207.516	6149844.944	2980774.711	3248397.684	4016105.82	3619455.389	3551774.053	3174642.868
17051714.39	15598188.98	10910090.02	10480516.58	13194531.8	14085983.74	12496139.65	13004948.92	9250833.663	3748372.129	9066221.006	10496914.33	7502490.576
935916.9198	1078101.412	677849.235	656225.5375	775853.0058	260333.8413	837623.899	293958.8309	300377.1408	286218.6916	306191.3939	294628.6798	295195.9283
3662650.874	4360105.311	4308155.065	9801787.579	10800755.51	7403156.405	6126590.563	7129500.94	8589127.353	5082379.387	4991892.347	8385438.767	8482379.365
10256978.96	13807928.75	6057820.85	9668583.259	9710990.521	15523739.52	7355905.705	15860762.82	32960594.95	94054249.08	20297917.29	29813652.44	31356446.88
15098551.25	18311904.55	18177390.59	13792765	17977141.21	27019898.84	20802290.12	25842633.27	9252933.946	1218344.479	7573894.117	14810051.54	11492478.71
103139421.2	151669665.3	144659970.9	233920050.5	205654369.4	331164767.8	239864454.9	320191320.8	507137834.5	97426804.17	157485122.4	296684235.8	139249440.5
63016229.09	55509338.38	39219874.15	67008264	67836976.78	57248482.98	56853182.85	54364155.16	85921956.71	678862992.2	197259014.8	97790508.06	195283484.4
4165901.352	4725972.857	1239160.08	5615012.463	5259300.085	2914254.983	2640333.888	1657205.312	10518835.32	45747942.21	3877576.603	15109935.76	11077352.18
2264766.783	4092211.123	1711625.464	6235028.249	4109525.059	2500017.636	1577117.09	3084067.92	9047296.85	77807048.47	11045574.06	7533169.138	11386329.83
175310379.3	230260768	118293909.1	95430783.61	118748065.3	158795510.7	174406722.6	153983742.3	50849834.45	35610731.26	47677924.46	51603805.84	45876535.46
40473466.05	60145333.59	29486617.26	43585585.2	49281053.09	42239448.32	36916684.28	50363394	40911403.91	28125967.2	61626017.84	68257328.86	46104489.44
15103464.47	11636250.78	11526119.08	13534655.71	14373506.19	7604672.414	5914191.341	26162601.6	15962038.27	12748096.83	8694450.093	34500450.67	15186046.32
409119.7674	448648.6727	304583.9478	1001735.026	1116132.705	1123540.075	682176.2547	719567.7066	1480959.009	5144495.079	2409393.247	2152270.216	2082746.275
103777353.7	110997990.9	85835877.06	4859850.428	6965407.538	6134665.096	4618087.008	11350838.52	2574937.186	5170427.086	3484354.964	2831944.631	3119905.98
19582448.59	20231196.23	9724456.16	10416804.98	12366695.25	18055574.08	12973070.69	16838252.12	12999888.76	2702467.227	8822126.273	12461052.57	9995562.857
2878125.197	1445355.67	2024686.782	1063185.935	2553118.947	2832559.715	3277493.916	4236991.209	3123687.711	2867874.378	2737930.881	2334255.006	5480792.36
63348756	74876371.54	64715145.88	133061704.2	157668279.1	151679081	127960688.6	126810865.9	164423033	103208128.8	170326898.5	168673033.5	155971955.5
3468839.459	8924520.027	632276.5305	6247430.258	3515933.213	4262471.738	2105407.393	6825719.408	16026936.21	24291707.57	7314034.509	24163061.38	24680372.72
32982550.49	53381586.91	22151139.36	207551857.9	155232288.3	79673283.07	19809838.06	90982860.3	54700179.38	66981268.49	36356875.48	19788426.17	55045849.69
16169562.38	28547280.37	3532273.794	16267910.68	12049185.51	11637887.09	7069818.248	143090102.7	27199417.34	40110603.24	18643616.68	194009619.1	36237604.53
616350.7468	619079.8067	536789.1372	388296.2196	406061.8294	460823.5641	417573.2545	419387.6286	2982099.932	16847522.6	5472600.393	2446616.562	6201403.743
7644479.118	8780550.803	9990946.479	5152140.328	5613117.686	4297505.345	5872447.806	4228981.561	3604871.168	1112430.05	3391825.6	3618299.61	3292268.815
30825608.05	32087542.24	38766300.48	29931182.17	33412784.86	17458815.32	32827406.97	8833940.494	19084471.11	26241381.89	24036140.53	16545798	22647837.26
5621857.633	5538975.875	4790897.022	37292838.1	36588653.82	16193128.34	11337471.08	21037620.62	15398803.92	52435500.36	13466984.45	49722039.21	33274501.76
51554148.16	87834204.78	43384918.68	108161729.2	77416933.56	57356972.32	33869402.29	52655584.63	164028267.4	1592588187	147080943	113184322.1	229037852.3
51251521.12	51030866.55	41999905.54	207070690.2	222253443.8	194222988.9	217228604.7	181863639.8	137483596.8	29797347.36	184493846.4	185975691.6	161082409.9

all_YP1_3	all_YP1_4	all_YP1_5	all_YP2_1	all_YP2_2	all_YP2_3	all_YP2_4	all_YP2_5	all_YP3_1	all_YP3_2	all_YP3_3	all_YP3_4	all_YP3_5
140680.2022	144316.9391	137724.6697	234191.0155	172476.0834	264827.1855	146161.4638	435015.2269	263498.3471	453859.1375	337309.1537	224988.4594	348931.2998
9468677.374	11786436.2	5296040.391	7818340.02	7492973.353	13335268.95	7481349.329	20521230.71	16962920.25	22125090.59	11688396.76	25566684.66	15712889.55
5362827.298	6099188.775	4868736.865	10287083.38	7979274.381	4806613.213	6430119.292	5325743.311	6639136.771	12904479.19	4297519.33	6761769.131	6546508.601
19395611.57	16959224.9	46424328.11	15021970.52	7483380.244	23579625.48	14860960.68	10990659.58	32078309.45	7771777.121	18361061.13	8561548.908	23667635.06
2406846.252	3631994.108	1536715.011	4687489.197	3947412.304	3490960.789	2556620.535	3790328.843	4614673.984	30053709.66	3785970.279	4503799.455	5195635.506
29357252.09	32339509.03	27729063.7	14936838.28	16927581.74	4151493.04	18611116.31	5162523.234	3271077.876	3240070.687	3838221.359	3527914.509	3680157.61
15963464.99	17120045.37	14159793.61	27383007.04	24912000.54	18605337.94	21726671.03	22920687.41	22021354.51	17771622.29	15760268.05	35303932.37	21105152.76
8651997.23	10700241.73	9499273.044	10325572.36	14127900.03	11233850.8	12544228.64	8596393.029	11689356.36	103353947.2	10875411.1	10697538.09	13935626.11
2079409.988	2860081.171	4155887.745	13939503.06	16926584.38	4976901.881	17386845.81	5087727.33	950321.1316	4271238.521	8383033.443	8047689.653	7509494.896
14896711.7	8266932.64	10930158.85	19340451.53	24202748.54	28941071.48	25243504.51	27535714.49	57157761.22	35132512.47	38530829.58	32290914.39	37165300.03
63523773.84	62673551.08	70685854.22	50274826.74	53128859.07	56885801.84	43796949.66	57012766.43	38786812.47	37802422.72	35797906.47	36148086.85	39762867.37
5526811.028	3046846.692	4887679.297	1320309.318	946625.8263	1737983.209	1033808.591	2330325.839	488705.2299	1856652.913	690834.5113	344241.3749	468255.1683
45903916.04	39907256.77	40346302.25	90671054.37	78297478.5	58642074.92	66220596.84	64843920.38	119658130.4	14660913.87	145084478.9	133967309	146912705
11907685.21	16669183.43	7198369.692	31424047.24	29521326.01	12747774.28	9234264.727	15083560.74	74059132.43	434945791.5	76727465.84	54783771.53	127386299.4
215408.8842	178442.9583	230965.4069	1062230.532	860738.7123	204432.3434	181152.6877	318443.897	258256.7399	4173085.36	471993.5902	347921.9412	1554472.748
602014.5378	440083.2313	398252.2236	1836226.4	1512394.531	1067177.53	1419859.011	870065.9715	1777233.102	1490344.913	2170542.761	1483873.769	1864256.712
36616251.46	43507079.21	30439439.36	70468005	77134363.44	68809681.98	74125059.45	63244636.92	63747338.57	48377920.39	64913231.51	60915336.82	57349529.23
90609896.71	106331154.8	151513155.1	81643832.98	91996290.7	52876384.67	96239246.37	50562796.03	50905625.76	58163300.29	50280291.13	52696779.26	55886027.75
149308053.1	155418587.4	114642365.4	876042678.4	912626749.4	917012933.5	903297097.8	850003193.1	694906804.4	149665196.7	433726160.2	615959045.1	447129724.5
170187618.4	153013722.2	143416774.7	240747262.4	307759062.6	507174447.8	293629414.4	622495329.8	560737622.5	242509315.3	379724459.1	540552064	509273822.6
1797263.045	1713323.079	1656937.427	18936305.07	17771458.41	6617892.725	8521978.722	6113756.249	19789615.25	6017591.822	16170747.79	16147064.11	21935162.96
4995652.335	529573.2934	2826522.577	2579531.862	4766563.258	380232.7408	1657029.915	1755038.195	975409.2673	673603.8214	582489.7986	476699.2206	319942.0009
1180656.998	2260648.583	2138711.659	3854871.975	4357844.618	4188795.336	4189929.292	4095000.177	5195256.846	2991248.98	4502262.627	5145791.146	4462509.044
22737966.45	21683683.7	8687201.371	66803848.31	82280623.9	127577769.1	91060407.75	113525990	86572819.17	71190939.85	82236034.71	86099382.17	74408745.03
18932137.95	29968353.12	1856824.013	14893497.69	6728199.423	5948251.87	8273173.192	17862876.81	8794909.055	20129560.78	6290272.385	34493878.9	13617520.9
